# Design, Synthesis, and Antifungal Activity of Some Novel Phenylthiazole Derivatives Containing an Acylhydrazone Moiety

**DOI:** 10.3390/molecules28207084

**Published:** 2023-10-14

**Authors:** Yao Tian, Jinchao Shi, Xiaoqian Deng, Tingyu Yu, Yong Hu, Richa Hu, Yufeng Lei, Linhua Yu, Xiang Zhu, Junkai Li

**Affiliations:** 1 Engineering Research Center of Ecology and Agricultural Use of Wetland, Ministry of Education, Hubei Key Laboratory of Waterlogging Disaster and Agricultural Use of Wetland, College of Agriculture, Yangtze University, Jingzhou 434025, China; yaotien@163.com (Y.T.); shijinchao1996@163.com (J.S.); dengxaioqian1999@163.com (X.D.); y18672395703@163.com (T.Y.); yong251li@163.com (Y.H.); huricha1999a@163.com (R.H.); m15616436561@163.com (Y.L.); linhuayu531@sina.com (L.Y.); 2 Institute of Pesticides, Yangtze University, Jingmi Road 88, Jingzhou 434025, China; 3 National Key Laboratory of Green Pesticide, Key Laboratory of Green Pesticide and Agricultural Bioengineering, Ministry of Education, Guizhou University, Guiyang 550025, China

**Keywords:** synthesis, phenylthiazole, acylhydrazone, antifungal activity, structure–activity relationship, rice blast

## Abstract

Crop fungal diseases pose a serious threat to global crop production and quality. Developing new and efficient fungicides is an important measure to control crop diseases. Phenylthiazole was found to be an excellent antifungal skeleton based on our previous study on the structural optimization and biological activity of the natural product thiasporine A. To find new fungicides, 45 phenylthiazole derivatives containing an acylhydrazone moiety were designed and synthesized by the principle of active substructure splicing. Forty-two of the forty-five compounds are novel, except for compounds **E1**, **E14**, and **E33**. Their structures were structurally characterized by ^1^H NMR, ^13^C NMR, and HRMS. The antifungal activities of the target compounds against *Magnaporthe oryzae Colletotrichum camelliaet*, *Bipolaris maydis*, and *Sclerotinia sclerotiorum* were evaluated at 25 μg/mL. The bioassay results revealed that most of these compounds exhibited excellent antifungal activities against *M. oryzae* and *C. camelliaet* at 25 μg/mL. In particular, compounds **E4**, **E10**, **E14**, **E17**, **E23**, **E26**, and **E27** showed the inhibition rate of more than 80% against *M. oryzae*, with EC_50_ values of 1.66, 2.01, 2.26, 1.45, 1.50, 1.29, and 2.65 μg/mL, respectively, which were superior to that of the commercial fungicides Isoprothiolane (EC_50_ = 3.22 μg/mL) and Phenazine-1-carboxylic acid (EC_50_ = 27.87 μg/mL). The preliminary structure–activity relationship (SAR) results suggested that introducing methyl, halogen, or methoxy at the ortho-position of R^1^ and the para-position of R^2^ can endow the final structure with excellent antifungal activity against *M. oryzae*. The current results provide useful data for developing phenylthiazole derivatives as new fungicides for controlling rice blast caused by *M. oryzae*.

## 1. Introduction

Plant diseases caused by pathogenic fungi seriously hinder the normal growth of crops, resulting in a significant reduction in the yield and quality of crops [1,2]. For example, rice blast, caused by *Magnaporthe oryzae*, is one of the most destructive rice diseases worldwide, causing a loss in rice production by over 30% [3,4,5]. Applying fungicides to control crop diseases is one of the most conventional and effective measures [6]. However, due to the long-term use of traditional fungicides, the resistance of phytopathogenic fungi to fungicides has gradually increased [7,8]. At the same time, increasingly stringent environmental and toxicological regulations further restrict the types of pesticide varieties that are allowed to be registered [9]. Thus, these factors prompt us to continuously develop novel fungicides with high efficiency and low toxicity for the control of crop fungal diseases.

**Figure 1:**
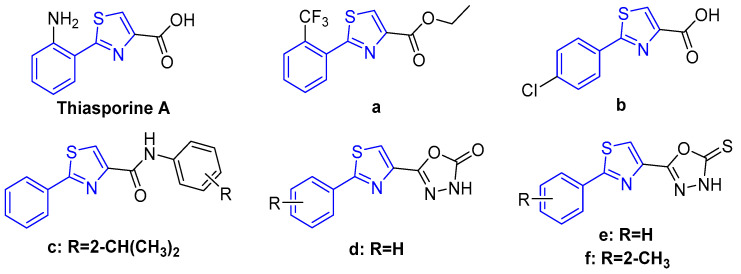
Structures of thiasporine A and its derivatives **a**–**f**.

Natural products have been regarded as an important source of potential lead structures in the discovery of novel green pesticides, due to their characteristics of wide biological activity, high structural diversity, and good environmental compatibility [10,11,12]. Thiasporine A (Figure 1), an interesting natural product containing a phenylthiazole moiety, was first isolated from the metabolites of marine-derived *Actinomycetospora chlora* SNC-032 by MacMillan in 2015, and its cytotoxicity against the non-small-cell lung cancer cell line H2122 was reported [13]. Some pharmacologists have focused on exploring the pharmacological activity of thiasporine A [14]. As part of our ongoing research on structural modification and bioactivity in the agricultural field of heterocyclic compounds found in natural products, a series of thiasporine A derivatives were synthesized, and their biological activities were systematically evaluated [15,16,17,18]. The results indicated that thiasporine A and its derivatives showed a wide range of bioactivities in the agricultural field, such as antifungal, insecticidal, and herbicidal activities [15]. It is particularly noteworthy that its derivatives exhibited excellent antifungal activities against some phytopathogenic fungi [16]. For example, its derivatives **a** and **b** (Figure 1) showed an inhibition rate of 82.3% and 90.6% against *Selerotium rolfsii* at a concentration of 100 μmol/L, respectively [17]. In addition, our group designed and synthesized some thiasporine A derivatives containing an amide (Figure 1, **c**) or a 1,3,4-oxadiazole (Figure 1, **d**–**f**) moiety. Some derivatives showed potent antifungal activities against some phytopathogenic fungi. For example, derivatives **e** and **f** showed significant antifungal activities against *Sclerotinia sclerotiorum*, with EC_50_ values of 0.22 μg/mL and 0.39 μg/mL, respectively, which were superior to that of the commercial fungicide carbendazim (0.70 μg/mL) [18]. The above results demonstrated that phenylthiazole is a promising antifungal skeleton for discovering new fungicides.

**Figure 2:**
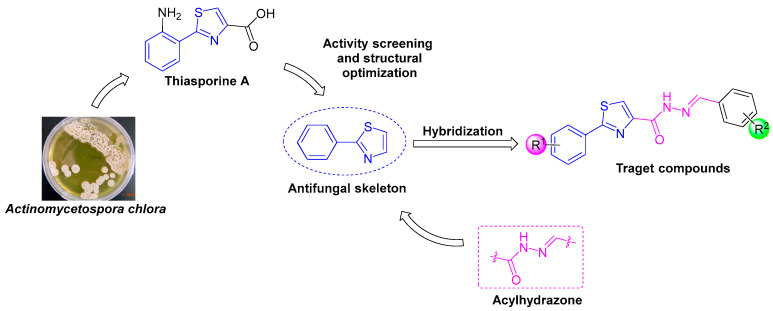
Design strategy of target compounds.

Acylhydrazone compounds possess a wide range of biological activities, such as antifungal [19,20,21,22,23,24], antibacterial [25,26,27], insecticidal [28], antiviral [29,30], and antitumor activities [31,32], which have been widely concerned in the field of pesticides and pharmaceuticals. At present, some compounds containing an acylhydrazone structure have been developed as pesticides or pharmaceuticals, such as benquinox (fungicide) [19,25,29], metaflumizone (insecticide) [20,23,29], and nitrofurazone (antibacterial drug) [31]. To extend our previous study and find new fungicides, in this study, a series of novel phenylthiazole derivatives containing an acylhydrazone moiety were designed and synthesized based on the principle of active substructure splicing. The design strategy of target compounds is shown in Figure 2. The antifungal activities of all synthesized compounds were evaluated against four destructive phytopathogenic fungi, and the preliminary structure–activity relationships were discussed.

## 2. Result and Discussion

### 2.1. Chemistry

**Scheme 1:**
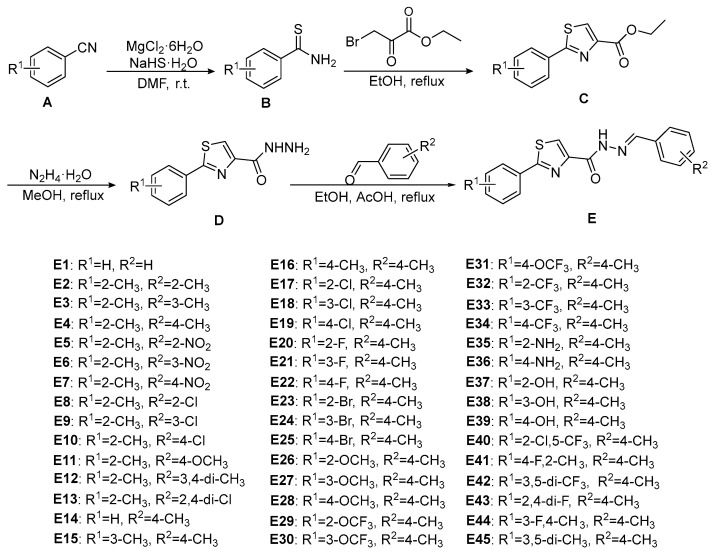
Synthetic route of target compounds **E1**–**E45**.

The synthetic routes of the target compounds **E1**–**E45** are depicted in Scheme 1. In brief, intermediate **B** was prepared using the corresponding substituted benzonitrile (A) as raw materials [33,34]. Subsequently, the reaction of intermediate **B** with ethyl bromopyruvate by ring-closure reaction afforded the corresponding substituted ethyl 2-phenylthiazole-4-carboxylate (intermediate **C**) [35]. As a key step, intermediate **D** was obtained by a classical hydrazinolysis reaction between intermediate **C** and hydrazine hydrate [36,37]. The target compounds **E1**–**E45** were prepared by the condensation reaction between the intermediate **D** and the corresponding benzaldehyde [19,21], which were obtained in yields varying from 61% to 78%. The structures of the target compounds were accurately characterized by ^1^H NMR, ^13^C NMR, and HRMS. All corresponding signals of protons and carbons were recorded in the ^1^H and ^13^C NMR spectra of the target compounds. The amide (CONH) signals of the target compounds in the ^1^H-NMR spectra appear as a single peak within the range of *δ* 12.20–*δ* 10.05 and the imine (N=CH) signals of the target compounds in the ^1^H-NMR spectra appear within the range of *δ* 9.05–*δ* 8.03. The carbonyl (C=O) and imine (N=CH) signals of target compounds in the ^13^C-NMR spectra appear as a single peak of about *δ* 157 and *δ* 149, respectively. More detailed information is provided in the Supporting Information.

### 2.2. Antifungal Activity

The preliminary antifungal activities of the target compounds against four phytopathogenic fungi (*M. oryzae*, *C. camelliaet*, *B. maydis*, and *S. sclerotiorum*) were evaluated at a concentration of 25 μg/mL by the mycelial growth rate method. The commercial fungicides IPT, PCA, and thiasporine A were employed as positive controls. The bioassay results are listed in Table 1. Most of the tested compounds demonstrated remarkable antifungal activities against the four phytopathogenic fungi compared with thiasporine A. Among them, compounds **E4**, **E10**, **E14**, **E17**, **E23**, **E26**, and **E27** exhibited excellent antifungal activities against *M. oryzae* with an inhibition rate of more than 80%, which was higher than that of the commercial fungicide PCA (44.79%), and the inhibition rate of compound **E26** (90.74%) was equivalent to that of the commercial fungicide IPT (91.81%). Meanwhile, the antifungal activities of compounds **E4**, **E10**, **E11**, **E20**, and **E26** displayed good antifungal activities against *C. camelliaet* with an inhibition rate of more than 50%, which was higher than that of IPT (35.57%) and PCA (46.98%). Regrettably, most of the target compounds had poor antifungal activities against *S. sclerotiorum* and *B. maydis*. Only compounds **E2**, **E3**, **E5**, **E6**, **E10**, **E19**, **E35**, **E39**, and **E44** showed moderate activities against *S. sclerotiorum*, with a growth inhibition rate range of 30% to 50%. Compounds **E4**, **E14**, **E26**, **E27**, **E28**, **E32**, and **E34** showed weak activities against *B. maydis*, with a growth inhibition rate range of 20% to 30%.

**Figure 3:**
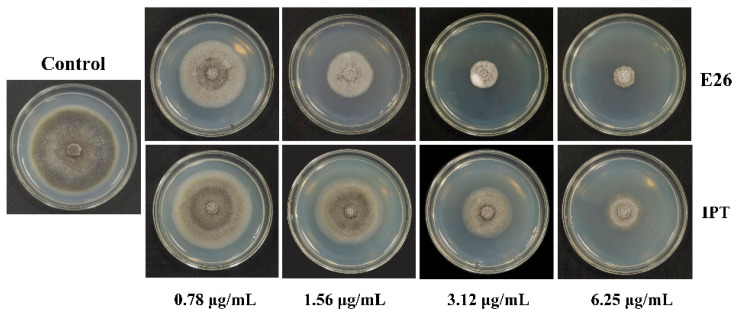
Antifungal activities of compound **E26** and IPT against *M. oryzae*.

Compounds **E4**, **E10**, **E14**, **E17**, **E23**, **E26**, and **E27** were chosen to determine EC_50_ values, because these compounds showed potent antifungal activities against *M. oryzae* with the inhibition rate of more than 80% at a concentration of 25 μg/mL. As shown in Table 2, all tested compounds showed excellent antifungal activities against *M. oryzae* with EC_50_ values in a range of 1.29 to 2.65 μg/mL, which were better than that of the commercial fungicides IPT (EC_50_ = 3.22 μg/mL) and PCA (EC_50_ = 27.87 μg/mL). In particular, compounds **E4**, **E17**, **E23**, and **E26** showed potent antifungal activities against *M. oryzae*, with EC_50_ values of 1.66, 1.45, 1.50, and 1.29 μg/mL, respectively. Figure 3 shows the mycelial growth of *M. oryzae* on a PDA medium containing different concentrations of compounds **E26** and IPT. It can be visually observed that the antifungal activity of compound **E26** against *M. oryzae* was more potent than that of IPT at the same concentration.

According to the antifungal activity results in Table 1 and Table 2, the preliminary structure–activity relationship (SAR) of phenylthiazole derivatives containing an acylhydrazone moiety were discussed. It revealed that the number, type, and position of substituents on the two benzene rings had a significant influence on antifungal activities against *M. oryzae*. On the one hand, it is beneficial to improve the antifungal activity of the compounds against *M. oryzae,* such as when methyl, halogen, or methoxy are introduced at the para-position of R^2^. For example, when R^1^ was ortho-methyl, the antifungal activities of compounds **E4** (R^2^ = 4-CH_3_), **E10** (R^2^ = 4-Cl), and **E11** (R^2^ = 4-OCH_3_) against *M. oryzae* significantly improved. On the contrary, the corresponding antifungal activity was knocked significantly down by introducing nitro or multi-substituted groups at different positions of R^2^. For example, compounds **E5** (R^2^ = 2-NO_2_), **E6** (R^2^ = 3-NO_2_), **E7** (R^2^ = 4-NO_2_), **E12** (R^2^ = 3,4-di-CH_3_), and **E13** (R^2^ = 2,4-di-Cl) failed to display antifungal activities. On the other hand, introducing methyl, halogen, or methoxy at para-positions of R^1^ is beneficial for improvement activity against *M. oryzae*. For instance, when R^2^ with the para-methyl group was kept, the compounds **E4** (R^1^ = 2-CH_3_), **E17** (R^1^ = 2-Cl), **E20** (R^1^ = 2-F), **E23** (R^1^ = 2-Br), and **E26** (R^1^ = 2-OCH_3_) appeared to retain excellent antifungal activities. Conversely, when trifluoromethyl, trifluoromethoxy, hydroxy, amino, or multi-substituted groups were introduced at different positions of R^1^, compounds **E29**–**E45** were enormously disadvantageous to the antifungal activities.

Satisfactorily, some novel compounds with potent antifungal activity against *M*. *oryzae* and the SAR results were obtained in this study. In our previous study, most phenylthiazole derivatives mainly showed excellent antifungal activities against *S. sclerotiorum* and *S. rolfsii* [15,16,18]. Only phenylthiazole derivatives bearing amide moiety showed good antifungal activity against *M. oryzae*. For example, derivative **c** (Figure 1) showed significant antifungal activity against *M. oryzae*, with an EC_50_ value of 4.84 μg/mL [17], which was lower than that of compound **E26** (EC_50_ = 1.29 μg/mL) in this study. The SAR results of these derivatives did not show significant similarity with the obtained results in this study. Furthermore, numerous studies reported that some acylhydrazone derivatives showed good antifungal activities against *Rhizoctonia solani* or *Botrytis cinerea* [19,20,21,22,23,24], and the selectivity and potency against pathogenic fungi of these acylhydrazone derivatives are in marked contrast to some target compounds. But the SAR results of some other acylhydrazone derivatives found that their antifungal activities were significantly influenced by the type and position of the substituent on the benzene ring of the acylhydrazone pendant, which have a slightly similar trend with the obtained results in this study [19,20,21,22,23,24]. Preliminary discussion results indicated that introducing an acylhydrazone moiety into a phenylthiazole skeleton can effectively improve the antifungal activity of phenylthiazole derivatives against *M. oryzae*. Regrettably, the mechanism of action of target compounds on *M. oryzae* is not clear in the present study. We will explore the mechanism of action of the target compounds after further optimizing the structure in future work.

## 3. Materials and Methods

### 3.1. Reagents and Analysis

Technical products of Isoprothiolane (IPT) and Phenazine-1-carboxylic acid (PCA) were provided by the College of Agriculture, Yangtze University. All chemicals and solvents were commercially obtained and used directly without further purification. Column chromatography was performed over pure silica gel 60 (200–300 mesh, Liang Chen Gui Yuan Co., Ltd., Anhui, China). Thin-layer chromatography (TLC) was performed on silica gel 60 GF254 (Qingdao Hai Yang Chemical Co., Ltd., Qingdao, China). The melting points of all target compounds were determined by using a WRR melting point apparatus (Shanghai Jingke Industrial Co., Ltd., Shanghai, China) and are uncorrected. ^1^H and ^13^C NMR spectra were recorded in DMSO-*d*_6_ or CDCl_3_ as the solvent using an AVANCE III HD 400 MHz spectrometer (Bruker Co., Ltd., Fällanden, Switzerland). Tetramethylsilane was used as the internal standard. High-Resolution Mass Spectra (HRMS) were obtained using electrospray ionization (ESI) technique by collision-induced dissociation on a Thermo Scientific Q Exactive instrument (Thermo Fisher Scientific, Bremen, Germany). The samples were dissolved in HPLC methanol and filtered by a 0.2 μm membrane. Then, it was infused using a syringe pump with a flow rate of 10 μL/min, and analyzed in the positive mode within a mass range *m*/*z* 100–1500.

### 3.2. Phytopathogenic Fungi

Phytopathogenic fungi, including *Magnaporthe oryzae*, *Colletotrichum camelliaet*, *Bipolaris maydis*, and *Sclerotinia sclerotiorum*, were provided by the Institute of Pesticide Research of Yangtze University. The fungi were maintained in a potato dextrose agar (PDA) medium at 4 °C.

### 3.3. Synthesis

#### 3.3.1. General Procedure for the Preparation of Intermediates **B**

Intermediate **B** was synthesized as previously described [33,34]. Starting material **A** (10 mmol, 1.0 equiv) and MgCl_2_·6H_2_O (11 mmol, 1.1 equiv) were added in *N,N*-Dimethylformamide (DMF, 50 mL), and the mixture was stirred for 20 min at room temperature. After being completely dissolved, NaHS·H_2_O (22 mmol, 2.2 equiv) was added to the mixture and stirred at room temperature for 16 h. The reaction process was monitored by TLC. After completion of the reaction, the reaction mixture was diluted with saturated salt water and extracted with ethyl acetate (3 × 100 mL). Subsequently, the organic phase was dried with anhydrous sodium sulfate, filtered, and condensed to obtain intermediate **B**.

#### 3.3.2. General Procedure for the Preparation of Intermediates **C**

Intermediate **C** was synthesized as reported method [35]. Intermediate **B** (10 mmol, 1.0 equiv) and 3-bromo pyruvic acid ethyl ester (10 mmol, 1.0 equiv) were added to ethanol (50 mL) and stirred for 4–6 h at 70 ℃. After the reaction was completed (monitored by TLC), the reaction mixture was adjusted to weak alkalinity and stirred for 0.5 h. After solvent removal, it was diluted with CH_2_Cl_2_ (3 × 100 mL), and extracted with saturated salt water. The combined organic extracts were dried with sodium sulfate anhydrous and condensed. The reaction products were purified by silica gel column chromatography (8:1 petroleum ether/ethyl acetate) to obtain intermediate **C**.

#### 3.3.3. General Procedure for the Preparation of Intermediates **D**

Intermediate **D** was synthesized as reported method [36,37]. To a solution of intermediate **C** (10 mmol, 1.0 equiv) in methanol (50 mL), 80% NH_2_NH_2_·H_2_O (100 mmol, 10.0 equiv) was slowly added at room temperature, and the resulting mixture was stirred for 4–5 h. After the reaction was completed (monitored by TLC), ice water (50 mL) was added to the mixture, and the precipitate was gradually generated. In the end, the suspension was washed with water several times, and filtered to give intermediate **D**.

#### 3.3.4. Preparation Procedure of the Target Compounds **E1**–**E45**

The target compounds **E1**–**E45** were synthesized as reported method [19,21]. To a 250 mL round bottom flask, a mixture of intermediate **D** (5 mmol, 1.0 equiv), corresponding substituted benzaldehyde (5.5 mmol, 1.1 equiv), a few drops of glacial acetic, and anhydrous ethanol (50 mL) were added and refluxed for 4-5 h. After completion of the reaction, the mixture was cooled to room temperature, and the precipitated residues were filtered and recrystallized from ethanol to obtain the purified target compounds **E1**–**E45**. The structures of the target compounds were accurately characterized by ^1^H NMR, ^13^C NMR, and HRMS. The structural characterization data of all target compounds are provided in the Supporting Information.

### 3.4. Antifungal Bioassay

The antifungal activities of target compounds **E1**–**E45** against four destructive phytopathogenic fungi (*M. oryzae*, *C. camelliaet*, *B. maydis*, and *S. sclerotiorum*) were evaluated by the mycelial growth rate method according to our previously reported procedures [18]. First, each tested compound was dissolved in DMSO and diluted to the corresponding concentration with 0.1% Tween 80 aqueous solution. The 5 mL solution was added to 45 mL of sterile molten PDA to obtain a final tested concentration. Then, PDA was poured into a 70 mm sterilized Petri dish with 15 mL per plate. The blank control was performed with 0.3% DMSO in sterile aqueous 0.01% Tween 80. The commercial fungicides IPT, PCA, and thiasporine A were employed as positive controls. The mycelial disks (diameter = 7 mm) cut from subcultured PDA dishes were inoculated in the PDA medium. The inoculated PDA dishes were incubated at 26 ± 2 °C. Each treatment was tested in triplicate. After the mycelium diameter of the blank control reached 55 mm–60 mm, the mycelium diameters (mm) of each sample were accurately measured by the cross-bracketing method. The growth inhibition rates were calculated according to the following formula. Each treatment was tested in triplicate. The growth inhibition rates and the standard deviations were calculated by Microsoft Excel 2016 software.

Inhibition rate (%) = [(*C* − *T*)/(*C* − 7 mm)] × 100 (1)

where *C* is the average colony diameter of the control blank, *T* is the average mycelium growth diameter of treatment, and 7 mm is the diameter of mycelial disks.

Selective compounds with good antifungal activities were further evaluated for their median effective concentration (EC_50_) values. According to the above-mentioned procedures and the screening results, a series of the tested concentrations of the compounds were set and their antifungal activities were evaluated by accurately determining the inhibition rate against the corresponding fungi. The log dose–response curves allowed for the determination of the EC_50_ value by using Data Processing System (DPS) (Version 20.05) software.

## 4. Conclusions

In summary, 45 phenylthiazole derivatives containing an acylhydrazone moiety were designed and synthesized based on the principle of active substructure splicing. Forty-two of the forty-five compounds are novel, except for compounds **E1**, **E14**, and **E33**. All target compounds were structurally characterized by ^1^H NMR, ^13^C NMR, and HRMS. The antifungal activities of the target compounds against *M. oryzae*, *C. camelliaet*, *B. maydis*, and *S. sclerotiorum* were evaluated at a concentration of 25 μg/mL. The bioassay results indicated that most of the synthesized compounds exhibited good antifungal activities against *M. oryzae* and *C. camelliaet*. In particular, compounds **E4**, **E10**, **E14**, **E17**, **E23**, **E26**, and **E27** possessed excellent antifungal activities against *M. oryzae*, with EC_50_ values of 1.66, 2.01, 2.26, 1.45, 1.50, 1.29, and 2.65 μg/mL, respectively, which were superior to that of the commercial fungicides IPT (EC_50_ = 3.22 μg/mL) and PCA (EC_50_ = 27.87 μg/mL). The preliminary structure–activity relationship (SAR) results suggested that introducing methyl, halogen, or methoxy at the ortho-position of R^1^ and the para-position of R^2^ can endow the final structure with excellent antifungal activity against *M. oryzae*. Compound **E26** emerges as a new lead compound for further structural optimization. The current results provide useful data for developing phenylthiazole derivatives as new fungicides for controlling rice blast caused by *M. oryzae*.

## Figures and Tables

**Table 1:** Antifungal activities of the target compounds **E1**–**E45** against four fungi at 25 μg/mL.

Compound	Mean Inhibition Rate ± SD (%) (*n* = 3)			
	*M. o* ^a^	*C. c*	*B. m*	*S. s*
**E1**	<10	29.19 ± 2.49	<10	15.98 ± 0.82
**E2**	<10	21.14 ± 0.9	<10	31.66 ± 0.35
**E3**	<10	19.46 ± 0.65	<10	38.17 ± 0.91
**E4**	**88.25** ** ± 0.17**	60.74 ± 0.23	29.51 ± 0.95	22.78 ± 0.4
**E5**	25.2 ± 1.08	24.83 ± 2.42	12.5 ± 1.7	37.57 ± 1.91
**E6**	<10	<10	<10	38.17 ± 0.91
**E7**	<10	19.46 ± 0.47	<10	15.38 ± 1.78
**E8**	<10	16.44 ± 2.61	<10	22.49 ± 0.46
**E9**	15.94 ± 1.6	16.78 ± 0.54	<10	27.81 ± 1.33
**E10**	**85.75 ± 0.8**	66.44 ± 2.28	<10	34.32 ± 0.62
**E11**	78.63 ± 0.76	68.12 ± 1.71	19.44 ± 0.64	21.89 ± 1.25
**E12**	21.99 ± 2.86	10.4 ± 1.56	<10	28.4 ± 1.47
**E13**	<10	10.74 ± 2.03	<10	12.13 ± 0.45
**E14**	**84.00 ± 2.86**	44.3 ± 1.63	22.92 ± 0.24	15.38 ± 1.78
**E15**	71.41 ± 2.15	<10	17.36 ± 0.63	17.46 ± 1.82
**E16**	64.35 ± 1.41	28.52 ± 2.06	12.85 ± 0.49	19.82 ± 1.28
**E17**	**81.40 ± 0.34**	33.22 ± 2.79	13.89 ± 1.45	23.08 ± 0.12
**E18**	<10	<10	12.85 ± 0.49	15.68 ± 1.37
**E19**	<10	11.07 ± 2.59	<10	35.5 ± 1.45
**E20**	74.16 ± 2.25	52.01 ± 0.88	15.63 ± 0.16	<10
**E21**	<10	<10	13.89 ± 2.9	20.12 ± 2.76
**E22**	56.01 ± 1.49	28.19 ± 1.87	15.97 ± 2.45	21.6 ± 0.47
**E23**	**80.12** ** ± 0.87**	37.25 ± 0.83	<10	<10
**E24**	<10	18.12 ± 5.12	12.85 ± 0.49	<10
**E25**	<10	11.07 ± 1.01	11.11 ± 1.21	<10
**E26**	**90.74 ± 0.74**	68.12 ± 1.27	30.21 ± 1.77	18.64 ± 0.81
**E27**	**83.32 ± 0.76**	45.64 ± 0.31	26.04 ± 0.77	25.15 ± 2.6
**E28**	<10	19.46 ± 1.27	22.22 ± 2.24	<10
**E29**	<10	<10	13.54 ± 0.14	<10
**E30**	<10	<10	<10	14.2 ± 1.84
**E31**	<10	24.5 ± 2.4	18.40 ± 3.18	15.09 ± 0.82
**E32**	69.03 ± 1.23	28.19 ± 1.15	24.31 ± 1.93	<10
**E33**	<10	<10	16.32 ± 2	<10
**E34**	<10	<10	20.49 ± 1.22	14.2 ± 1.57
**E35**	<10	<10	12.5 ± 1.7	34.62 ± 0.9
**E36**	<10	<10	14.93 ± 1.49	<10
**E37**	<10	21.81 ± 2.95	17.71 ± 1.23	22.49 ± 1.99
**E38**	<10	<10	10.76 ± 0.61	<10
**E39**	<10	<10	11.11 ± 1.53	45.56 ± 1.16
**E40**	<10	<10	14.93 ± 2.07	28.7 ± 1.25
**E41**	66.90 ± 1.53	31.54 ± 1.09	15.28 ± 1.5	<10
**E42**	<10	<10	<10	<10
**E43**	<10	20.47 ± 2.2	<10	<10
**E44**	<10	<10	<10	35.5 ± 1.62
**E45**	15.73 ± 0.56	<10	13.89 ± 3.13	<10
**Thiasporine A**	<10	<10	10.76 ± 1.48	16.27 ± 1.44
**IPT** ^b^	91.81 ± 0.72	35.57 ± 1.34	<10	75.15 ± 1.48
**PCA** ^c^	44.79 ± 1.38	46.98 ± 0.89	81.94 ± 1.65	97.63 ± 0.57

^a^ *M. o: M. oryzae*, *C. c*: *C. camelliaet*, *B. m*: *B. maydis*, *S. s*: *S. sclerotiorum*. ^b^ Commercialized fungicide IPT, ^c^ Commercialized fungicide PCA.

**Table 2:** EC_50_ values of selected compounds against *M. oryzae*.

Compound	Regression Equation	R	EC_50_ (μg/mL)	95% Confidence Interval (μg/mL)
**E4**	y = 4.4560 + 2.4582x	0.99	1.66	1.4621~1.8953
**E10**	y = 4.8513 + 1.2331x	0.98	2.01	1.5059~2.6873
**E14**	y = 4.1548 + 2.3913x	0.99	2.26	2.0124~2.5302
**E17**	y = 4.7745 + 1.3753x	0.98	1.45	1.1513~1.8211
**E23**	y = 4.8359 + 0.9221x	0.99	1.50	1.1483~1.9762
**E26**	y = 4.7570 + 2.1660x	0.99	1.29	1.1741~1.4018
**E27**	y = 4.2101 + 1.8652x	0.98	2.65	2.2483~3.1269
**IPT** ^a^	y = 4.6250 + 1.0746x	0.99	3.22	2.8494~3.6391
**PCA** ^b^	y *=* 1.6473 + 2.2696x	0.99	27.87	26.5822~29.2249

^a^ Commercialized fungicide IPT, ^b^ Commercialized fungicide PCA.

## Data Availability

Data are contained within the article and Supplementary Materials.

## Supplementary Materials

The following supporting information can be downloaded at: https://www.mdpi.com/article/10.3390/molecules28207084/s1, The structural characterization data of target compounds **E1**–**E45**.

## Sample Availability

Samples of the compounds are available from the authors.

## References

[1] Zhang C., Zhao C., Zheng H., Li L., Zheng Y., Wu Z. (2023). Design, Synthesis, and Study of the Dual Action Mode of Novel *N*-Thienyl-1, 5-disubstituted-4-pyrazole Carboxamides against *Nigrospora oryzae*. J. Agric. Food Chem..

[2] Chen L., Zhao B., Fan Z., Hu M., Li Q., Hu W., Li J., Zhang J. (2019). Discovery of novel isothiazole, 1, 2, 3-thiadiazole, and thiazole-based cinnamamides as fungicidal candidates. J. Agric. Food Chem..

[3] Yoon M.-Y., Kim Y.S., Ryu S.Y., Choi G.J., Choi Y.H., Jang K.S., Cha B., Han S.-S., Kim J.-C. (2011). In vitro and in vivo antifungal activities of decursin and decursinol angelate isolated from *Angelica gigas* against *Magnaporthe oryzae*, the causal agent of rice blast. Pestic. Biochem. Phys..

[4] Xu S., Wang B., Li L., Zhou Q., Tian M., Zhao X., Peng J., Liu F., Chen Y., Xu Y. (2020). Effects of camptothecin on the rice blast fungus *Magnaporthe oryzae*. Pestic. Biochem. Phys..

[5] Asibi A.E., Chai Q., Coulter J.A. (2019). Rice Blast: A Disease with Implications for Global Food Security. Agronomy.

[6] Ngo M.T., Han J.W., Yoon S., Bae S., Kim S.-Y., Kim H., Choi G.J. (2019). Discovery of new triterpenoid saponins isolated from *Maesa japonica* with antifungal activity against rice blast fungus *Magnaporthe oryzae*. J. Agric. Food Chem..

[7] Wang R., Du S., Wang J., Chu Q., Tang C., Zhang Z., Yang C., He Y., Li H., Wu T. (2021). Design, synthesis, and antifungal evaluation of Luotonin a derivatives against phytopathogenic fungi. J. Agric. Food Chem..

[8] Phillips M.W.A. (2020). Agrochemical industry development, trends in R&D and the impact of regulation. Pest Manag. Sci..

[9] Sparks T.C., Lorsbach B.A. (2017). Perspectives on the agrochemical industry and agrochemical discovery. Pest Manag. Sci..

[10] Gao Y., Xu R., Gu S., Chen K., Li J., He X., Shang S., Song Z., Song J. (2022). Discovery of natural rosin derivatives containing oxime ester moieties as potential antifungal agents to control tomato gray mold caused by *Botrytis cinerea*. J. Agric. Food Chem..

[11] Kang J., Gao Y., Zhang M., Ding X., Wang Z., Ma D., Wang Q. (2020). Streptindole and its derivatives as novel antiviral and anti-phytopathogenic fungus agents. J. Agric. Food Chem..

[12] Lorsbach B.A., Sparks T.C., Cicchillo R.M., Garizi N.V., Hahn D.R., Meyer K.G. (2019). Natural products: A strategic lead generation approach in crop protection discovery. Pest Manag. Sci..

[13] Fu P., MacMillan J.B. (2015). Thiasporines A-C, thiazine and thiazole derivatives from a marine-derived *Actinomycetospora chlora*. J. Nat. Prod..

[14] Liu Y., Ma Z., Zhao X., Shan Q., He P., Du Y., Wang Y. (2019). Simple and Efficient Synthesis of Anithiactins A-C, Thiasporine A and Their Potent Antitumor 2, 4-Linked Oligothiazole Derivatives. ChemistrySelect.

[15] Shi J., Chen S., Zhu X., Zhang Y., Li J. (2021). Synthesis and Bioactivity of Thiasporine A and Its Analogues. Agrochemicals.

[16] Zhang Y., Shi J., Hu Y., Liao C., Wang M., Zhu X., Yu L., Li J. (2023). Synthesis and fungicidal activity of the *N*-(phenyl) thiazolamide derivatives. Chin. J. Syn. Chem..

[17] Chen S., Zhu X., Shi J., Wang M., Hu C., Liao C., Zhang Y., Li J. (2022). Design, synthesis and fungicidal activities of Thiasporine A analogues. Chin. J. Pestic. Sci..

[18] Shi J., Tian Y., Chen S., Liao C., Mao G., Deng X., Yu L., Zhu X., Li J. (2023). Design, synthesis and antifungal evaluation of phenylthiazole-1, 3, 4-oxadiazole thione (ketone) derivatives inspired by natural thiasporine A. Pest Manag. Sci..

[19] Xu R., Gu S., Chen K., Chen J., Wang Y., Gao Y., Shang S., Song Z., Song J., Li J. (2023). Discovery of rosin-based acylhydrazone derivatives as potential antifungal agents against rice *Rhizoctonia solani* for sustainable crop protection. Pest Manag. Sci..

[20] Liu Y., Song H., Huang Y., Li J., Zhao S., Song Y., Yang P., Xiao Z., Liu Y., Li Y. (2014). Design, synthesis, and antiviral, fungicidal, and insecticidal activities of tetrahydro-*β*-carboline-3-carbohydrazide derivatives. J. Agric. Food Chem..

[21] Malik M.A., Al-Thabaiti S.A., Malik M.A. (2012). Synthesis, structure optimization and antifungal screening of novel tetrazole ring bearing acyl-hydrazones. Int. J. Mol. Sci..

[22] Zhang J., Yang R., Li L., Liu J., Liu Y., Song H., Wang Q. (2022). Design, synthesis, and bioactivity study of novel tryptophan derivatives containing azepine and acylhydrazone moieties. Molecules.

[23] Chen L., Xie J., Song H., Liu Y., Gu Y., Wang L., Wang Q. (2016). Design, synthesis, and biological activities of spirooxindoles containing acylhydrazone fragment derivatives based on the biosynthesis of alkaloids derived from tryptophan. J. Agric. Food Chem..

[24] Zhang J., Li X., Dong Y., Qin Y., Li X., Song B., Yang X. (2017). Synthesis and biological evaluation of 4-methyl-1, 2, 3-thiadiazole-5-carboxaldehyde benzoyl hydrazone derivatives. Chin. Chem. Lett..

[25] Zhang J., Wei C., Li S., Hu D., Song B. (2020). Discovery of novel bis-sulfoxide derivatives bearing acylhydrazone and benzothiazole moieties as potential antibacterial agents. Pestic. Biochem. Phys..

[26] Zhou Z., Zhou T. (2018). Synthesis and antibacterial activity of C-7 acylhydrazone derivatives of dehydroabietic acid. J. Chem. Res..

[27] Lu Y., Zhao Z., Chen Y., Wang J., Xu S., Gu Y. (2021). Synthesis and biological activity of pyridine acylhydrazone derivatives of isopimaric acid. J. Asian Nat. Prod. Res..

[28] Ren Z., Lv M., Sun Z., Li T., Zhang S., Xu H. (2021). Regioselective hemisynthesis and insecticidal activity of C8-hydrazones/acylhydrazones/sulfonylhydrazones coumarin-type derivatives of osthole. Bioorg. Med. Chem. Lett..

[29] Wang Z., Xie D., Gan X., Zeng S., Zhang A., Yin L., Song B., Jin L., Hu D. (2017). Synthesis, antiviral activity, and molecular docking study of trans-ferulic acid derivatives containing acylhydrazone moiety. Bioorg. Med. Chem. Lett..

[30] Ni W., Song H., Wang L., Liu Y., Wang Q. (2023). Design, Synthesis and Various Bioactivity of Acylhydrazone-Containing Matrine Analogues. Molecules.

[31] Kassab A.E. (2023). Anticancer agents incorporating the *N*-acylhydrazone scaffold: Progress from 2017 to present. Arch. Pharm..

[32] Shin S.Y., Lee J., Ahn S., Yoo M., Lee Y.H., Koh D., Lim Y. (2021). Design, synthesis, and evaluation of 4-chromenone derivatives combined with *N*-acylhydrazone for aurora kinase A inhibitor. Appl. Biol. Chem..

[33] Seitz T., Fu P., Haut F.-L., Adam L., Habicht M., Lentz D., MacMillan J.B., Christmann M. (2016). One-pot synthesis of 5-hydroxy-4 *H*-1, 3-thiazin-4-ones: Structure revision, synthesis, and NMR shift dependence of thiasporine A. Org. Lett..

[34] Guo J., Hao Y., Ji X., Wang Z., Liu Y., Ma D., Li Y., Pang H., Ni J., Wang Q. (2019). Optimization, Structure–Activity Relationship, and Mode of Action of Nortopsentin Analogues Containing Thiazole and Oxazole Moieties. J. Agric. Food Chem..

[35] Li Z., Chen Y., Zhou Z., Deng L., Xu Y., Hu L., Liu B., Zhang L. (2019). Discovery of first-in-class thiazole-based dual FFA1/PPARδ agonists as potential anti-diabetic agents. Eur. J. Med. Chem..

[36] Liu L., Tan C., Fan R., Wang Z., Du H., Xu K., Tan J. (2019). I_2_/TBHP-Mediated tandem cyclization and oxidation reaction: Facile access to 2-substituted thiazoles and benzothiazoles. Org. Biomol. Chem..

[37] Thore S.N., Gupta S.V., Baheti K.G. (2013). Docking, synthesis, and pharmacological investigation of novel substituted thiazole derivatives as non-carboxylic, anti-inflammatory, and analgesic agents. Med. Chem. Res..

