# Supplementary material for: Design, Synthesis, and Antifungal Activity of Some Novel Phenylthiazole Derivatives Containing an Acylhydrazone Moiety
Source: Molecules. 2023 Oct 14;28(20):7084. doi: 10.3390/molecules28207084 (PMC10608836; doi:10.3390/molecules28207084)
Supplement: Supplementary file 1 [file molecules-28-07084-s001.zip › molecules-2610361-supplementary.pdf]

Supporting Information

Design, Synthesis, and Antifungal Activity of Some Novel Phenylthiazole Derivatives Containing an Acylhydrazone Moiety

Yao Tian [1,2], Jinchao Shi [1,2], Xiaoqian Deng [1,2], Tingyu Yu [1,2], Yong Hu [1,2], Richa Hu [1,2], Yufeng Lei [1,2], Linhua Yu [1,2], Xiang Zhu [1,2,3,*] and Junkai Li [1,2,*]

1 Engineering Research Center of Ecology and Agricultural Use of Wetland, Ministry of Education, Hubei Key Laboratory of Waterlogging Disaster and Agricultural Use of Wetland, College of Agriculture, Yangtze University, Jingzhou 434025, China; yaotien@163.com (Y.T.); shijinchao1996@163.com (J.S.); dengxaioqian1999@163.com (X.D.); y18672395703@163.com (T.Y.); yong251li@163.com (Y.H.); huricha1999a@163.com (R.H.); m15616436561@163.com (Y.L.); linhuayu531@sina.com (L.Y.)

2 Institute of Pesticides, Yangtze University, Jingmi Road 88, Jingzhou 434025, China

3 National Key Laboratory of Green Pesticide, Key Laboratory of Green Pesticide and Agricultural Bioengineering, Ministry of Education, Guizhou University, Guiyang 550025, China

** Correspondence: xiangzhu1992@yangtzeu.edu.cn (X.Z.); junkaili@sina.com (J.L.)*

Table of Contents

1. The structure and data of target compounds (**E1-E45**) **2**

The structure and data of target compounds (E1-E45)

Compound **E1**, *N'*-benzylidene-2-phenylthiazole-4-carbohydrazide:

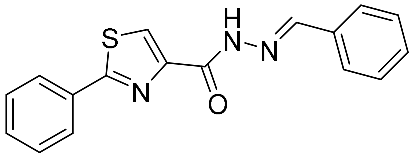

white solid, yield 77%, m.p. 217.6-219.5 °C; ^{1}H NMR (400 MHz, DMSO-d_6) δ 11.80 (s, 1H), 8.65 (s, 1H), 8.50 (s, 1H), 8.13 (dd, J = 6.4, 2.8 Hz, 2H), 7.80 – 7.71 (m, 2H), 7.60 – 7.43 (m, 6H). ^{13}C NMR (101 MHz, DMSO-d_6) δ 168.03, 157.42, 149.83, 149.39, 134.76, 132.86, 131.38, 130.70, 129.72, 129.36, 127.68, 127.12, 126.14. HRMS(ESI) calcd for $C_{17}H_{13}N_3OS$ [M+H]$^+$: 308.0852, found 308.0850.

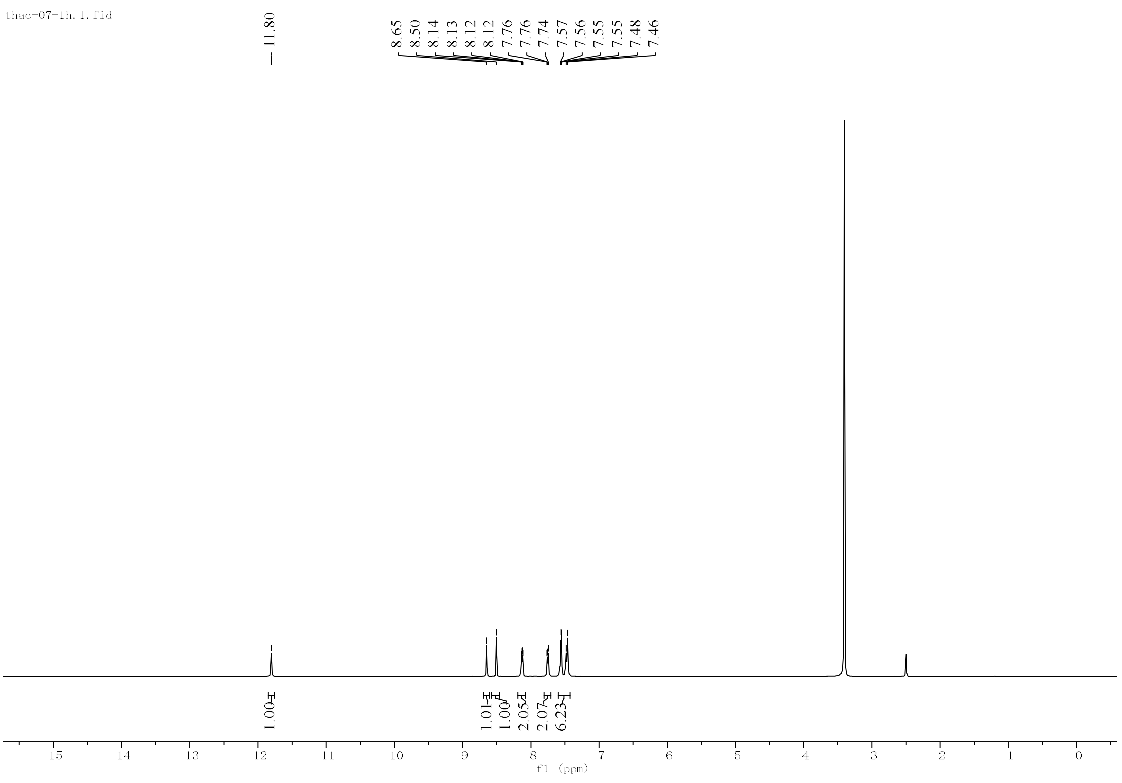

The ^{1}H NMR spectrogram of compound **E1**

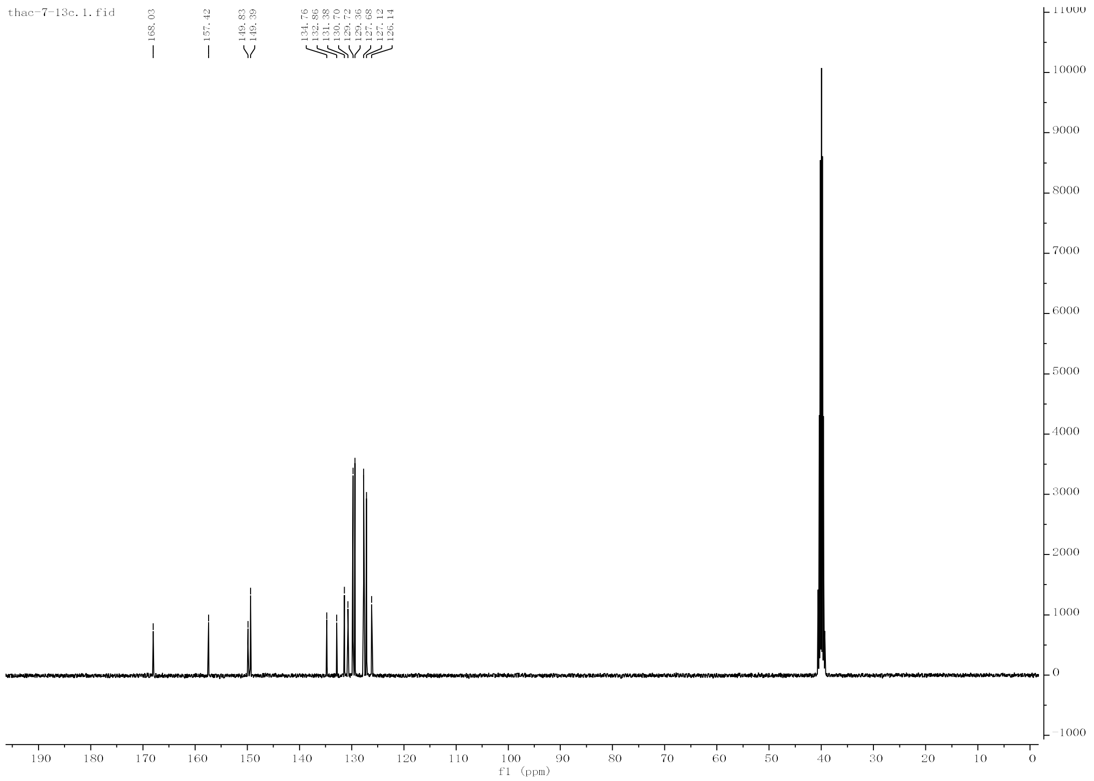

The ^{13}C NMR spectrogram of compound **E1**

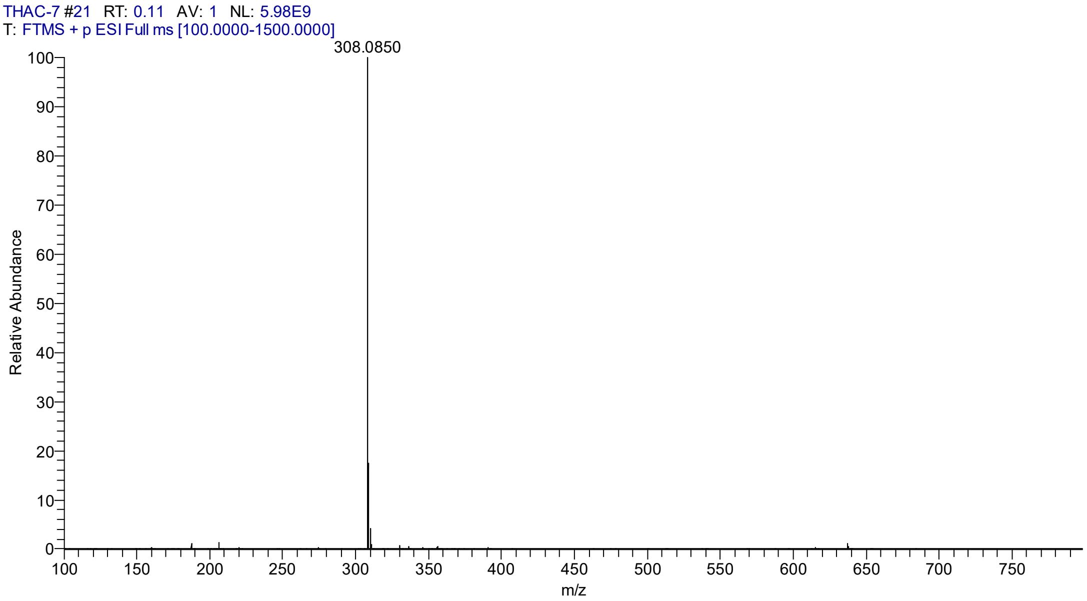

The HRMS spectrogram of compound **E1**

Compound **E2**, *N'*-(2-methylbenzylidene)-2-(*o*-tolyl)thiazole-4-carbohydrazide:

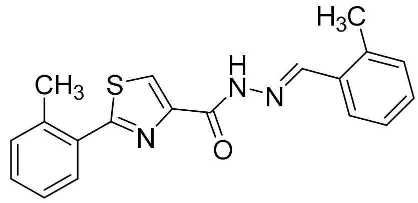

white solid, yield 66%, m.p. 198.1-199.6 °C; ^{1}H NMR (400 MHz, DMSO-d_6) δ 11.77 (s, 1H), 8.92 (s, 1H), 8.58 (s, 1H), 7.88 (d, J = 7.2 Hz, 2H), 7.48 – 7.23 (m, 6H), 2.59 (s, 3H), 2.44 (s, 3H). ^{13}C NMR (101 MHz, DMSO-d_6) δ 167.53, 157.49, 149.40, 148.01, 137.60, 136.70, 132.83, 132.41, 131.91, 131.32, 130.64, 130.36, 130.32, 126.85, 126.78, 126.63, 126.34, 21.53, 19.49. HRMS(ESI) calcd for $C_{19}H_{17}N_3OS$ [M+H]$^+$: 336.1165, found 336.1163.

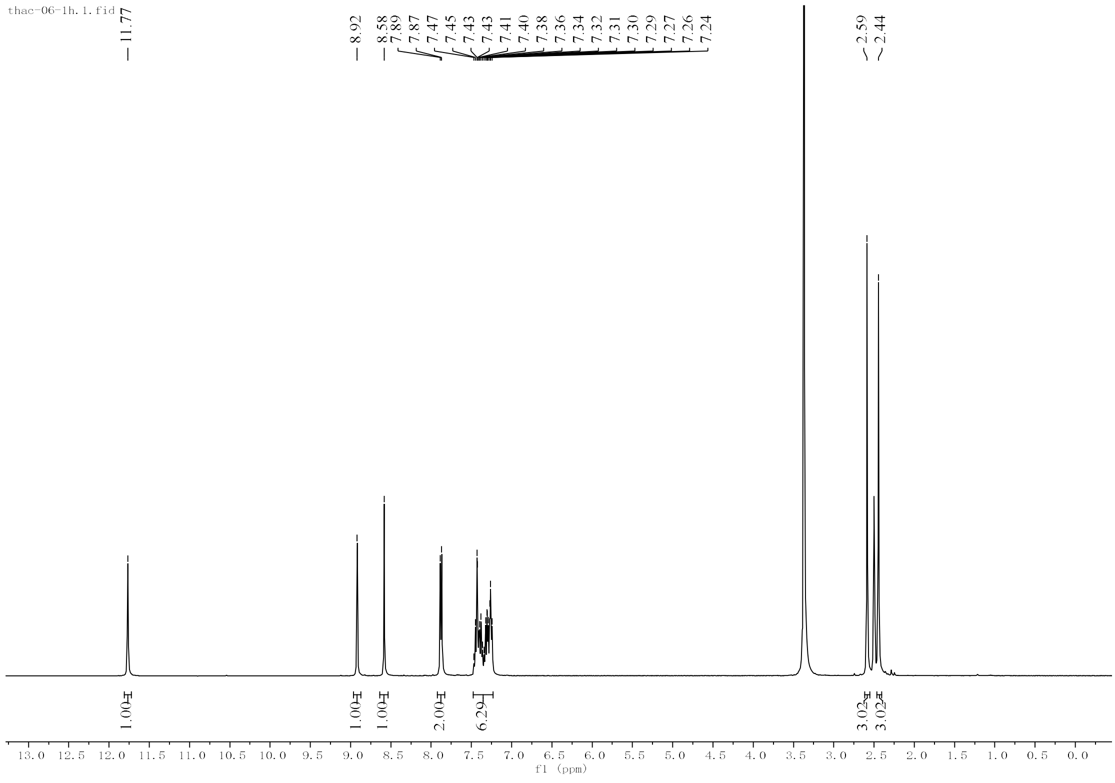

The ^{1}H NMR spectrogram of compound **E2**

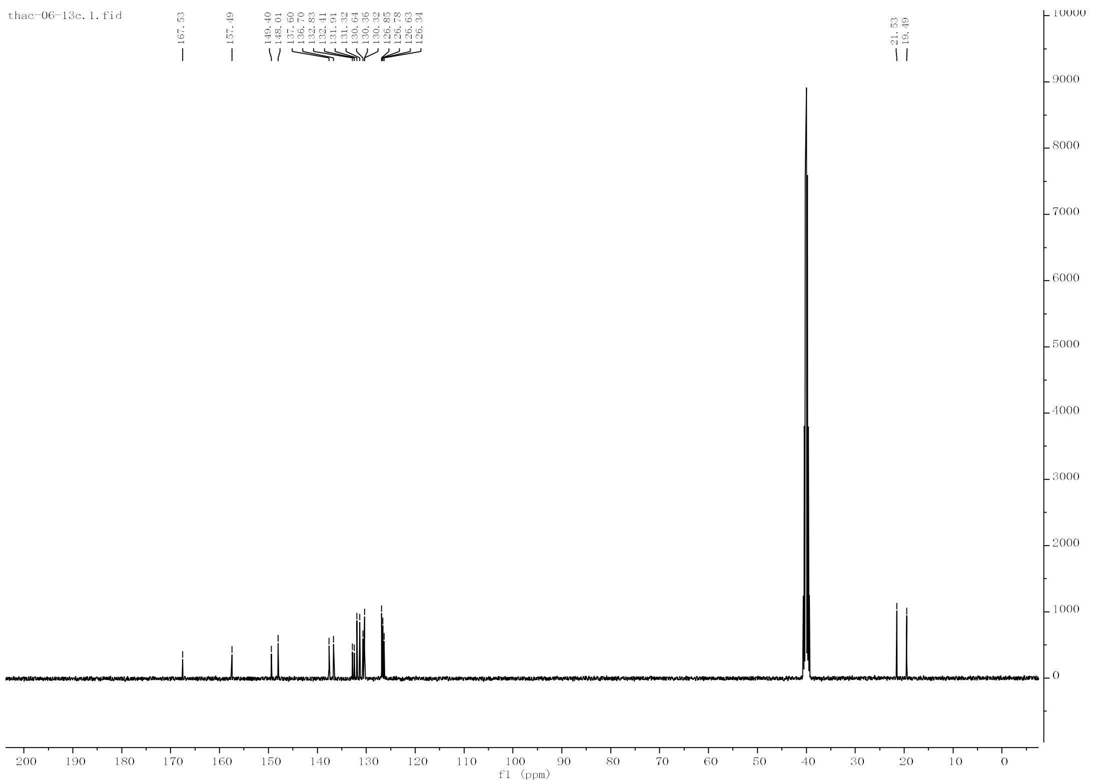

The ^{13}C NMR spectrogram of compound **E2**

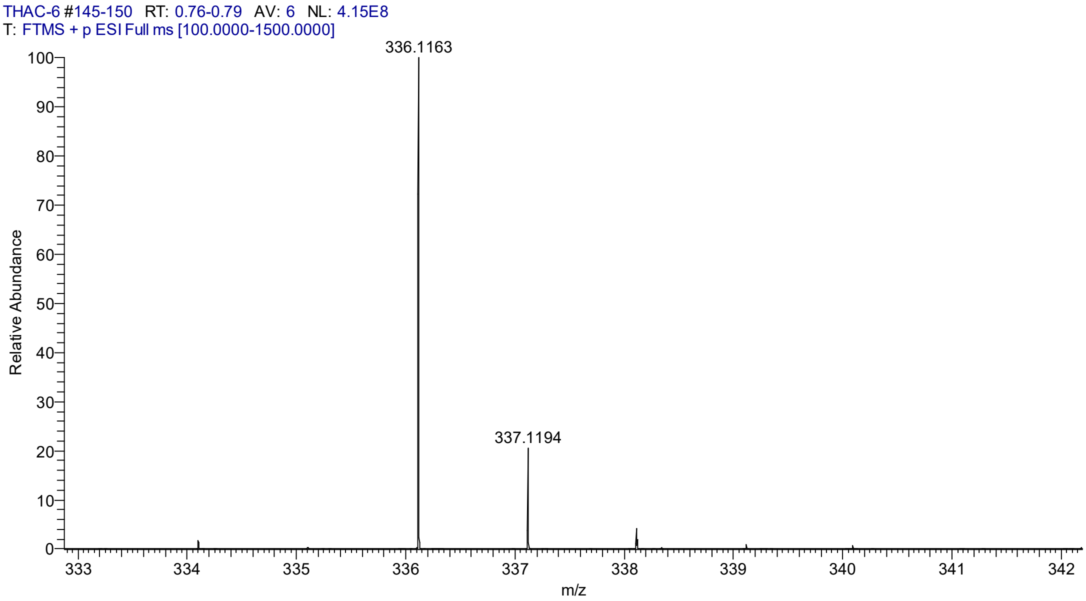

The HRMS spectrogram of compound **E2**

Compound **E3**, *N'*-(3-methylbenzylidene)-2-(*o*-tolyl)thiazole-4-carbohydrazide:

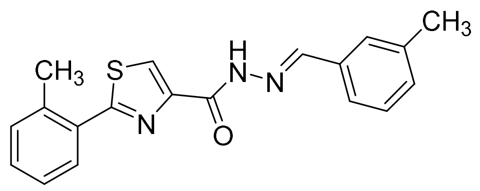

white solid, yield 71%, m.p. 150.5-153.1 °C; ^{1}H NMR (400 MHz, DMSO-d_6) δ 11.73 (s, 1H), 8.58 (s, 2H), 7.88 (d, J = 7.6 Hz, 1H), 7.57 (s, 1H), 7.50 (d, J = 7.6 Hz, 1H), 7.40 (dq, J = 30.0, 7.2 Hz, 4H), 7.26 (d, J = 7.6 Hz, 1H), 2.59 (s, 3H), 2.36 (s, 3H). ^{13}C NMR (101 MHz, DMSO-d_6) δ 167.55, 157.53, 149.48, 149.37, 138.57, 136.70, 134.75, 132.35, 131.94, 131.37, 130.63, 130.35, 129.22, 127.83, 126.84, 126.73, 125.16, 21.58, 21.36. HRMS(ESI) calcd for $C_{19}H_{17}N_3OS$ $[M+H]^+$: 336.1165, found 336.1164.

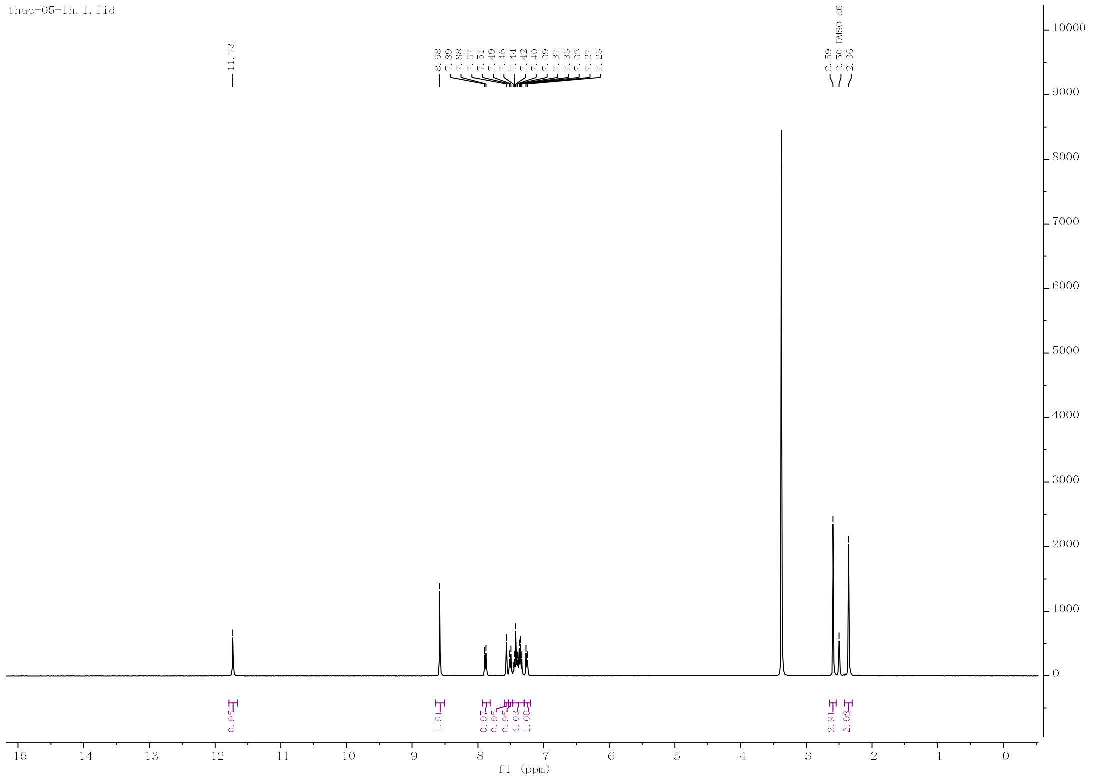

The ^{1}H NMR spectrogram of compound **E3**

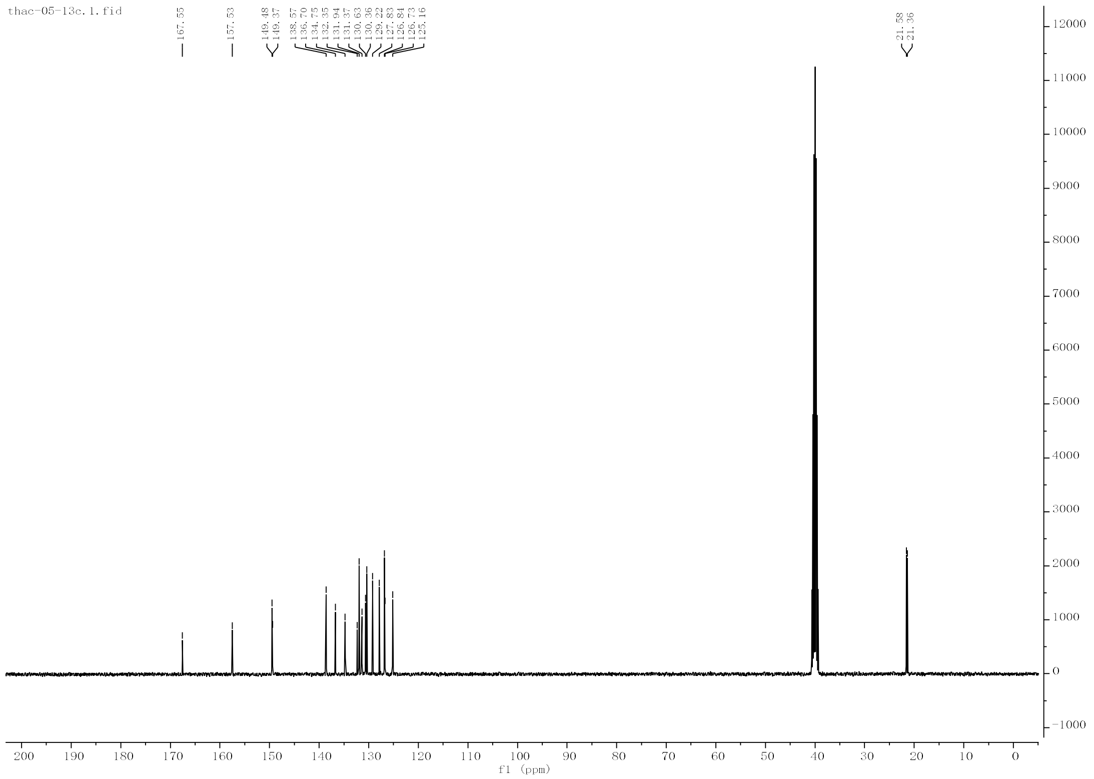

The ^{13}C NMR spectrogram of compound **E3**

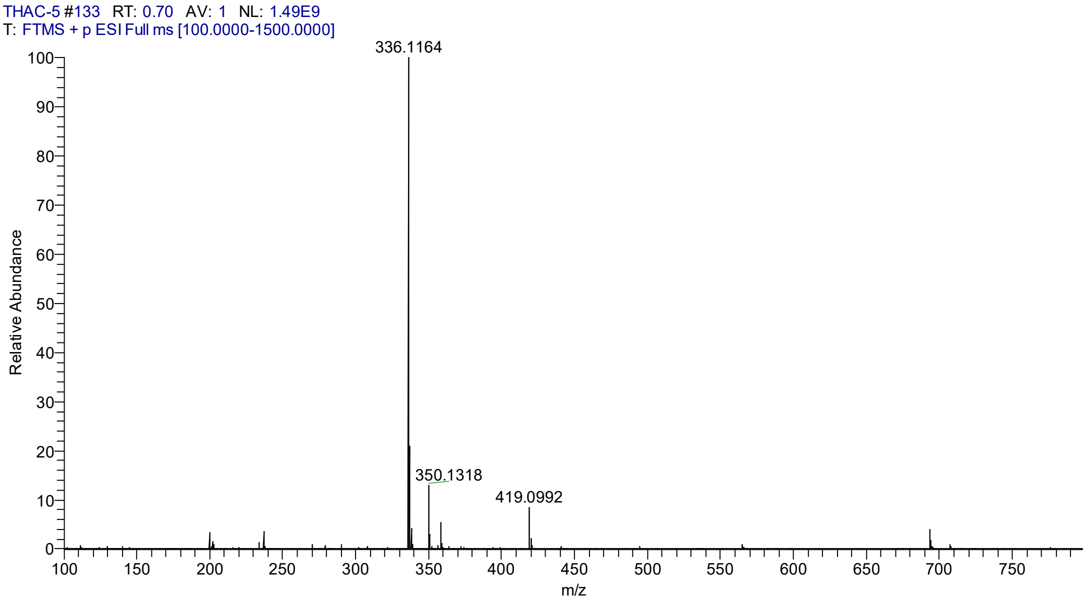

The HRMS spectrogram of compound **E3**

Compound **E4**, *N'*-(4-methylbenzylidene)-2-(*o*-tolyl)thiazole-4-carbohydrazide:

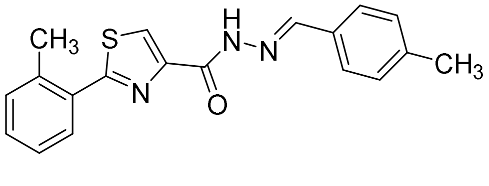

white solid, yield 70%, m.p. 146.6-148.6 °C; ^{1}H NMR (400 MHz, $CDCl_3$) δ 10.32 (s, 1H), 8.32 (s, 1H), 8.24 (s, 1H), 7.71 (t, J = 6.8 Hz, 3H), 7.35 (tt, J = 14.8, 7.6 Hz, 3H), 7.22 (d, J = 8.0 Hz, 2H), 2.62 (s, 3H), 2.38 (s, 3H). ^{13}C NMR (101 MHz, $CDCl_3$) δ 168.17, 157.14, 148.95, 148.83, 140.97, 136.59, 131.99, 131.67, 130.94, 130.19, 130.00, 129.43, 127.85, 126.35, 125.00, 21.52, 21.49. HRMS(ESI) calcd for $C_{19}H_{17}N_3OS$ $[M+H]^+$: 336.1165, found 336.1162.

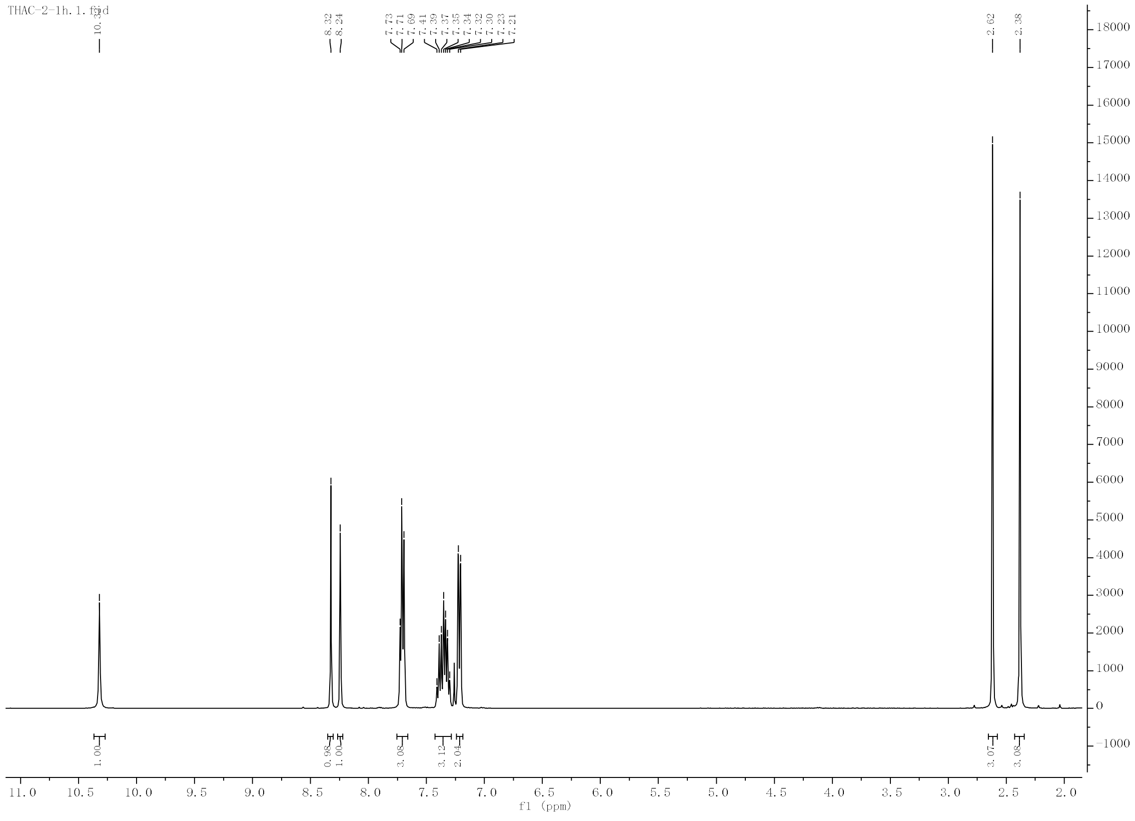

The ^{1}H NMR spectrogram of compound **E4**

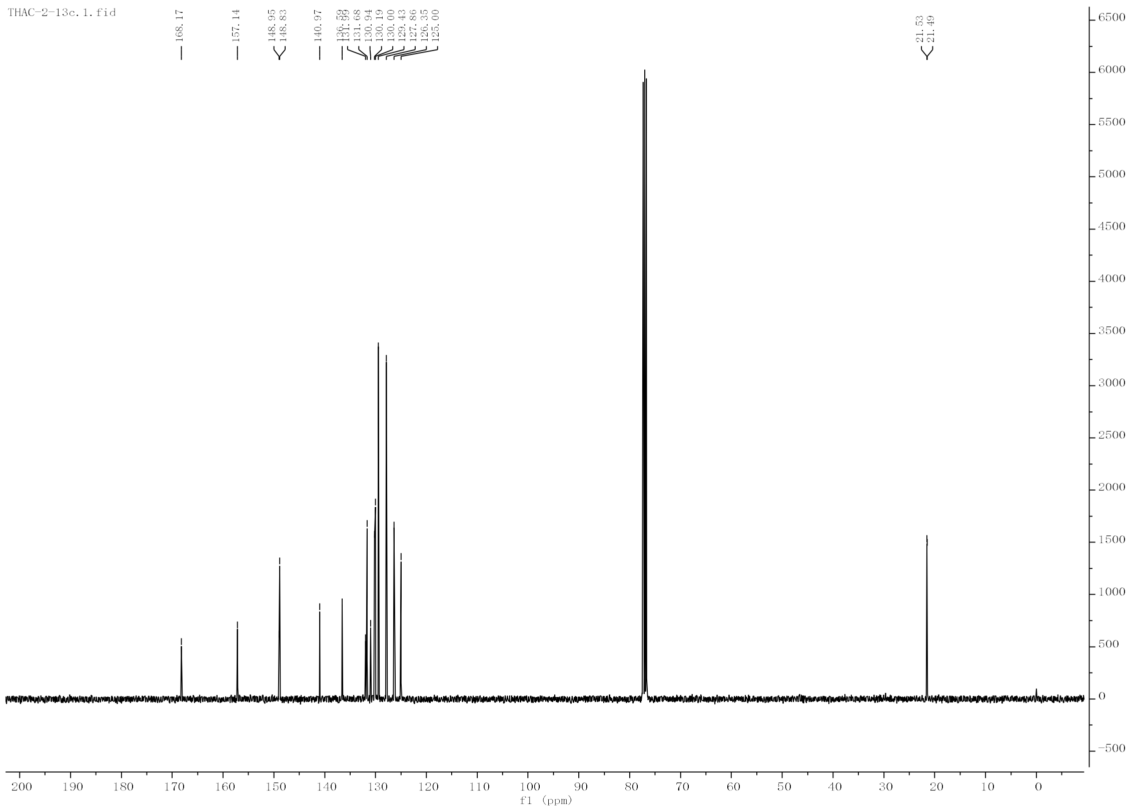

The ^{13}C NMR spectrogram of compound **E4**

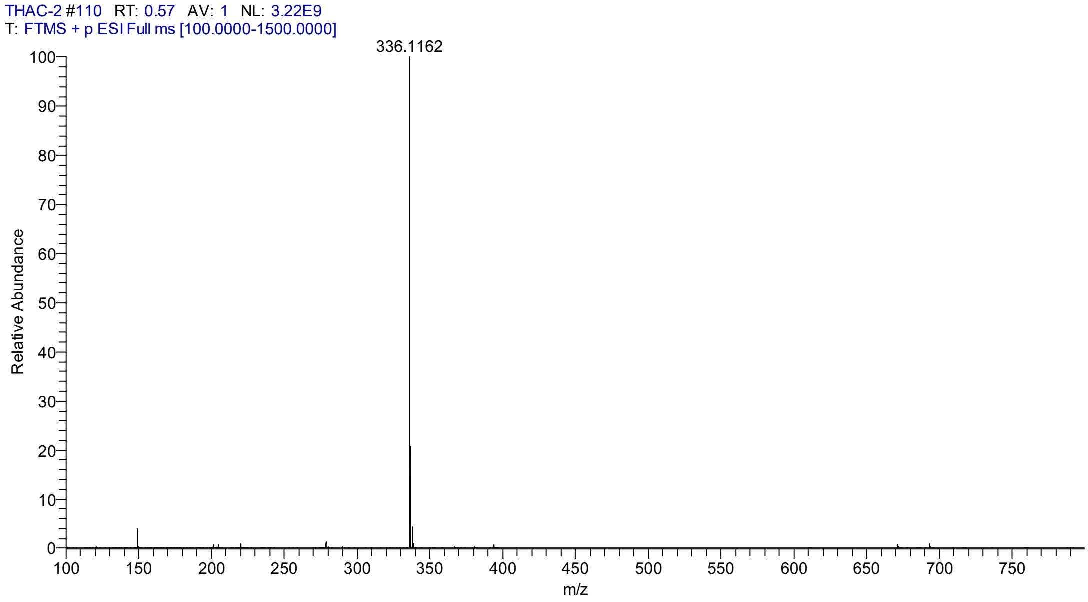

The HRMS spectrogram of compound **E4**

Compound **E5**, *N'*-(2-nitrobenzylidene)-2-(*o*-tolyl)thiazole-4-carbohydrazide:

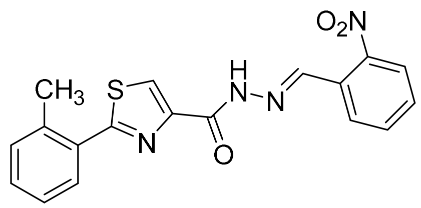

yellow solid, yield 64%, m.p. 124.7-126.5 °C; ^{1}H NMR (400 MHz, DMSO-d_6) δ 12.20 (s, 1H), 9.02 (s, 1H), 8.63 (s, 1H), 8.14 (d, J = 7.6 Hz, 1H), 8.07 (d, J = 8.0 Hz, 1H), 7.89 (d, J = 7.6 Hz, 1H), 7.83 (t, J = 7.6 Hz, 1H), 7.69 (t, J = 7.2 Hz, 1H), 7.48 – 7.35 (m, 3H), 2.59 (s, 3H). ^{13}C NMR (101 MHz, DMSO-d_6) δ 167.57, 157.87, 149.06, 148.88, 144.24, 136.70, 134.16, 132.36, 131.93, 131.24, 130.66, 130.38, 129.15, 128.53, 127.38, 126.86, 125.07, 21.55. HRMS(ESI) calcd for $C_{18}H_{14}N_4O_3S$ $[M+H]^+$: 367.0859, found 367.0858.

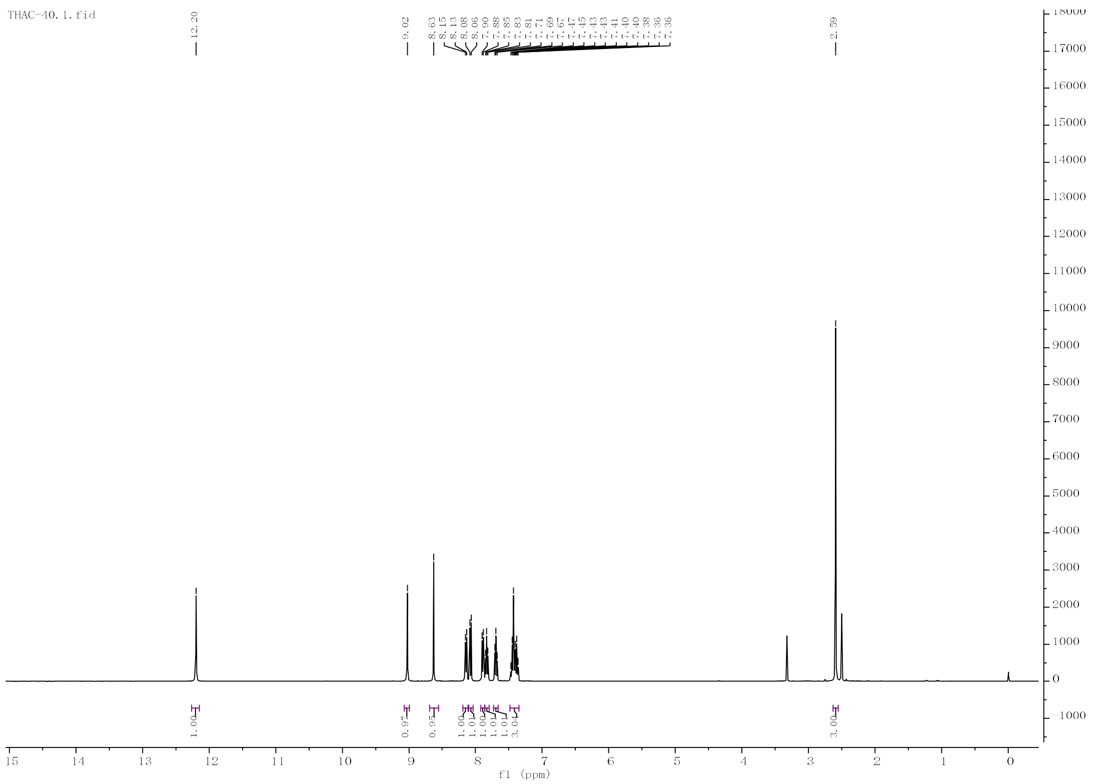

The ^{1}H NMR spectrogram of compound **E5**

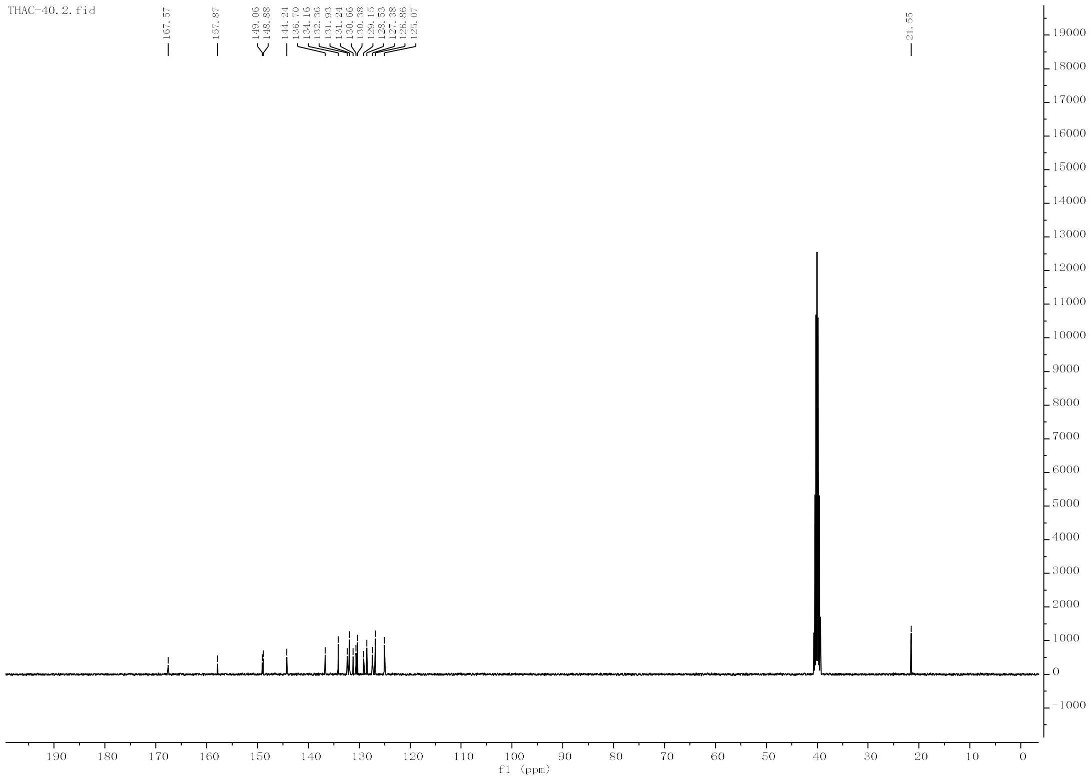

The ^{13}C NMR spectrogram of compound **E5**

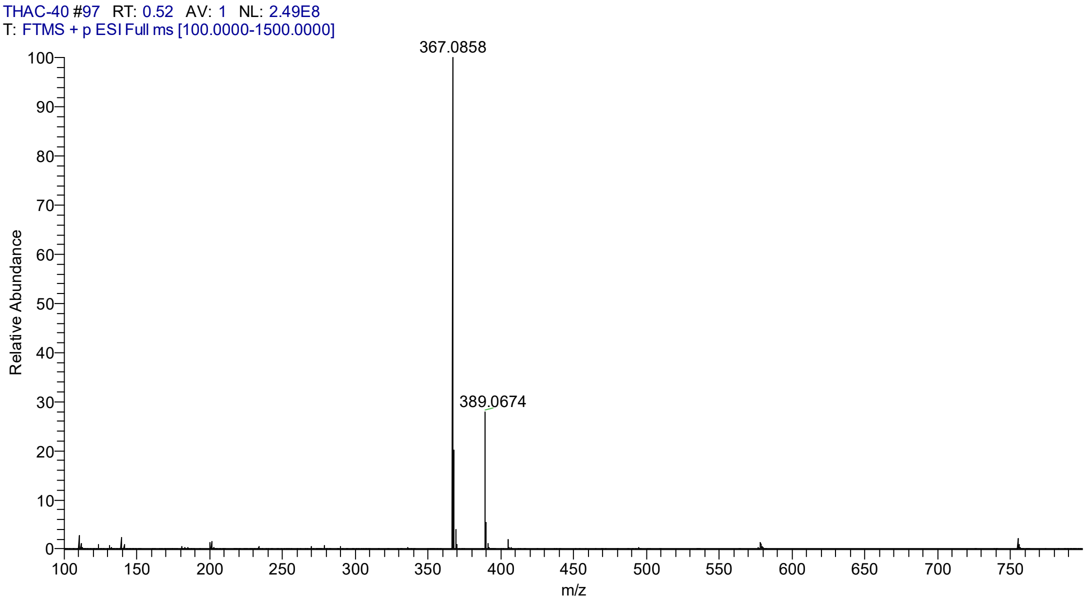

The HRMS spectrogram of compound **E5**

Compound **E6**, *N'*-(3-nitrobenzylidene)-2-(*o*-tolyl)thiazole-4-carbohydrazide:

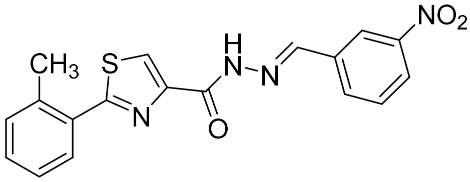

white solid, yield 68%, m.p. 176.4-177.5 °C; ^{1}H NMR (400 MHz, DMSO-d_6) δ 11.99 (s, 1H), 8.74 (s, 1H), 8.63 (s, 1H), 8.54 (s, 1H), 8.28 (dd, J = 8.0, 1.7 Hz, 1H), 8.14 (d, J = 7.6 Hz, 1H), 7.89 (d, J = 7.6 Hz, 1H), 7.77 (t, J = 8.0 Hz, 1H), 7.48 – 7.36 (m, 3H), 2.60 (s, 3H). ^{13}C NMR (101 MHz, DMSO-d_6) δ 167.66, 157.74, 149.07, 148.74, 146.99, 136.72, 136.64, 133.98, 132.32, 131.96, 131.03, 130.69, 130.38, 127.29, 126.87, 124.83, 121.38, 21.57. HRMS(ESI) calcd for $C_{18}H_{14}N_4O_3S$ $[M+H]^+$: 367.0859, found 367.0858.

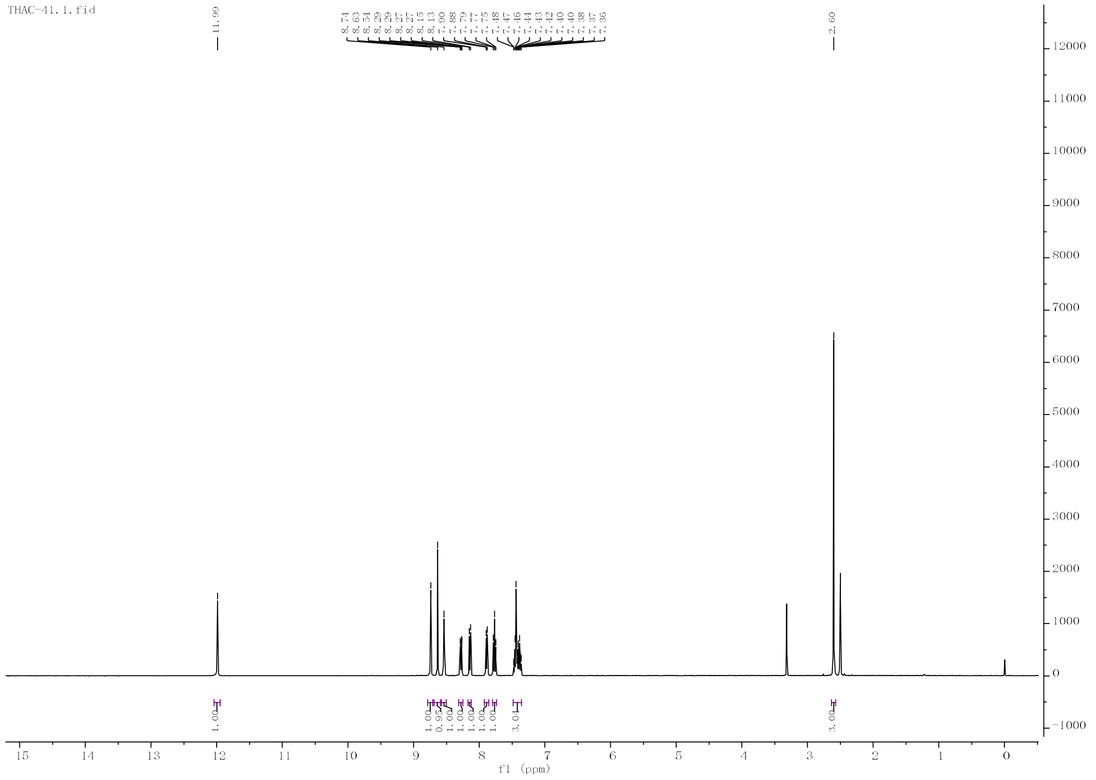

The ^{1}H NMR spectrogram of compound **E6**

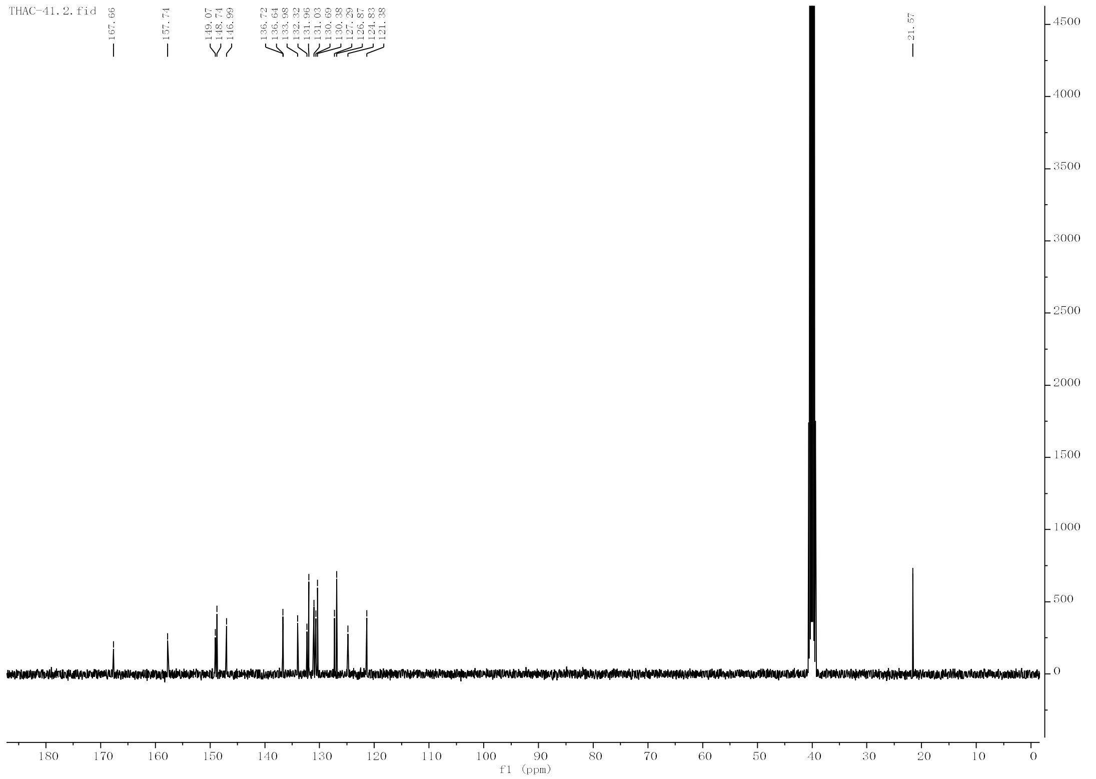

The ^{13}C NMR spectrogram of compound **E6**

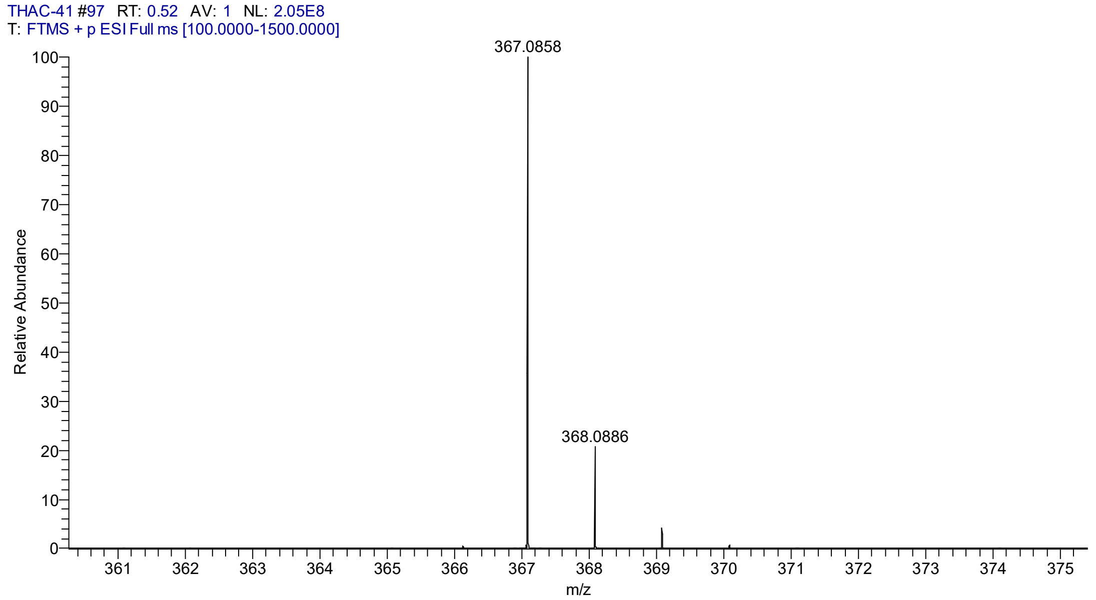

The HRMS spectrogram of compound **E6**

Compound **E7**, *N'*-(4-nitrobenzylidene)-2-(*o*-tolyl)thiazole-4-carbohydrazide:

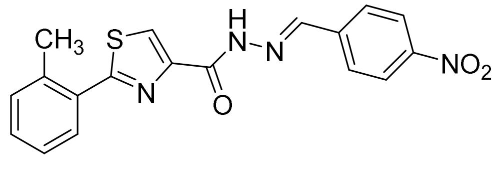

yellow solid, yield 66%, m.p. 177.1-179.4°C; ^{1}H NMR (400 MHz, $CDCl_3$) δ 10.57 (s, 1H), 8.47 (s, 1H), 8.36 (s, 1H), 8.26 (d, J = 8.8 Hz, 2H), 7.96 (d, J = 8.8 Hz, 2H), 7.72 (d, J = 7.6 Hz, 1H), 7.36 (td, J = 16.0, 14.4, 7.6 Hz, 3H), 2.62 (s, 3H). ^{13}C NMR (101 MHz, $CDCl_3$) δ 168.51, 157.42, 148.73, 148.36, 145.74, 139.87, 136.56, 131.81, 131.72, 130.36, 130.00, 128.25, 126.42, 125.80, 123.99, 21.47. HRMS (ESI): calcd for $C_{18}H_{14}N_4O_3S$ [M+H]$^+$: 367.0859; found, 367.0858.

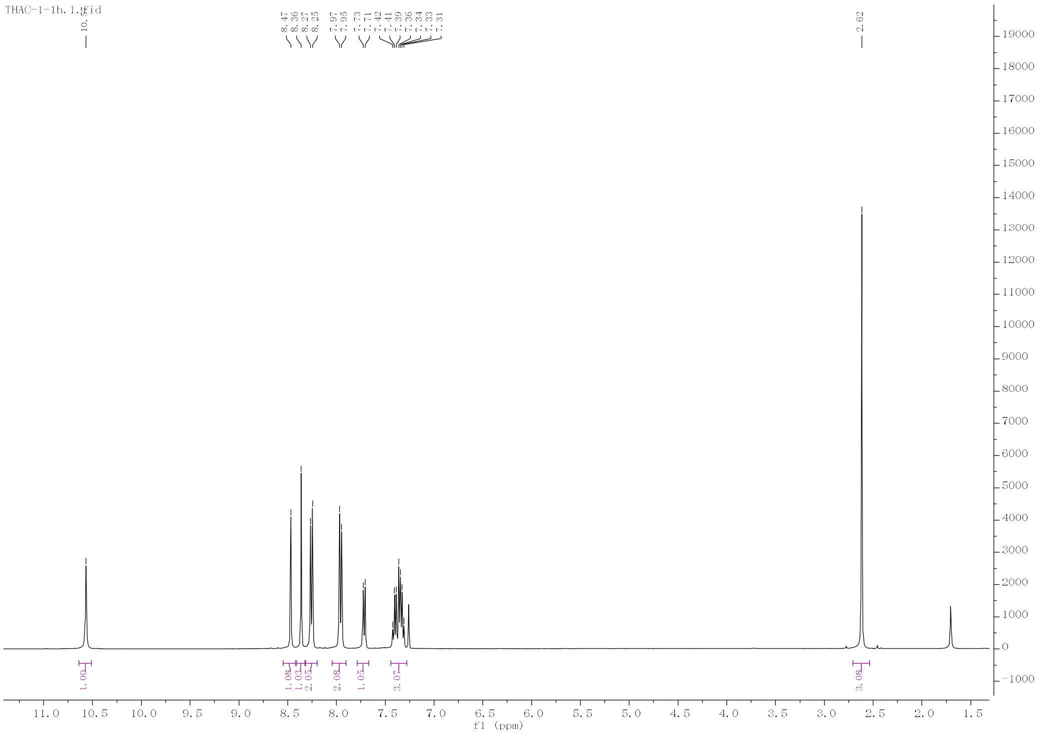

The ^{1}H NMR spectrogram of compound **E7**

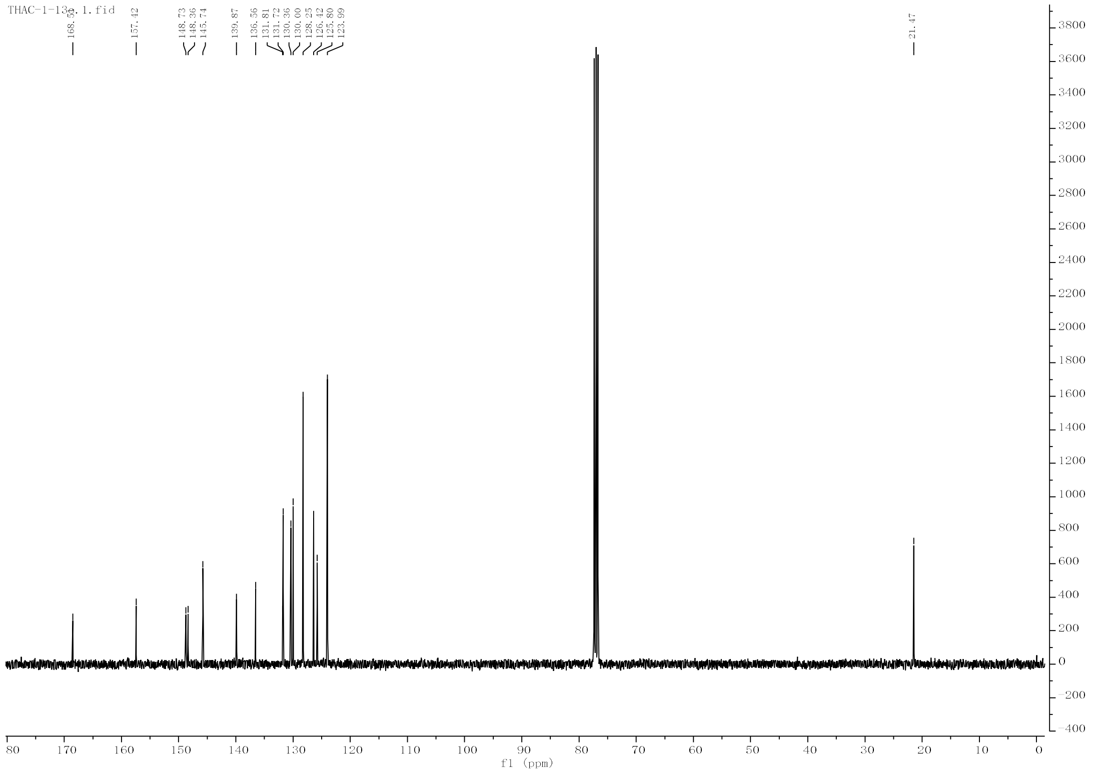

The ^{13}C NMR spectrogram of compound **E7**

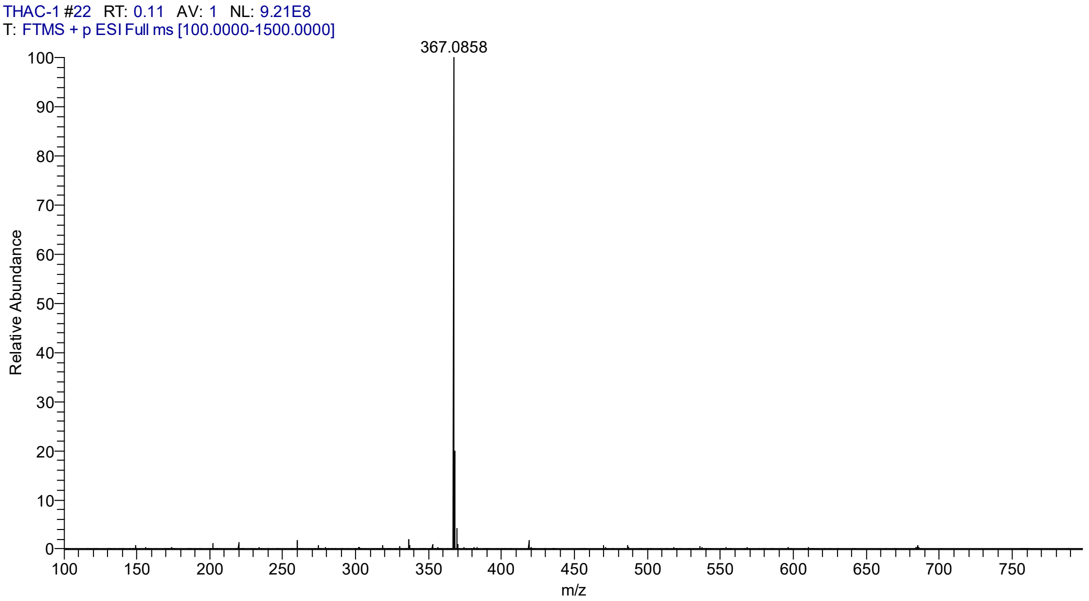

The HRMS spectrogram of compound **E7**

Compound **E8**, *N′*-(2-chlorobenzylidene)-2-(*o*-tolyl)thiazole-4-carbohydrazide:

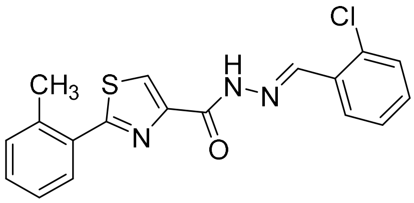

white solid, yield 69%, m.p. 159.0-161.7 °C; ^{1}H NMR (400 MHz, DMSO-d_6) δ 12.13 (s, 1H), 9.05 (s, 1H), 8.62 (s, 1H), 8.12 – 7.98 (m, 1H), 7.89 (d, J = 7.6 Hz, 1H), 7.54 – 7.34 (m, 6H), 2.59 (s, 3H). ^{13}C NMR (101 MHz, DMSO-d_6) δ 167.51, 157.76, 149.20, 145.21, 136.70, 133.83, 132.38, 132.22, 132.00, 131.92, 130.63, 130.40, 130.37, 128.06, 127.49, 127.16, 126.84, 21.57. HRMS(ESI) calcd for $C_{18}H_{14}ClN_3OS$ $[M+H]^+$: 356.0619, found 356.0618.

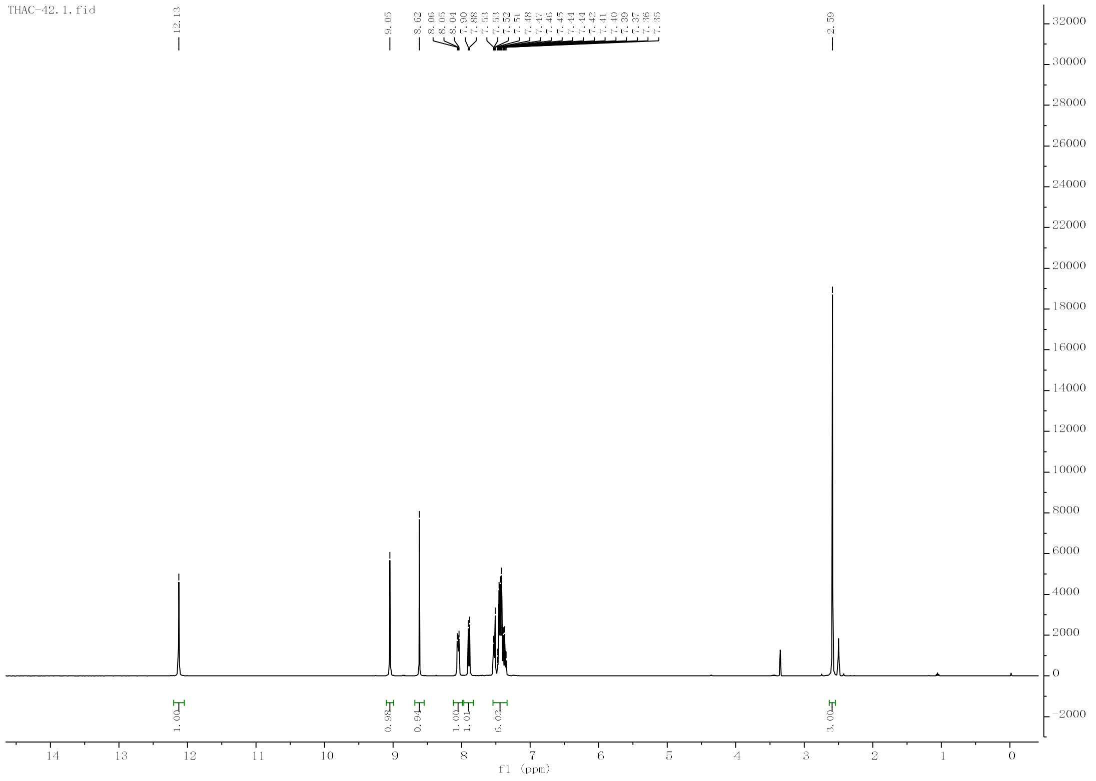

The ^{1}H NMR spectrogram of compound **E8**

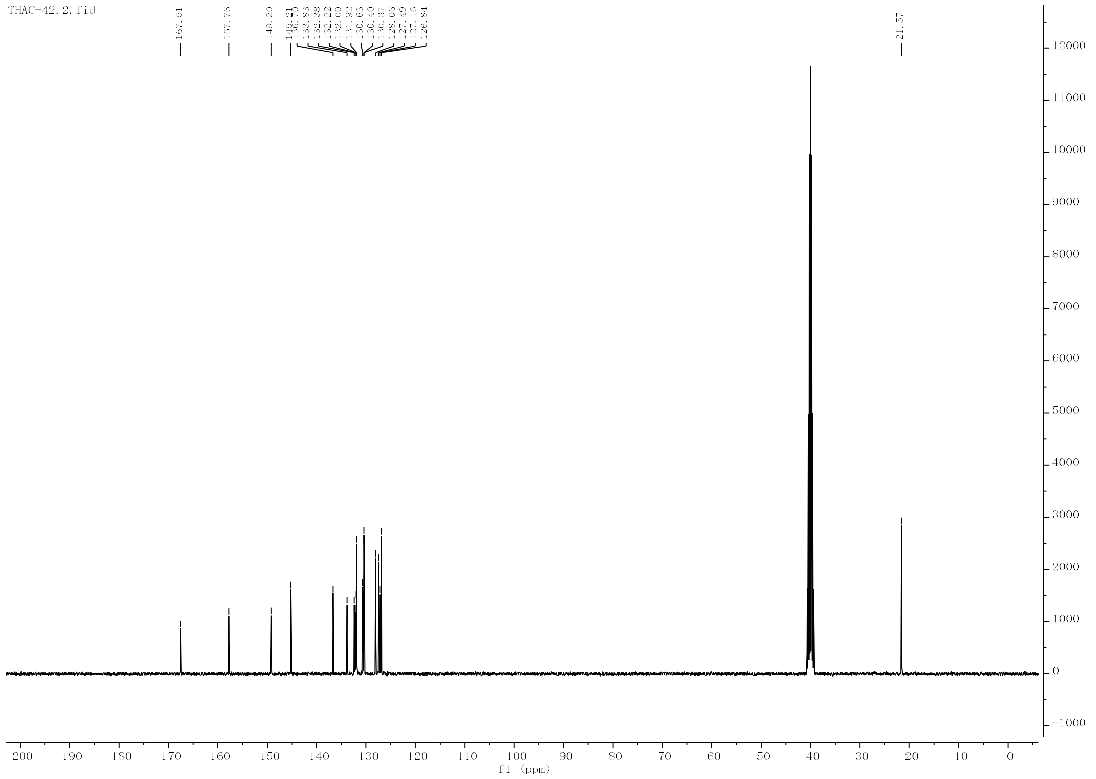

The ^{13}C NMR spectrogram of compound **E8**

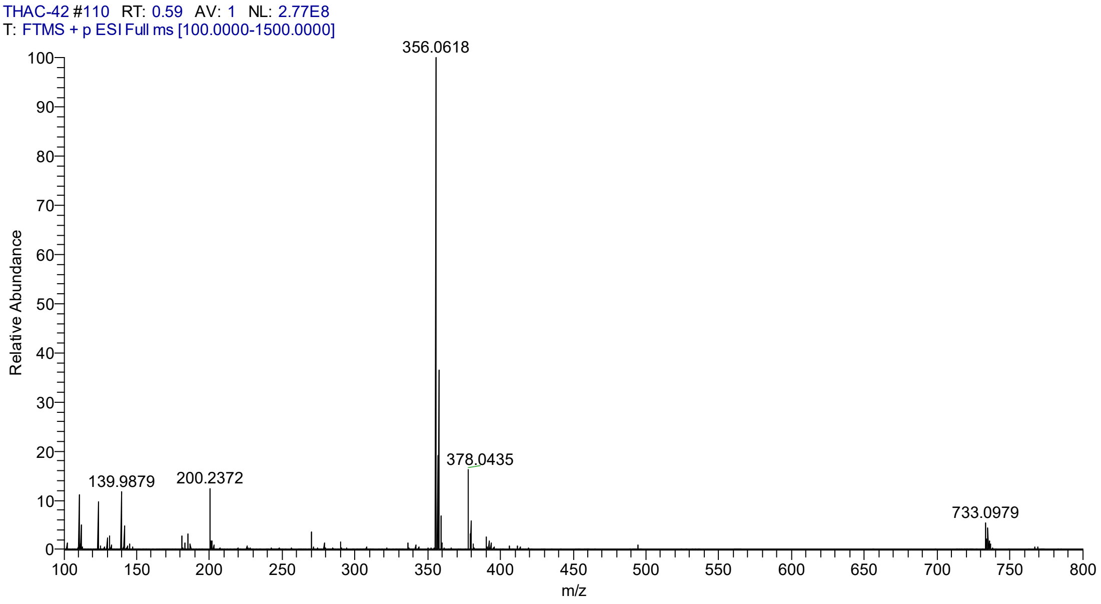

The HRMS spectrogram of compound **E8**

Compound **E9**, *N'*-(3-chlorobenzylidene)-2-(*o*-tolyl)thiazole-4-carbohydrazide:

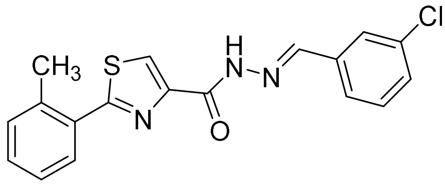

white solid, yield 68%, m.p. 141.7-143.4 °C; ^{1}H NMR (400 MHz, DMSO-d_6) δ 11.90 (s, 1H), 8.61 (s, 2H), 7.93 – 7.61 (m, 3H), 7.55 – 7.33 (m, 5H), 2.59 (s, 3H). ^{13}C NMR (101 MHz, DMSO-d_6) δ 167.58, 157.64, 149.17, 147.68, 137.03, 136.71, 134.15, 132.32, 131.96, 131.28, 130.66, 130.37, 130.28, 127.09, 126.86, 126.72, 126.45, 21.60. HRMS(ESI) calcd for $C_{18}H_{14}ClN_3OS$ [M+H]$^+$: 356.0619, found 356.0617.

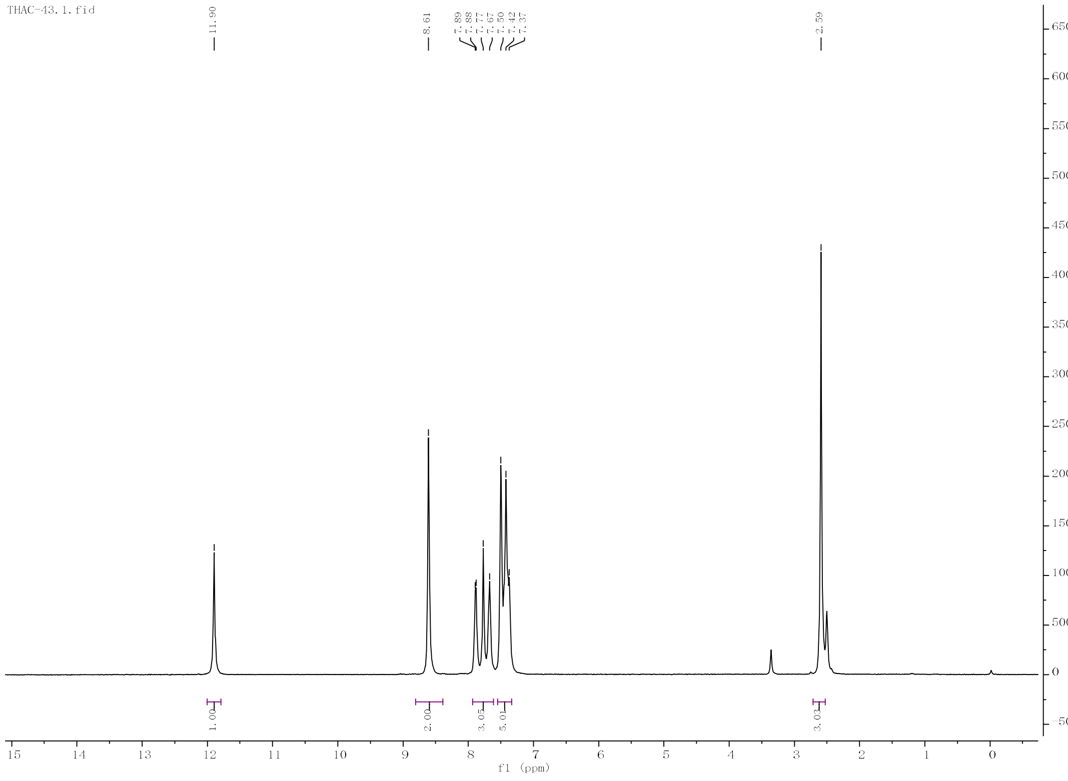

The ^{1}H NMR spectrogram of compound **E9**

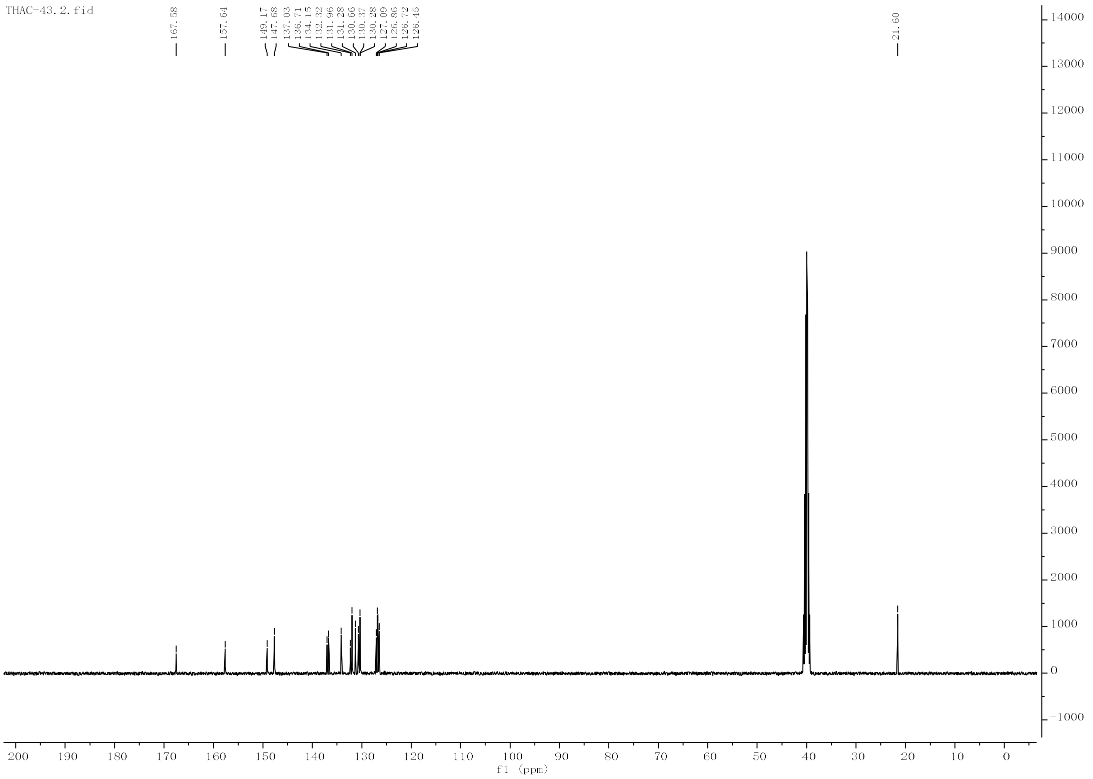

The ^{13}C NMR spectrogram of compound **E9**

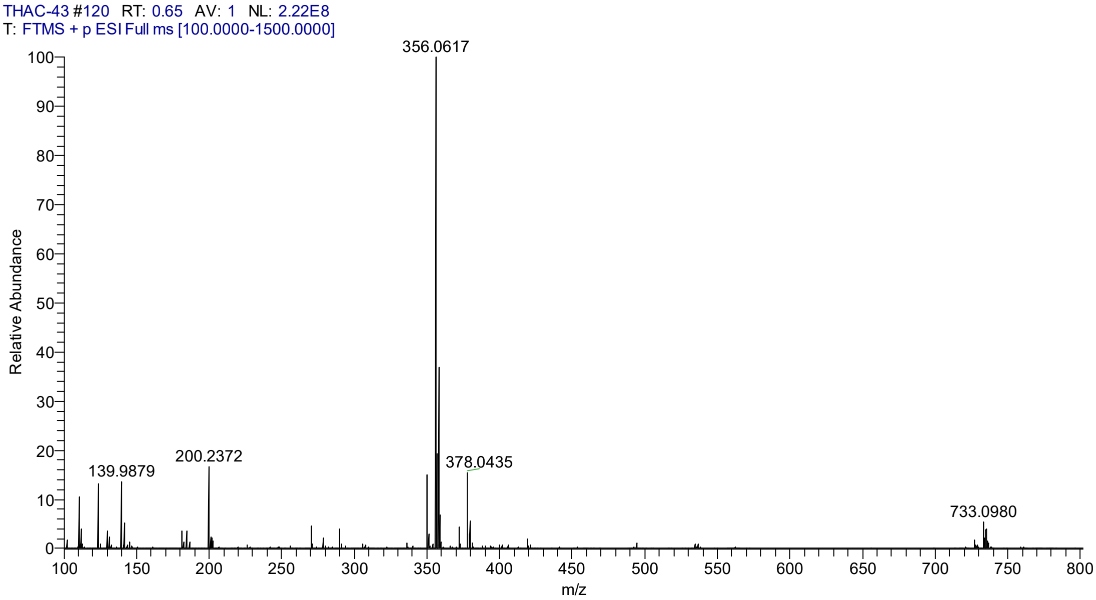

The HRMS spectrogram of compound **E9**

Compound **E10**, *N'*-(4-chlorobenzylidene)-2-(*o*-tolyl)thiazole-4-carbohydrazide:

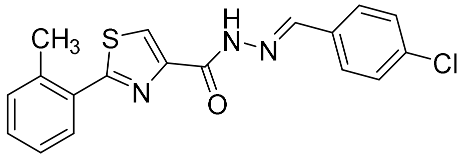

white solid, yield 68%, m.p. 137.3-139.4 °C; ^{1}H NMR (400 MHz, DMSO-d_6) δ 11.80 (s, 1H), 8.60 (d, J = 7.2 Hz, 2H), 7.89 (d, J = 7.6 Hz, 1H), 7.75 (d, J = 8.8 Hz, 2H), 7.53 (d, J = 8.8 Hz, 2H), 7.40 (dt, J = 20.0, 7.5 Hz, 3H), 2.59 (s, 3H). ^{13}C NMR (101 MHz, DMSO-d_6) δ 167.57, 157.56, 149.25, 148.05, 136.71, 135.08, 133.75, 132.34, 131.95, 130.65, 130.36, 129.45, 129.27, 126.94, 126.86, 21.59. HRMS(ESI) calcd for $C_{18}H_{14}ClN_3OS$ $[M+H]^+$: 356.0619, found 356.0618.

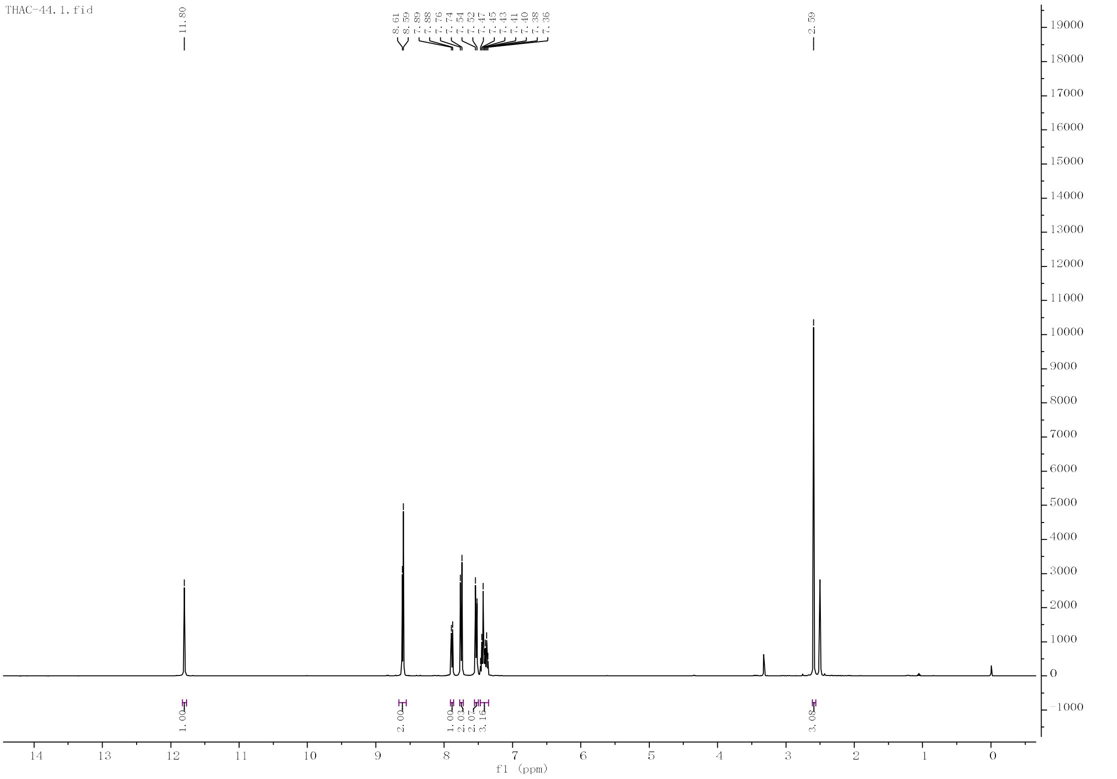

The ^{1}H NMR spectrogram of compound **E10**

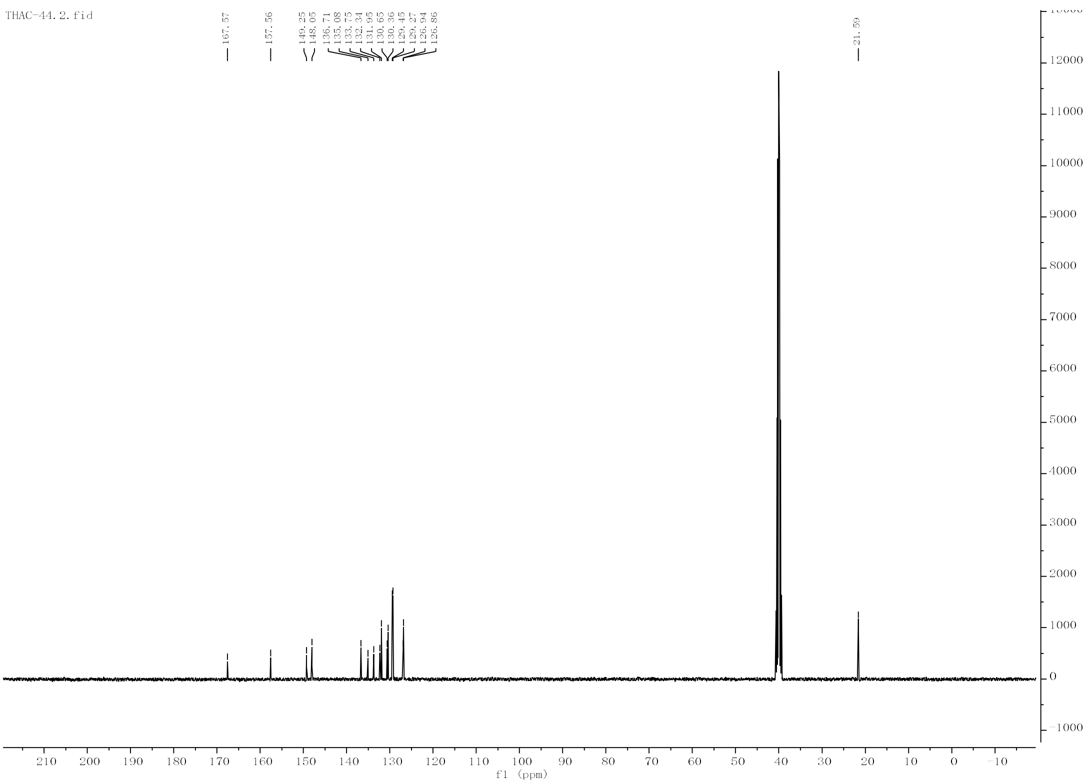

The ^{13}C NMR spectrogram of compound **E10**

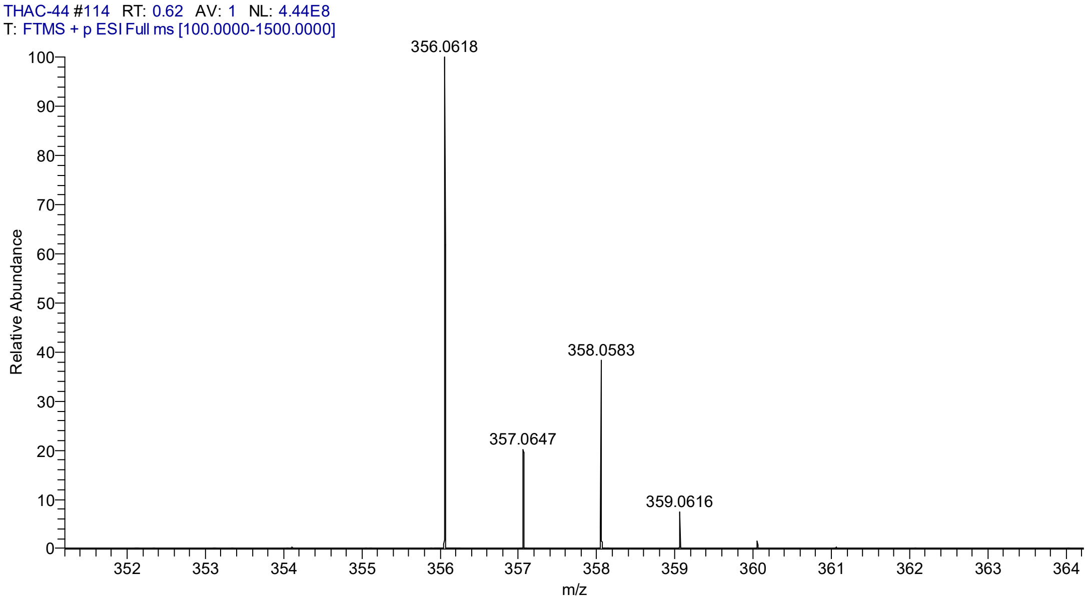

The HRMS spectrogram of compound **E10**

Compound **E11**, *N'*-(4-methoxybenzylidene)-2-(*o*-tolyl)thiazole-4-carbohydrazide:

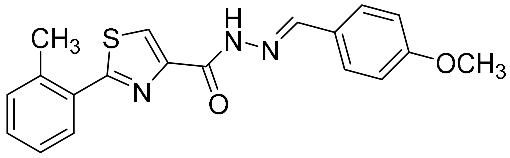

white solid, yield 72 %, m.p. 147.8-149.5°C; ^{1}H NMR (400 MHz, DMSO-d_6) δ 11.57 (s, 1H), 8.55 (s, 2H), 7.89 (d, J = 7.6 Hz, 1H), 7.68 (d, J = 8.8 Hz, 2H), 7.48 – 7.34 (m, 3H), 7.03 (d, J = 8.8 Hz, 2H), 3.81 (s, 3H), 2.60 (s, 3H). ^{13}C NMR (101 MHz, DMSO-d_6) δ 167.49, 161.42, 157.34, 149.53, 149.29, 136.70, 132.37, 131.95, 130.61, 130.36, 129.27, 127.36, 126.85, 126.44, 114.86, 55.80, 21.61. HRMS (ESI): calcd for $C_{19}H_{17}N_3O_2S$ [M+H]$^+$, 352.1114; found, 352.1114.

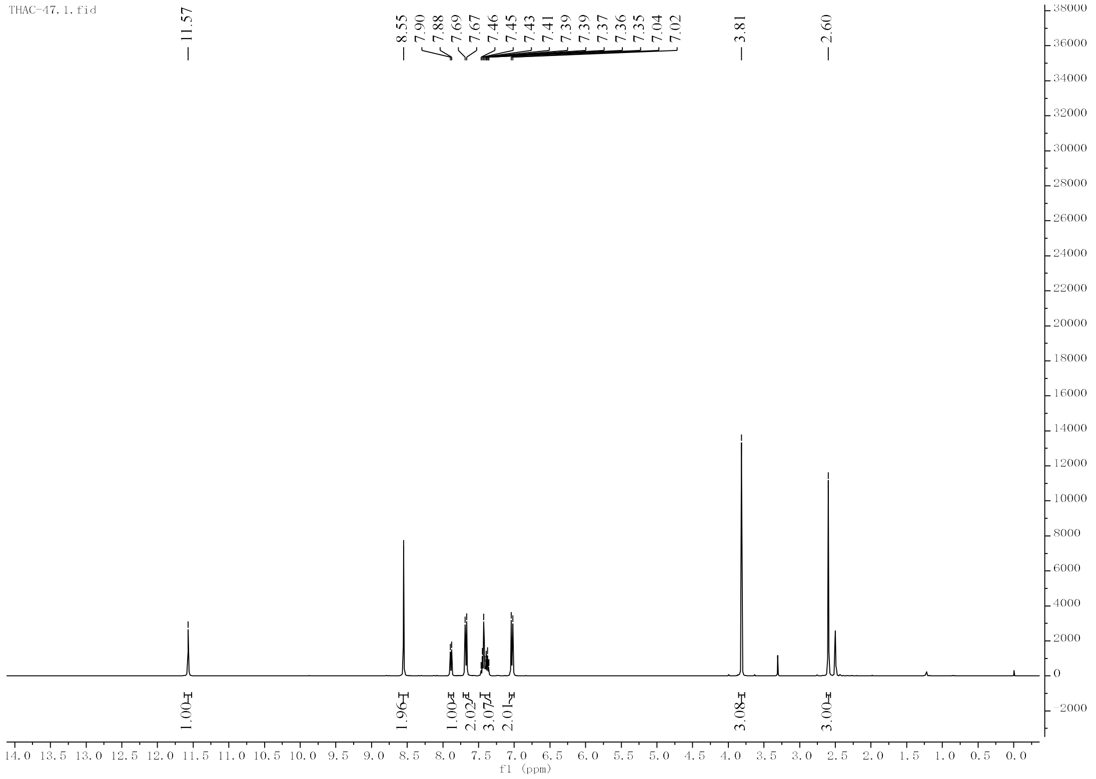

The ^{1}H NMR spectrogram of compound **E11**

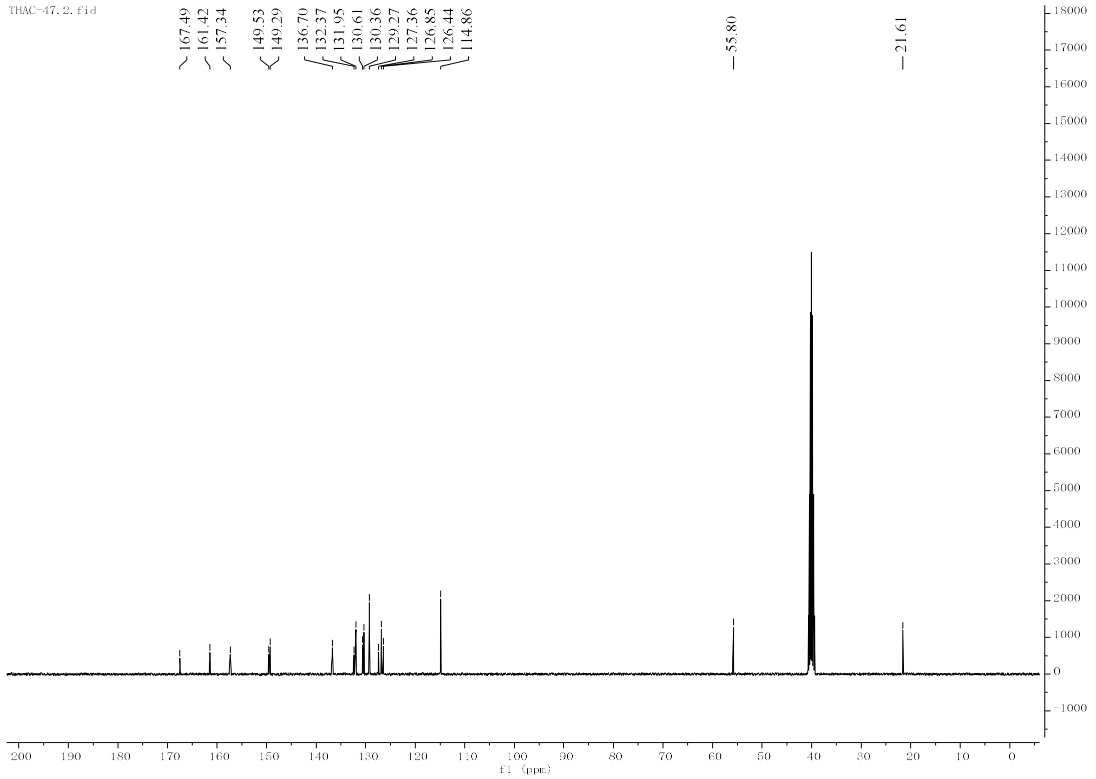

The ^{13}C NMR spectrogram of compound **E11**

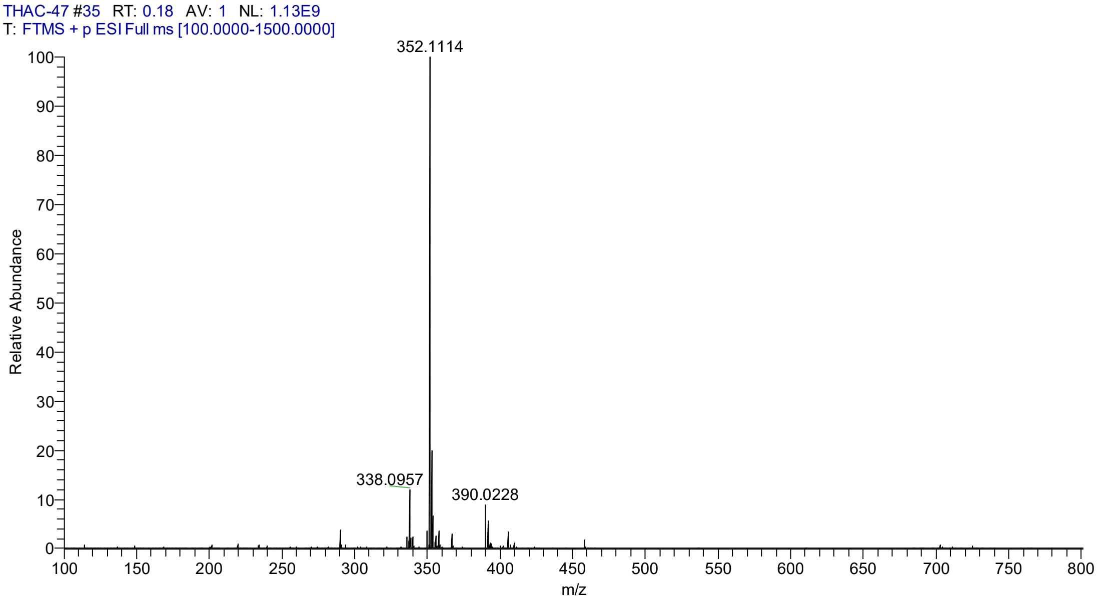

The HRMS spectrogram of compound **E11**

Compound **E12**, *N'*-(3,4-dimethylbenzylidene)-2-(*o*-tolyl)thiazole-4-carbohydrazide:

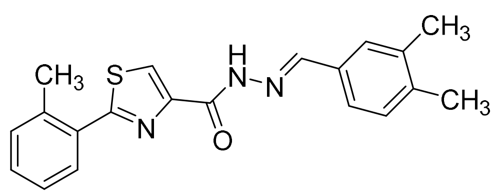

white solid, yield 74%, m.p. 169.8-170.5 °C; ^{1}H NMR (400 MHz, DMSO-d_6) δ 11.65 (s, 1H), 8.55 (d, J = 13.6 Hz, 2H), 7.88 (d, J = 7.6 Hz, 1H), 7.52 (s, 1H), 7.47 – 7.35 (m, 4H), 7.23 (d, J = 8.0 Hz, 1H), 2.59 (s, 3H), 2.27 (d, J = 6.0 Hz, 6H). ^{13}C NMR (101 MHz, DMSO-d_6) δ 167.50, 157.43, 149.56, 149.45, 139.35, 137.27, 136.71, 132.41, 132.36, 131.95, 130.63, 130.42, 130.36, 128.33, 126.85, 126.62, 125.53, 21.61, 19.90, 19.79. HRMS(ESI) calcd for $C_{20}H_{19}N_{3}OS$ [M+H]$^{+}$: 350.1322, found 350.1321.

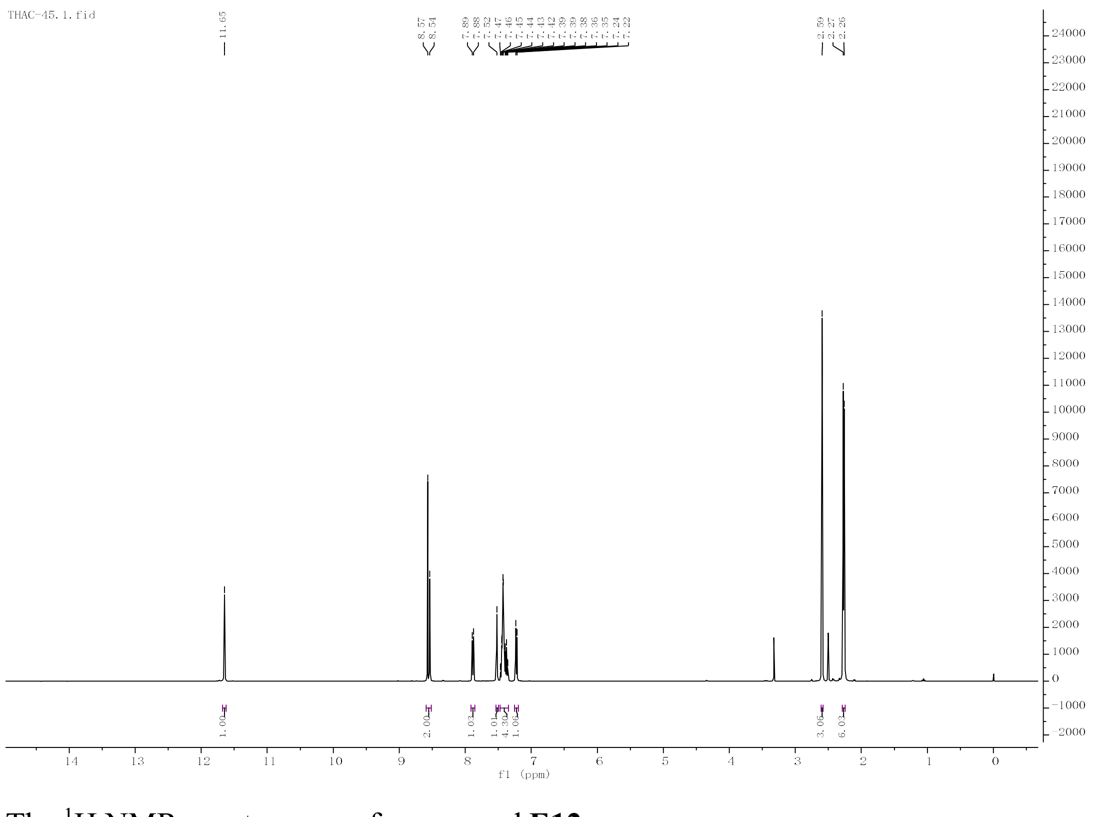

The ^{1}H NMR spectrogram of compound **E12**

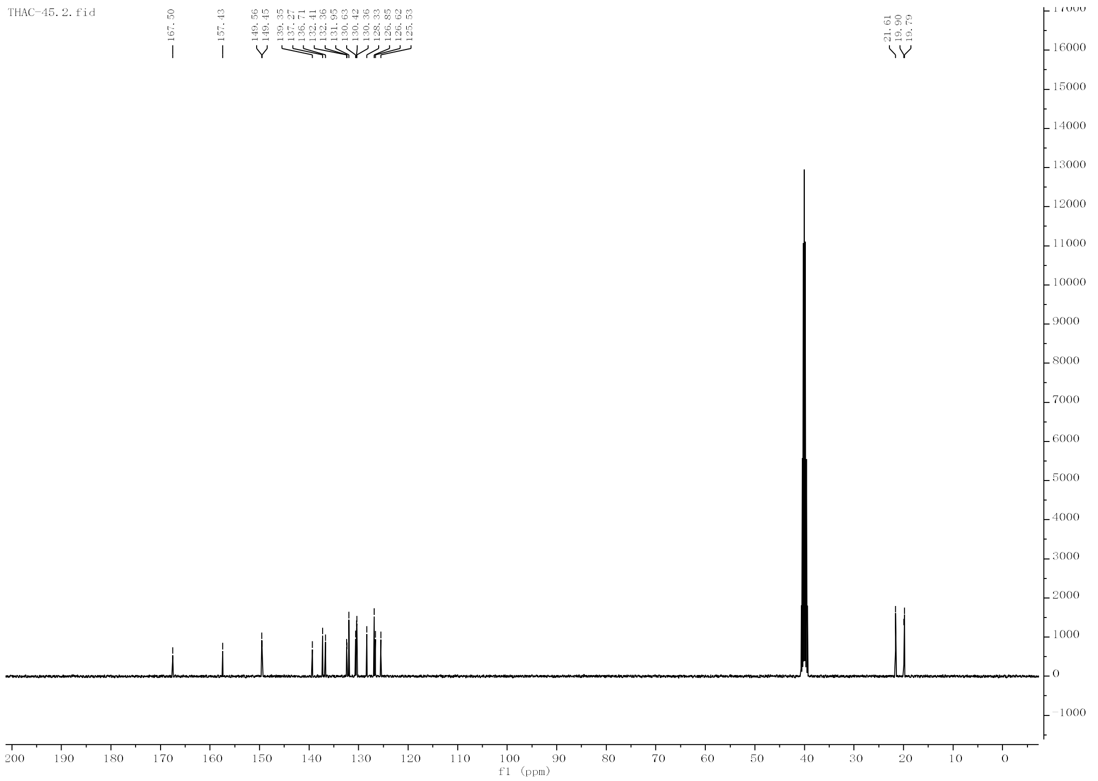

The ^{13}C NMR spectrogram of compound **E12**

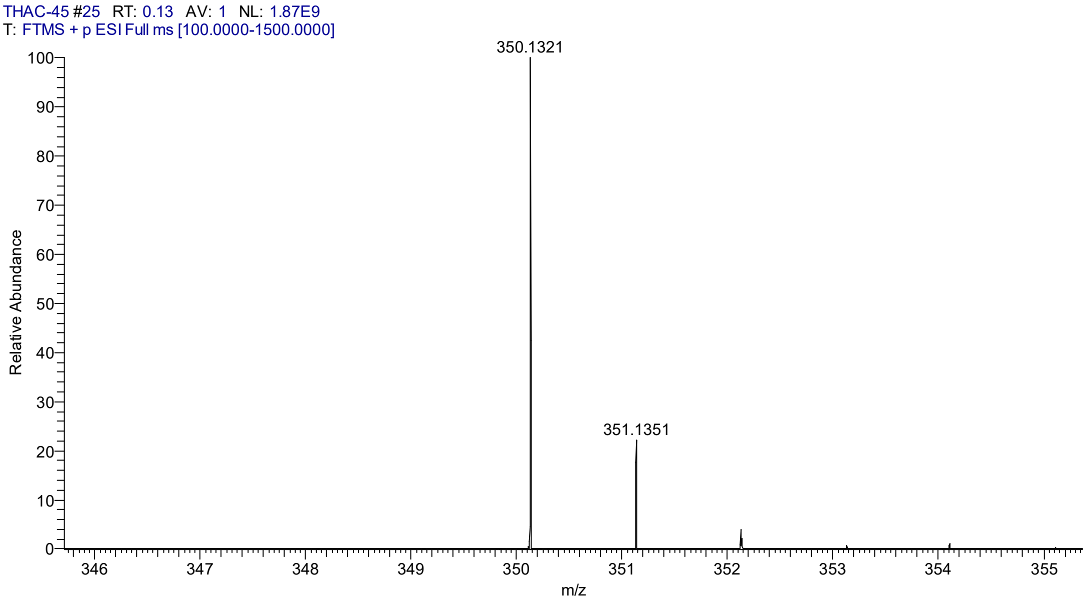

The HRMS spectrogram of compound **E12**

Compound **E13**, *N'*-(2,4-dichlorobenzylidene)-2-(*o*-tolyl)thiazole-4-carbohydrazide:

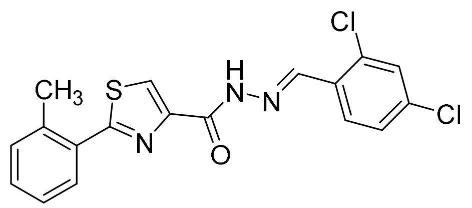

white solid, yield 67%, m.p. 162.2-163.2 °C; ^{1}H NMR (400 MHz, DMSO-d_6) δ 12.16 (s, 1H), 9.00 (s, 1H), 8.63 (s, 1H), 8.05 (d, J = 8.8 Hz, 1H), 7.90 (d, J = 7.6 Hz, 1H), 7.72 (d, J = 2.0 Hz, 1H), 7.57 – 7.36 (m, 4H), 2.60 (s, 3H). ^{13}C NMR (101 MHz, DMSO-d_6) δ 167.55, 157.75, 149.09, 144.13, 136.71, 135.61, 134.49, 132.38, 131.92, 131.35, 130.66, 130.37, 129.87, 128.68, 128.49, 127.31, 126.85, 21.55. HRMS(ESI) calcd for $C_{18}H_{13}Cl_2N_3OS$ [M+H]$^+$: 390.0229, found 390.0230.

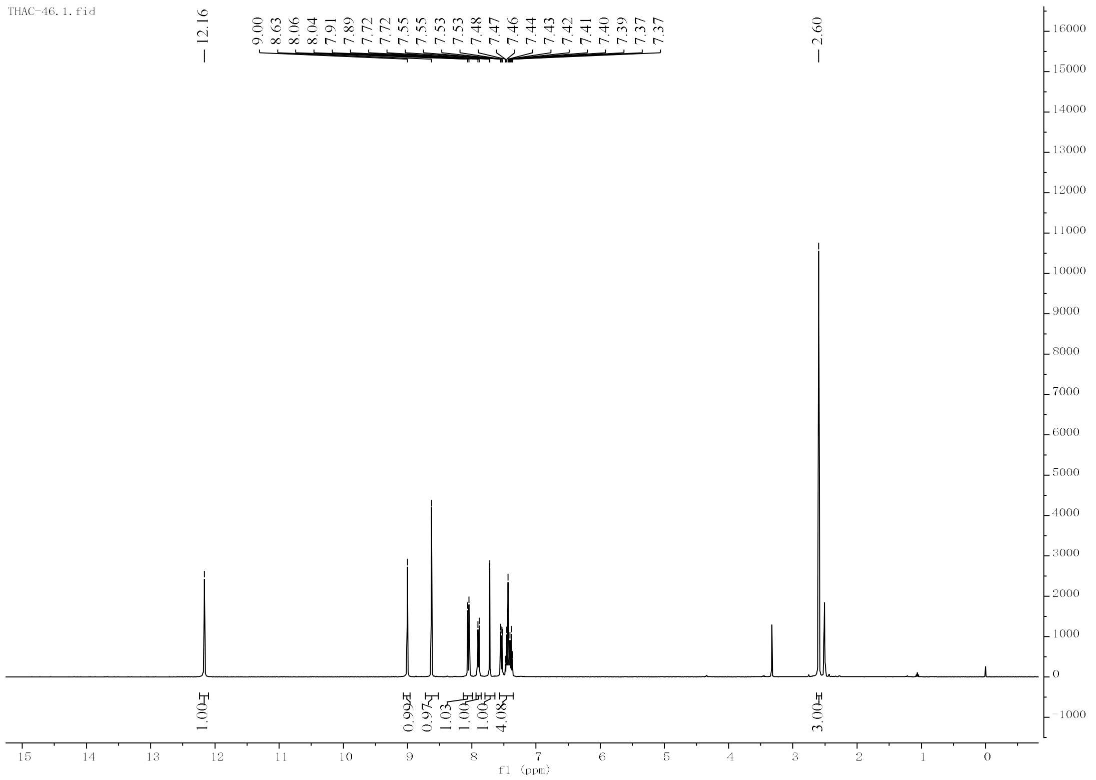

The ^{1}H NMR spectrogram of compound **E13**

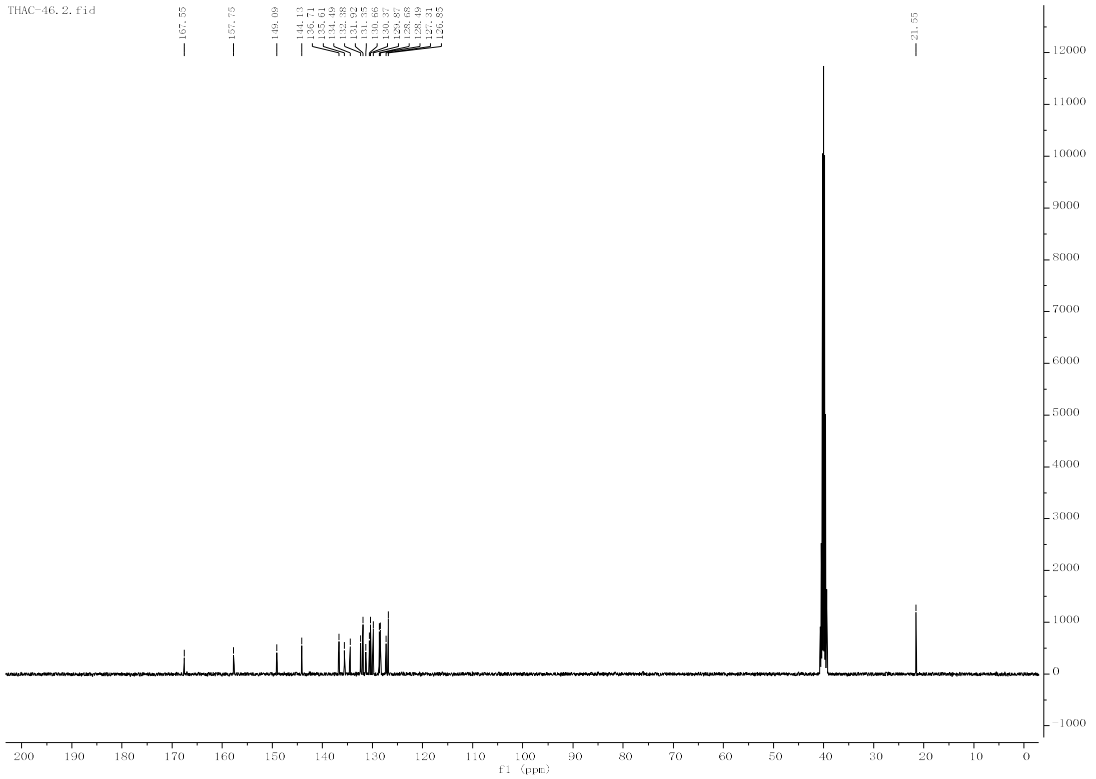

The ^{13}C NMR spectrogram of compound **E13**

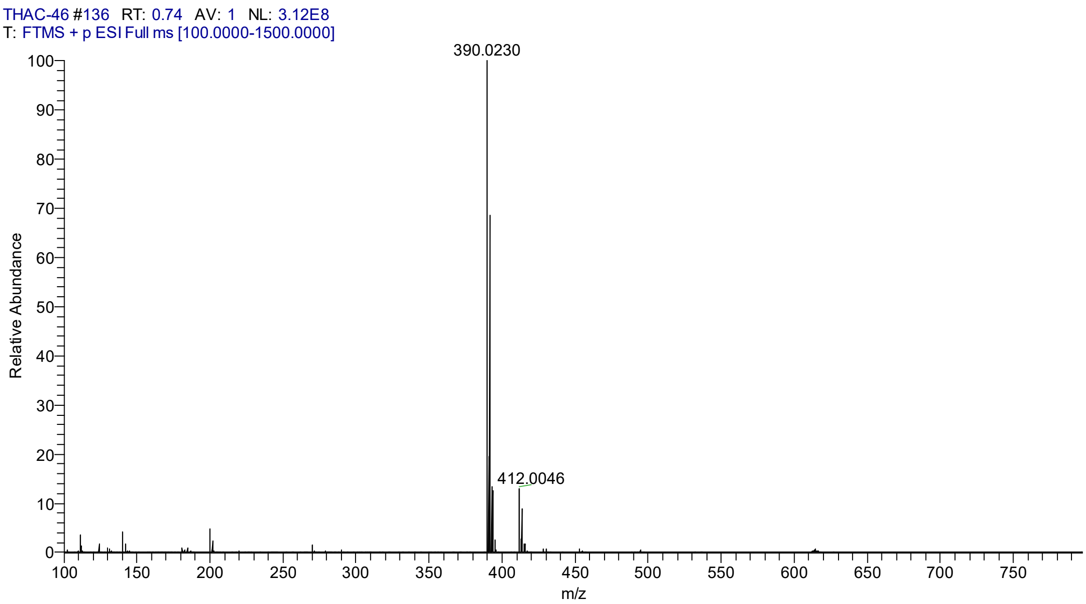

The HRMS spectrogram of compound **E13**

Compound **E14**, *N′*-(4-methylbenzylidene)-2-phenylthiazole-4-carbohydrazide:

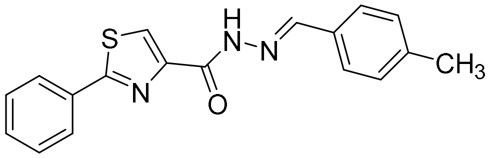

white solid, yield 64%, m.p. 177.4-179.1 °C; ^{1}H NMR (400 MHz, $CDCl_3$) δ 10.38 (s, 1H), 8.28 (d, J = 17.2 Hz, 2H), 7.98 (dd, J = 6.4, 3.0 Hz, 2H), 7.72 (d, J = 7.6 Hz, 2H), 7.55 – 7.44 (m, 3H), 7.22 (d, J = 7.6 Hz, 2H), 2.39 (s, 3H). ^{13}C NMR (101 MHz, $CDCl_3$) δ 168.56, 157.04, 149.48, 148.83, 141.01, 132.66, 130.91, 130.85, 129.46, 129.15, 127.87, 126.74, 124.44, 21.55. HRMS(ESI) calcd for $C_{18}H_{15}N_3OS$ $[M+H]^+$: 322.1009, found 322.1008.

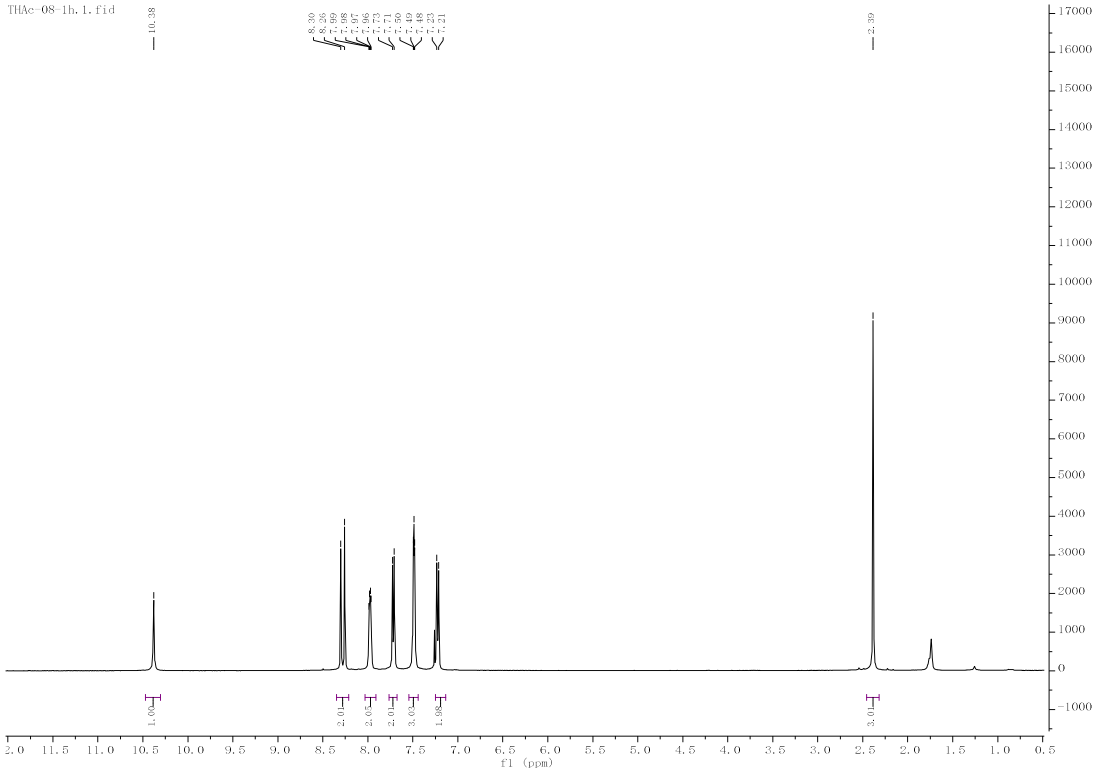

The ^{1}H NMR spectrogram of compound **E14**

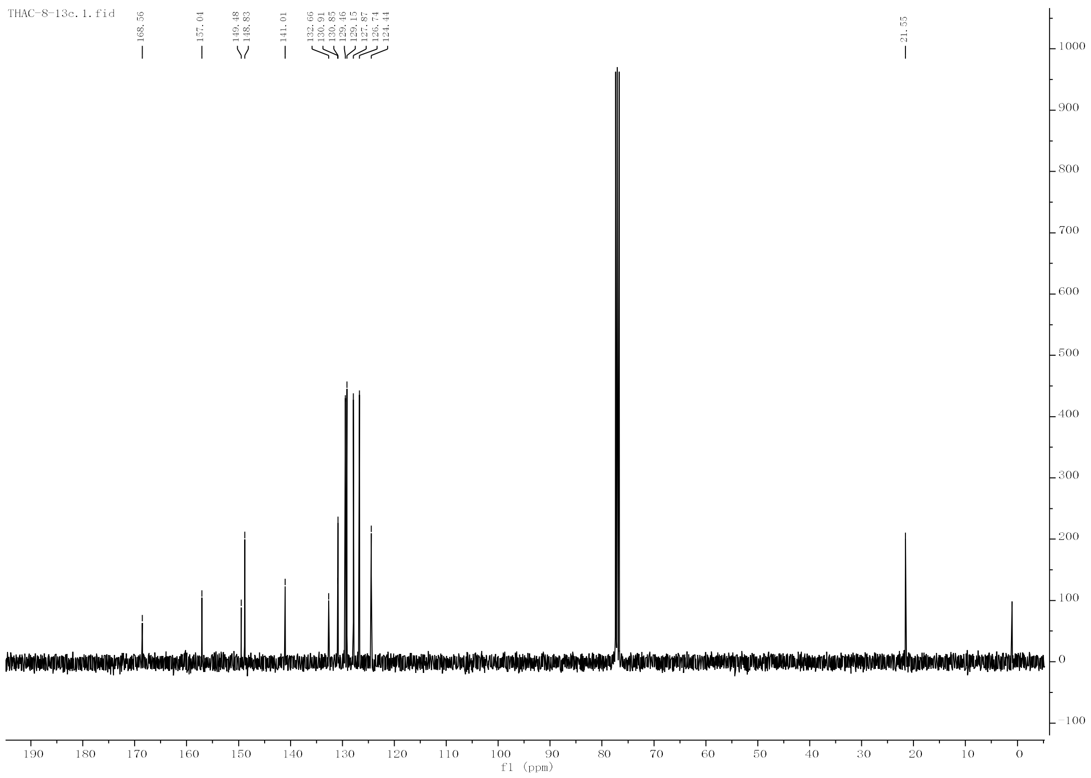

The ^{13}C NMR spectrogram of compound **E14**

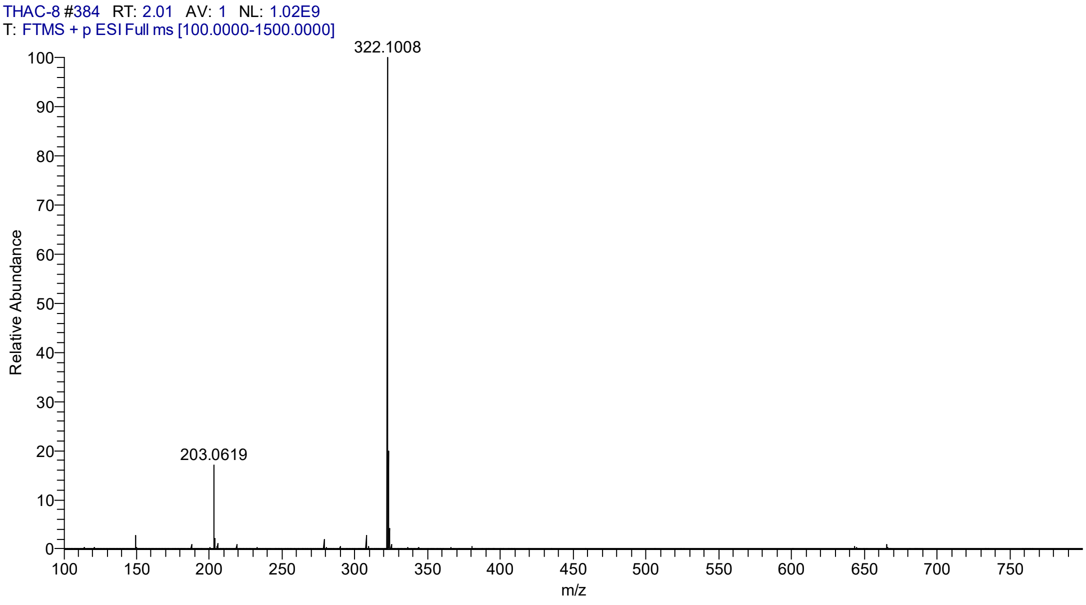

The HRMS spectrogram of compound **E14**

Compound **E15**, *N*′-(4-methylbenzylidene)-2-(*m*-tolyl)thiazole-4-carbohydrazide:

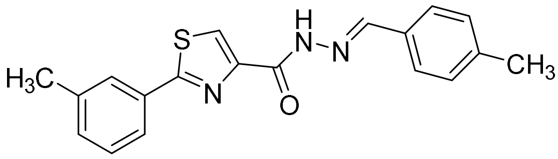

white solid, yield 62%, m.p. 152.8-154.1 °C; ^{1}H NMR (400 MHz, DMSO-*d$_6$*) δ 11.65 (s, 1H), 8.62 (s, 1H), 8.46 (s, 1H), 7.98 – 7.87 (m, 2H), 7.64 (d, *J* = 8.0 Hz, 2H), 7.44 (t, *J* = 7.6 Hz, 1H), 7.36 (d, *J* = 7.6 Hz, 1H), 7.29 (d, *J* = 8.0 Hz, 2H), 2.43 (s, 3H), 2.36 (s, 3H). ^{13}C NMR (101 MHz, DMSO-*d$_6$*) δ 168.17, 157.33, 149.98, 149.43, 140.50, 139.16, 132.89, 132.13, 131.99, 129.94, 129.59, 127.64, 127.46, 125.77, 124.40, 21.50, 21.36. HRMS(ESI) calcd for $C_{19}H_{17}N_3OS$ [M+H]$^+$: 336.1165, found 336.1165.

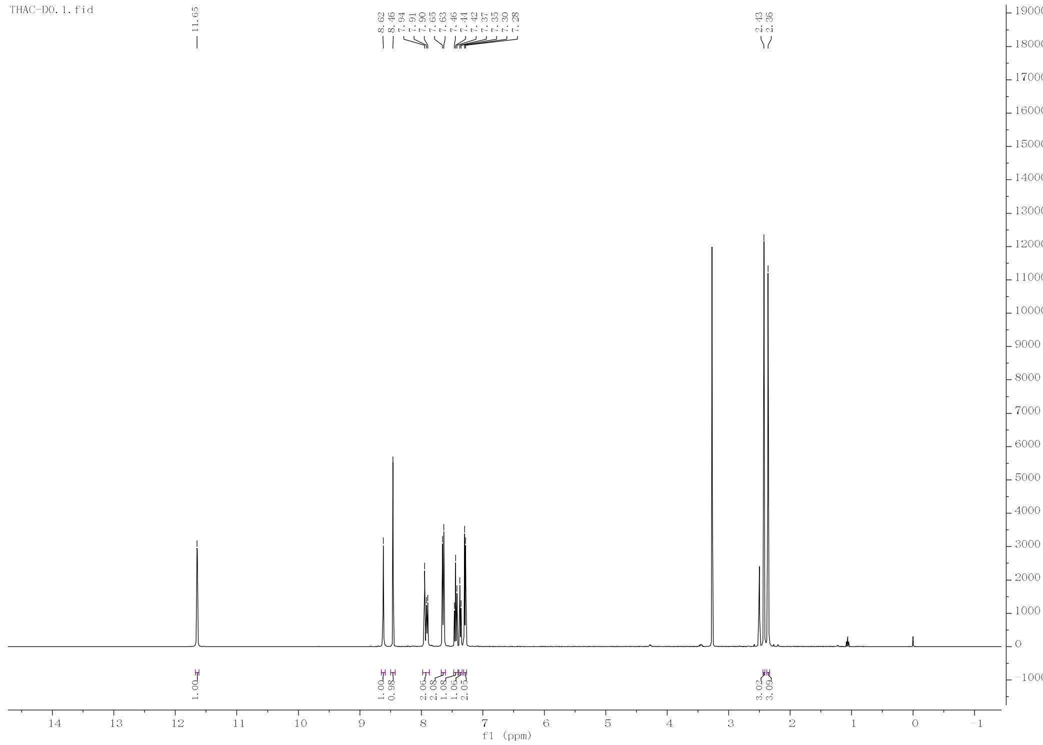

The ^{1}H NMR spectrogram of compound **E15**

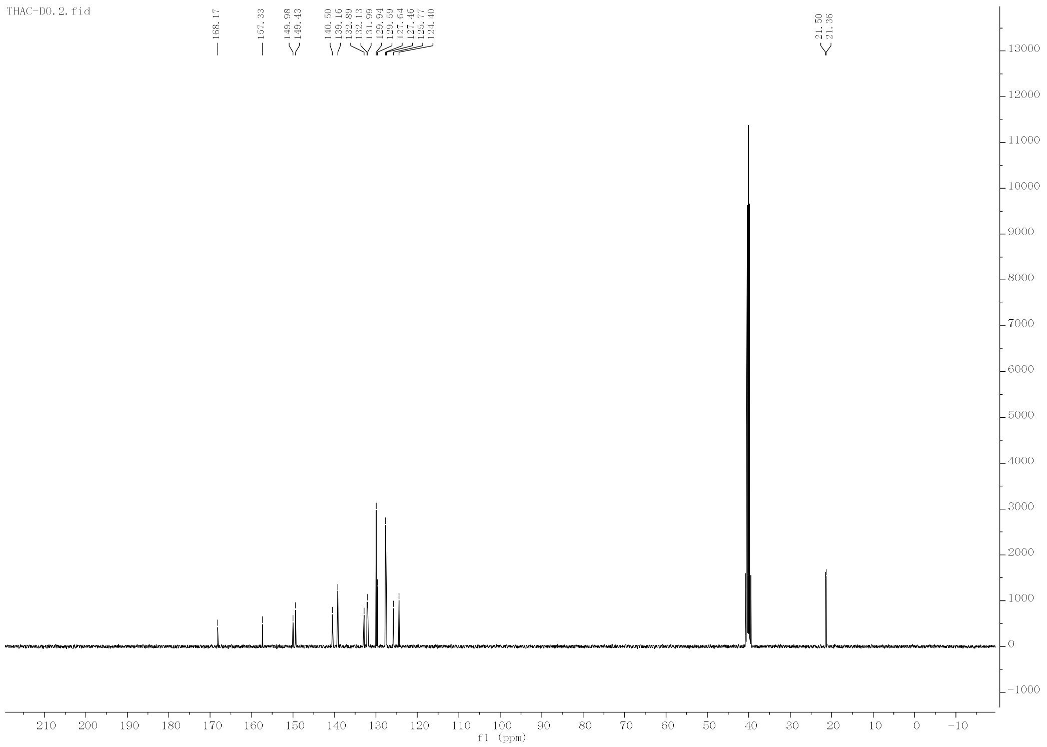

The ^{13}C NMR spectrogram of compound **E15**

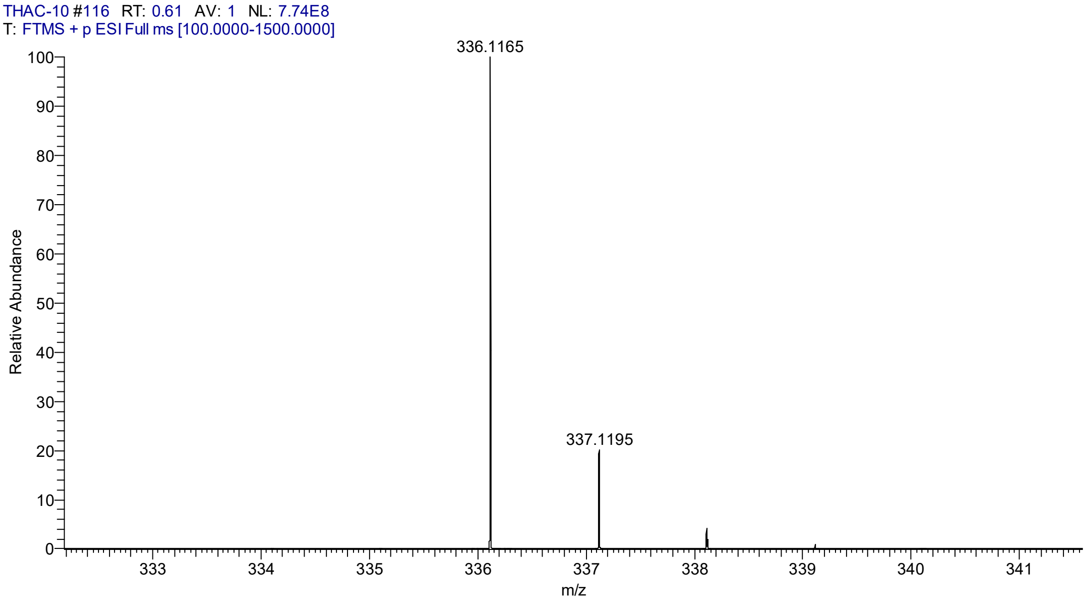

The HRMS spectrogram of compound **E15**

Compound **E16**, *N′*-(4-methylbenzylidene)-2-(*p*-tolyl)thiazole-4-carbohydrazide

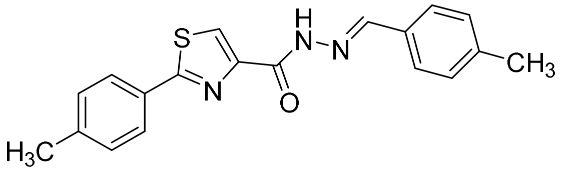

white solid, yield 68 %, m.p. 144.4-145.4 °C; ^{1}H NMR (400 MHz, DMSO-d_6) δ 11.71 (s, 1H), 8.62 (s, 1H), 8.48 (s, 1H), 7.95 (s, 1H), 7.91 (d, *J* = 7.6 Hz, 1H), 7.64 (d, *J* = 8.0 Hz, 2H), 7.44 (t, *J* = 7.6 Hz, 1H), 7.36 (d, *J* = 7.6 Hz, 1H), 7.29 (d, *J* = 8.0 Hz, 2H), 2.42 (s, 3H), 2.35 (s, 3H). ^{13}C NMR (101 MHz, DMSO-d_6) *δ* 168.13, 157.31, 149.90, 149.37, 140.52, 139.16, 132.84, 132.09, 132.03, 129.96, 129.61, 127.65, 127.44, 125.92, 124.39, 21.53, 21.37. HRMS(ESI) calcd for $C_{19}H_{17}N_3OS$ [M+H]$^+$: 336.1165, found 336.1164

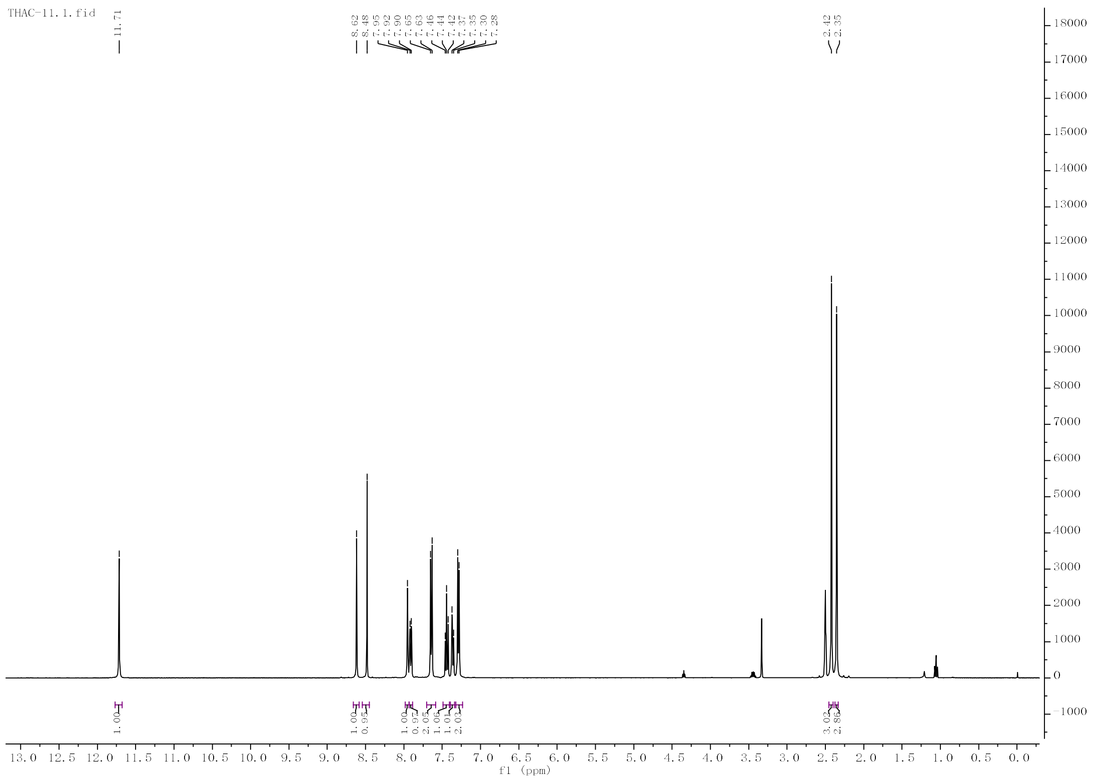

The ^{1}H NMR spectrogram of compound **E16**

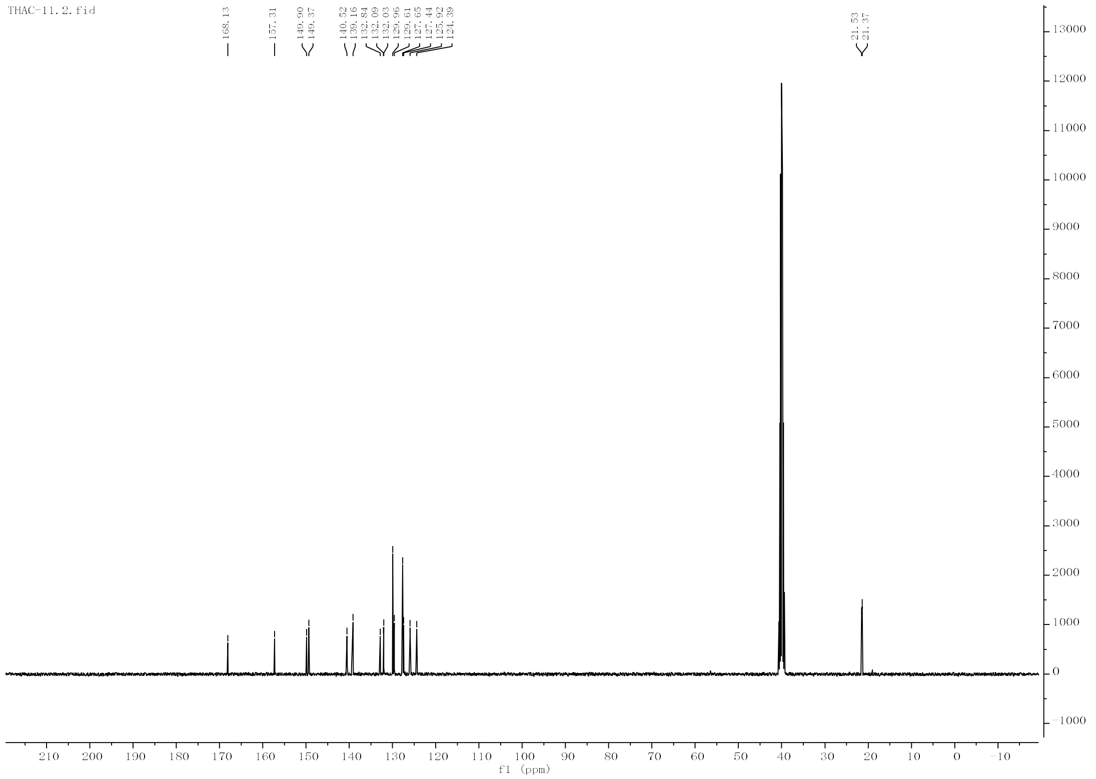

The ^{13}C NMR spectrogram of compound **E16**

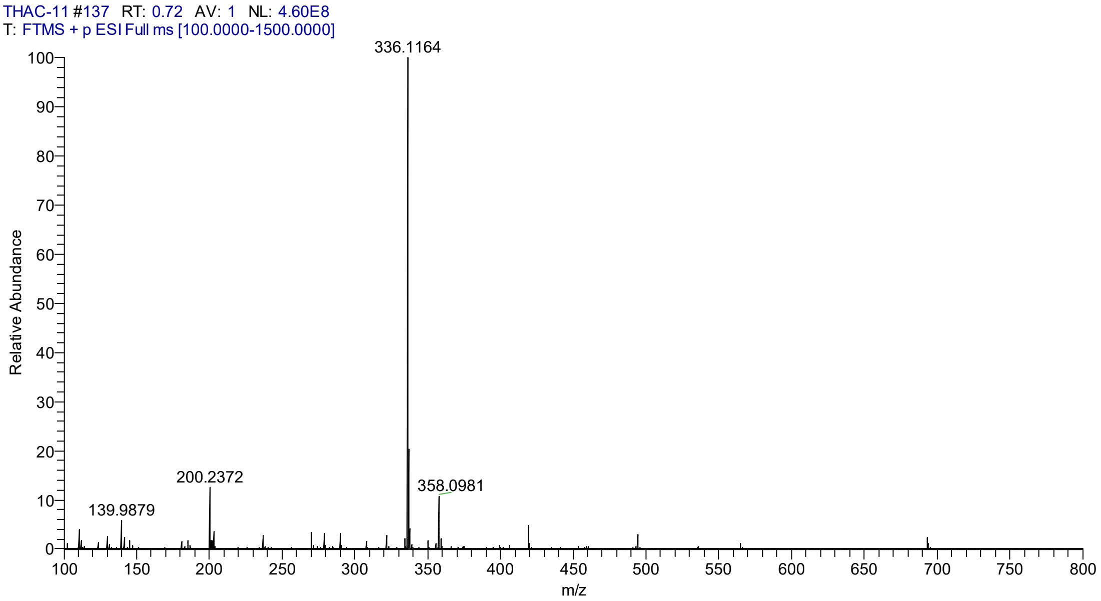

The HRMS spectrogram of compound **E16**

Compound **E17**

2-(2-chlorophenyl)-*N'*-(4-methylbenzylidene)thiazole-4-carbohydrazide:

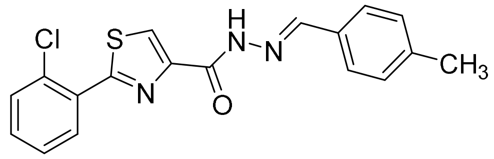

white solid, yield 63%, m.p. 170.6-172.5 °C; 1H NMR (400 MHz, $CDCl_3$) δ 10.36 (s, 1H), 8.40 (s, 1H), 8.30 (s, 1H), 8.21 (dd, J = 6.0, 3.6 Hz, 1H), 7.71 (d, J = 8.0 Hz, 2H), 7.60 – 7.49 (m, 1H), 7.46 – 7.34 (m, 2H), 7.22 (d, J = 7.9 Hz, 2H), 2.39 (s, 3H). ^{13}C NMR (101 MHz, $CDCl_3$) δ 163.91, 157.01, 148.90, 148.32, 141.00, 132.30, 131.10, 131.06, 130.93, 130.91, 129.45, 127.86, 127.18, 126.04, 126.01, 21.53. HRMS(ESI) calcd for $C_{18}H_{14}ClN_3OS$ $[M+H]^+$: 356.0619, found 356.0618.

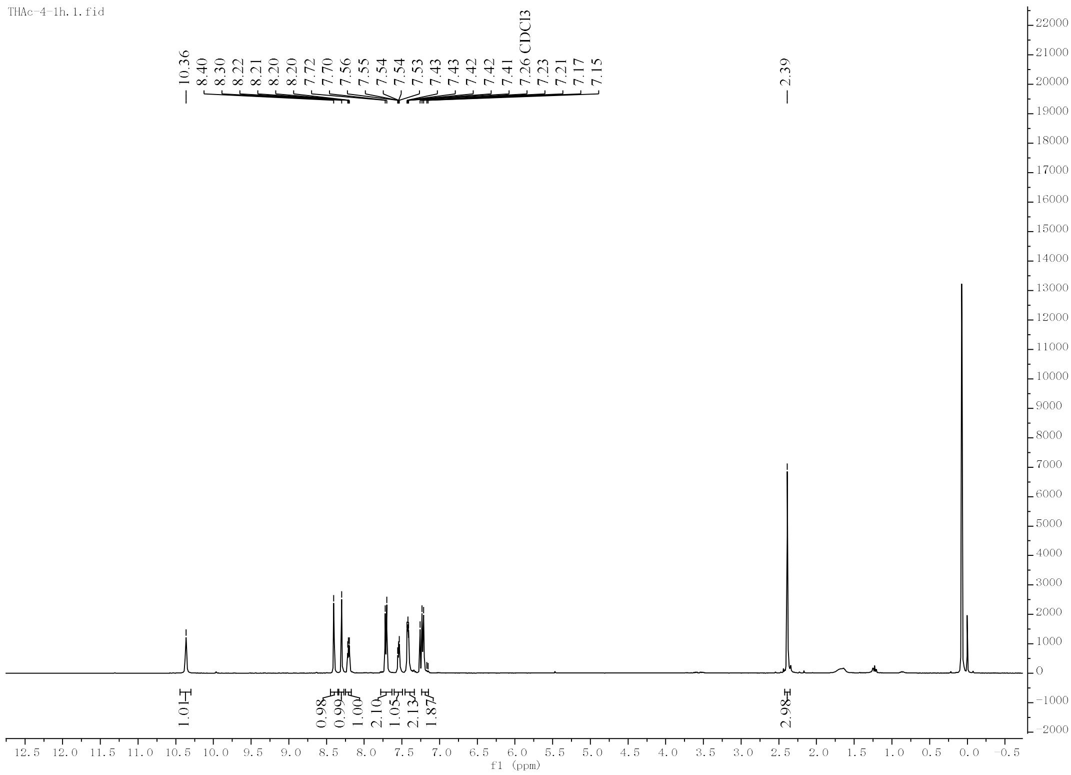

The ^1H NMR spectrogram of compound **E17**

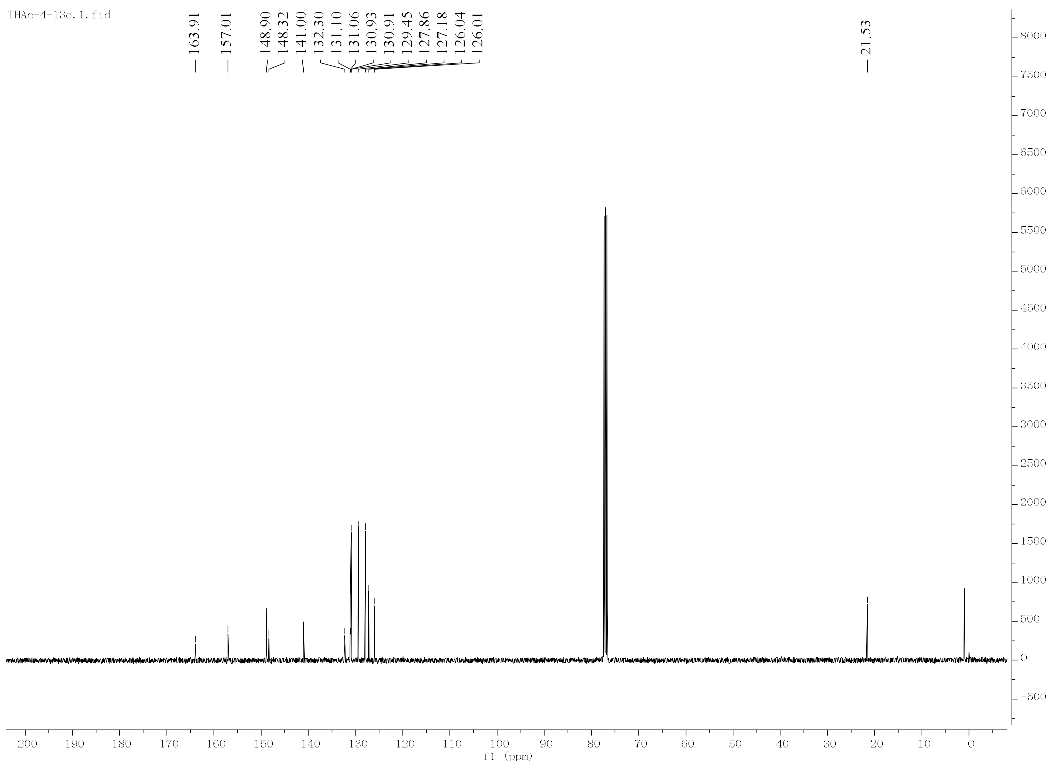

The ^{13}C NMR spectrogram of compound **E17**

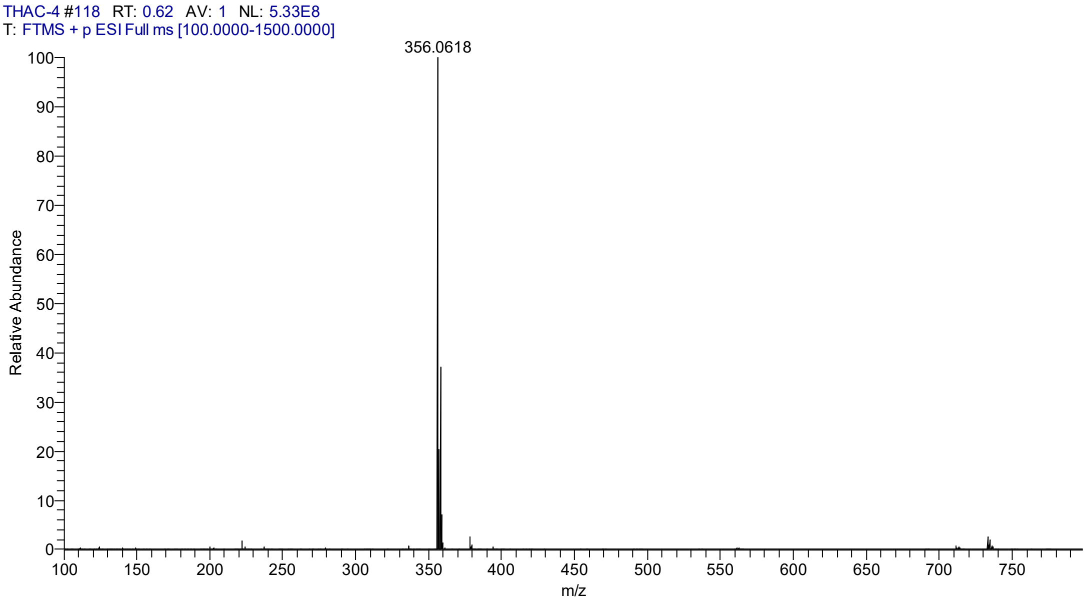

The HRMS spectrogram of compound **E17**

Compound **E18**, 2-(3-chlorophenyl)-*N'*-(4-methylbenzylidene)thiazole-4-carbohydrazide:

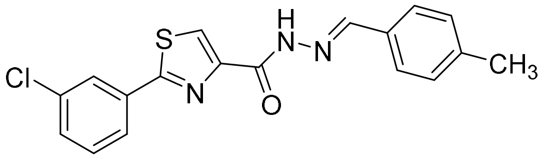

white solid, yield 78%, m.p. 179.1-180.8 °C; ^{1}H NMR (400 MHz, DMSO-d_6) δ 11.77 (s, 1H), 8.61 (s, 1H), 8.55 (s, 1H), 8.29 (s, 1H), 8.03 (d, J = 7.2 Hz, 1H), 7.67 – 7.56 (m, 4H), 7.29 (d, J = 8.0 Hz, 2H), 2.36 (s, 3H). ^{13}C NMR (101 MHz, DMSO-d_6) δ 166.16, 157.14, 149.96, 149.52, 140.57, 134.74, 134.61, 132.05, 131.68, 131.04, 129.97, 127.67, 126.81, 126.42, 125.97, 21.53. HRMS(ESI) calcd for $C_{18}H_{14}ClN_3OS$ $[M+H]^+$: 356.0619, found 356.0619.

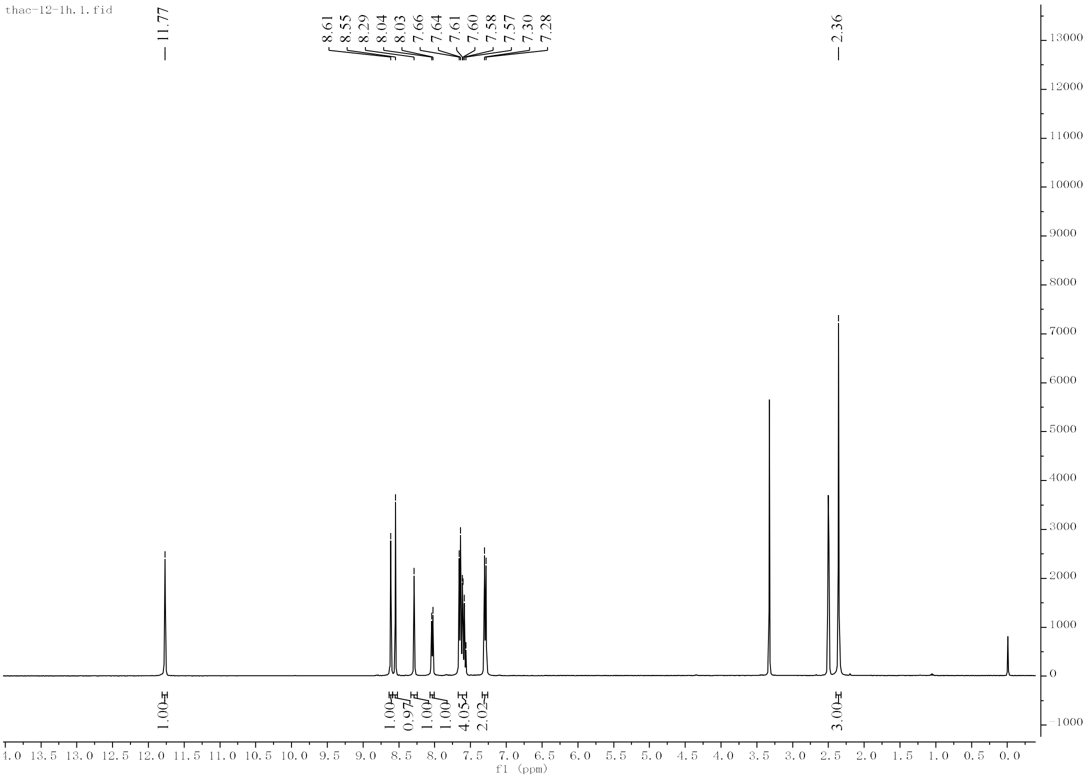

The ^{1}H NMR spectrogram of compound **E18**

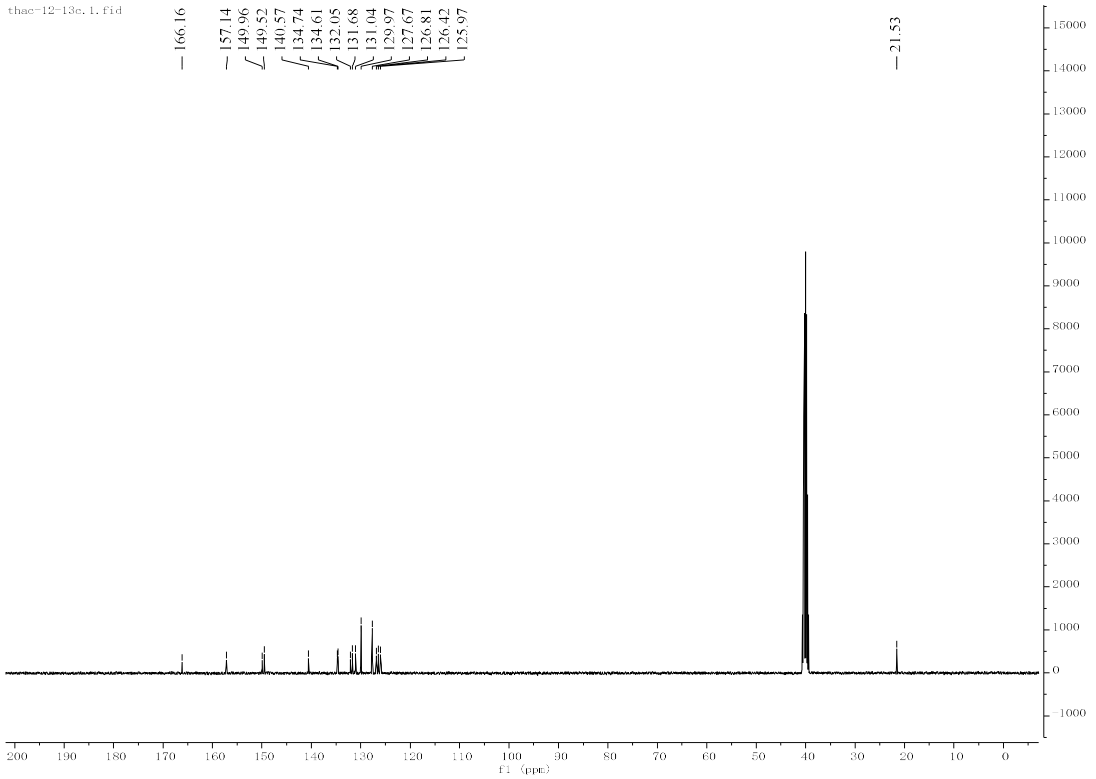

The ^{13}C NMR spectrogram of compound **E18**

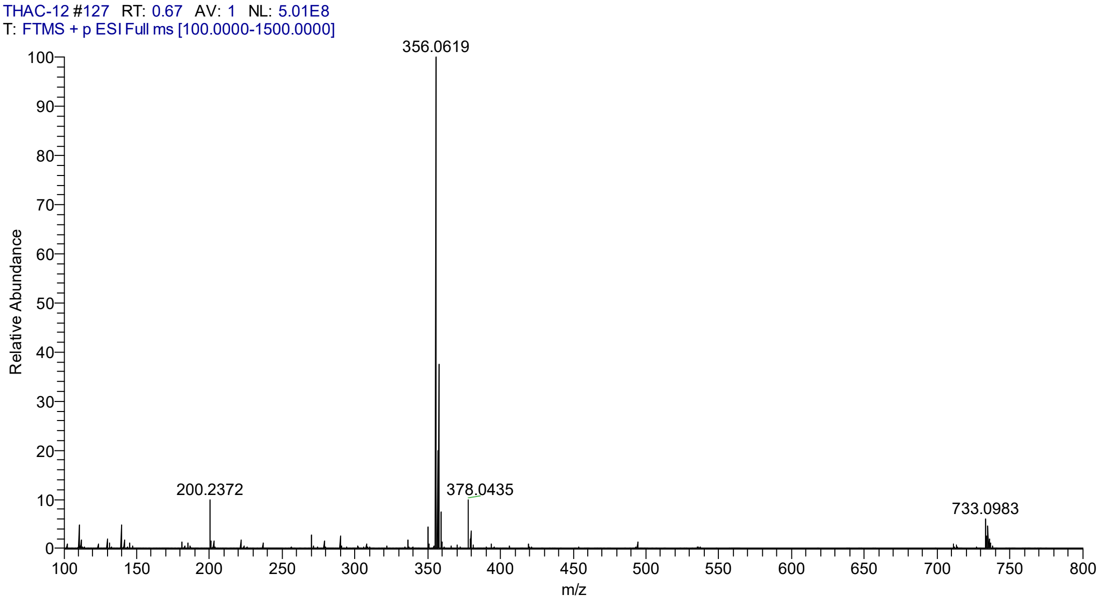

The HRMS spectrogram of compound **E18**

Compound **E19**, 2-(4-chlorophenyl)-*N′*-(4-methylbenzylidene)thiazole-4-carbohydrazide:

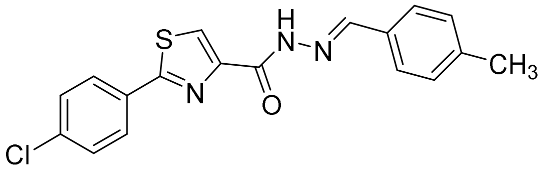

white solid, yield 62%, m.p. 176.4-178.6 °C; ^{1}H NMR (400 MHz, DMSO-d_6) δ 11.74 (s, 1H), 8.55 (d, J = 29.6 Hz, 2H), 8.15 (d, J = 8.4 Hz, 2H), 7.64 (d, J = 8.0 Hz, 4H), 7.29 (d, J = 7.6 Hz, 2H), 2.35 (s, 3H). ^{13}C NMR (101 MHz, DMSO-d_6) δ 166.59, 157.19, 149.99, 149.42, 140.55, 135.96, 132.05, 131.73, 129.96, 129.77, 128.83, 127.66, 126.44, 21.53. HRMS(ESI) calcd for $C_{18}H_{14}ClN_3OS$ $[M+H]^+$: 356.0619, found 356.0619.

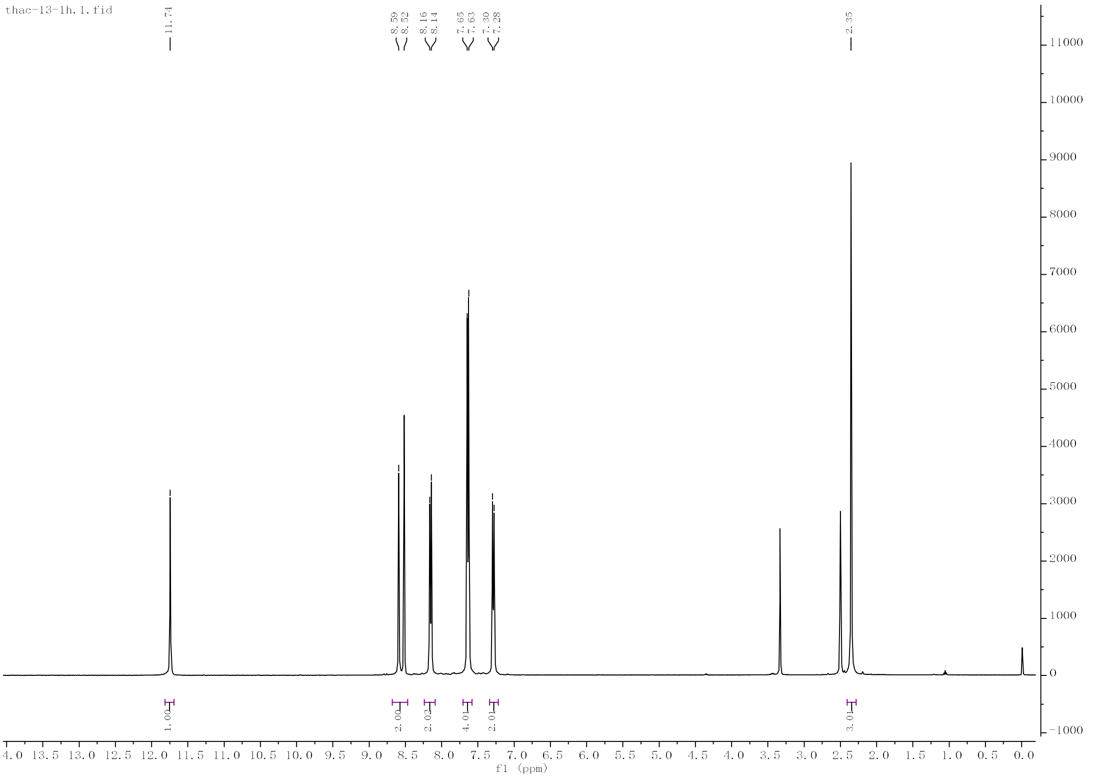

The ^{1}H NMR spectrogram of compound **E19**

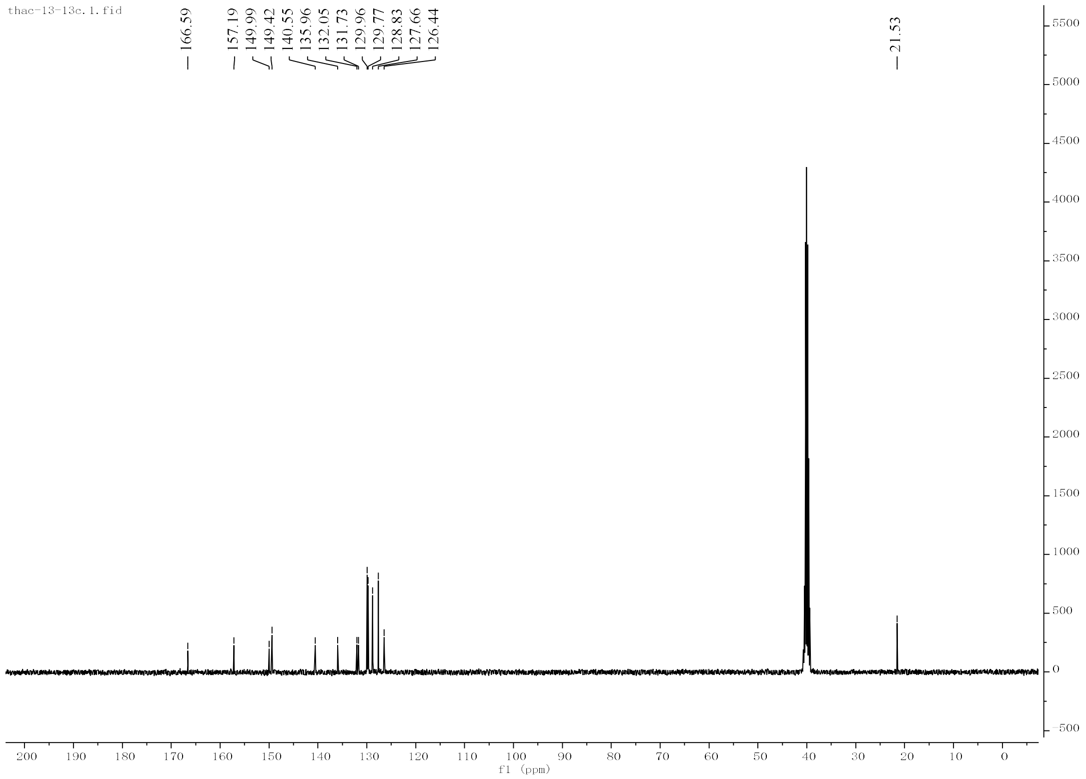

The ^{13}C NMR spectrogram of compound **E19**

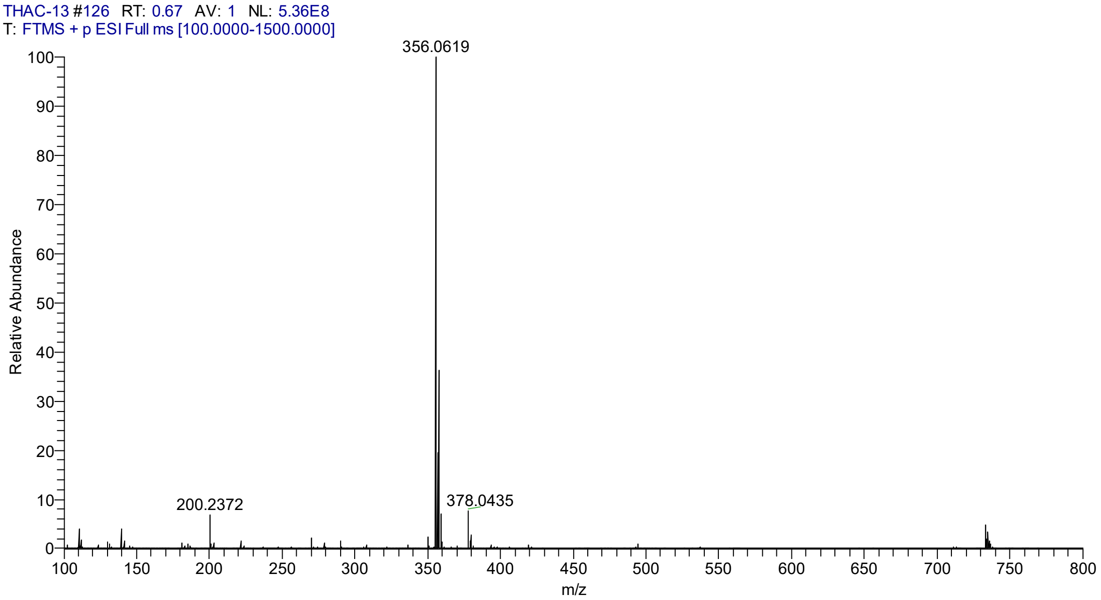

The HRMS spectrogram of compound **E19**

Compound **E20**, 2-(2-fluorophenyl)-*N'*-(4-methylbenzylidene)thiazole-4-carbohydrazide:

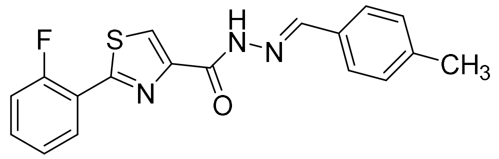

white solid, yield 66%, m.p. 169.9-172.5 °C; ^{1}H NMR (400 MHz, $CDCl_3$) δ 10.39 (s, 1H), 8.38 – 8.27 (m, 3H), 7.71 (d, J = 8.0 Hz, 2H), 7.46 (q, J = 6.0 Hz, 1H), 7.31 (t, J = 7.6 Hz, 1H), 7.22 (d, J = 8.0 Hz, 3H), 2.38 (s, 3H). ^{13}C NMR (101 MHz, $CDCl_3$) δ 161.11 (d, J = 5.4 Hz), 160.34 (d, J = 252.8 Hz), 157.12, 149.02, 148.56, 141.14, 132.10 (d, J = 8.6 Hz), 131.05, 129.58, 128.88 (d, J = 2.4 Hz), 127.99, 125.86 (d, J = 8.9 Hz), 124.84 (d, J = 3.3 Hz), 120.66 (d, J = 11.4 Hz), 116.52 (d, J = 21.6 Hz), 21.68. HRMS(ESI) calcd for $C_{18}H_{14}FN_3OS$ $[M+H]^+$: 340.0914, found 340.0913.

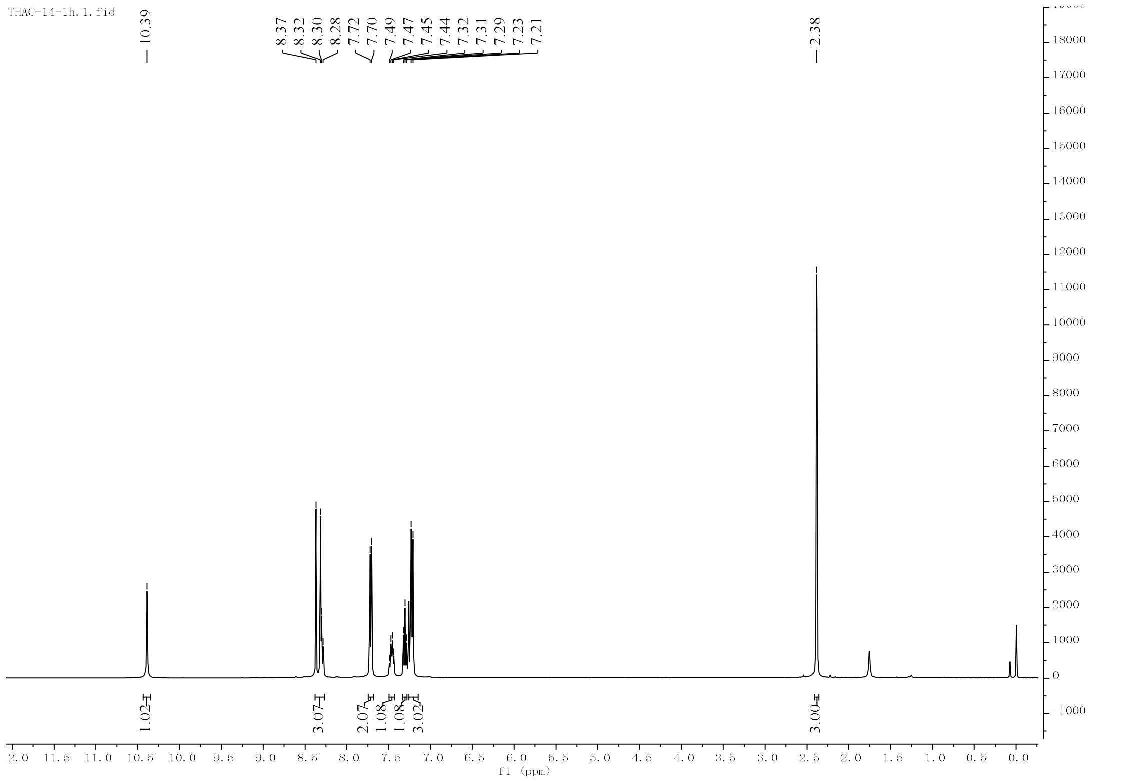

The ^{1}H NMR spectrogram of compound **E20**

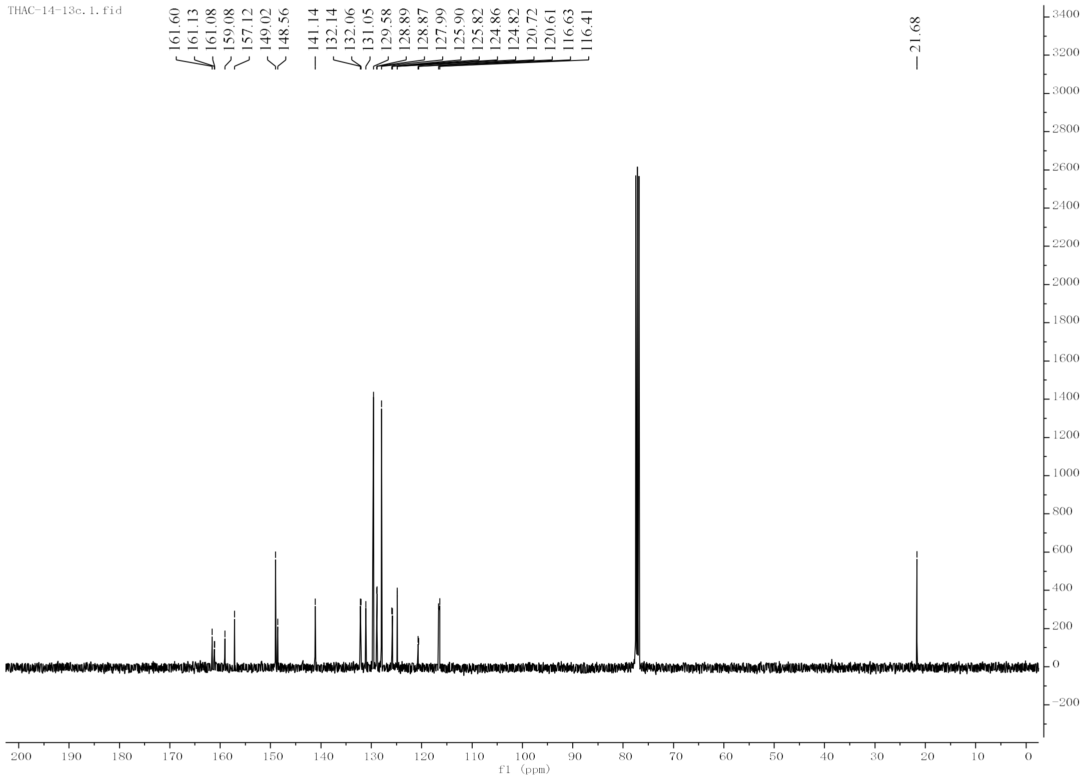

The ^{13}C NMR spectrogram of compound **E20**

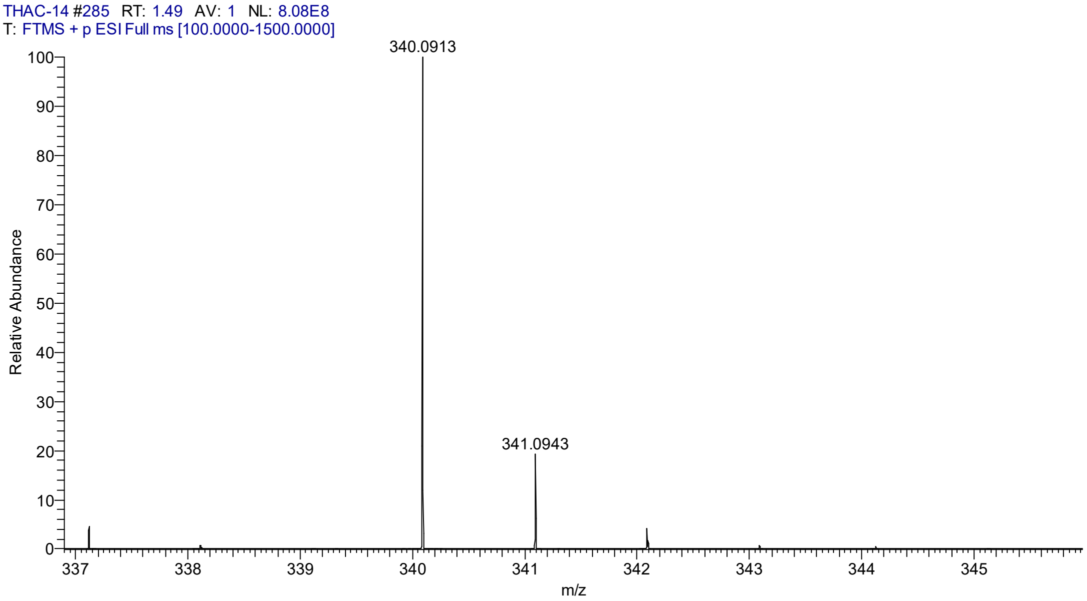

The HRMS spectrogram of compound **E20**

Compound **E21**, 2-(3-fluorophenyl)-*N′*-(4-methylbenzylidene)thiazole-4-carbohydrazide:

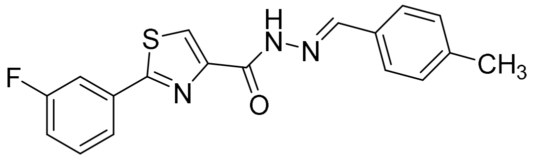

white solid, yield 66%, m.p. 188.0-181.3 °C; ^{1}H NMR (400 MHz, DMSO-d_6) δ 11.75 (s, 1H), 8.61 (s, 1H), 8.54 (s, 1H), 8.06 (dt, J = 10.0, 2.0 Hz, 1H), 7.92 (d, J = 7.6 Hz, 1H), 7.68 – 7.57 (m, 3H), 7.40 (td, J = 8.4, 2.4 Hz, 1H), 7.29 (d, J = 8.0 Hz, 2H), 2.35 (s, 3H). ^{13}C NMR (101 MHz, DMSO-d_6) δ 165.82 (d, J = 3.2 Hz), 162.52 (d, J = 244.4 Hz), 156.63, 149.35, 148.95, 140.08, 134.54 (d, J = 8.5 Hz), 131.54, 131.46 (d, J = 8.5 Hz), 129.48, 127.17, 126.26, 122.92 (d, J = 2.9 Hz), 117.62 (d, J = 21.2 Hz), 113.13 (d, J = 23.6 Hz), 21.05. HRMS(ESI) calcd for $C_{18}H_{14}FN_3OS$ $[M+H]^+$: 340.0914, found 340.0914.

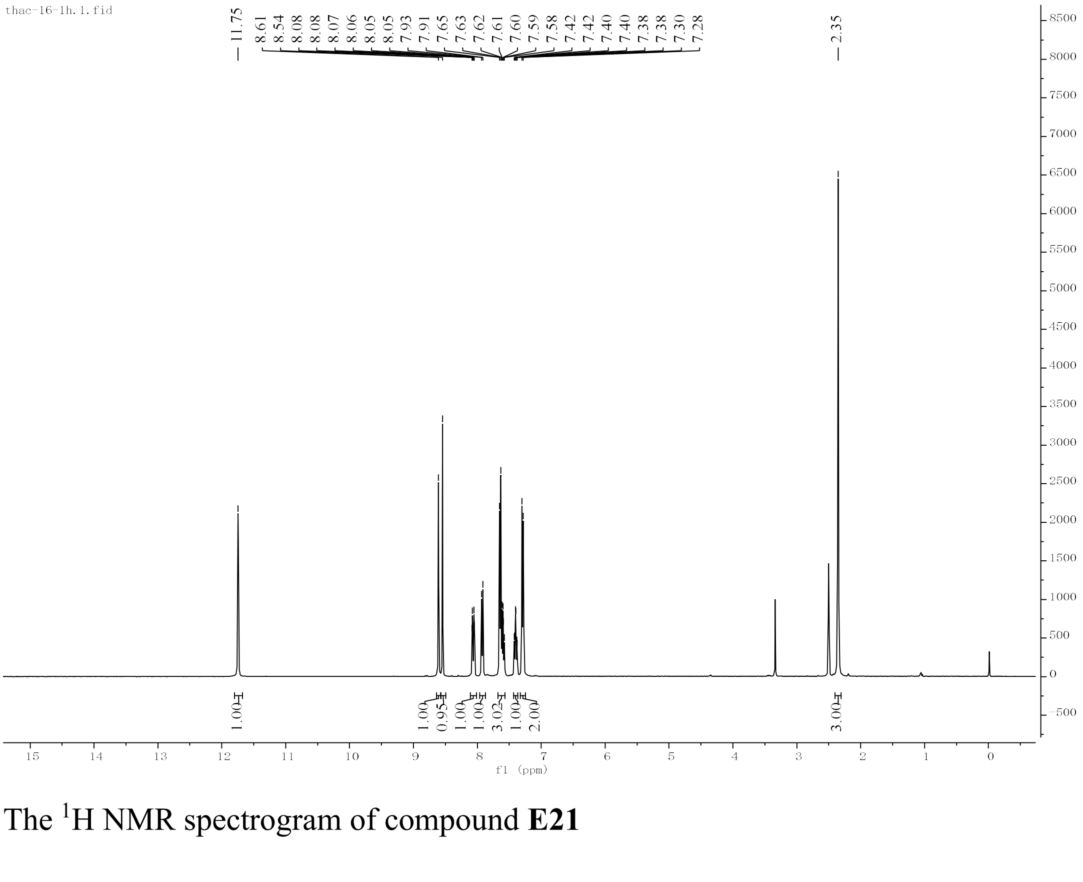

The ^{1}H NMR spectrogram of compound **E21**

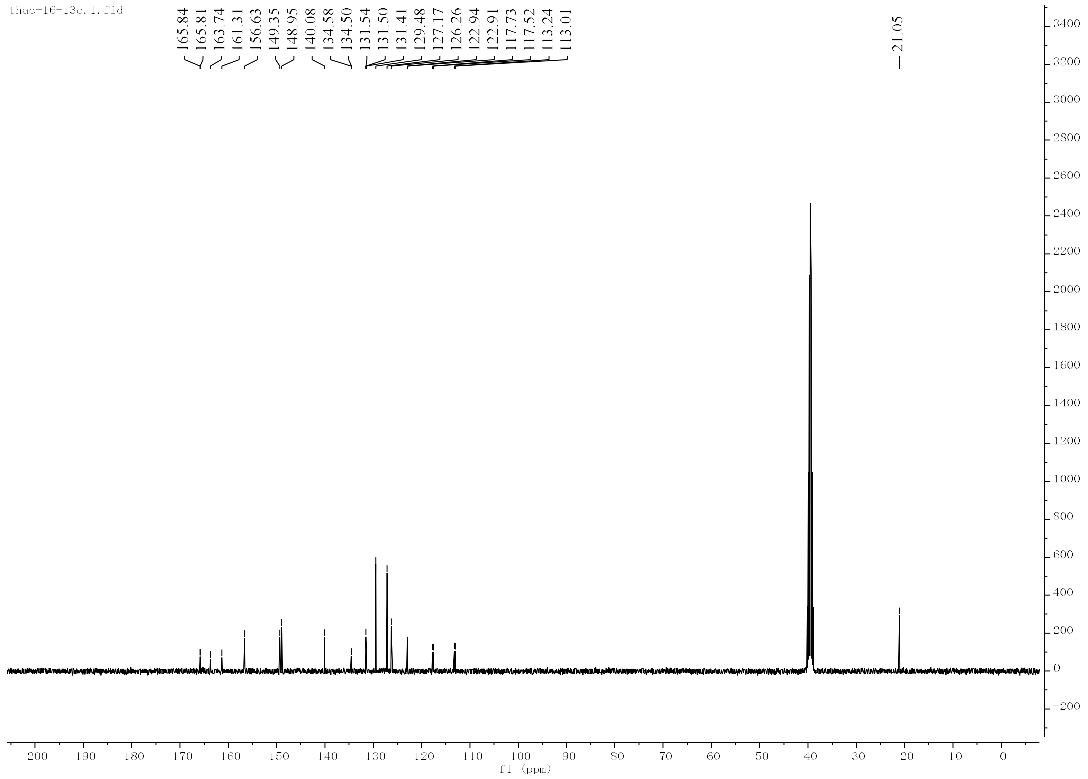

The ^{13}C NMR spectrogram of compound **E21**

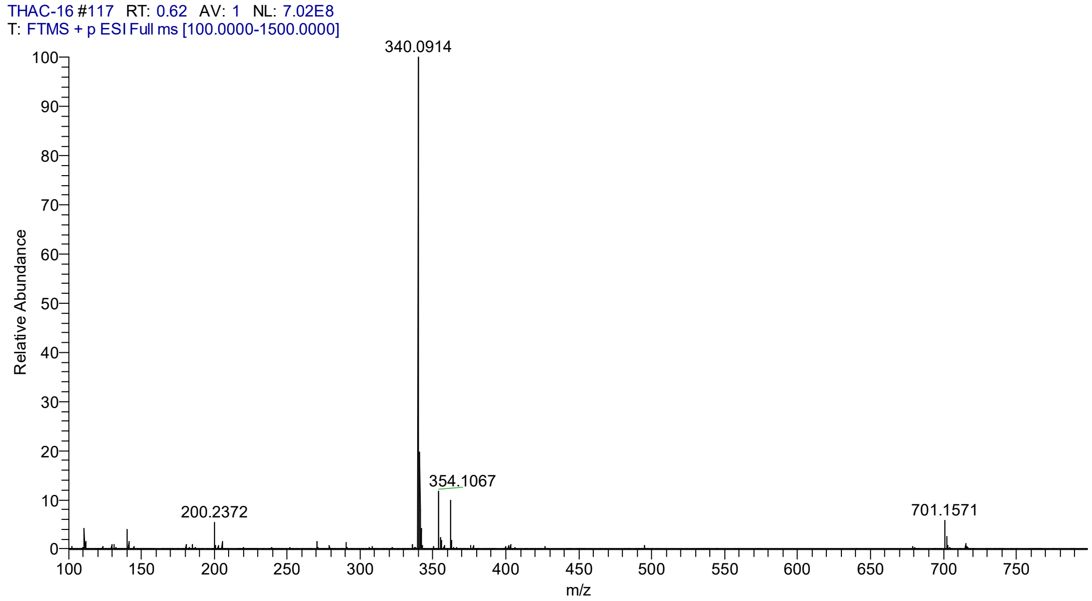

The HRMS spectrogram of compound **E21**

Compound **E22**, 2-(4-fluorophenyl)-*N'*-(4-methylbenzylidene)thiazole-4-carbohydrazide:

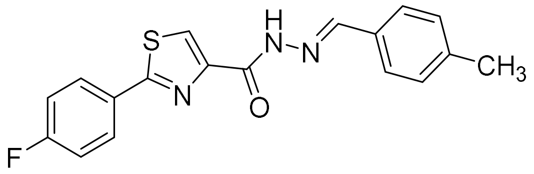

white solid, yield 70%, m.p. 180.0-181.3 °C; ^{1}H NMR (400 MHz, DMSO-d_6) δ 11.73 (s, 1H), 8.59 (s, 1H), 8.49 (s, 1H), 8.19 (dd, J = 8.8, 5.6 Hz, 2H), 7.64 (d, J = 8.0 Hz, 2H), 7.42 (t, J = 8.8 Hz, 2H), 7.29 (d, J = 8.0 Hz, 2H), 2.35 (s, 3H). ^{13}C NMR (101 MHz, DMSO-d_6) δ 166.25, 163.54 (d, J = 249.0 Hz), 156.72, 149.35, 148.86, 140.02, 131.53, 129.44, 129.05, 128.96, 127.13, 125.57, 116.25 (d, J = 22.1 Hz), 21.01. HRMS(ESI) calcd for $C_{18}H_{14}FN_3OS$ $[M+H]^+$: 340.0914, found 340.0914.

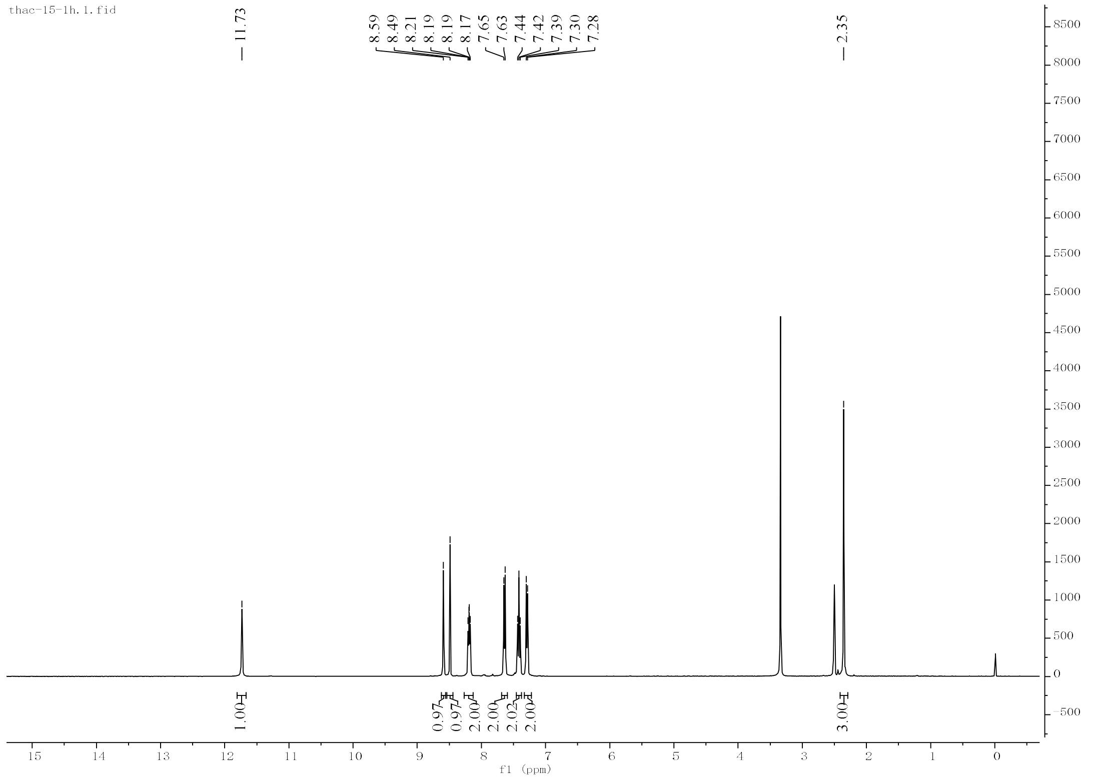

The ^{1}H NMR spectrogram of compound **F22**

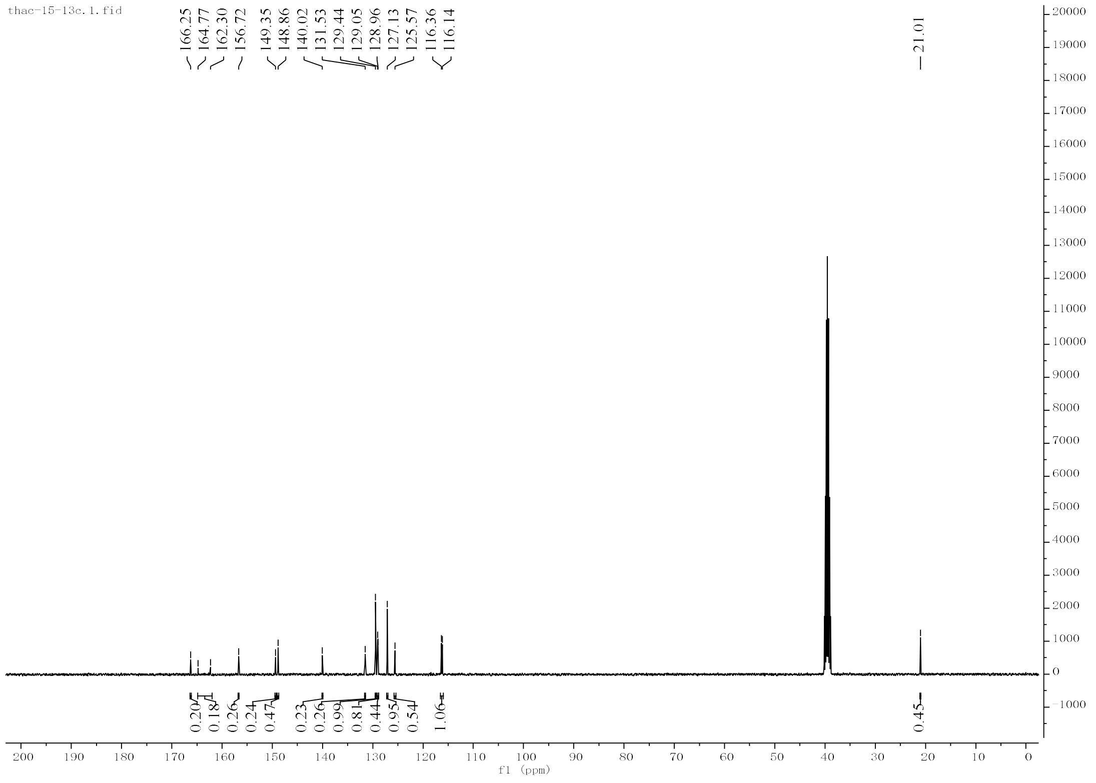

The ^{13}C NMR spectrogram of compound **E22**

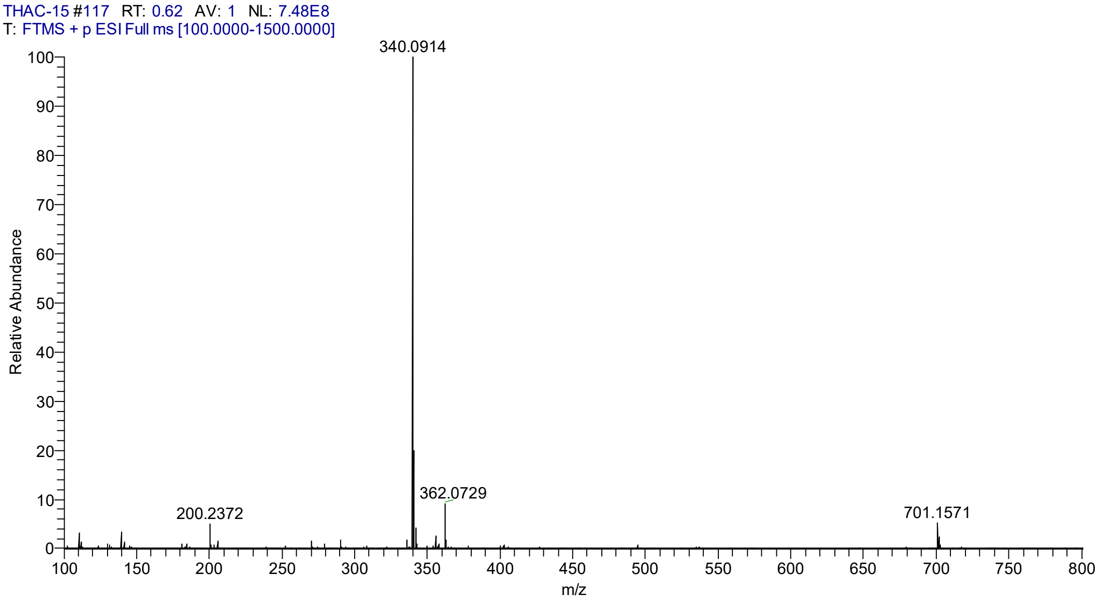

The HRMS spectrogram of compound **E22**

Compound **E23**, 2-(2-bromophenyl)-*N'*-(4-methylbenzylidene)thiazole-4-carbohydrazide:

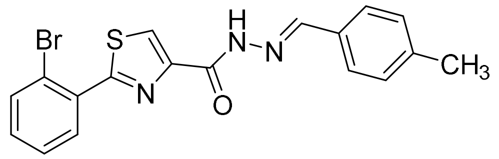

yellow solid, yield 75%, m.p. 172.7-175.3 °C; ^{1}H NMR (400 MHz, $CDCl_3$) δ 10.35 (s, 1H), 8.41 (s, 1H), 8.27 (s, 1H), 8.01 (dd, J = 7.6, 1.6 Hz, 1H), 7.78 – 7.69 (m, 3H), 7.46 (td, J = 7.6, 1.2 Hz, 1H), 7.34 (td, J = 7.6, 1.8 Hz, 1H), 7.22 (d, J = 8.0 Hz, 2H), 2.39 (s, 3H). ^{13}C NMR (101 MHz, $CDCl_3$) δ 165.41, 156.99, 148.81, 148.44, 141.05, 134.31, 133.15, 131.61, 131.31, 130.85, 129.46, 127.88, 127.72, 126.04, 121.83, 21.57. HRMS(ESI) calcd for $C_{18}H_{14}BrN_3OS$ $[M+H]^+$: 400.0114, found 400.0114.

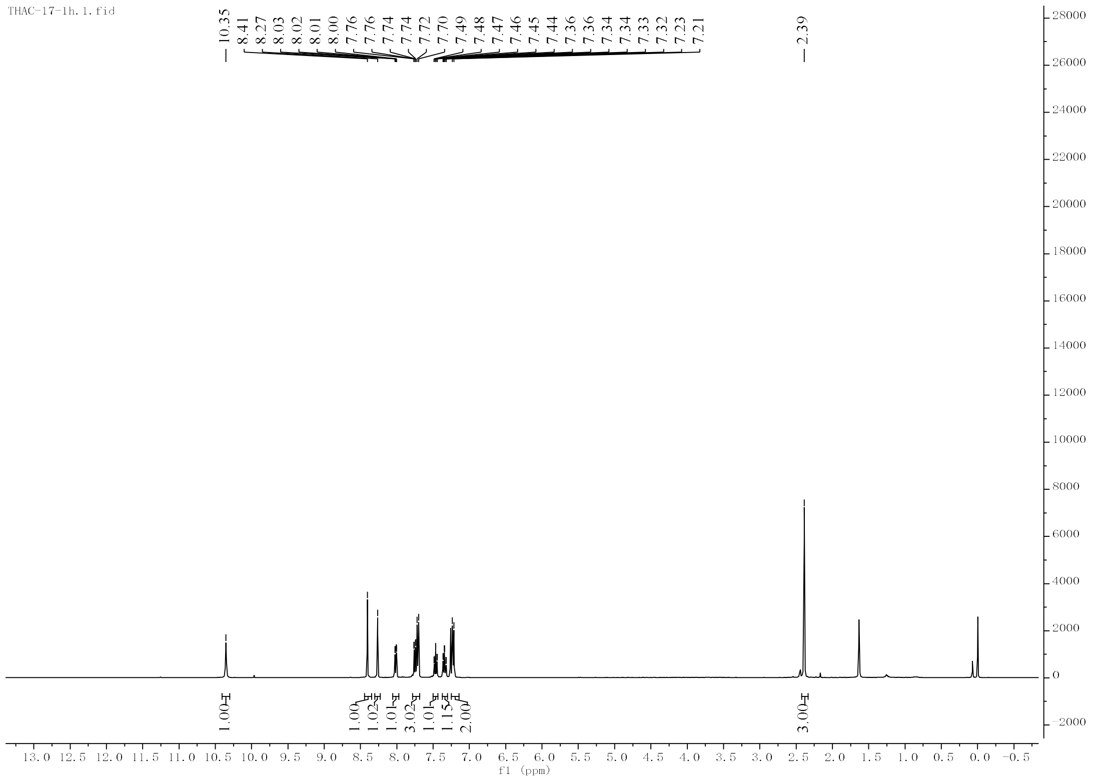

The ^{1}H NMR spectrogram of compound **E23**

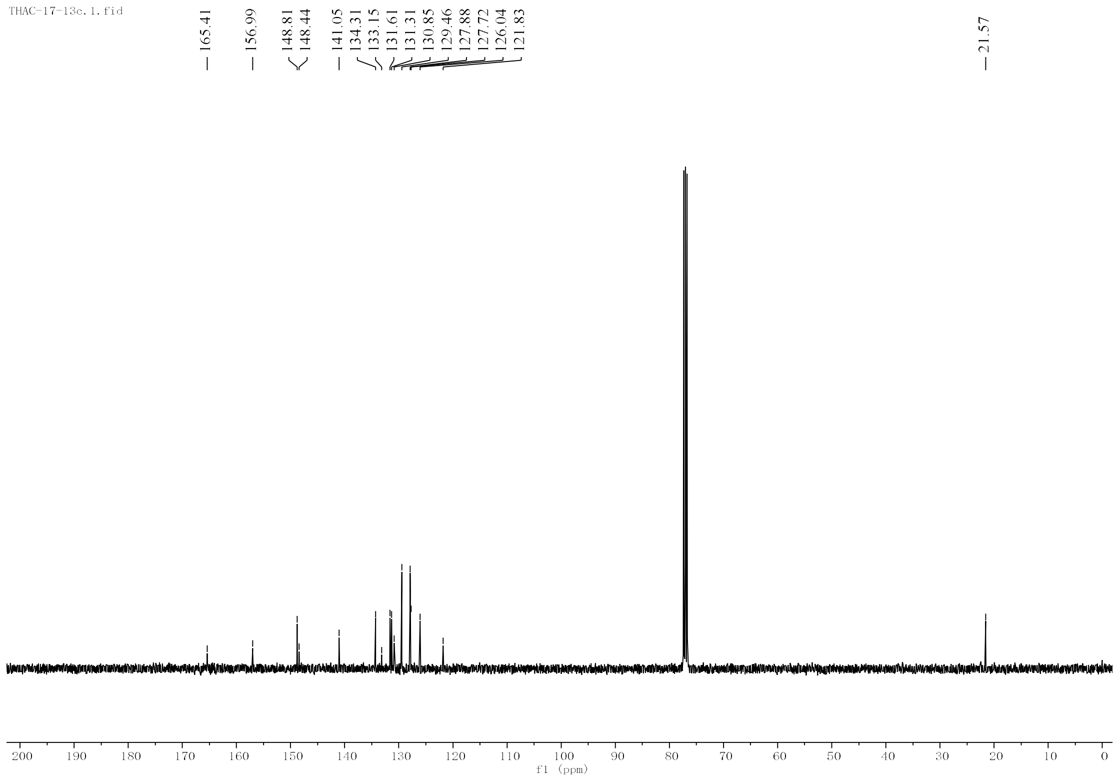

The ^{13}C NMR spectrogram of compound **E23**

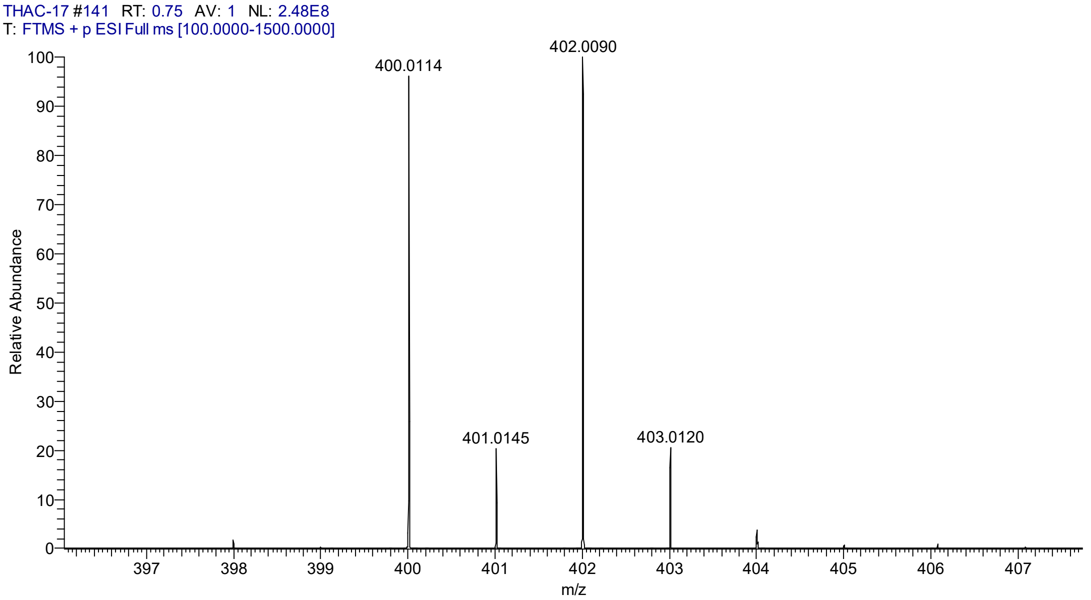

The HRMS spectrogram of compound **E23**

Compound **E24**, 2-(3-bromophenyl)-*N′*-(4-methylbenzylidene)thiazole-4-carbohydrazide:

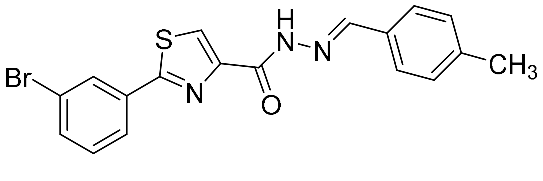

white solid, yield, 71%, m.p. 190.7-192.9 °C; ^{1}H NMR (400 MHz, DMSO-d_6) δ 11.77 (s, 1H), 8.62 (s, 1H), 8.54 (s, 1H), 8.45 – 8.37 (m, 1H), 8.06 (d, J = 8.0 Hz, 1H), 7.76 – 7.72 (m, 1H), 7.65 (d, J = 8.0 Hz, 2H), 7.51 (t, J = 8.0 Hz, 1H), 7.29 (d, J = 8.0 Hz, 2H), 2.35 (s, 3H). ^{13}C NMR (101 MHz, DMSO-d_6) δ 166.06, 157.15, 149.98, 149.52, 140.56, 134.91, 133.94, 132.05, 131.89, 129.96, 129.20, 127.67, 126.84, 126.35, 123.10, 21.54. HRMS(ESI) calcd for $C_{18}H_{14}BrN_3OS$ [M+H]$^+$: 400.0114, found 400.0115.

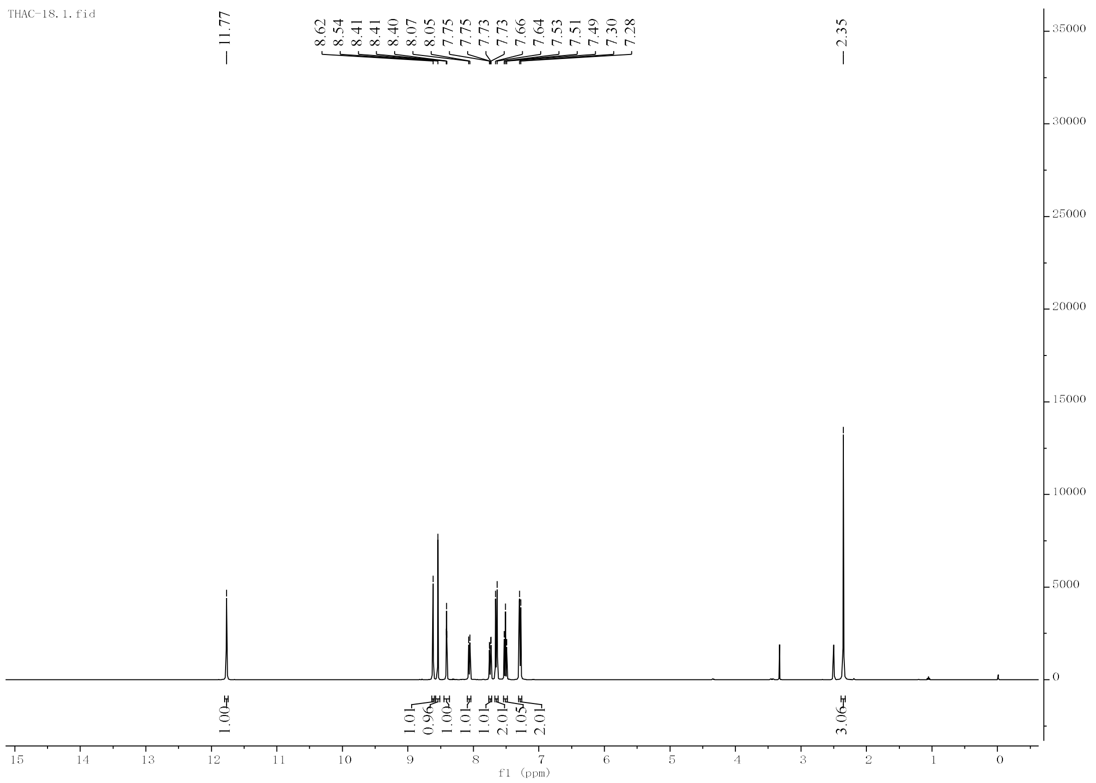

The ^{1}H NMR spectrogram of compound **E24**

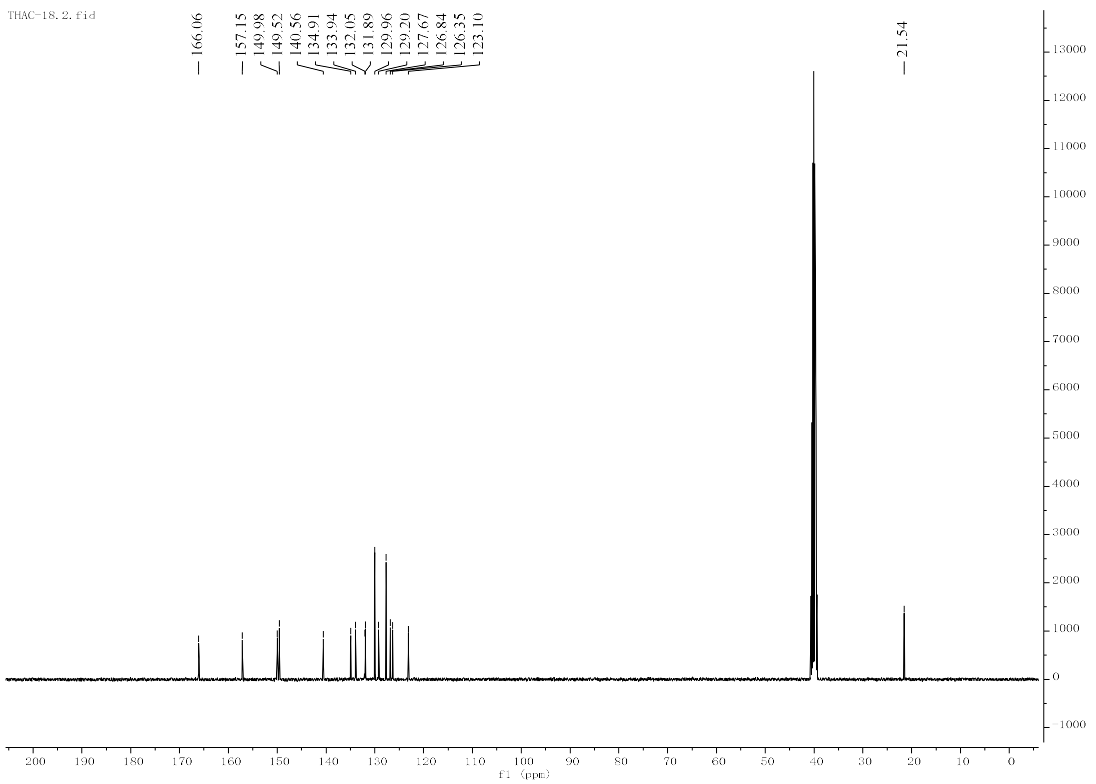

The ^{13}C NMR spectrogram of compound **E24**

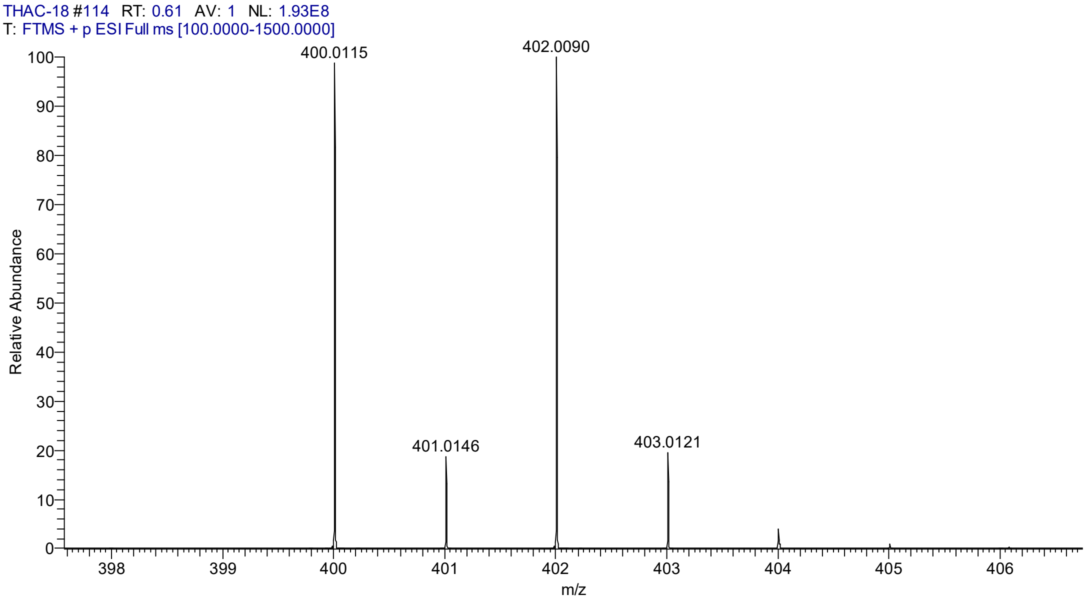

The HRMS spectrogram of compound **E24**

Compound **E25**, 2-(4-bromophenyl)-*N'*-(4-methylbenzylidene)thiazole-4-carbohydrazide:

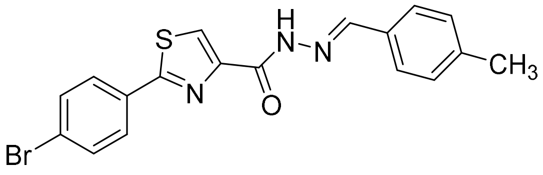

white solid, yield, 74%, m.p. 182.5-184.8 °C; ^{1}H NMR (400 MHz, DMSO-d_6) δ 11.74 (s, 1H), 8.56 (d, J = 31.6 Hz, 2H), 8.07 (d, J = 8.4 Hz, 2H), 7.76 (d, J = 8.4 Hz, 2H), 7.63 (d, J = 8.0 Hz, 2H), 7.28 (d, J = 8.0 Hz, 2H), 2.34 (s, 3H). ^{13}C NMR (101 MHz, DMSO-d_6) δ 166.68, 157.19, 150.00, 149.41, 140.54, 132.67, 132.04, 129.95, 128.99, 127.66, 126.48, 124.77, 21.54. HRMS(ESI) calcd for $C_{18}H_{14}BrN_3OS$ $[M+H]^+$: 400.0114, found 400.0115.

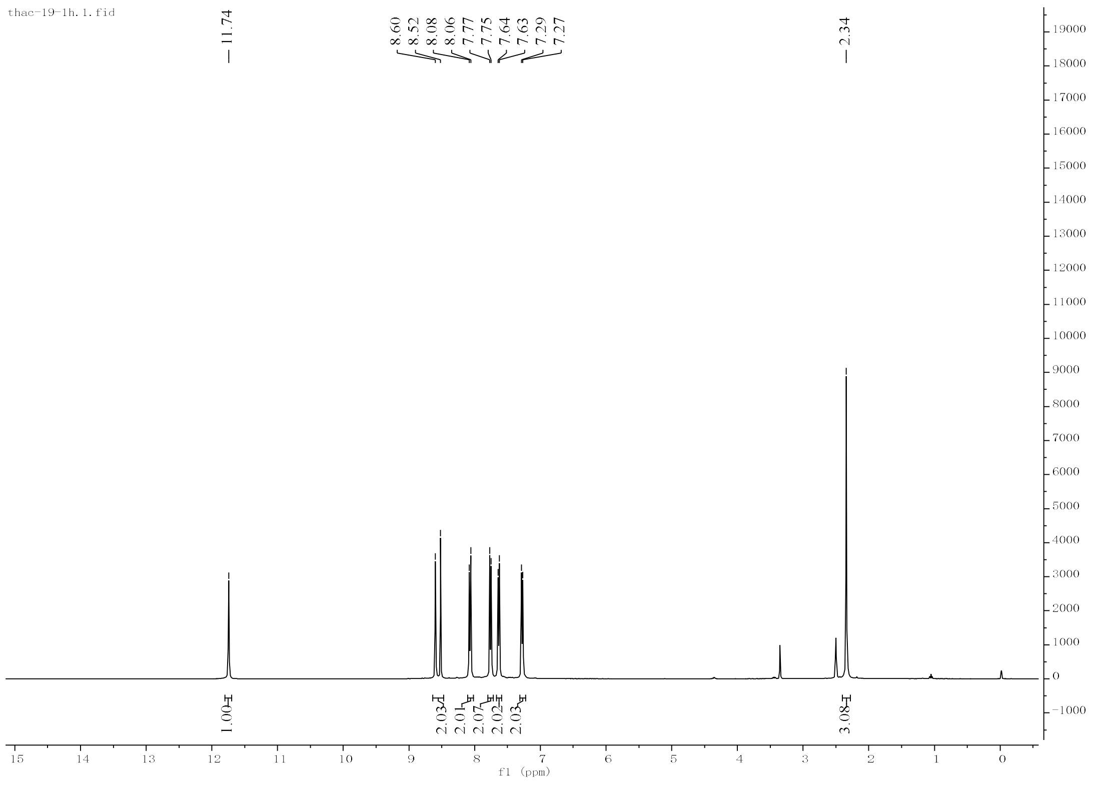

The ^{1}H NMR spectrogram of compound **E25**

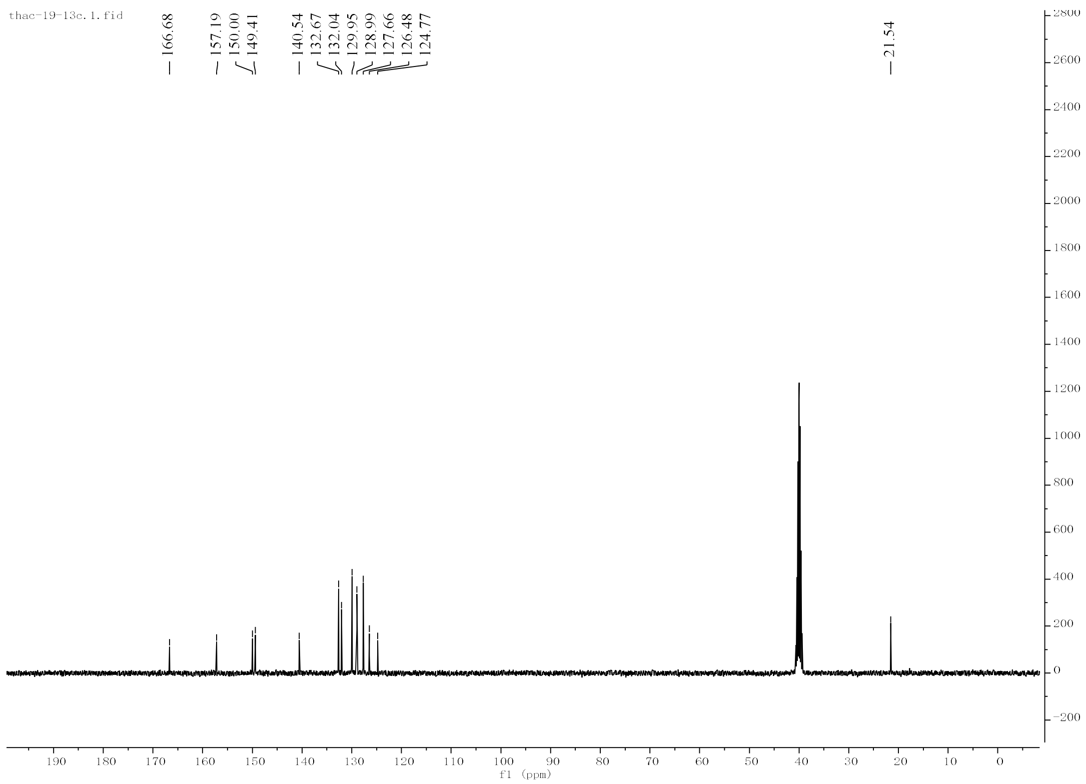

The ^{13}C NMR spectrogram of compound **E25**

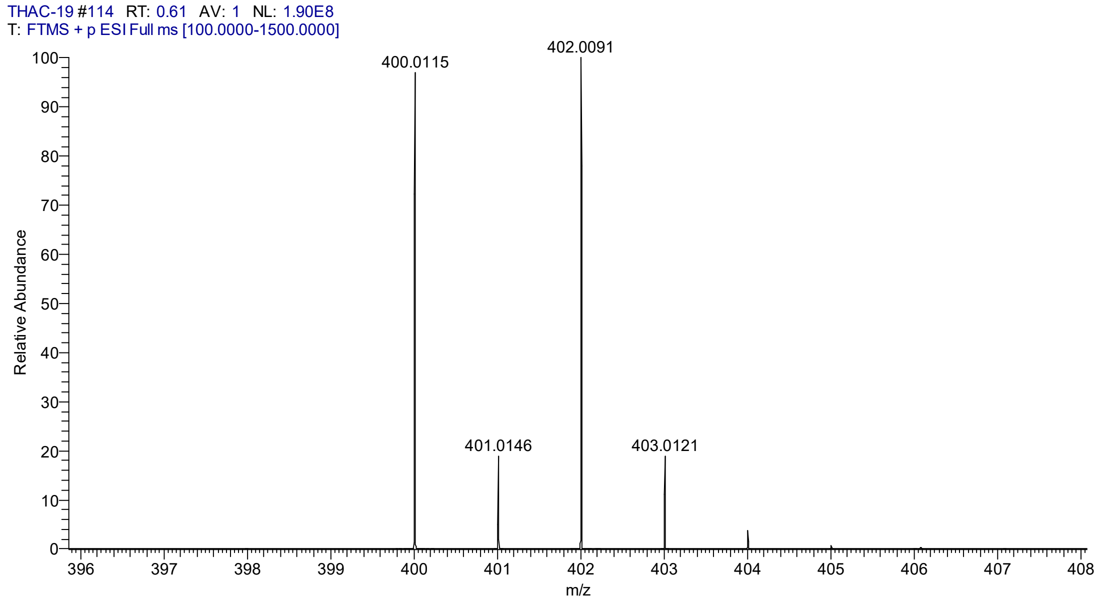

The HRMS spectrogram of compound **E25**

Compound **E26**, 2-(2-methoxyphenyl)-*N′*-(4-methylbenzylidene)thiazole-4-carbohydrazide:

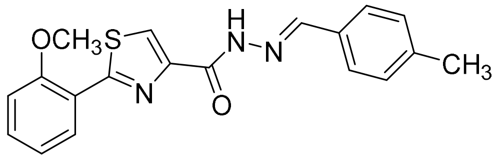

white solid, yield 73%, m.p. 138.2-140.1 °C; ^{1}H NMR (400 MHz, $CDCl_3$) δ 10.44 (s, 1H), 8.41 (dd, J = 8.0, 1.8 Hz, 1H), 8.31 (d, J = 4.0 Hz, 2H), 7.73 (d, J = 8.0 Hz, 2H), 7.49 – 7.41 (m, 1H), 7.23 (d, J = 8.0 Hz, 2H), 7.13 (t, J = 7.6 Hz, 1H), 7.07 (d, J = 8.4 Hz, 1H), 4.05 (s, 3H), 2.39 (s, 3H). ^{13}C NMR (101 MHz, $CDCl_3$) δ 162.64, 157.46, 156.70, 148.50, 147.46, 140.87, 131.48, 131.04, 129.43, 128.33, 127.83, 125.36, 121.43, 121.05, 111.52, 55.63, 21.54. HRMS(ESI) calcd for $C_{19}H_{17}N_3O_2S$ $[M+H]^+$: 352.1114, found 352.1113.

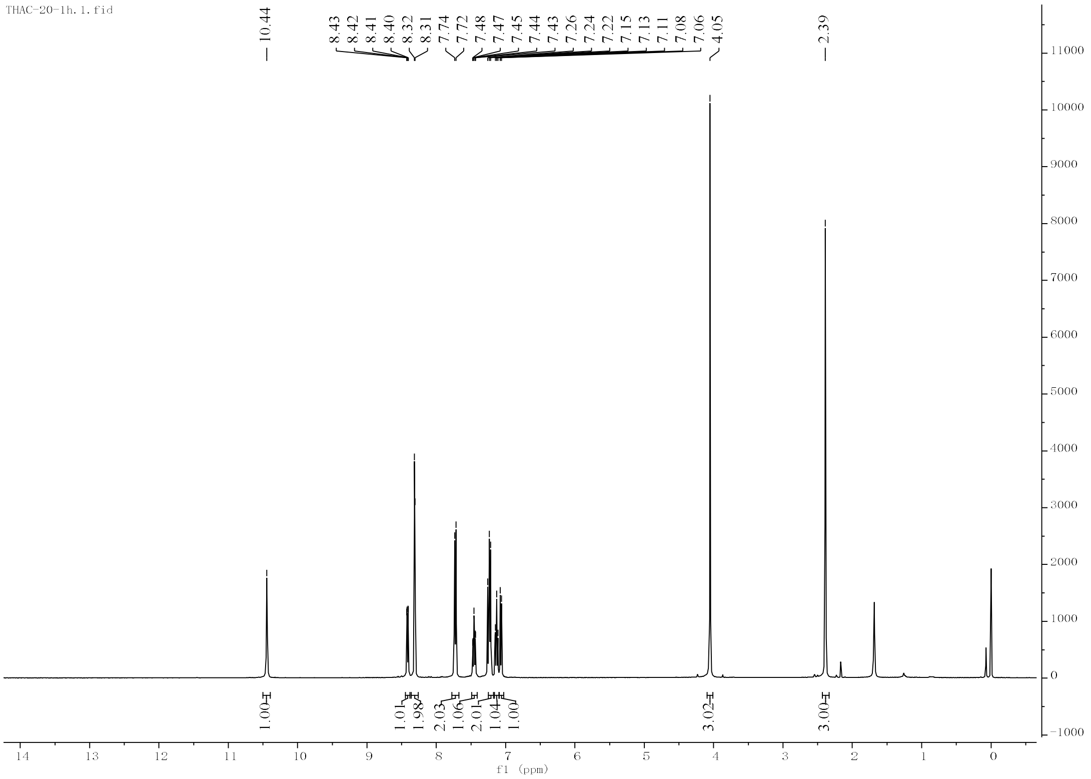

The ^{1}H NMR spectrogram of compound **E26**

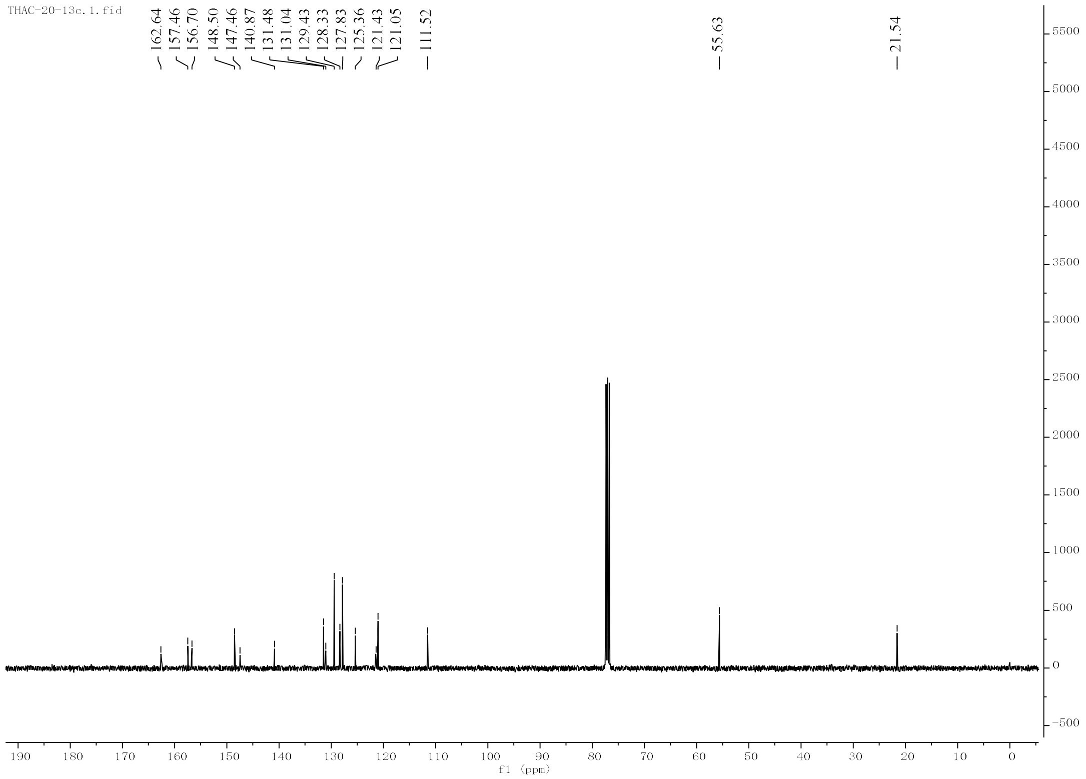

The ^{13}C NMR spectrogram of compound **E26**

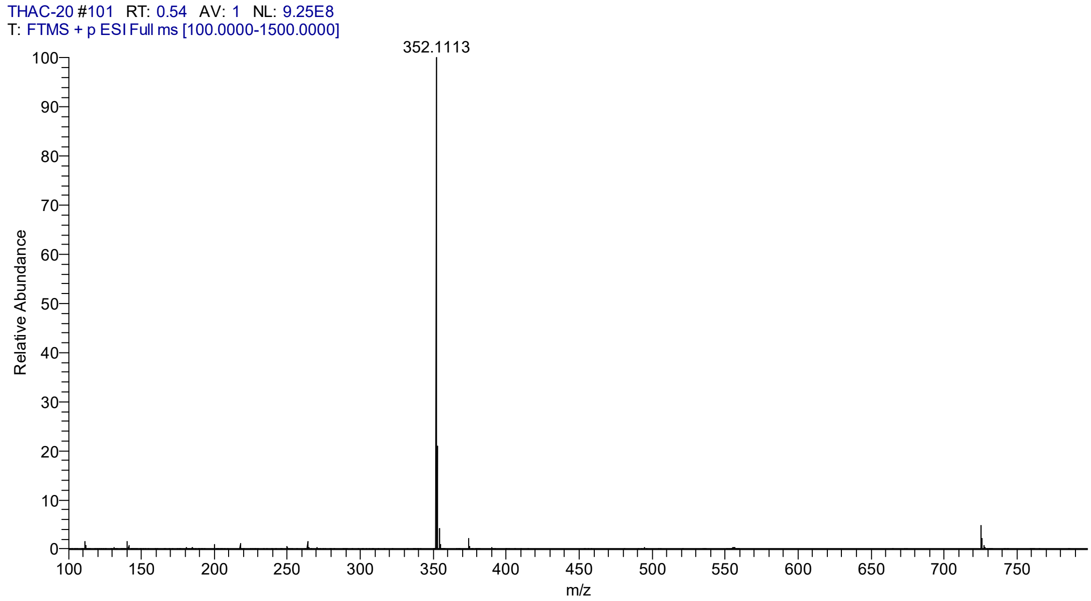

The HRMS spectrogram of compound **E26**

Compound **E27**, 2-(3-methoxyphenyl)-*N'*-(4-methylbenzylidene)thiazole-4-carbohydrazide:

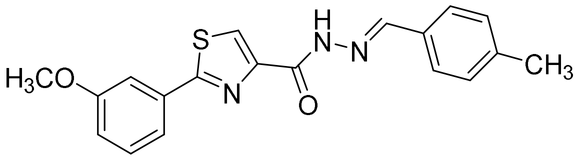

white solid, yield 66%, m.p. 117.4-119.9 °C;^{1}H NMR (400 MHz, DMSO-d_6) δ 11.73 (s, 1H), 8.62 (s, 1H), 8.50 (s, 1H), 7.71 (s, 1H), 7.66 (t, J = 6.8 Hz, 3H), 7.48 (t, J = 8.0 Hz, 1H), 7.30 (d, J = 8.0 Hz, 2H), 7.14 (dd, J = 8.4, 2.8 Hz, 1H), 3.89(s,3H), 2.36 (s, 3H). ^{13}C NMR (101 MHz, DMSO-d_6) δ 167.73, 160.30, 157.27, 149.84, 149.43,140.54,134.14, 132.07, 130.95, 129.97, 127.66, 126.15, 119.61, 116.93, 112.36, 55.94, 21.53. HRMS(ESI) calcd for $C_{19}H_{17}N_3O_2S$ [M+H]$^+$: 352.1114, found 352.1114.

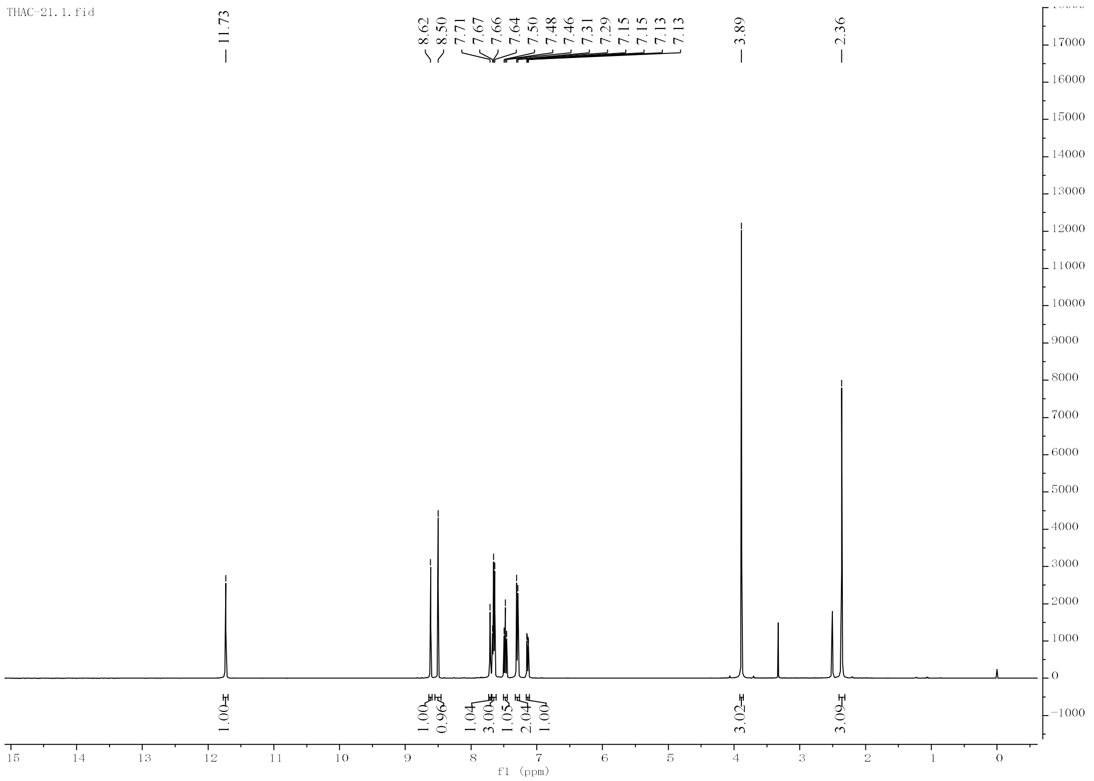

The ^{1}H NMR spectrogram of compound **E27**

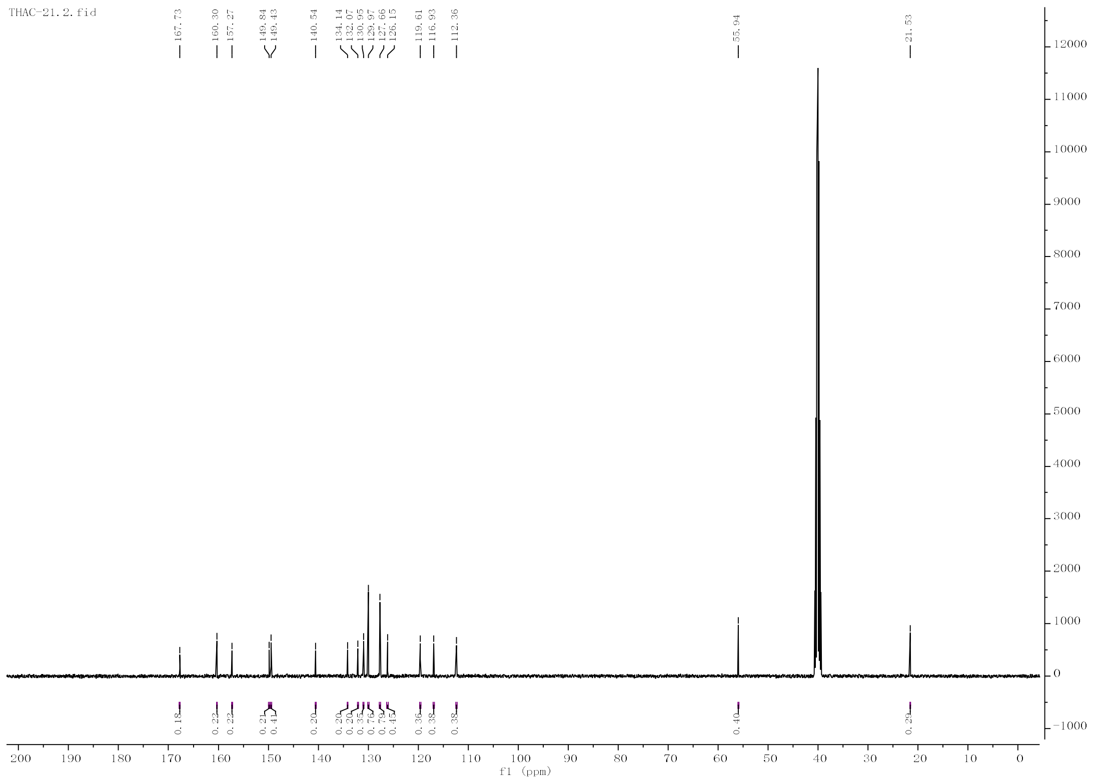

The ^{13}C NMR spectrogram of compound **E27**

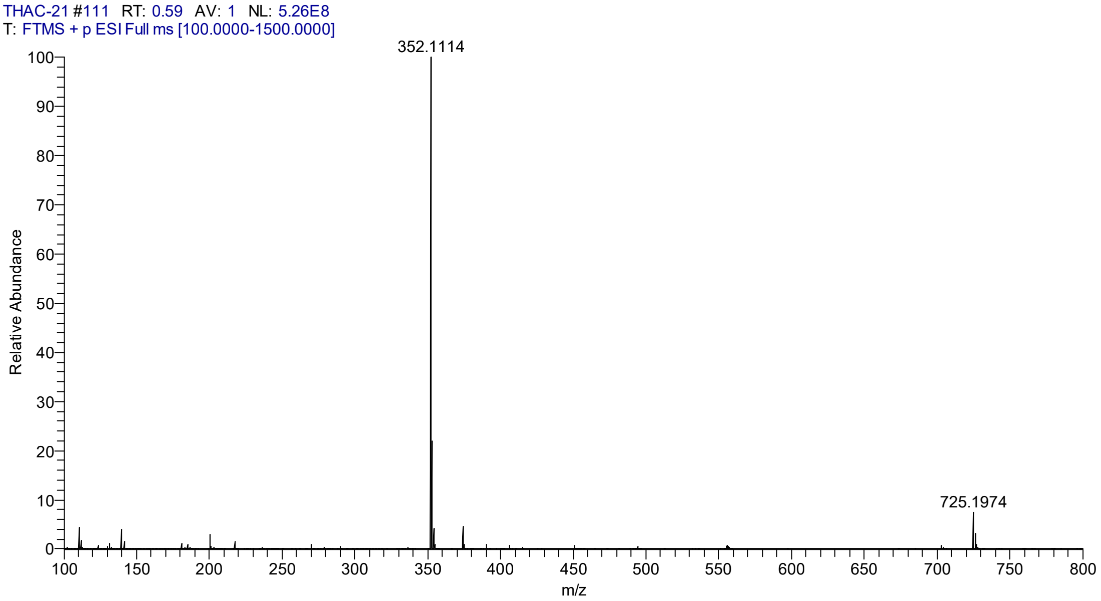

The HRMS spectrogram of compound **E27**

Compound **E28**, 2-(4-methoxyphenyl)-*N′*-(4-methylbenzylidene)thiazole-4-carbohydrazide:

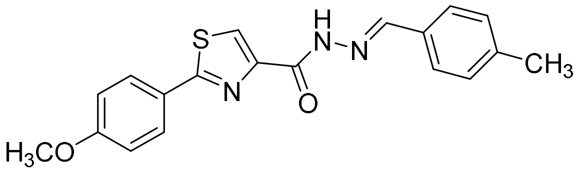

white solid, yield 68%, m.p. 163.1-164.2 °C; ^{1}H NMR (400 MHz, DMSO-d_6) δ 11.68 (s, 1H), 8.59 (s, 1H), 8.39 (s, 1H), 8.06 (d, J = 8.8 Hz, 2H), 7.64 (d, J = 7.6 Hz, 2H), 7.29 (d, J = 7.6 Hz, 2H), 7.10 (d, J = 8.8 Hz, 2H), 3.85 (s, 3H), 2.36 (s, 3H). ^{13}C NMR (101 MHz, DMSO-d_6) δ 167.89, 161.84, 157.34, 149.69, 149.29, 140.50, 132.10, 129.96, 128.78, 127.64, 125.69, 124.96, 115.05, 55.96, 21.53. HRMS(ESI) calcd for $C_{19}H_{17}N_3O_2S$ [M+H]$^+$: 352.1114, found 352.1111.

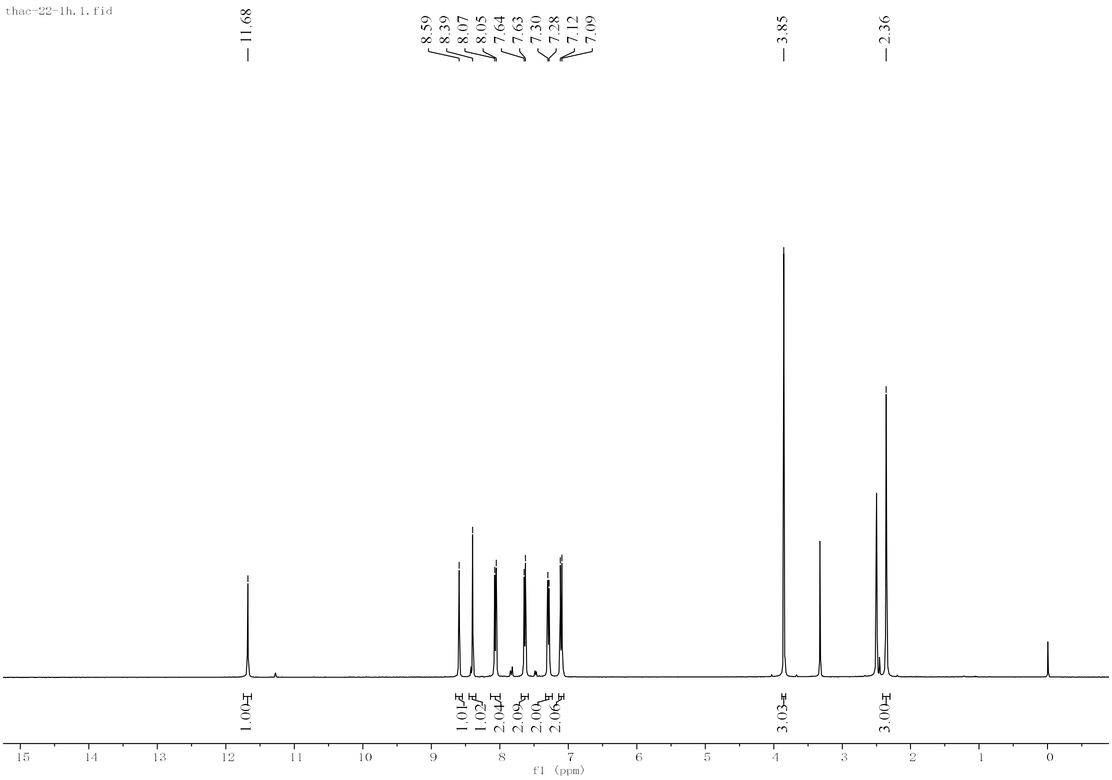

The ^{1}H NMR spectrogram of compound **E28**

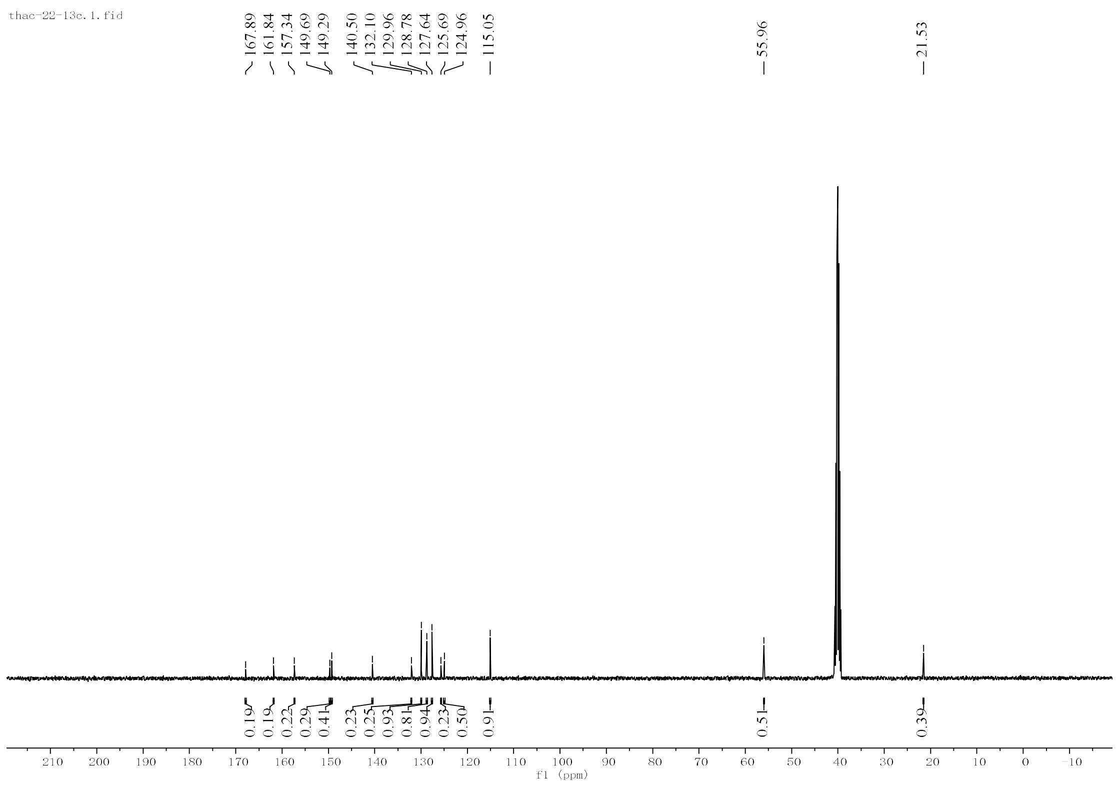

The ^{13}C NMR spectrogram of compound **E28**

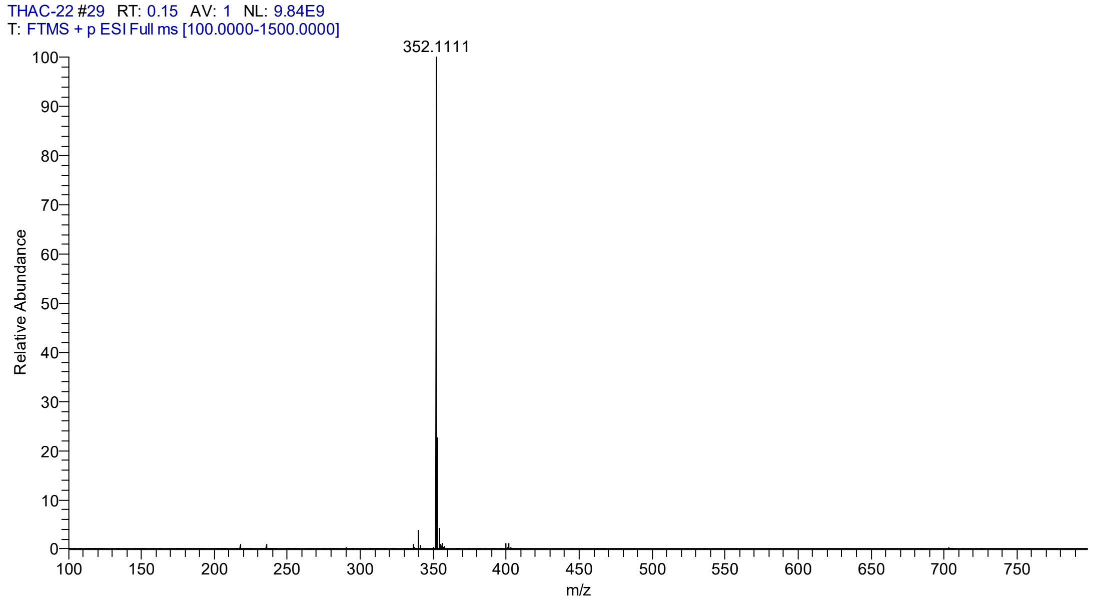

The HRMS spectrogram of compound **E28**

Compound **E29**, *N'*-(4-methylbenzylidene)-2-(2-(trifluoromethoxy)phenyl)thiazole-4-carbohydrazide:

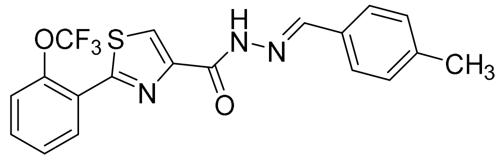

white solid, yield 73 %, m.p. 161.7-163.2 °C; ^{1}H NMR (400 MHz, DMSO-d_6) δ 11.76 (s, 1H), 8.70 – 8.60 (m, 3H), 7.69 – 7.51 (m, 5H), 7.24 (d, J = 8.0 Hz, 2H), 2.31 (s, 3H). ^{13}C NMR (101 MHz, DMSO-d_6) δ 160.03, 156.66, 148.98, 148.45, 145.34, 140.05, 132.24, 131.52, 130.17, 129.44, 127.85, 127.15, 125.07, 120.88, 120.05 (q, J = 259.2 Hz), 21.00. HRMS(ESI) calcd for $C_{19}H_{14}F_3N_3O_2S$ [M+H]$^+$: 406.0832, found 406.0832.

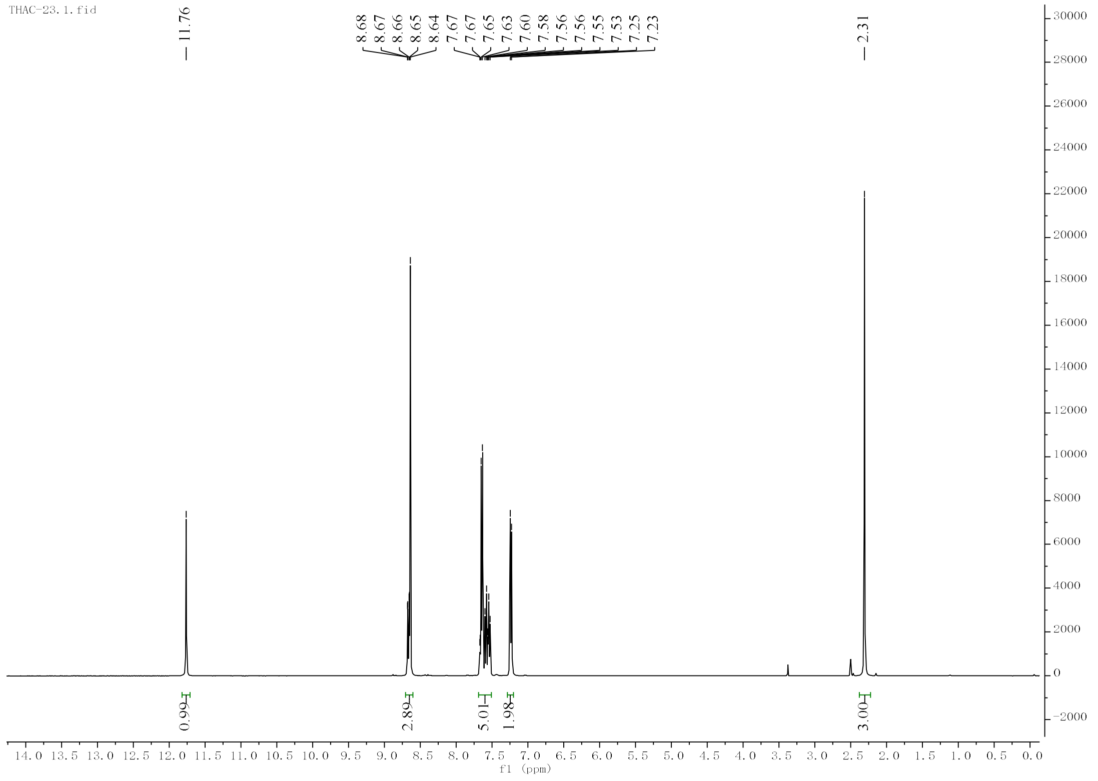

The ^{1}H NMR spectrogram of compound **E29**

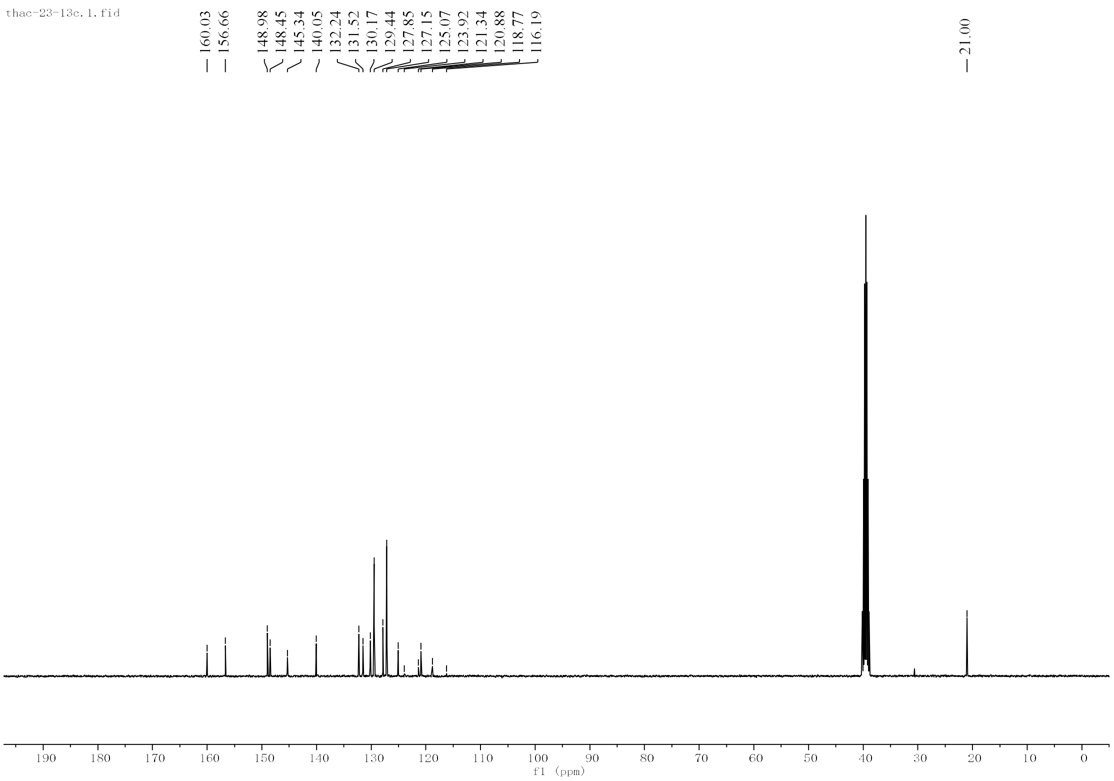

The ^{13}C NMR spectrogram of compound **E29**

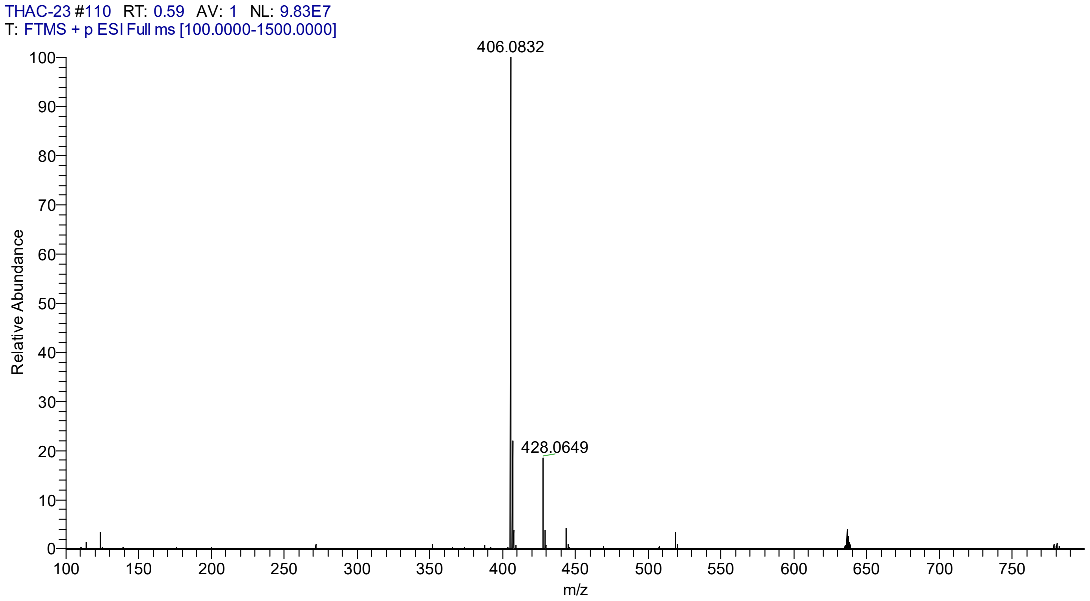

The HRMS spectrogram of compound **E29**

Compound **E30**, *N'*-(4-methylbenzylidene)-2-(3-(trifluoromethoxy)phenyl)thiazole-4-carbohydrazide:

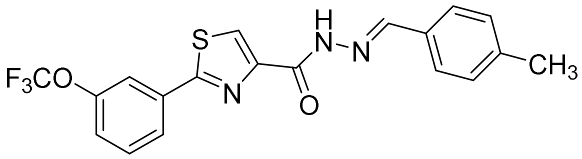

white yield 67%, m.p. 156.5-157.9 °C; ^{1}H NMR (400 MHz, DMSO-d_6) δ 11.76 (s, 1H), 8.61 (s, 1H), 8.57 (s, 1H), 8.18 (s, 1H), 8.11 (d, J = 8.0 Hz, 1H), 7.70 (t, J = 8.0 Hz, 1H), 7.65 (d, J = 8.0 Hz, 2H), 7.56 (d, J = 8.4 Hz, 1H), 7.29 (d, J = 8.0 Hz, 2H), 2.35 (s, 3H). ^{13}C NMR (101 MHz, DMSO-d_6) δ 165.45, 156.62, 149.48, 149.02, 148.93 (d, J = 1.9 Hz), 140.06, 134.48, 131.52, 131.47, 129.46, 127.16, 126.49, 125.91, 122.98, 120.06 (q, J = 257.2 Hz), 118.90, 21.02. HRMS(ESI) calcd for $C_{19}H_{14}F_3N_3O_2S$ [M+H]$^+$: 406.0832, found 406.0830.

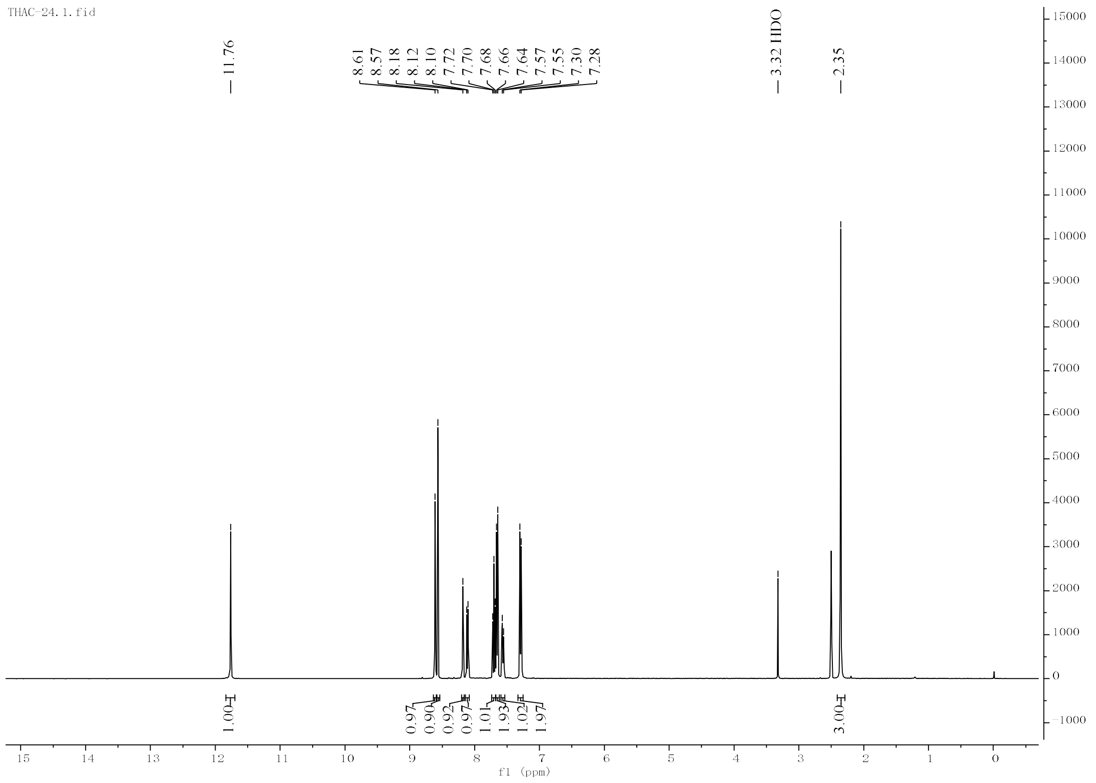

The ^{1}H NMR spectrogram of compound **E30**

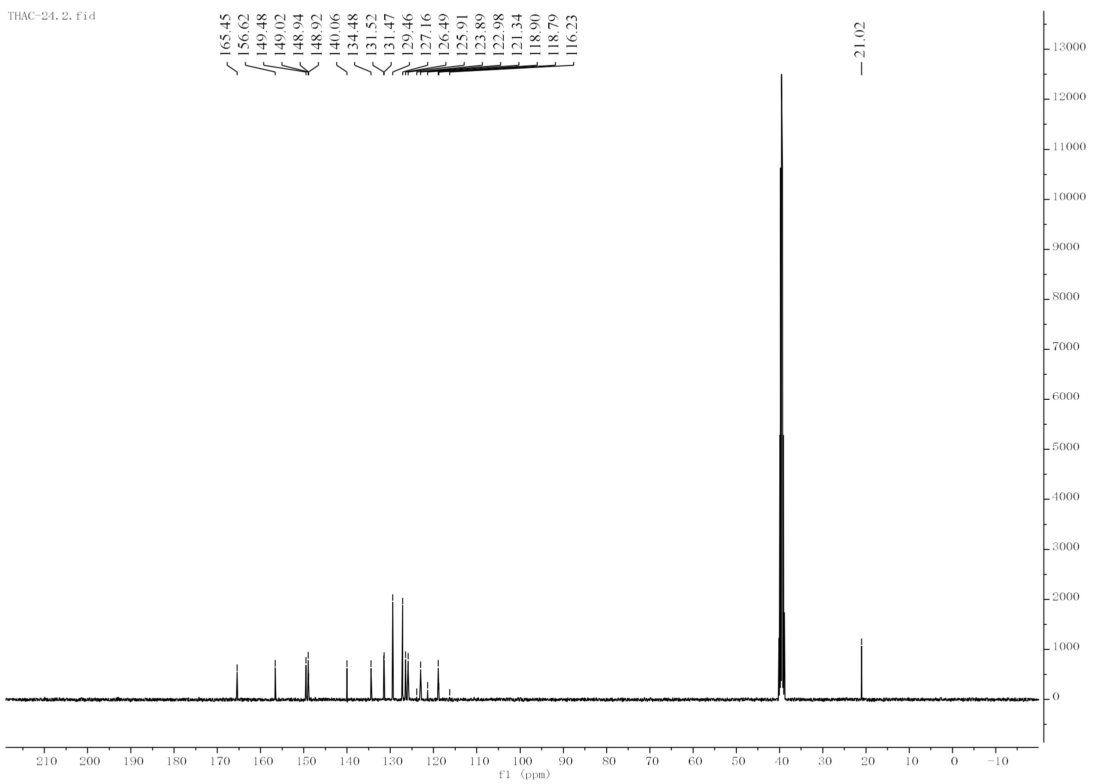

The ^{13}C NMR spectrogram of compound **E30**

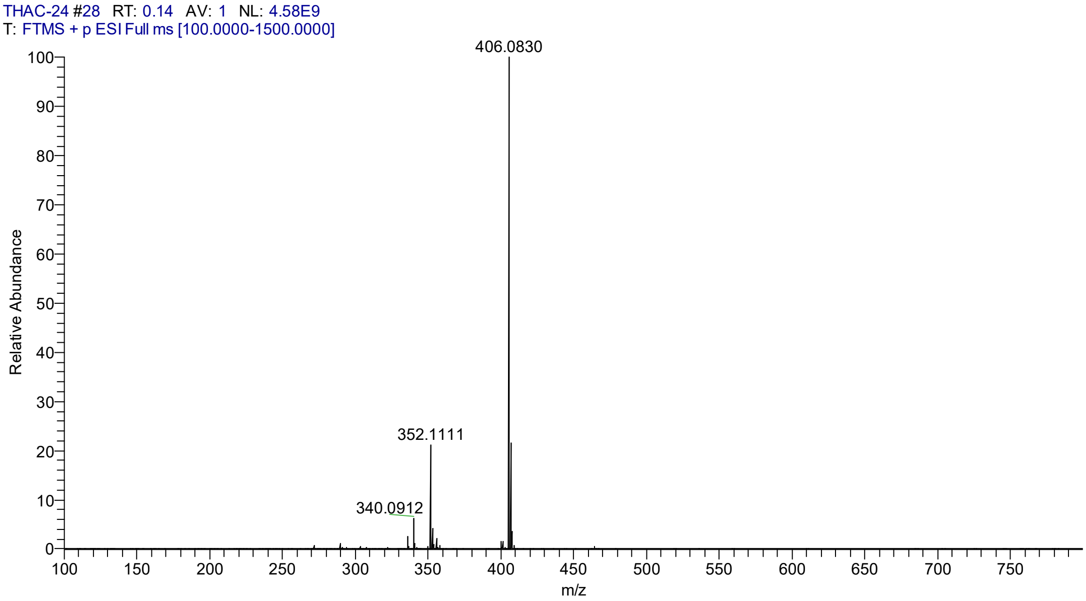

The HRMS spectrogram of compound **E30**

Compound **E31**, *N'*-(4-methylbenzylidene)-2-(4-(trifluoromethoxy)phenyl)thiazole-4-carbohydrazide:

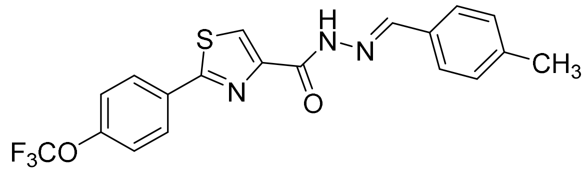

white solid, yield 62%, m.p. 172.8-173.9 °C; ^{1}H NMR (400 MHz, DMSO-d_6) δ 11.75 (s, 1H), 8.60 (s, 1H), 8.53 (s, 1H), 8.29 – 8.21 (m, 2H), 7.64 (d, J = 8.0 Hz, 2H), 7.56 (d, J = 8.4 Hz, 2H), 7.28 (d, J = 8.0 Hz, 2H), 2.35 (s, 3H). ^{13}C NMR (101 MHz, DMSO-d_6) δ 165.76, 156.69, 149.82 (d, J = 1.6 Hz), 149.54, 148.92, 140.04, 131.53, 129.44, 128.69, 127.15, 126.16, 121.62, 119.97 (q, J = 257.1 Hz), 21.01. HRMS(ESI) calcd for $C_{19}H_{14}F_3N_3O_2S$ $[M+H]^+$: 406.0832, found 406.0832.

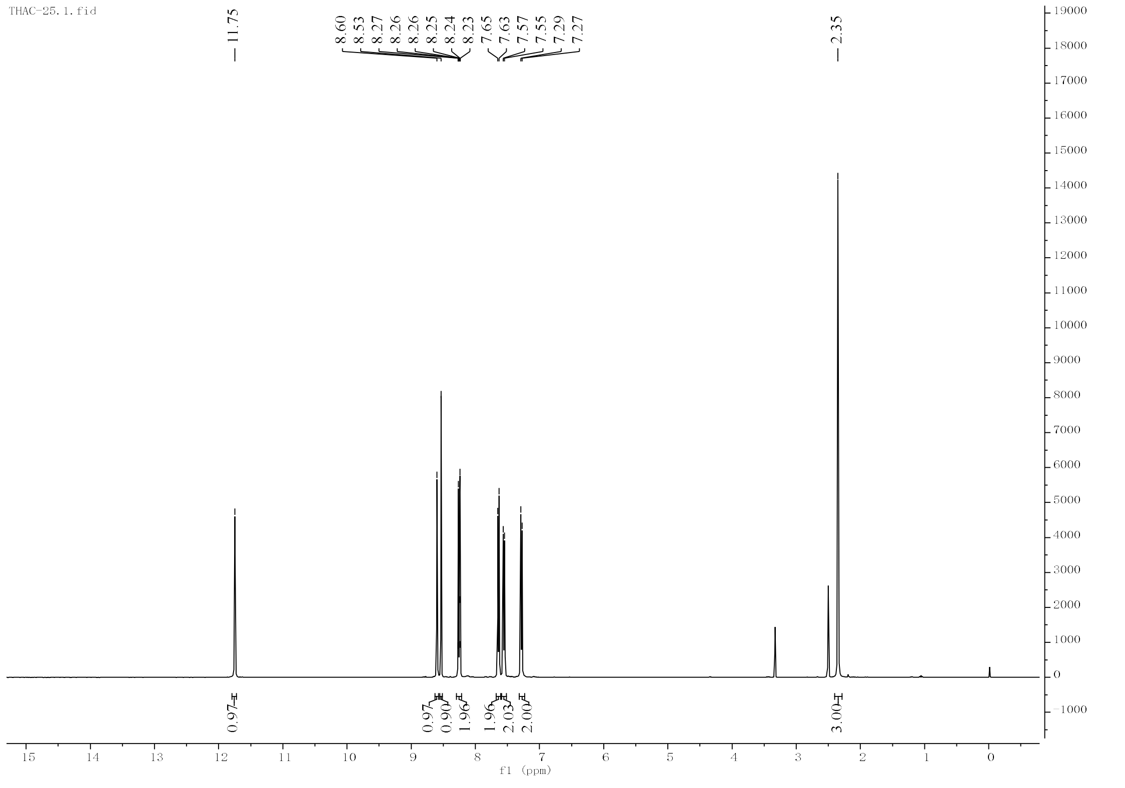

The ^{1}H NMR spectrogram of compound **E31**

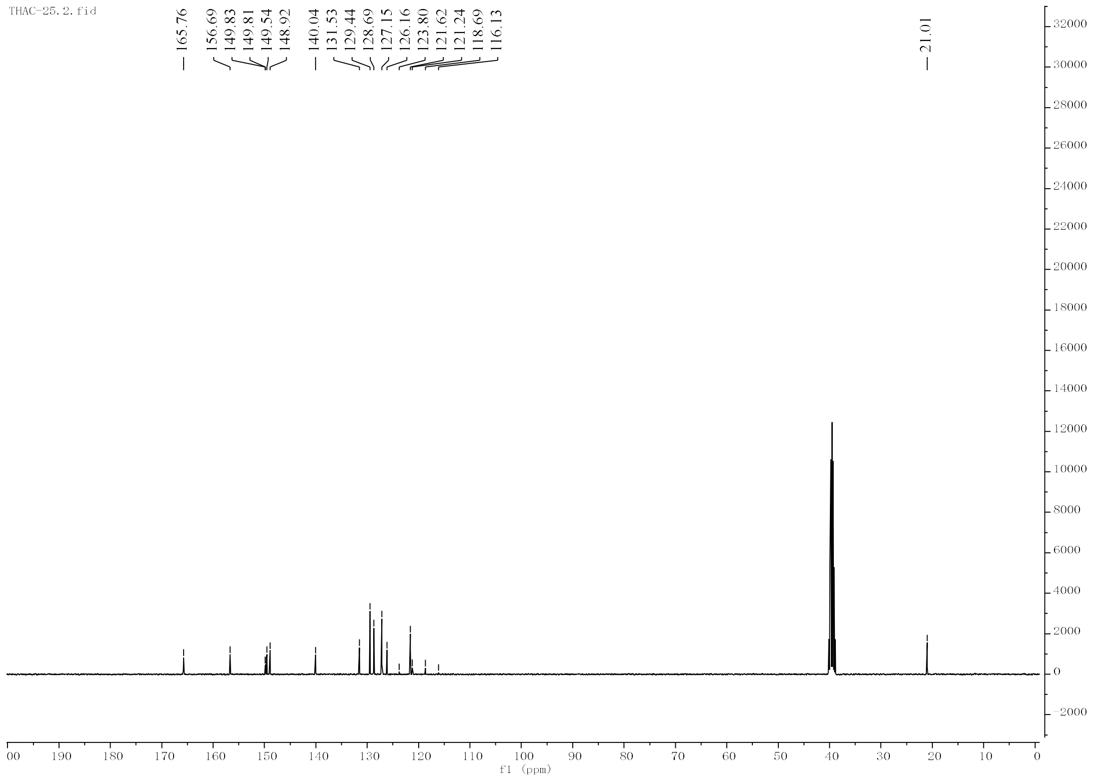

The ^{13}C NMR spectrogram of compound **E31**

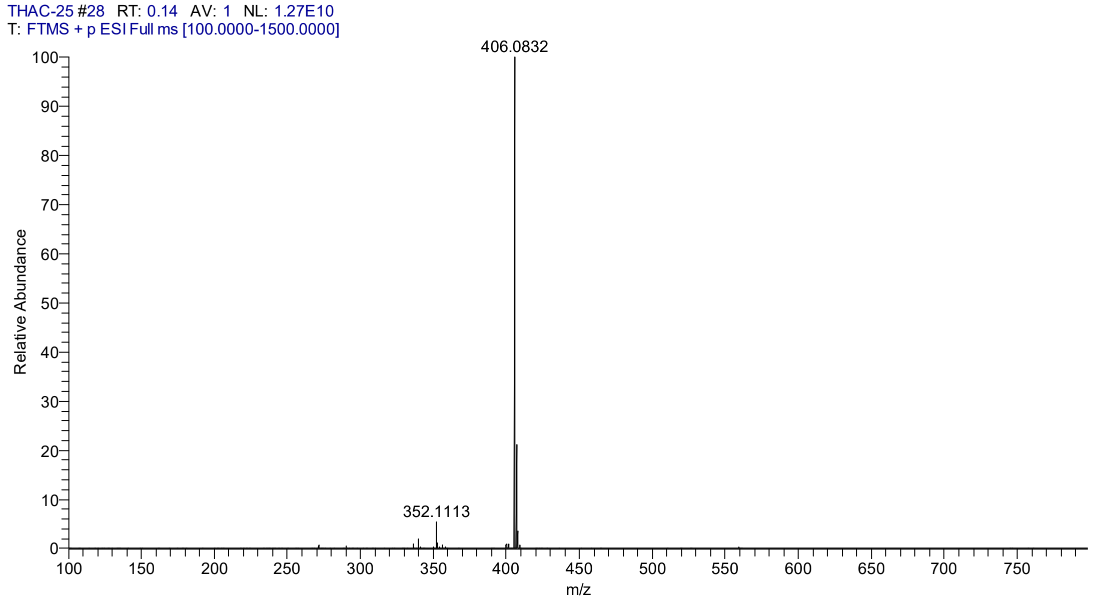

The HRMS spectrogram of compound **E31**

Compound **E32**, *N'*-(4-methylbenzylidene)-2-(2-(trifluoromethyl)phenyl)thiazole-4-carbohydrazide:

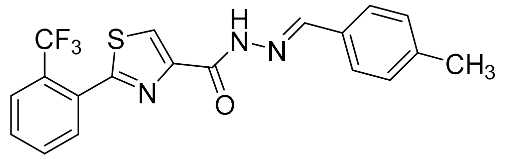

white solid, yield 74%, m.p. 154.0-155.2 °C; ^{1}H NMR (400 MHz, DMSO-d_6) δ 11.63 (s, 1H), 8.67 (s, 1H), 8.53 (s, 1H), 7.96 (d, J = 7.6 Hz, 1H), 7.85 (d, J = 4.4 Hz, 2H), 7.81 (dd, J = 8.0, 4.4 Hz, 1H), 7.61 (d, J = 8.0 Hz, 2H), 7.26 (d, J = 8.0 Hz, 2H), 2.34 (s, 3H). ^{13}C NMR (101 MHz, DMSO-d_6) δ 163.83, 156.68, 148.95, 148.91, 139.95, 132.65, 132.60, 131.54, 131.20, 130.85, 129.37, 127.70, 127.35 (q, J = 30.5 Hz), 127.12, 126.66 (q, J = 5.2 Hz), 122.22 (q, J = 274.0 Hz) 20.97. HRMS(ESI) calcd for $C_{19}H_{14}F_3N_3OS$ $[M+H]^+$: 390.0882, found 390.0882.

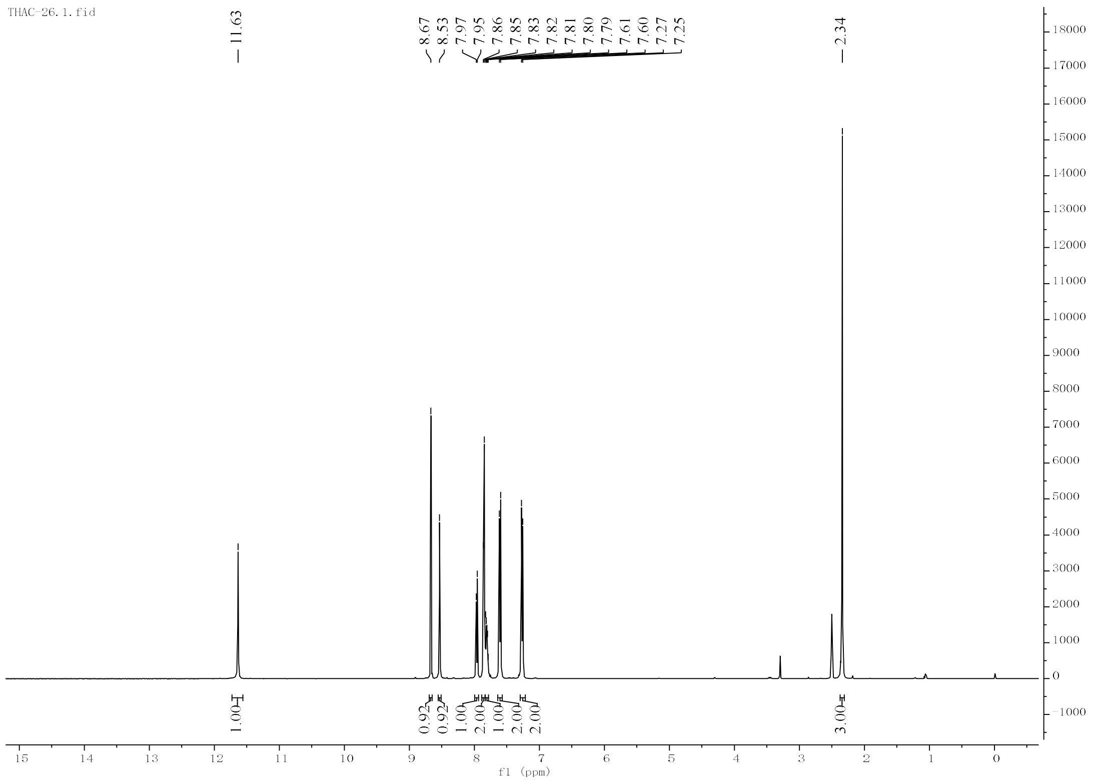

The ^{1}H NMR spectrogram of compound **E32**

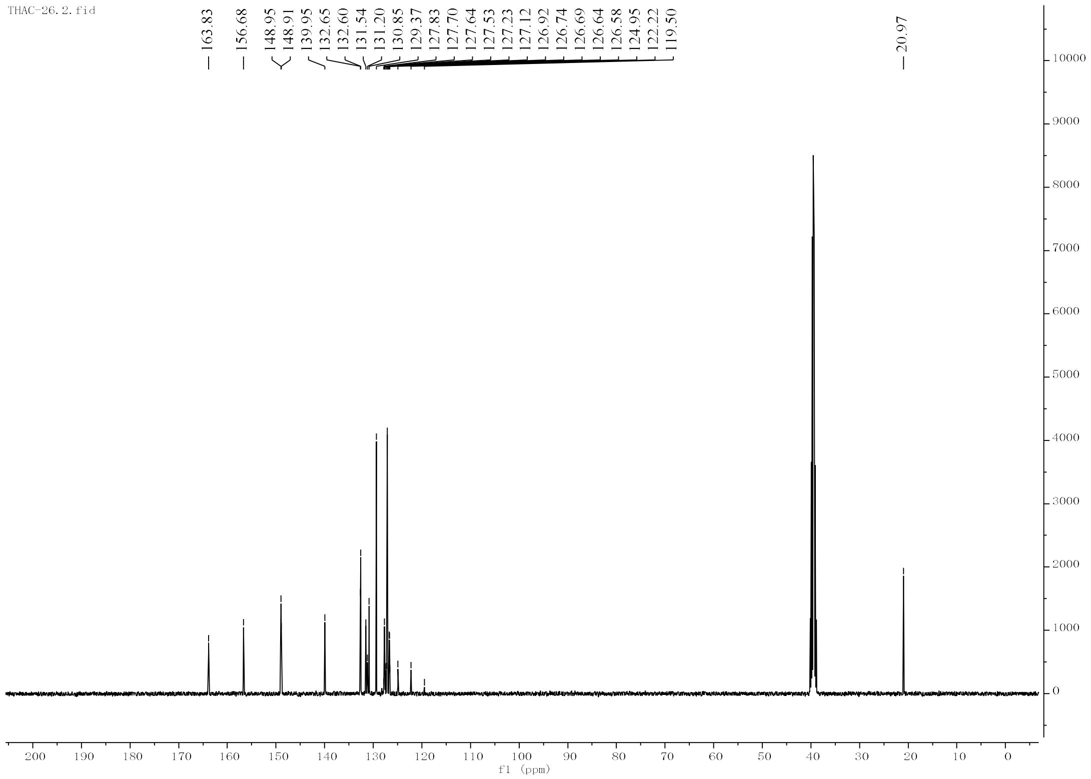

The ^{13}C NMR spectrogram of compound **E32**

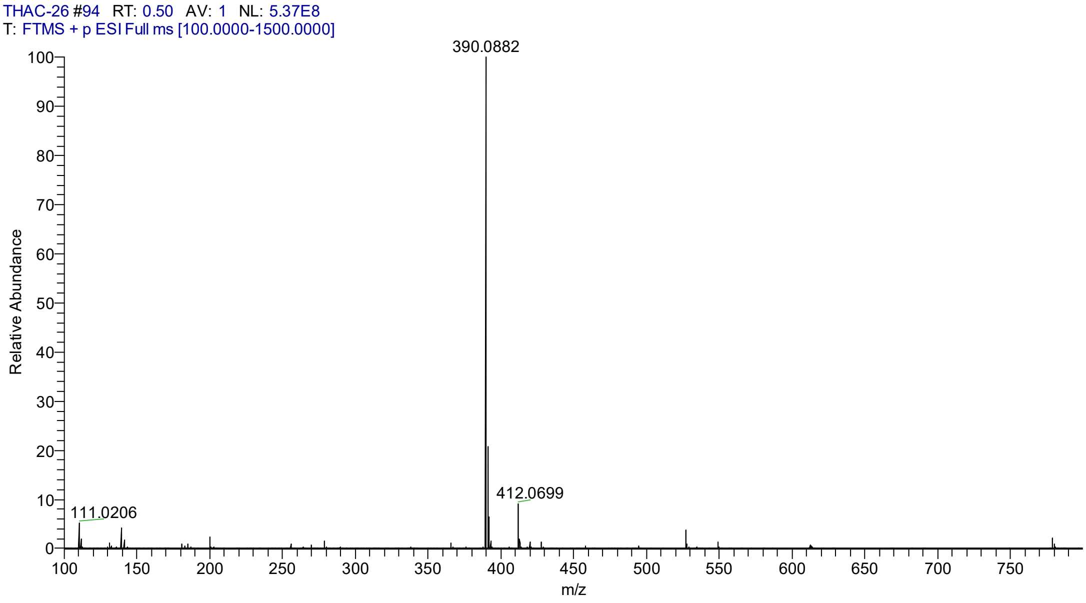

The HRMS spectrogram of compound **E32**

Compound **E33**, *N'*-(4-methylbenzylidene)-2-(3-(trifluoromethyl)phenyl)thiazole-4-carbohydrazide:

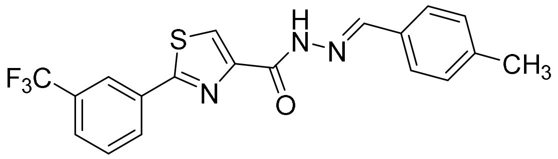

white solid, yield 66%, m.p. 174.5-176.4 °C; ^{1}H NMR (400 MHz, DMSO-d_6) δ 11.78 (s, 1H), 8.63 (s, 1H), 8.57 (s, 1H), 8.47 (s, 1H), 8.33 (d, J = 7.6 Hz, 1H), 7.87 (d, J = 8.0 Hz, 1H), 7.77 (t, J = 8.0 Hz, 1H), 7.65 (d, J = 7.6 Hz, 2H), 7.25 (d, J = 7.6 Hz, 2H), 2.32 (s, 3H). ^{13}C NMR (101 MHz, DMSO-d_6) δ 165.54, 156.63, 149.70, 149.66, 149.03, 139.91, 133.25, 131.52, 130.59, 130.34, 130.14 (q, J = 32.5 Hz), 129.30, 127.08, 126.30, 124.82 (dd, J = 428.1, 4.0 Hz), 123.79 (q, J = 272.6 Hz), 20.88. HRMS(ESI) calcd for $C_{19}H_{14}F_3N_3OS$ [M+H]$^+$: 390.0882, found 390.0882.

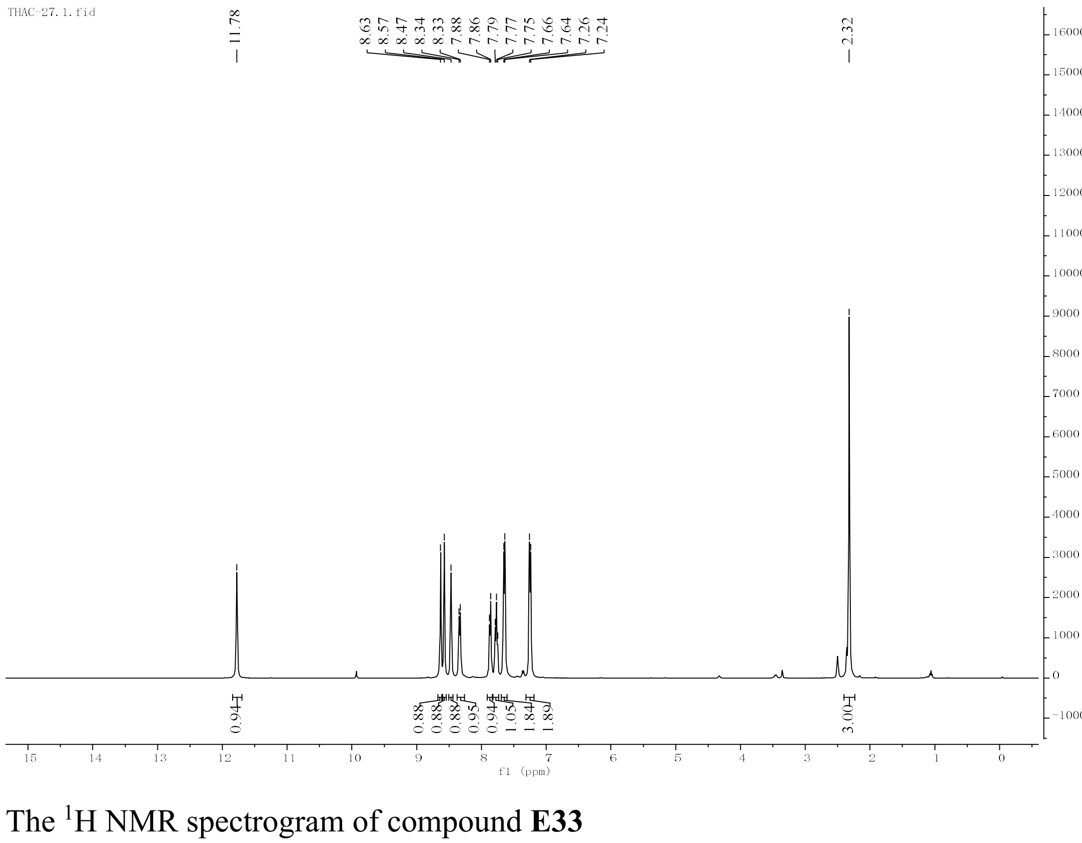

The ^{1}H NMR spectrogram of compound **E33**

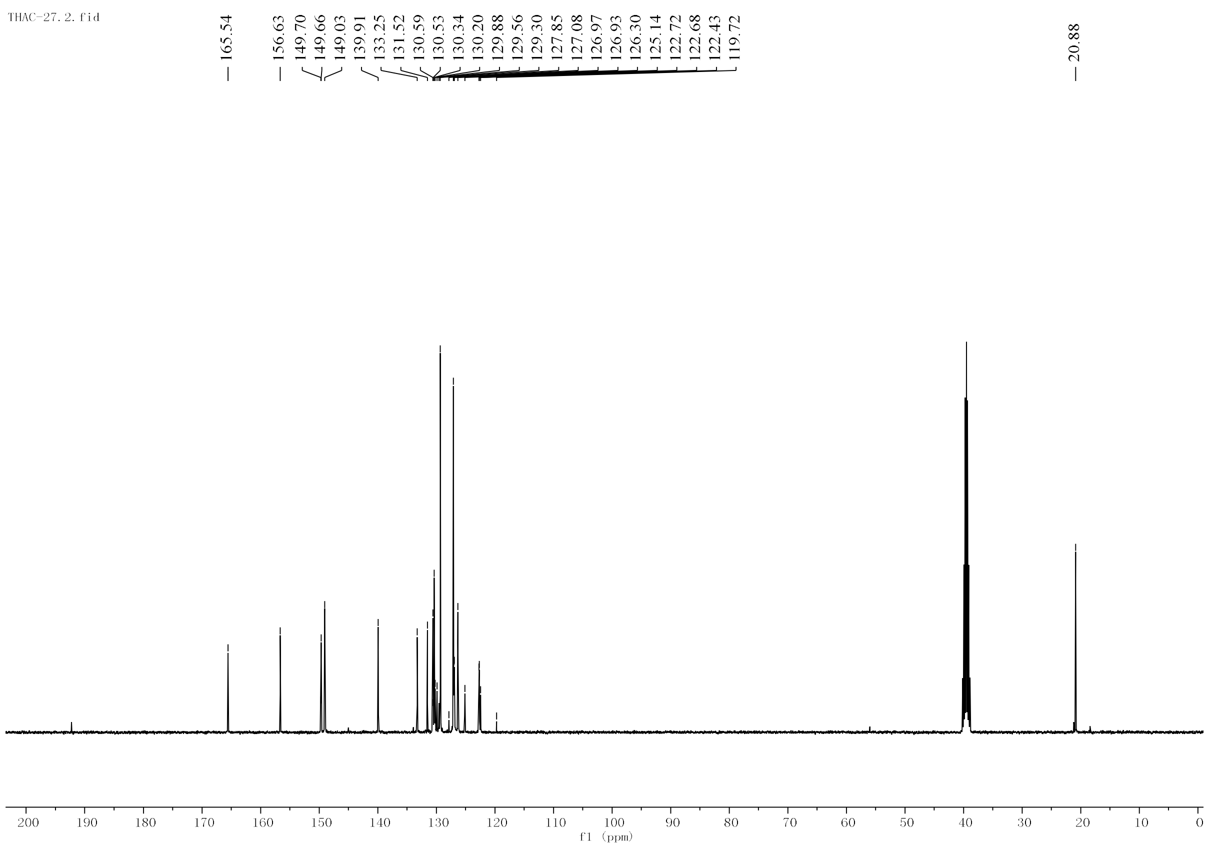

The ^{13}C NMR spectrogram of compound **E33**

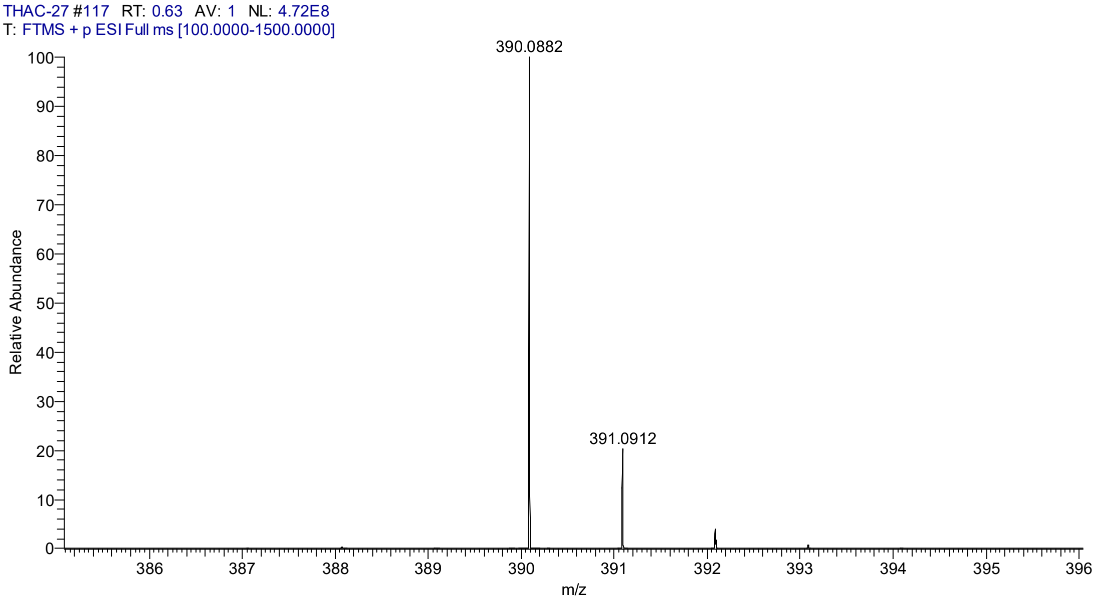

The HRMS spectrogram of compound **E33**

Compound **E34**, *N′*-(4-methylbenzylidene)-2-(4-(trifluoromethyl)phenyl)thiazole-4-carbohydrazide:

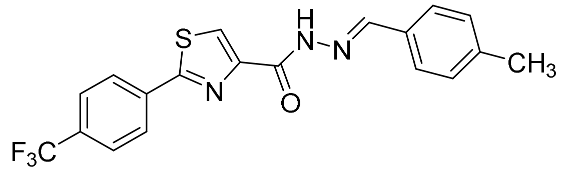

white solid, yield 64%, m.p. 218.6-220.1 °C; ^{1}H NMR (400 MHz, DMSO-d_6) δ 11.83 (s, 1H), 8.61 (d, J = 5.6 Hz, 2H), 8.33 (d, J = 8.0 Hz, 2H), 7.90 (d, J = 8.4 Hz, 2H), 7.64 (d, J = 8.0 Hz, 2H), 7.26 (d, J = 8.0 Hz, 2H), 2.33 (s, 3H). ^{13}C NMR (101 MHz, DMSO-d_6) δ 165.62, 156.72, 149.80, 149.04, 140.11, 135.92, 131.58, 130.52 (q, J = 32.0 Hz), 129.49, 127.34, 127.23, 126.91, 126.20 (q, J = 3.8 Hz), 123.97 (q, J = 272.1 Hz), 21.07. HRMS(ESI) calcd for $C_{19}H_{14}F_3N_3OS$ $[M+H]^+$: 390.0882, found 390.0880.

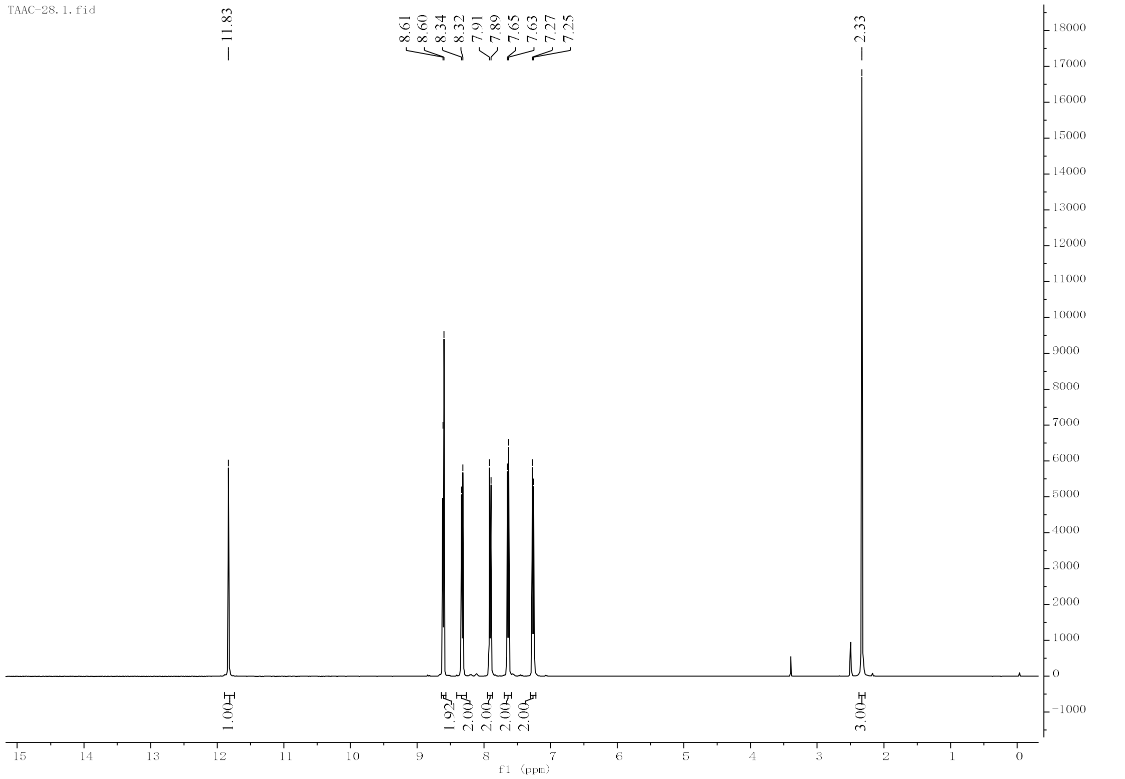

The ^{1}H NMR spectrogram of compound **E34**

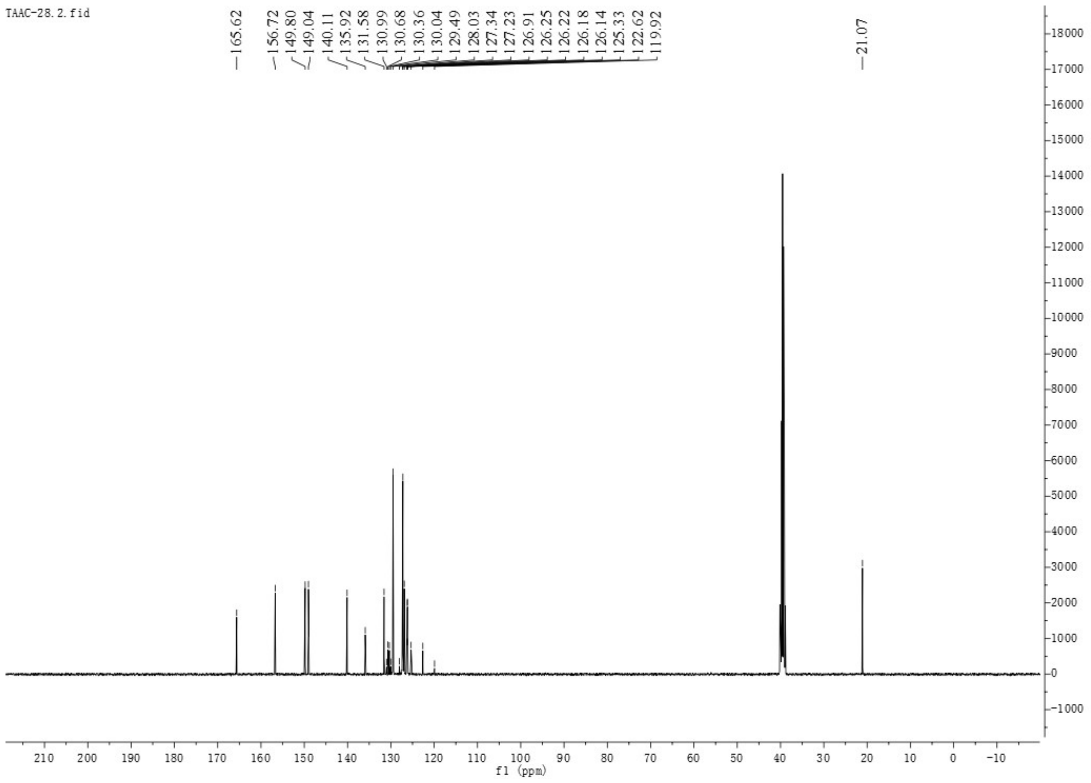

The ^{13}C NMR spectrogram of compound **E34**

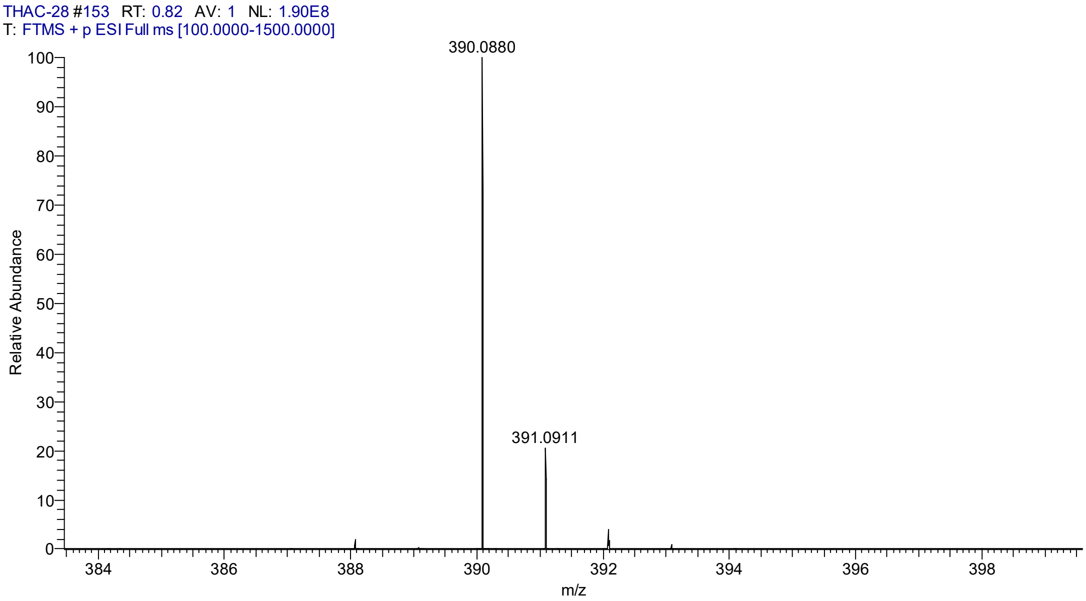

The HRMS spectrogram of compound **E34**

Compound **E35**, 2-(2-aminophenyl)-*N′*-(4-methylbenzylidene)thiazole-4-carbohydrazide:

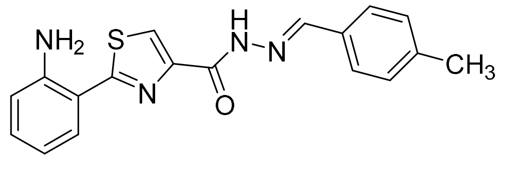

yellow solid, yield 61%, m.p. 213.3-215.6 °C; ^{1}H NMR (400 MHz, $CDCl_3$) δ 10.05 (s, 1H), 8.32 (s, 1H), 8.20 (s, 1H), 7.68 (d, J = 8.0 Hz, 2H), 7.61 (d, J = 8.0 Hz, 1H), 7.22 (t, J = 7.6 Hz, 3H), 6.79 (dd, J = 18.0, 8.0 Hz, 2H), 5.74 (s, 2H), 2.38 (s, 3H). ^{13}C NMR (101 MHz, $CDCl_3$) δ 169.98, 157.14, 149.34, 148.62, 145.17, 141.12, 131.65, 130.82, 129.69, 129.48, 127.85, 123.37, 117.82, 117.14, 115.37, 21.56. HRMS(ESI) calcd for $C_{18}H_{16}N_4OS$ $[M+H]^+$: 337.1118, found 337.1117.

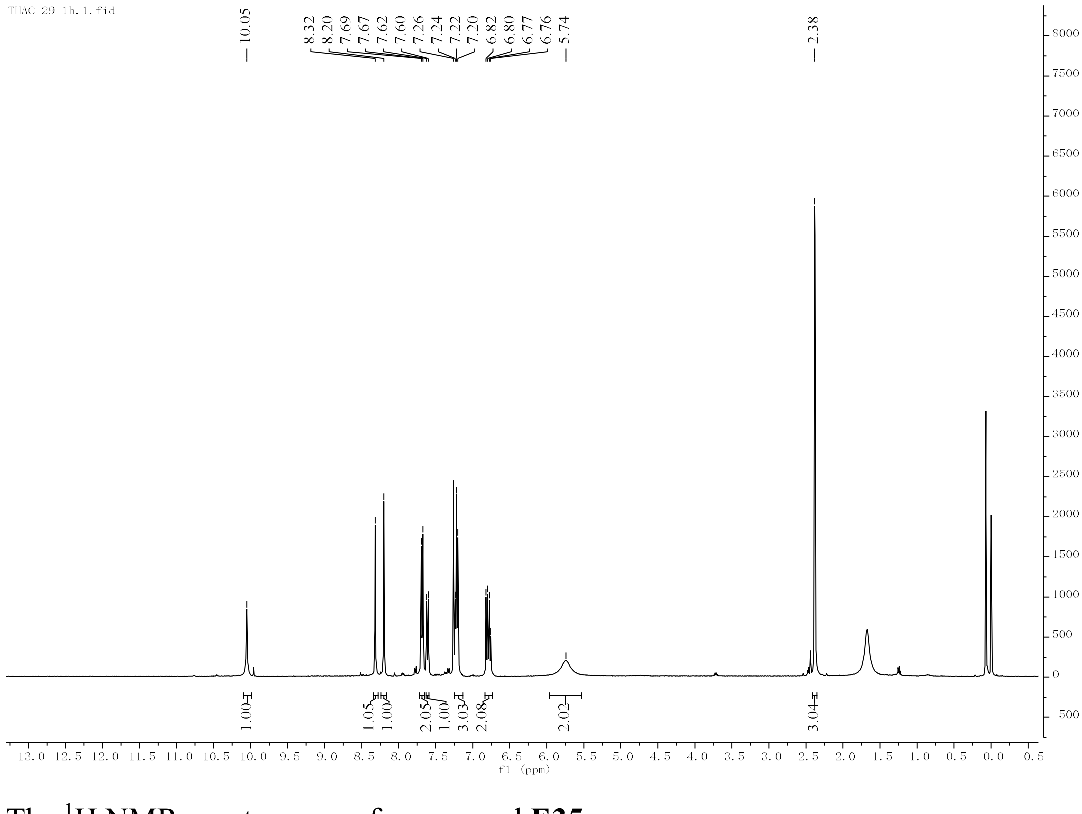

The ^{1}H NMR spectrogram of compound **E35**

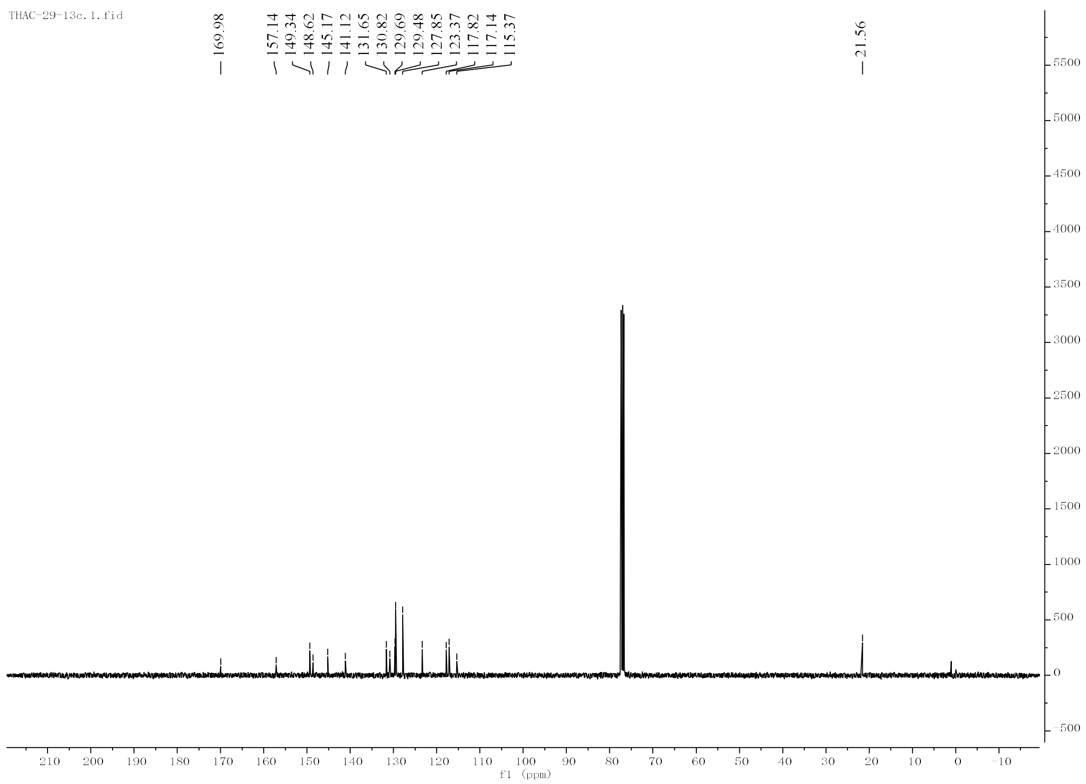

The ^{13}C NMR spectrogram of copound **E35**

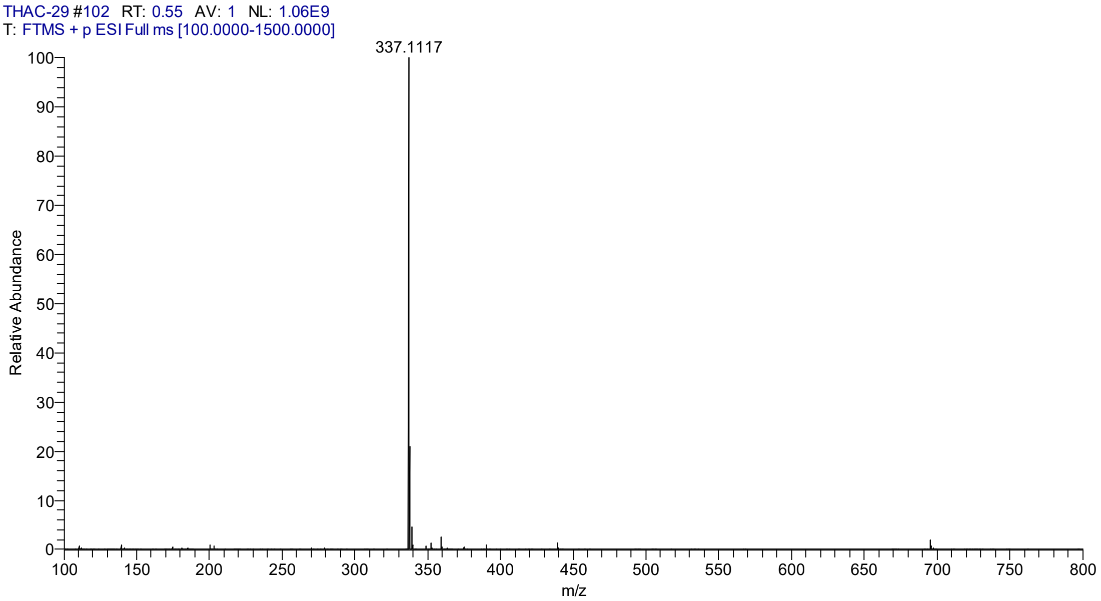

The HRMS spectrogram of compound **E35**

Compound **E36**,

2-(4-aminophenyl)-*N'*-(4-methylbenzylidene)thiazole-4-carbohydrazide:

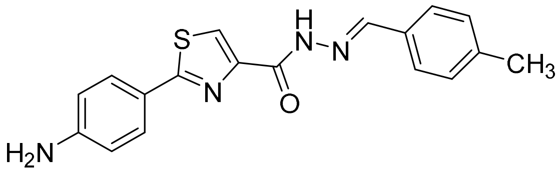

yellow solid, yield 65%, m.p. 215.6-217.2 °C; ^{1}H NMR (400 MHz, DMSO-d_6) δ 11.53 (s, 1H), 8.58 (s, 1H), 8.22 (s, 1H), 7.78 (d, J = 8.4 Hz, 2H), 7.63 (d, J = 8.0 Hz, 2H), 7.29 (d, J = 8.0 Hz, 2H), 6.67 (d, J = 8.8 Hz, 2H), 5.74 (s, 2H), 2.36 (s, 3H). ^{13}C NMR (101 MHz, DMSO-d_6) δ 169.21, 157.47, 152.09, 149.27, 149.08, 140.45, 132.13, 129.96, 128.56, 127.61, 123.10, 120.46, 113.95, 21.53. HRMS(ESI) calcd for $C_{18}H_{16}N_4OS$ [M+H]$^+$: 337.1118, found 337.1118.

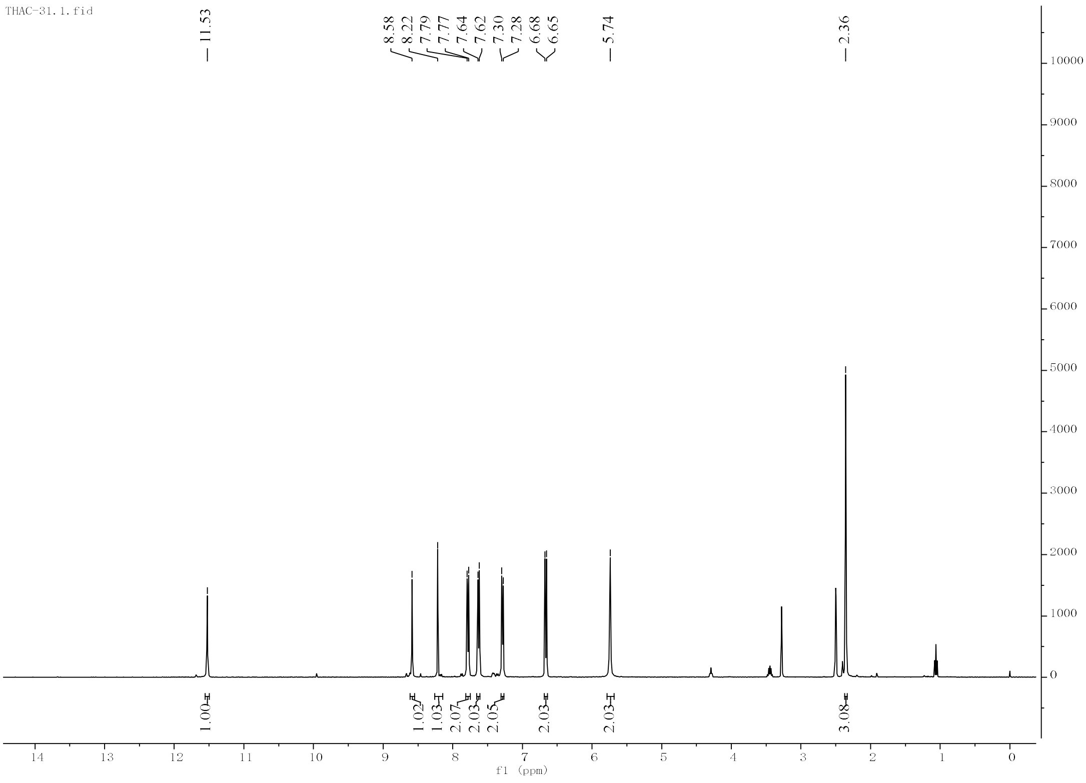

The ^{1}H NMR spectrogram of compound **E36**

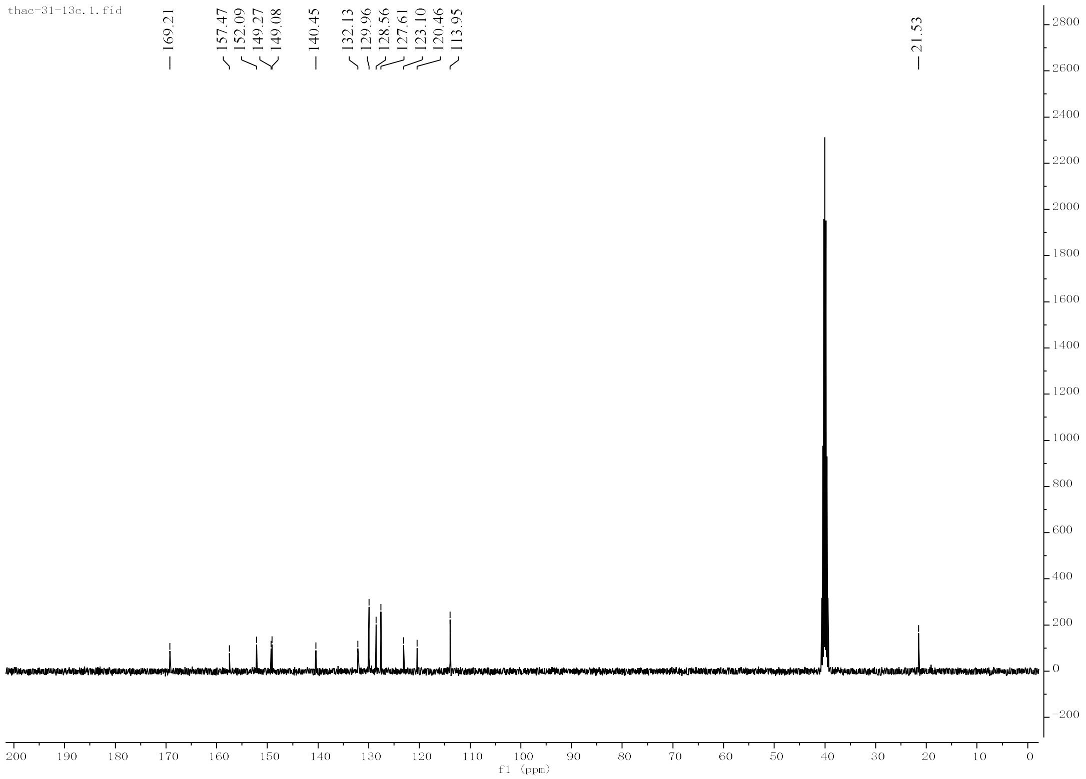

The ^{13}C NMR spectrogram of compound **E36**

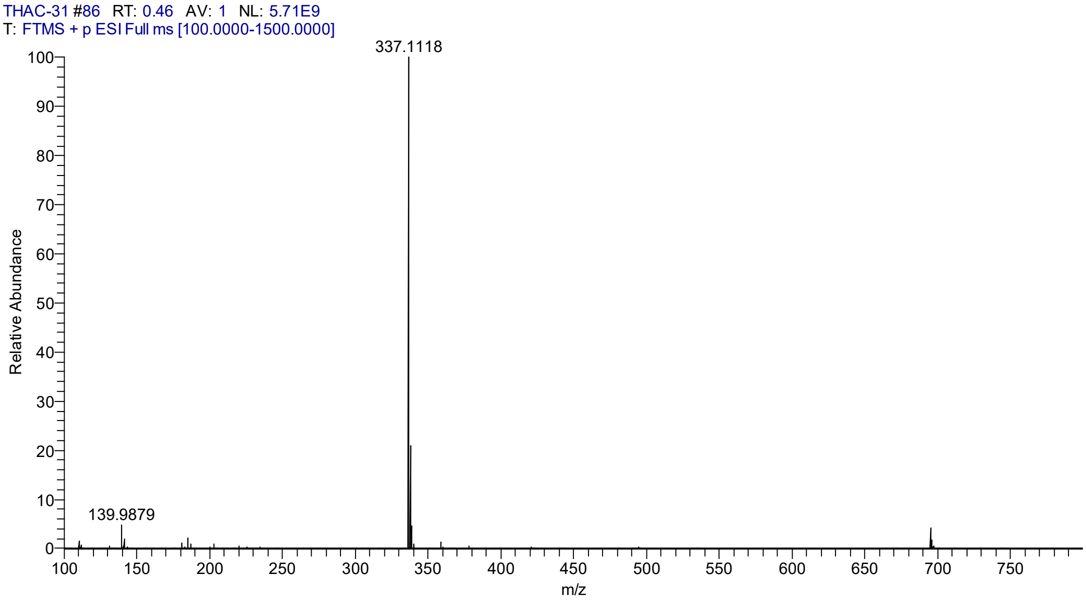

The HRMS spectrogram of compound **E36**

Compound **E37**,

2-(2-hydroxyphenyl)-*N'*-(4-methylbenzylidene)thiazole-4-carbohydrazide:

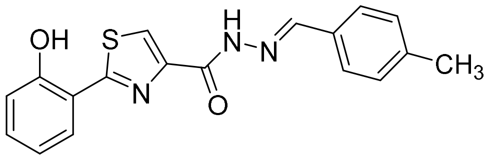

white solid, yield 72%, m.p. 220.2-221.6 °C; ^{1}H NMR (400 MHz, DMSO-d_6) δ 11.72 (s, 1H), 11.16 (s, 1H), 8.62 (s, 1H), 8.54 – 8.39 (m, 2H), 7.64 (d, J = 7.6 Hz, 2H), 7.36 (t, J = 7.0 Hz, 1H), 7.30 (d, J = 8.0 Hz, 2H), 7.11 – 6.97 (m, 2H), 2.36 (s, 3H). ^{13}C NMR (101 MHz, DMSO-d_6) δ 163.44, 157.57, 155.56, 149.18, 147.99, 140.47, 132.14, 131.98, 129.97, 128.74, 127.62, 125.96, 119.87, 119.57, 116.88, 21.54. HRMS(ESI) calcd for $C_{18}H_{15}N_3O_2S$ $[M+H]^+$: 338.0958, found 338.0956.

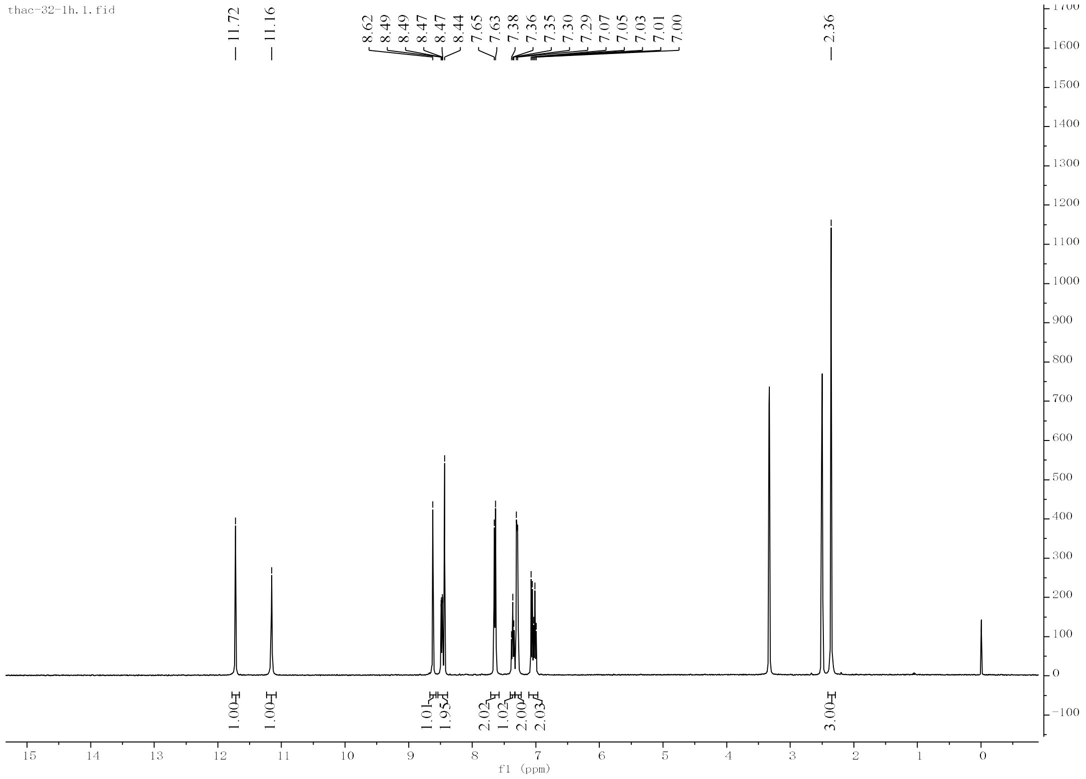

The ^{1}H NMR spectrogram of compound **E37**

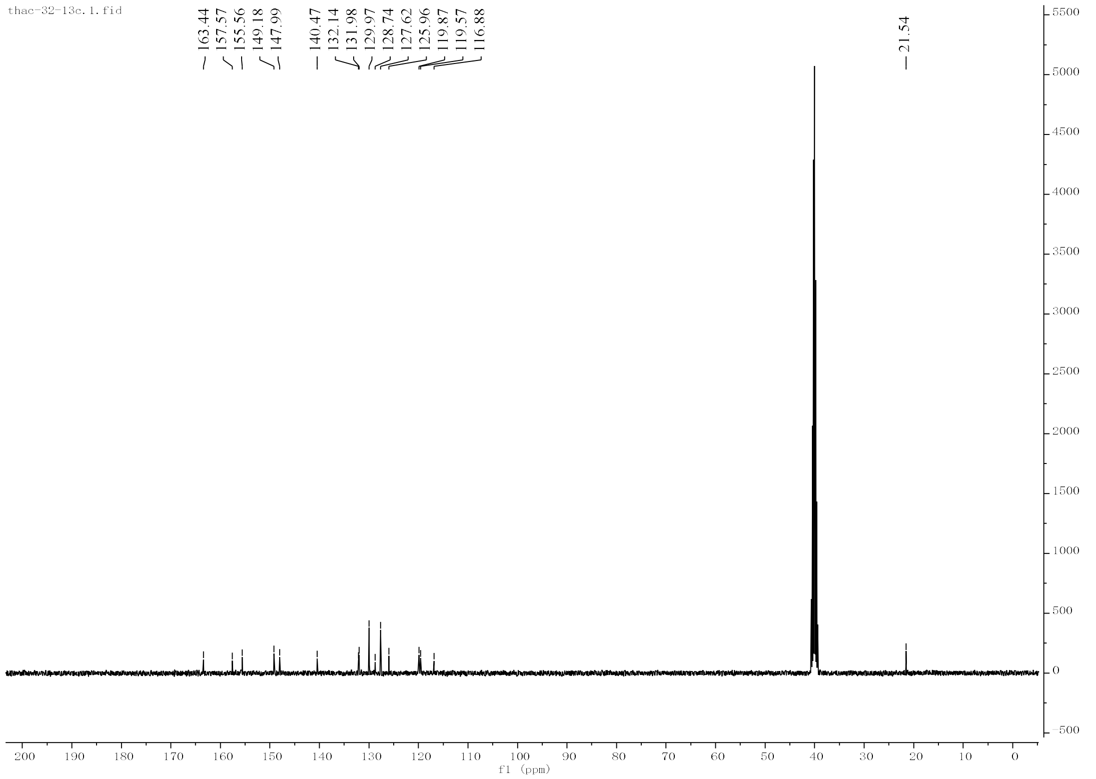

The ^{13}C NMR spectrogram of compound **E37**

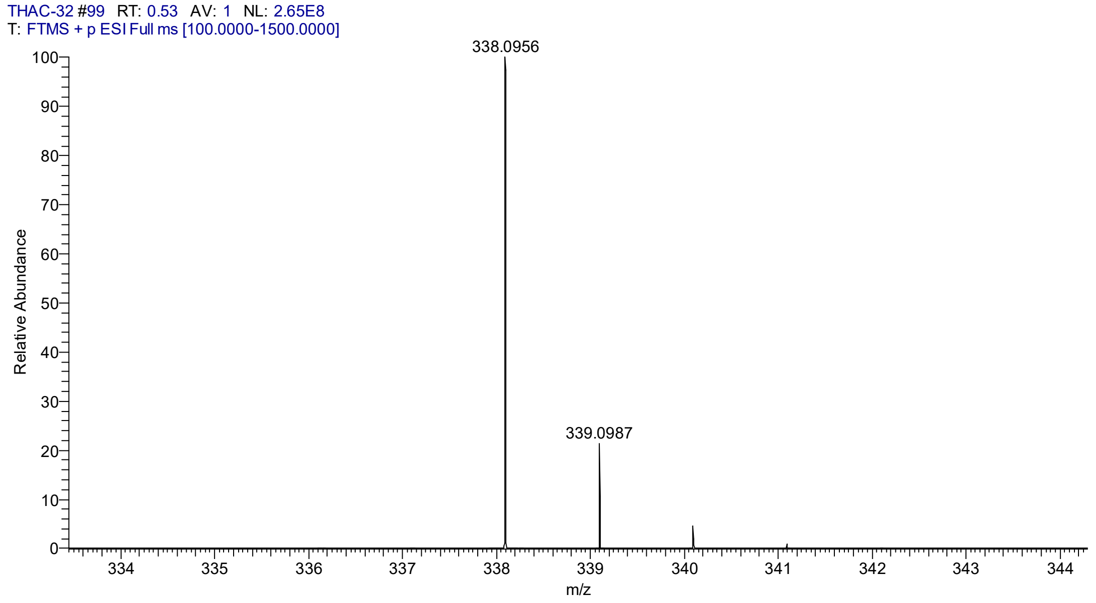

The HRMS spectrogram of compound **E37**

Compound **E38**,

2-(3-hydroxyphenyl)-*N'*-(4-methylbenzylidene)thiazole-4-carbohydrazide:

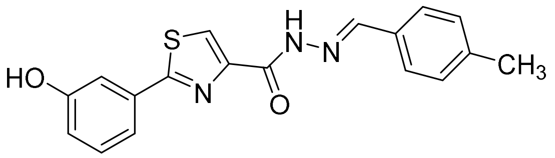

white solid, yield 68%, m.p. >220 °C; ^{1}H NMR (400 MHz, DMSO-d_6) δ 11.72 (s, 1H), 9.84 (s, 1H), 8.59 (s, 1H), 8.46 (s, 1H), 7.64 (d, J = 8.0 Hz, 2H), 7.58 – 7.43 (m, 2H), 7.32 (dt, J = 20.0, 8.0 Hz, 3H), 7.01 – 6.87 (m, 1H), 2.35 (s, 3H). ^{13}C NMR (101 MHz, DMSO-d_6) δ 168.08, 158.45, 157.34, 149.87, 149.30, 140.52, 134.07, 132.08, 130.82, 129.96, 127.65, 125.82, 118.41, 117.96, 113.65, 21.53 HRMS(ESI) calcd for $C_{18}H_{15}N_3O_2S$ [M+H]$^+$: 338.0958, found 338.0956.

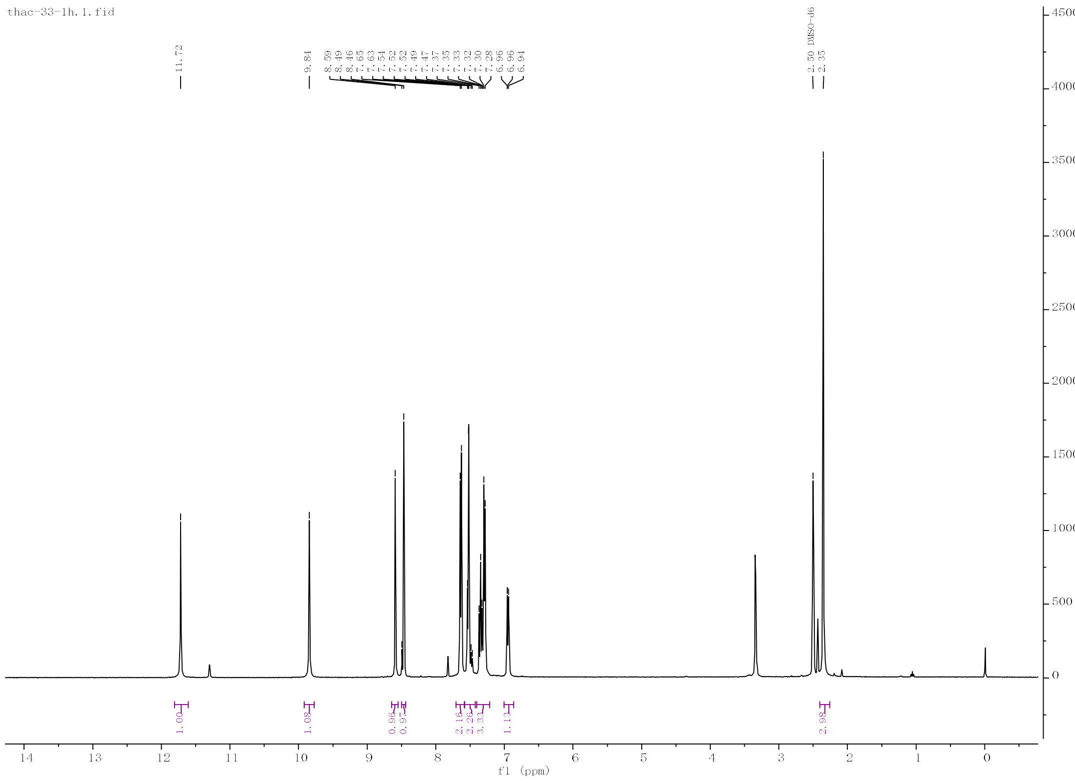

The ^{1}H NMR spectrogram of compound **E38**

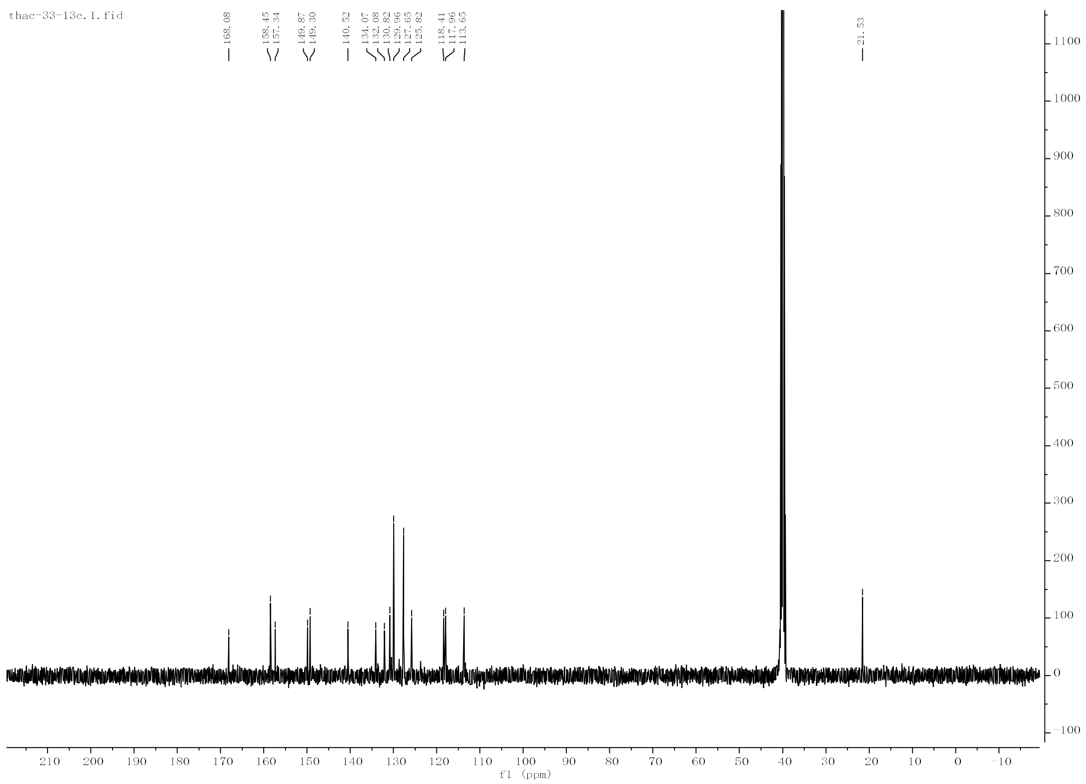

The ^{13}C NMR spectrogram of compound **E38**

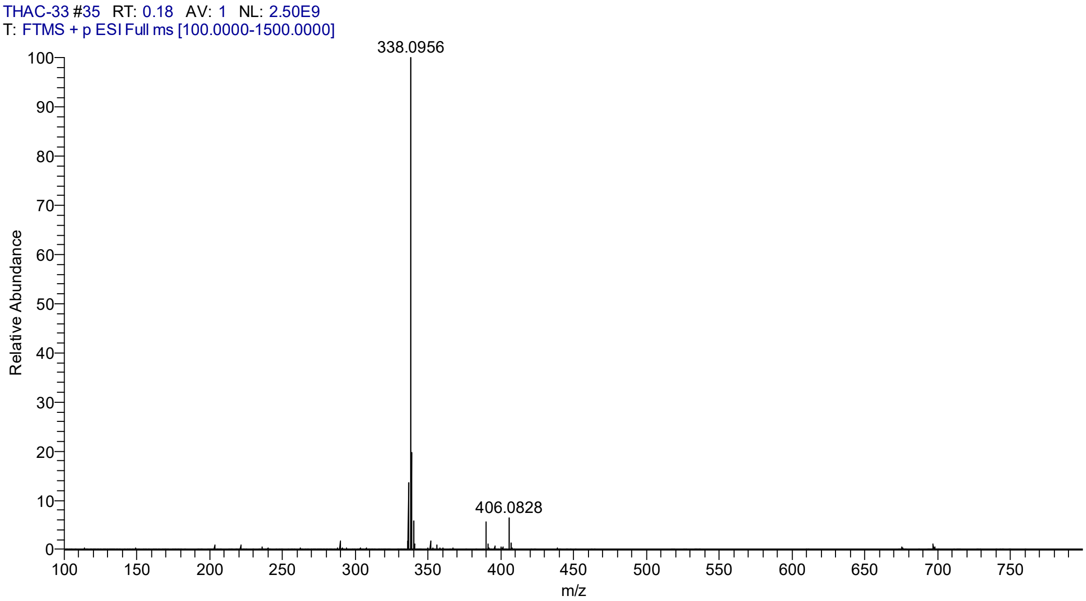

The HRMS spectrogram of compound **E38**

Compound **E39**,

2-(4-hydroxyphenyl)-*N'*-(4-methylbenzylidene)thiazole-4-carbohydrazide:

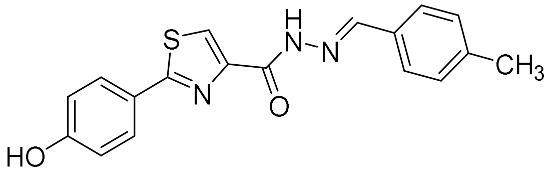

white solid, yield 65%, m.p. >220 °C; ^{1}H NMR (400 MHz, DMSO-d_6) δ 11.66 (s, 1H), 10.13 (s, 1H), 8.59 (s, 1H), 8.35 (s, 1H), 7.96 (d, J = 8.8 Hz, 2H), 7.63 (d, J = 8.0 Hz, 2H), 7.28 (d, J = 8.0 Hz, 2H), 6.92 (d, J = 8.8 Hz, 2H), 2.34 (s, 3H). ^{13}C NMR (101 MHz, DMSO-d_6) δ 168.34, 160.48, 157.40, 149.54, 149.23, 140.49, 132.09, 129.96, 128.91, 127.63, 124.51, 124.23, 116.36, 21.53. HRMS(ESI) calcd for $C_{18}H_{15}N_3O_2S$ $[M+H]^+$: 338.0958, found 338.0957.

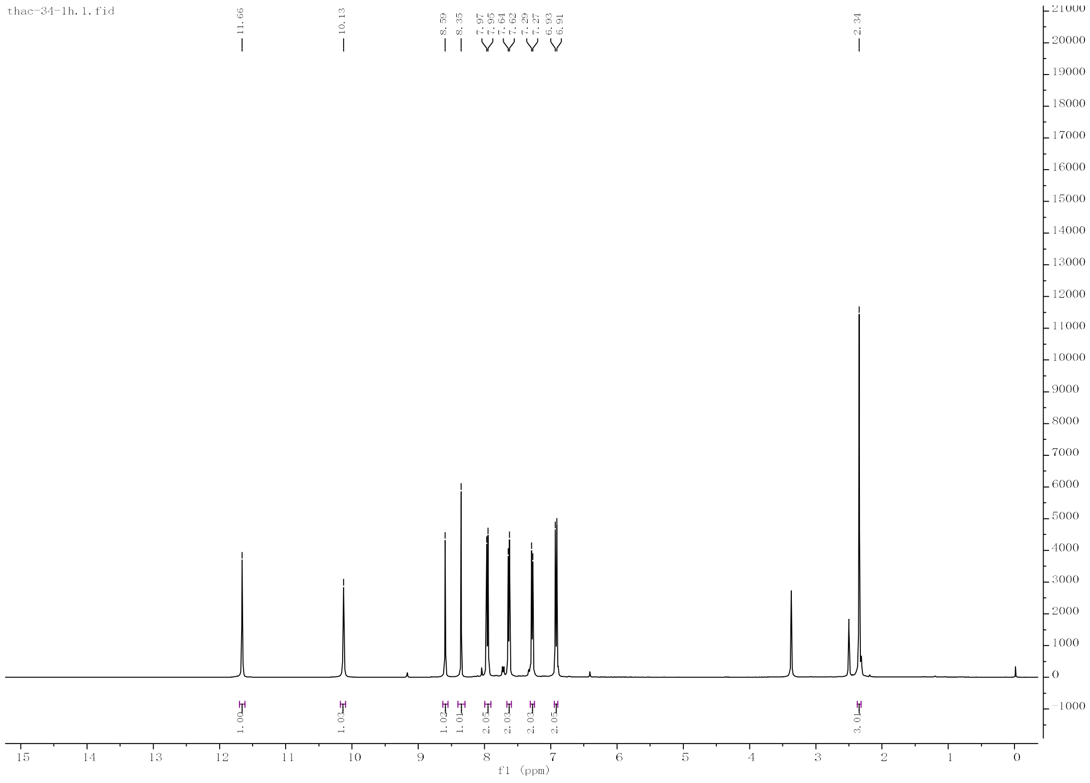

The ^{1}H NMR spectrogram of compound **E39**

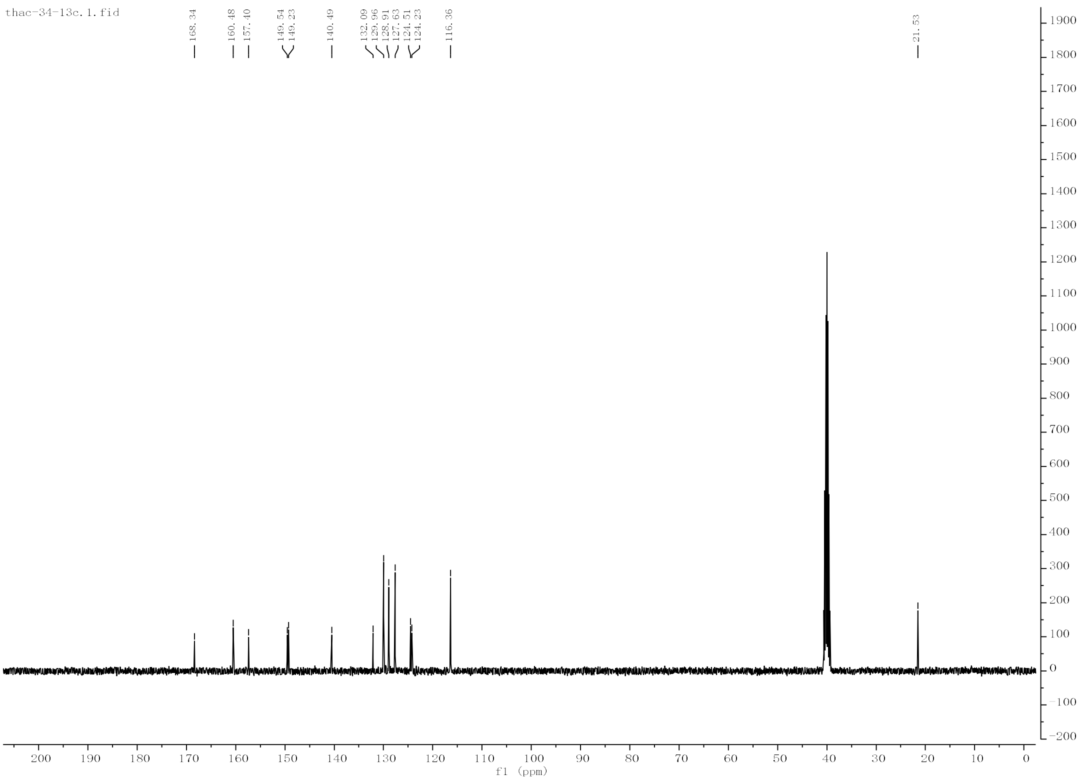

The ^{13}C NMR spectrogram of compound **E39**

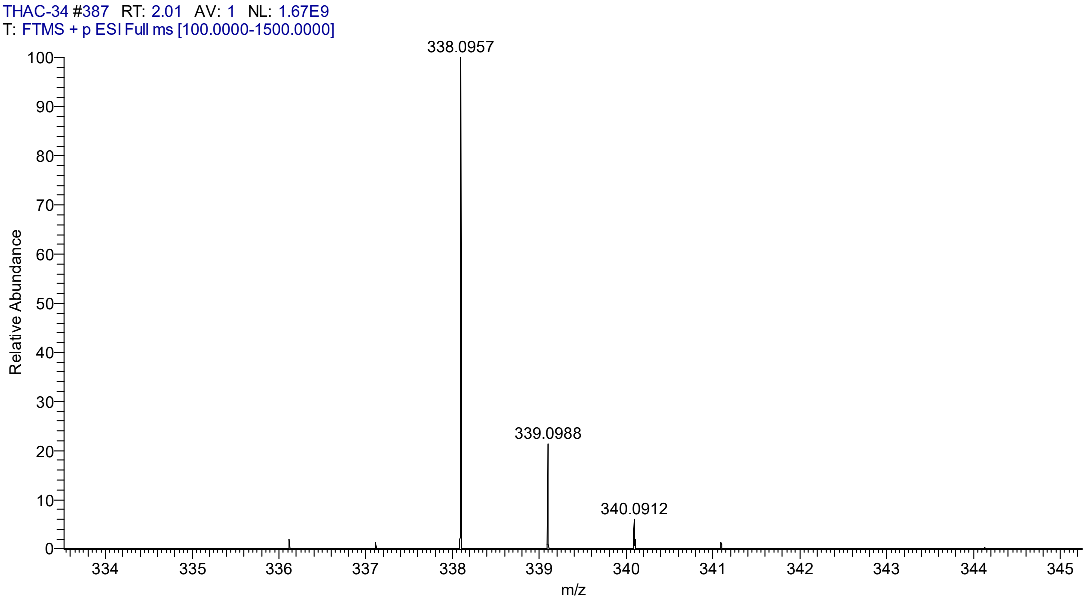

The HRMS spectrogram of compound **E39**

Compound **E40**,

2-(2-chloro-5-(trifluoromethyl)phenyl)-*N′*-(4-methylbenzylidene)thiazole-4-carbohydrazide:

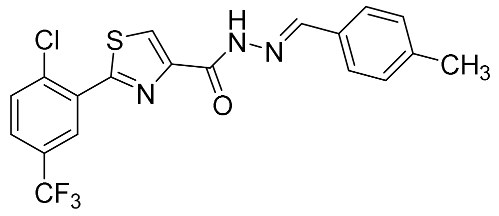

white solid, yield 65%, m.p. 187.6-189.1 °C; ^{1}H NMR (400 MHz, DMSO-d_6) δ 11.84 (s, 1H), 8.83 (d, J = 2.0 Hz, 1H), 8.70 (s, 1H), 8.62 (s, 1H), 7.95 – 7.87 (m, 2H), 7.65 (d, J = 8.0 Hz, 2H), 7.27 (d, J = 8.0 Hz, 2H), 2.35 (s, 3H). ^{13}C NMR (101 MHz, DMSO-d_6) δ 161.02, 156.62, 149.21, 148.55, 139.91, 134.96, 131.93, 131.48, 131.42, 129.45 (d, J = 14.8 Hz), 129.28, 128.51 (q, J = 32.9 Hz), 127.71 (d, J = 5.1 Hz), 127.64, 127.06, 123.43 (q, J = 272.7 Hz), 20.87. HRMS(ESI) calcd for $C_{19}H_{13}ClF_3N_3OS$ [M+H]$^+$: 424.0493, found 424.0491.

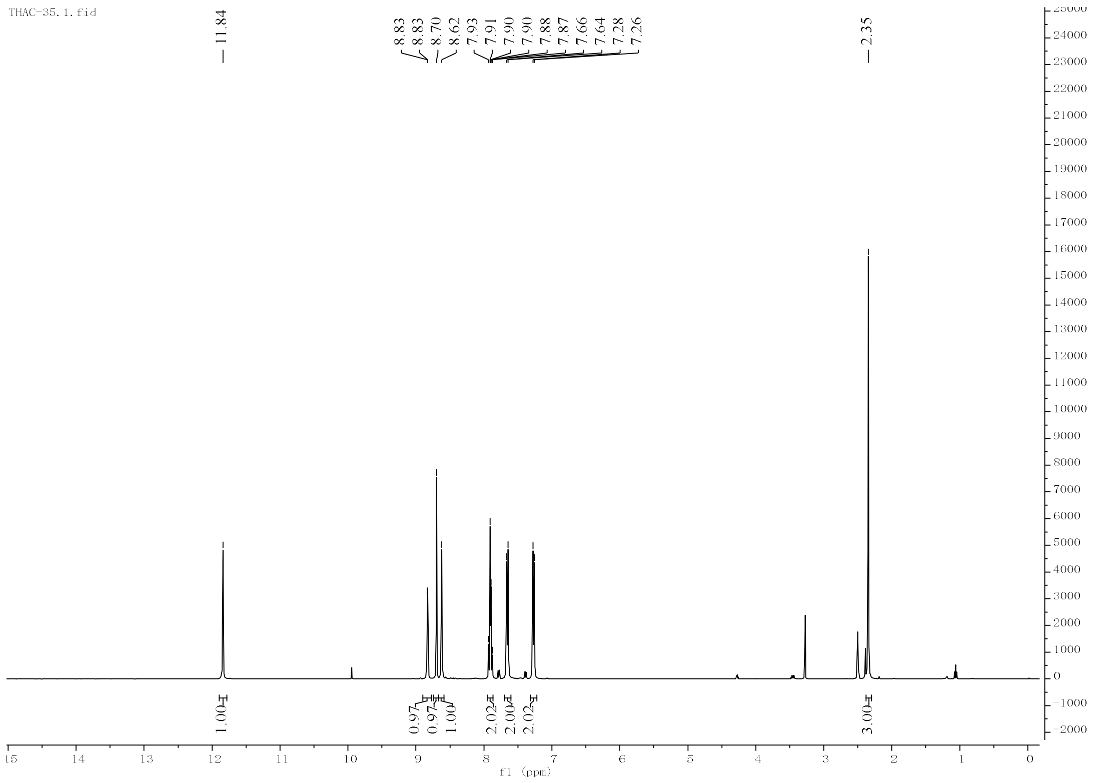

The ^{1}H NMR spectrogram of compound **E40**

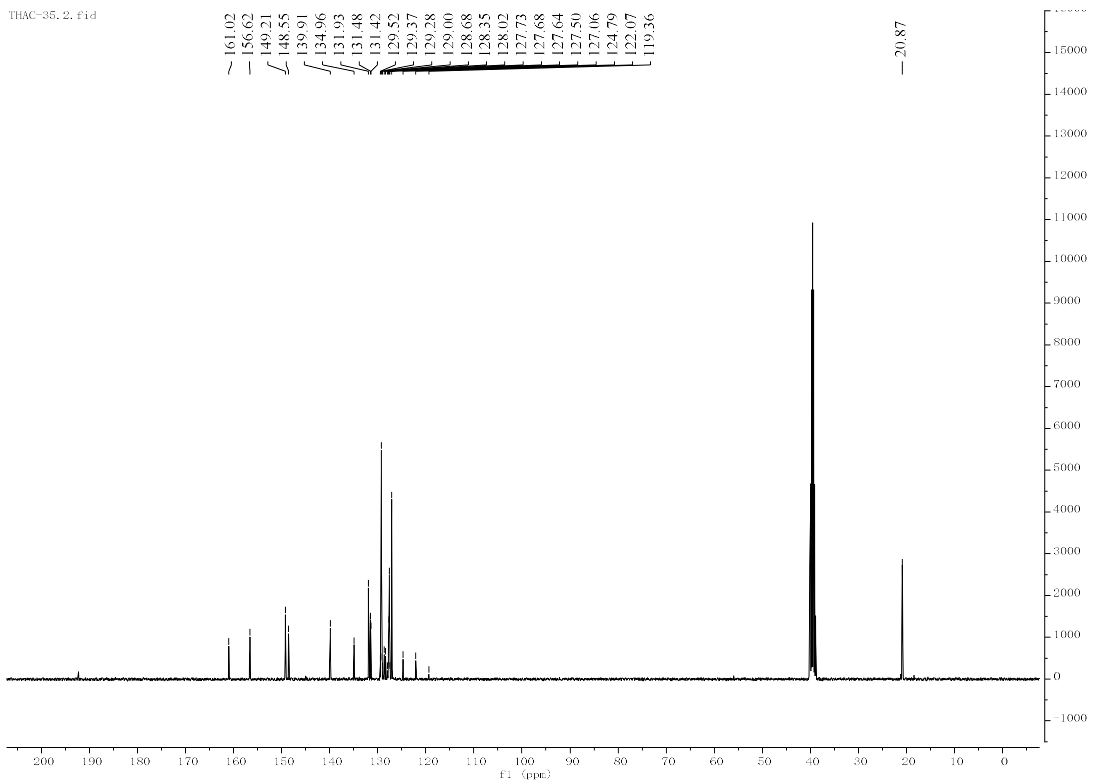

The ^{13}C NMR spectrogram of compound **E40**

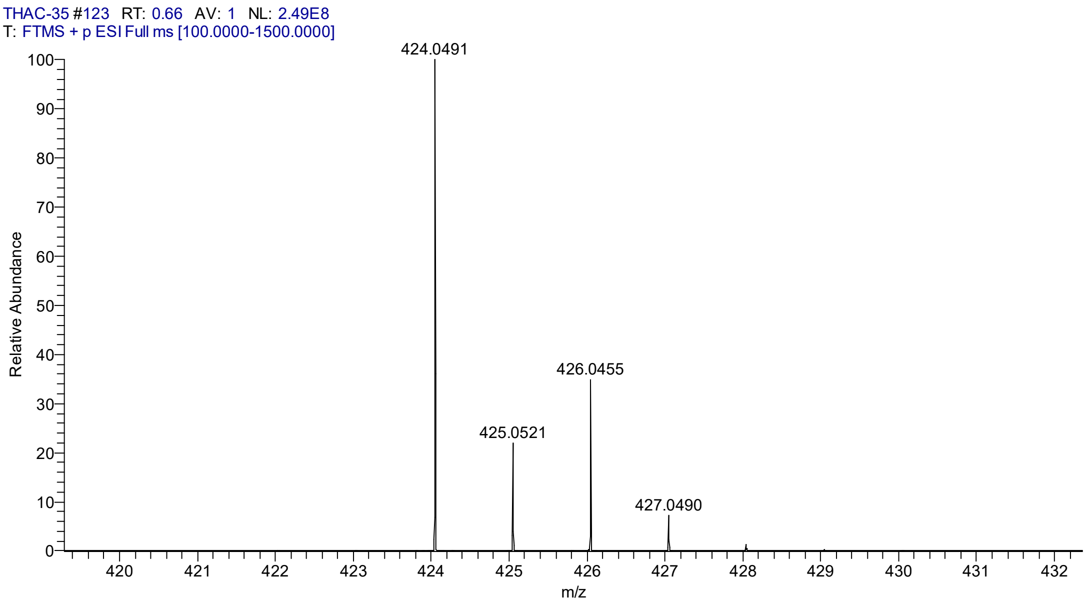

The HRMS spectrogram of compound **E40**

Compound **E41**,

2-(4-fluoro-2-methylphenyl)-*N'*-(4-methylbenzylidene)thiazole-4-carbohydrazide:

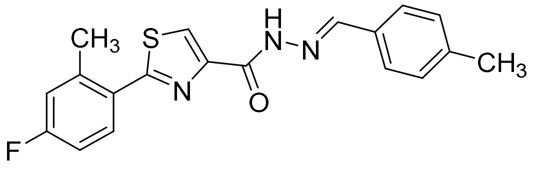

white solid, yield 74%, m.p. 157.4-159.5 °C; ^{1}H NMR (400 MHz, DMSO-d_6) δ 11.61 (s, 1H), 8.60 (s, 1H), 8.54 (s, 1H), 7.93 (dd, J = 8.8, 6.0 Hz, 1H), 7.62 (d, J = 8.0 Hz, 2H), 7.25 (d, J = 8.4 Hz, 3H), 7.18 (td, J = 8.4, 2.8 Hz, 1H), 2.59 (s, 3H), 2.33 (s, 3H). ^{13}C NMR (101 MHz, DMSO-d_6) δ 165.83, 162.60 (d, J = 248.3 Hz), 156.84, 148.91, 148.88, 139.86, 139.40 (d, J = 8.7 Hz), 132.08 (d, J = 9.0 Hz), 131.55, 129.30, 128.44 (d, J = 3.0 Hz), 127.05, 125.93, 117.83 (d, J = 21.6 Hz), 113.16 (d, J = 21.6 Hz), 20.97 (d, J = 1.4 Hz), 20.90. HRMS(ESI) calcd for $C_{19}H_{16}FN_3OS$ [M+H]$^+$: 354.1071, found 354.1071.

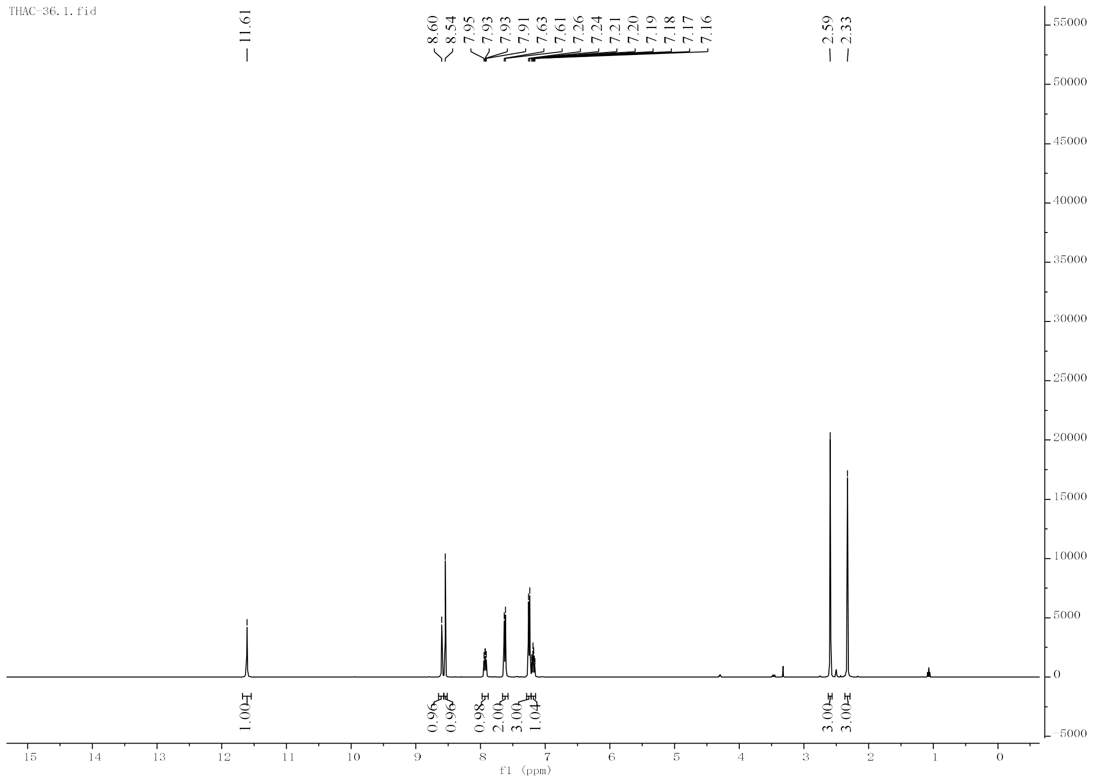

The ^{1}H NMR spectrogram of compound **E41**

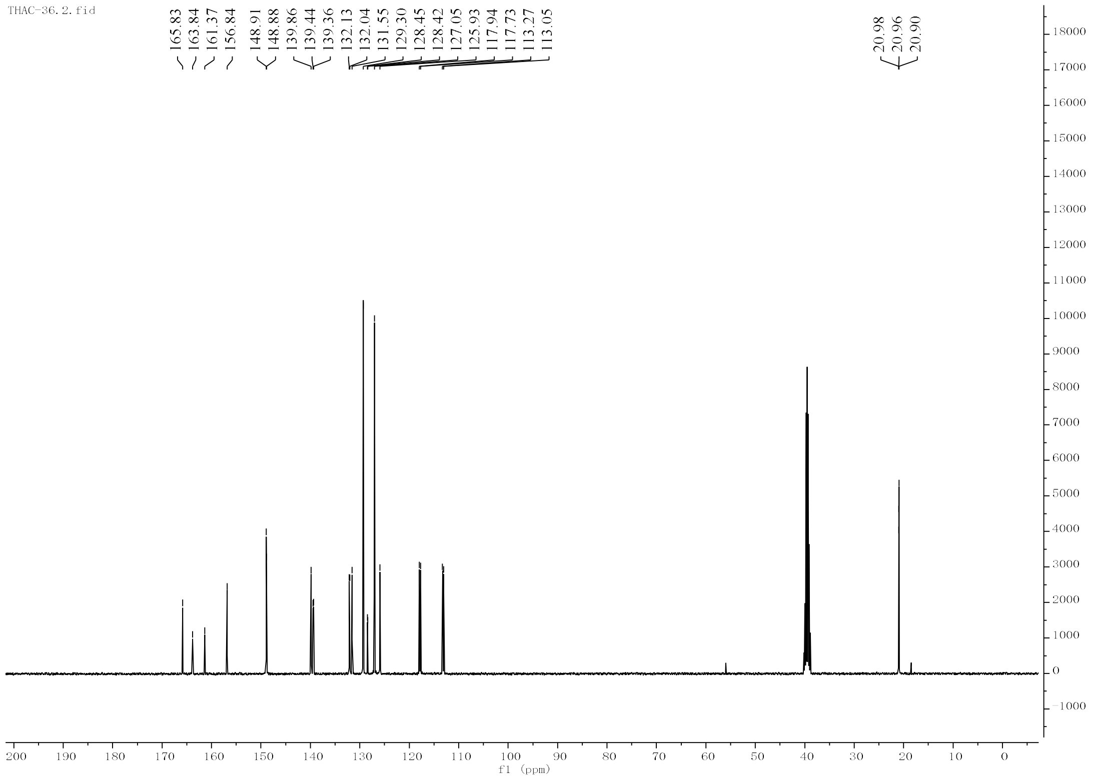

The ^{13}C NMR spectrogram of compound **E41**

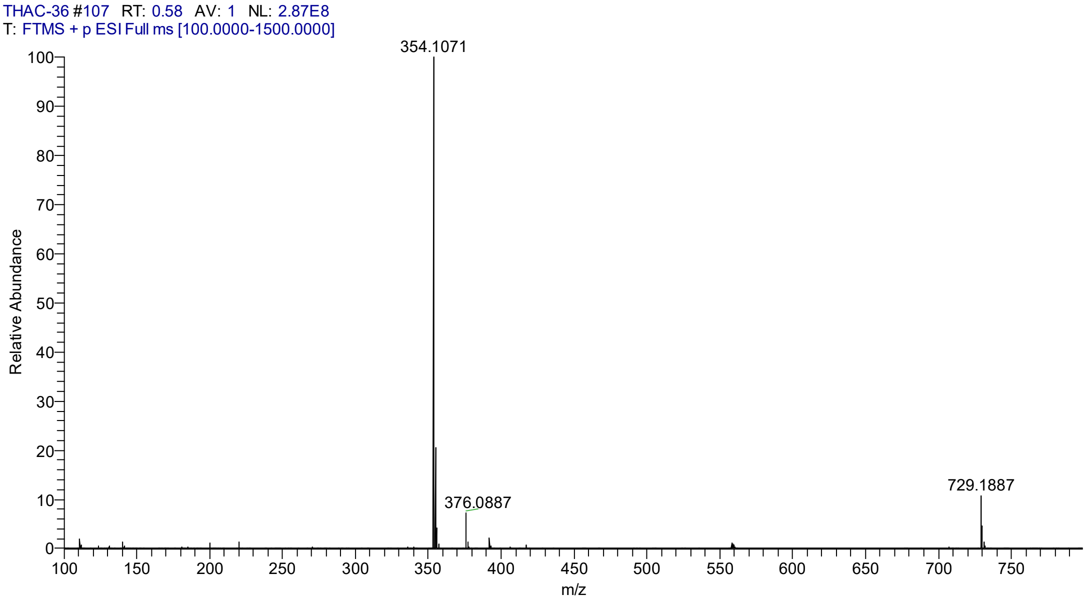

The HRMS spectrogram of compound **E41**

Compound **E42**, 2-(3,5-bis(trifluoromethyl)phenyl)-*N'*-(4-methylbenzylidene)thiazole-4-carbohydrazide

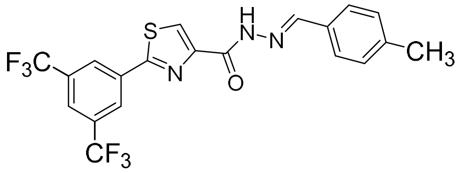

white solid, yield 66%, m.p. >220 °C; ^{1}H NMR (400 MHz, DMSO-d_6) δ 11.82 (s, 1H), 8.90 – 8.03 (m, 5H), 7.46 (d, J = 153.2 Hz, 4H), 2.34 (s, 3H). ^{13}C NMR (101 MHz, DMSO-d_6) δ 163.90, 156.52, 149.66, 149.27, 140.09, 134.71, 131.48, 131.26 (q, J = 33.2 Hz), 129.44, 127.62, 127.18, 127.11, 123.93 – 123.64 (m), 123.01 (q, J = 272.8 Hz), 21.01. HRMS(ESI) calcd for $C_{20}H_{13}F_6N_3OS$ [M+H]$^+$: 458.0756, found 458.0755.

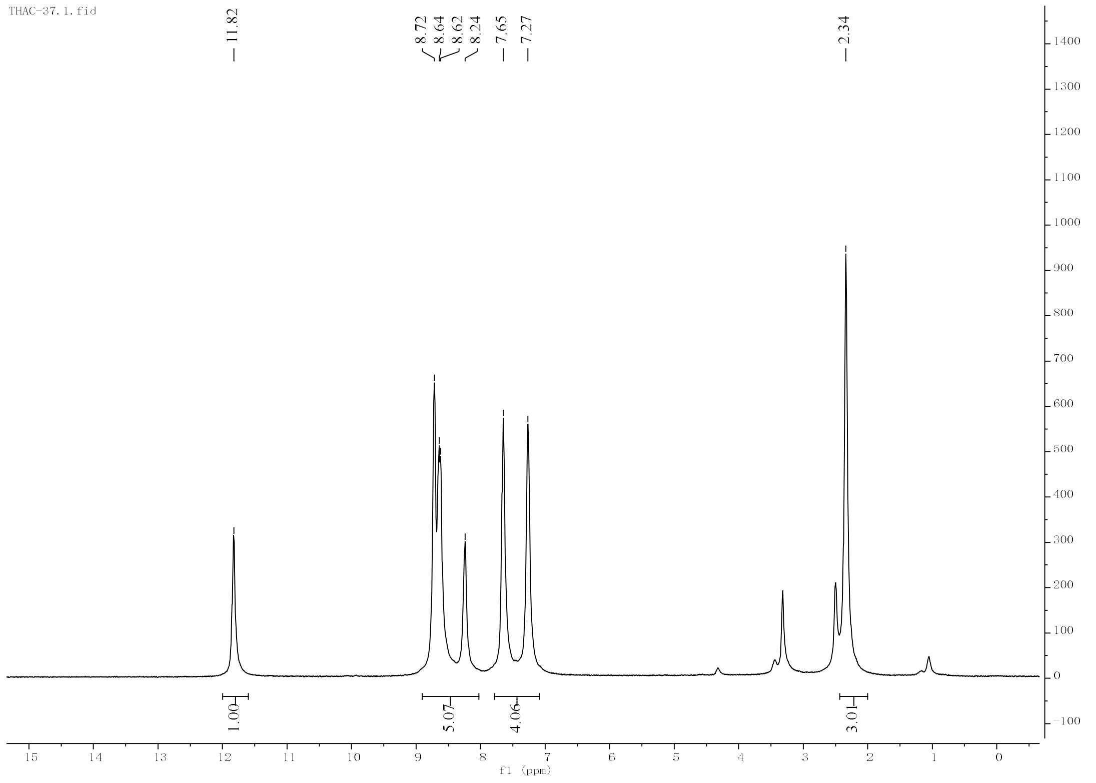

The ^{1}H NMR spectrogram of compound **E42**

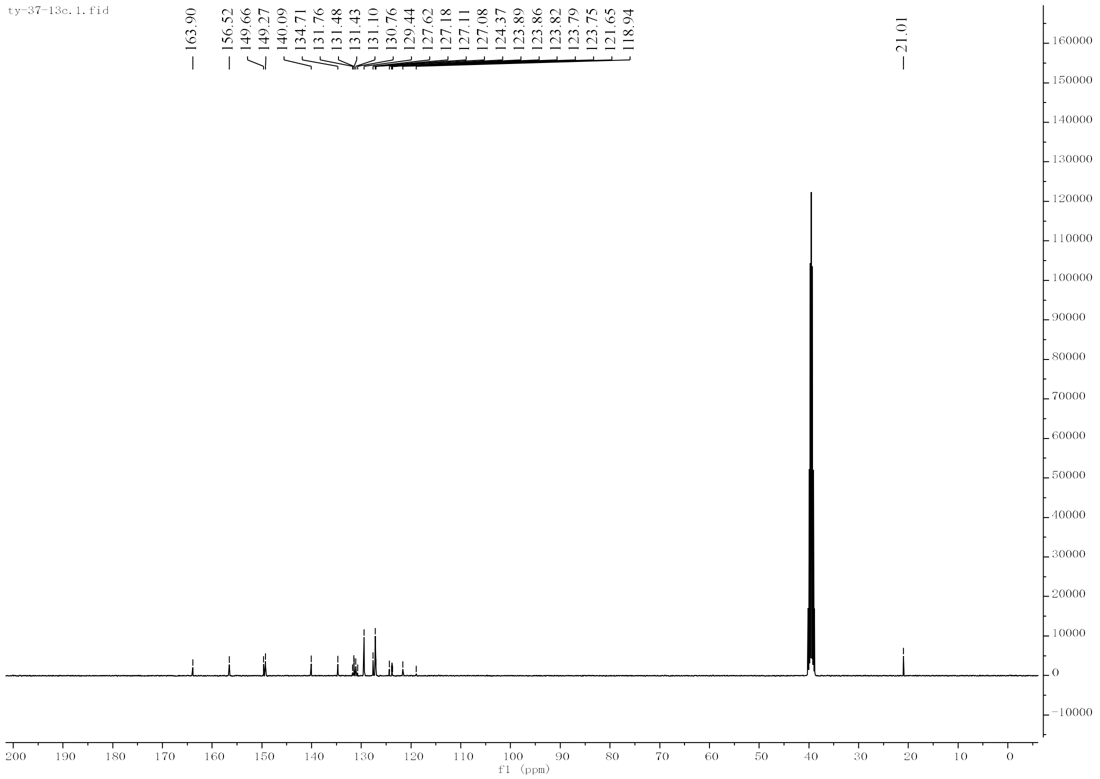

The ^{13}C NMR spectrogram of compound **E42**

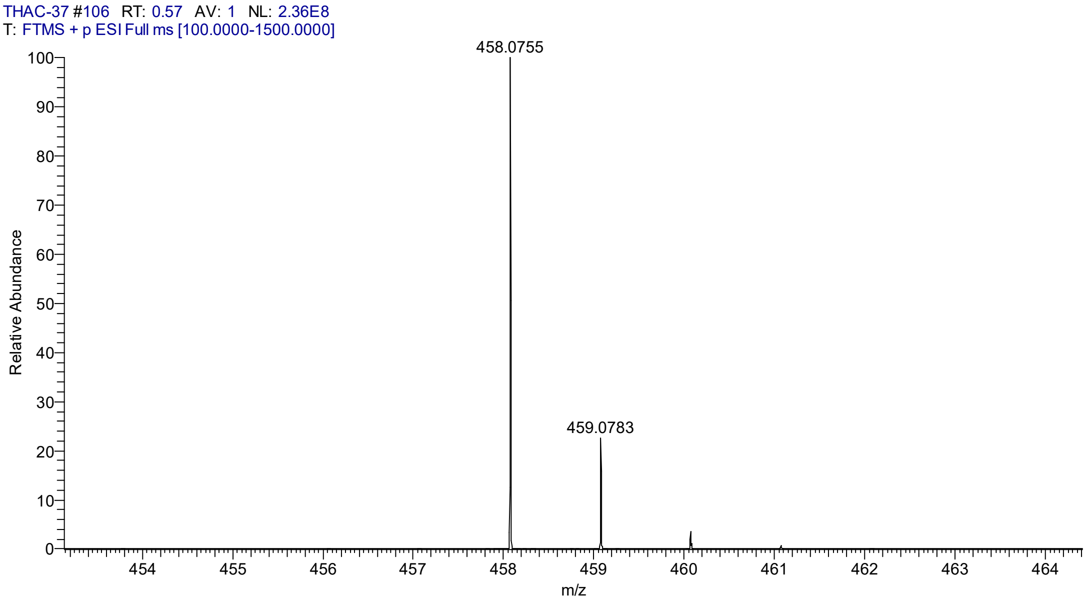

The HRMS spectrogram of compound **E42**

Compound **E43,**

2-(2,4-difluorophenyl)-*N'*-(4-methylbenzylidene)thiazole-4-carbohydrazide:

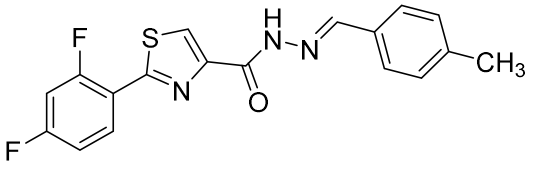

white solid, yield 67%, m.p. 181.6-183.5 °C; ^{1}H NMR (400 MHz, DMSO-d_6) δ 11.71 (s, 1H), 8.67 – 8.51 (m, 3H), 7.63 (d, J = 8.0 Hz, 2H), 7.47 (ddd, J = 11.6, 9.2, 2.4 Hz, 1H), 7.32 (td, J = 8.4, 2.4 Hz, 1H), 7.25 (d, J = 7=8.0 Hz, 2H), 2.32 (s, 3H). ^{13}C NMR (101 MHz, DMSO-d_6) δ 163.28 (dd, J = 251.7, 12.5 Hz), 159.68 (dd, J = 252.9, 12.7 Hz), 158.89 (d, J = 5.4 Hz), 156.61, 148.88, 148.34, 139.92, 131.49, 130.61 (dd, J = 10.2, 4.0 Hz), 129.32, 127.06, 126.13 (d, J = 8.6 Hz), 116.92 (dd, J = 11.8, 3.7 Hz), 112.61 (dd, J = 22.0, 3.2 Hz), 104.73 (t, J = 26.2 Hz), 20.91. HRMS(ESI) calcd for $C_{18}H_{13}F_2N_3OS$ [M+H]$^+$: 358.0820, found 358.0818.

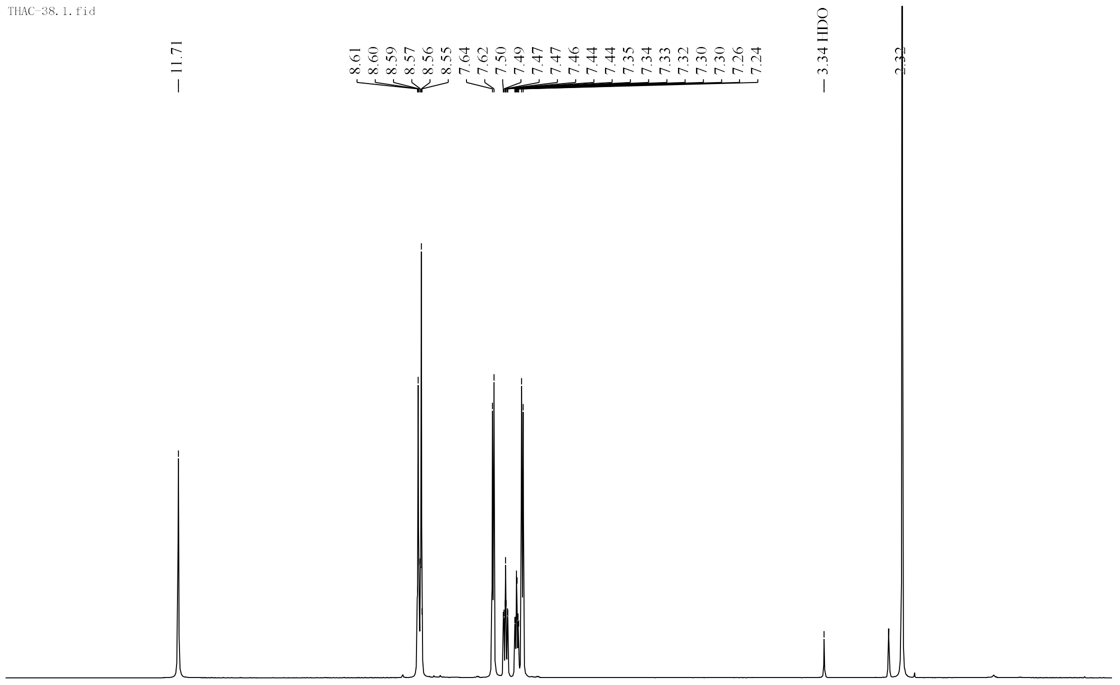

The ^{1}H NMR spectrogram of compound **E43**

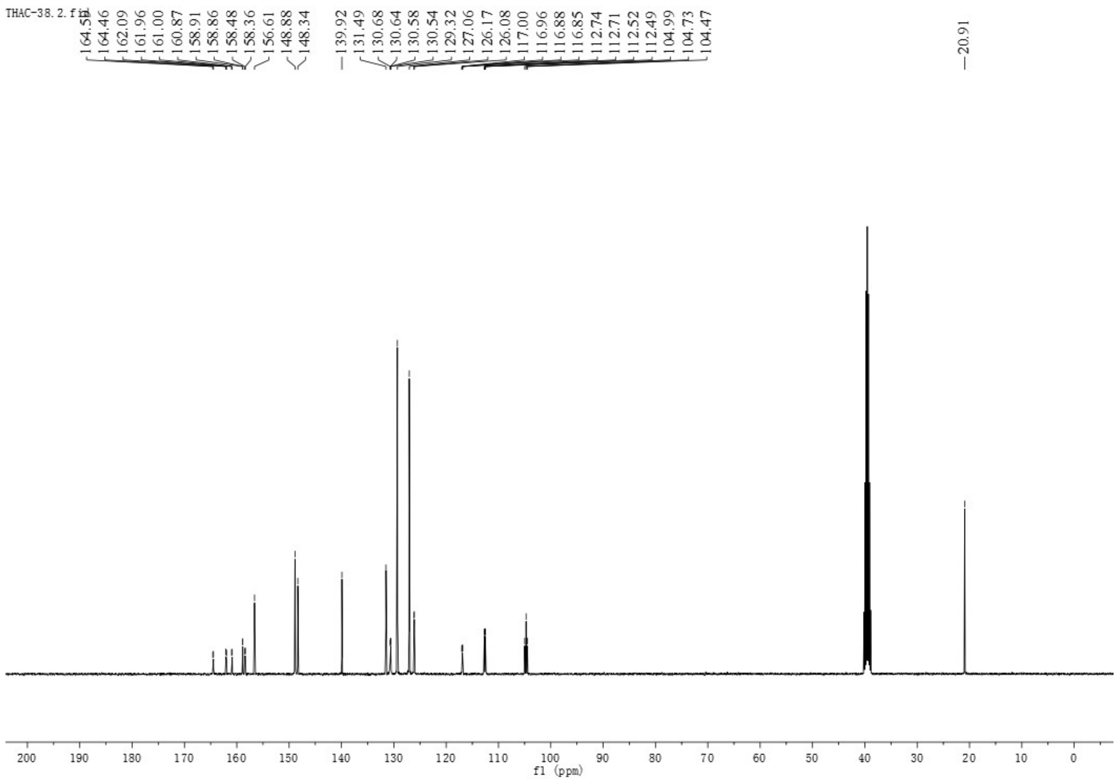

The ^{13}C NMR spectrogram of compound **E43**

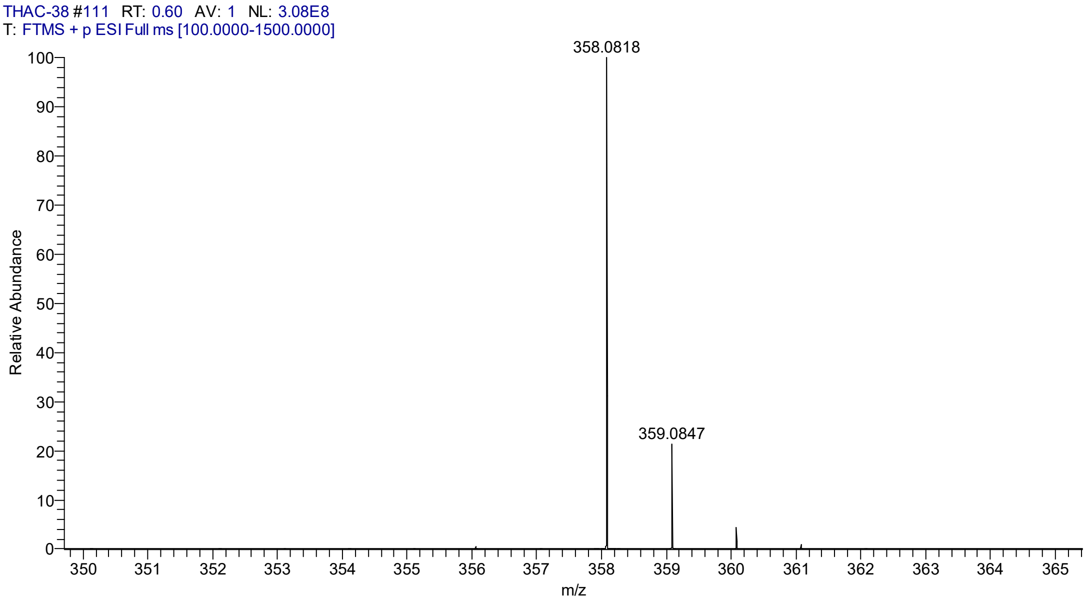

The HRMS spectrogram of compound **E43**

Compound **E44**, 2-(3-fluoro-4-methylphenyl)-*N'*-(4-methylbenzylidene)thiazole-4-carbohydrazide:

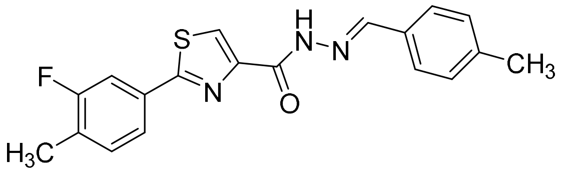

white solid, yield 68%, m.p. 186.1-187.7 °C; ^{1}H NMR (400 MHz, DMSO-d_6) δ 11.65 (s, 1H), 8.61 (s, 1H), 8.47 (s, 1H), 7.97 (dd, J = 10.8, 1.8 Hz, 1H), 7.80 (dd, J = 8.0, 2.0 Hz, 1H), 7.64 (d, J = 8.0 Hz, 2H), 7.45 (t, J = 8.0 Hz, 1H), 7.28 (d, J = 8.0 Hz, 2H), 2.35 (s, 3H), 2.31 (d, J = 2.0 Hz, 3H). ^{13}C NMR (101 MHz, DMSO-d_6) δ 165.94 (d, J = 3.2 Hz), 160.81 (d, J = 243.6 Hz), 156.57, 149.22, 148.85, 139.91, 132.33 (d, J = 5.2 Hz), 132.02 (d, J = 8.4 Hz), 131.49, 129.33, 127.19 (d, J = 17.6 Hz), 127.03, 125.47, 122.42 (d, J = 3.2 Hz), 112.62 (d, J = 24.4 Hz), 20.90, 14.03 (d, J = 3.2 Hz). HRMS(ESI) calcd for $C_{19}H_{16}FN_3OS$ [M+H]$^+$: 354.1071, found 354.1071.

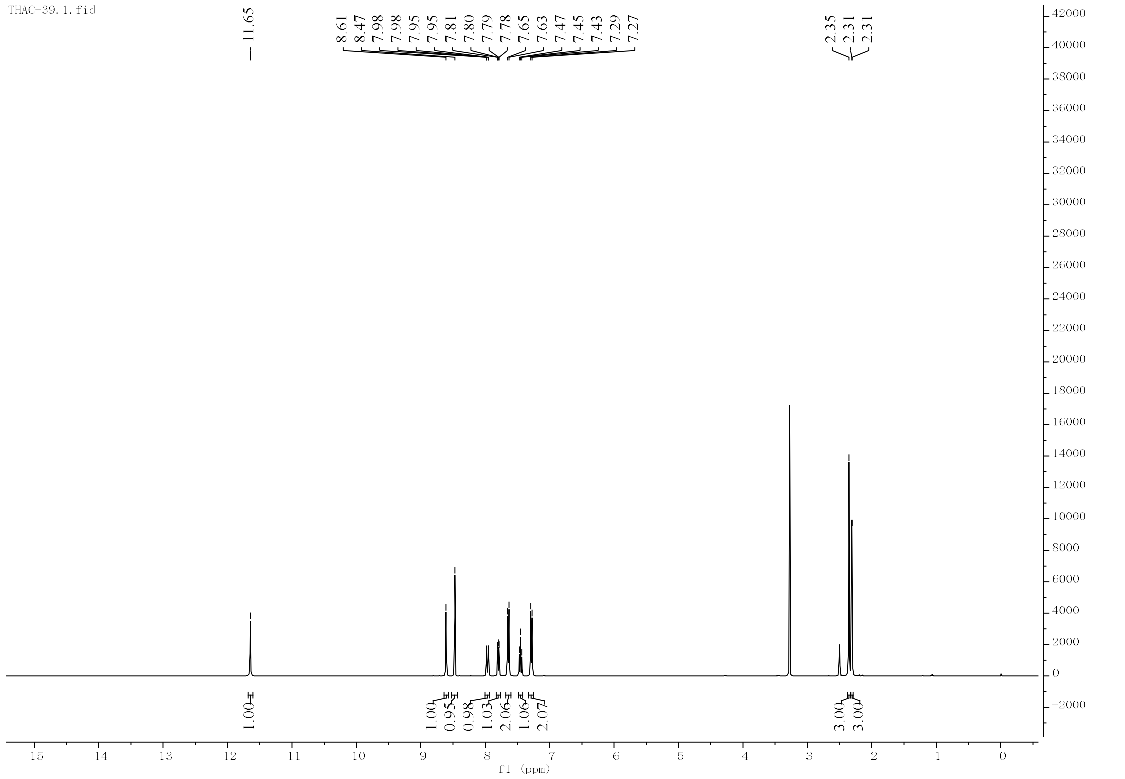

The ^{1}H NMR spectrogram of compound **E44**

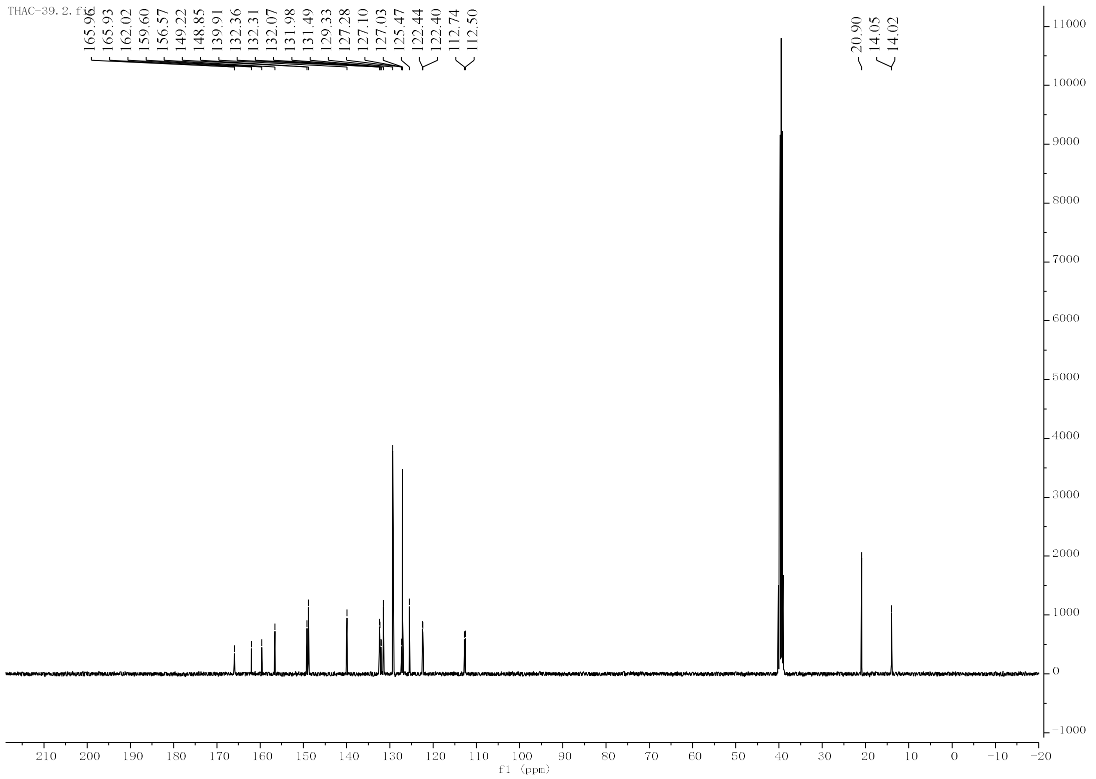

The ^{13}C NMR spectrogram of compound **E44**

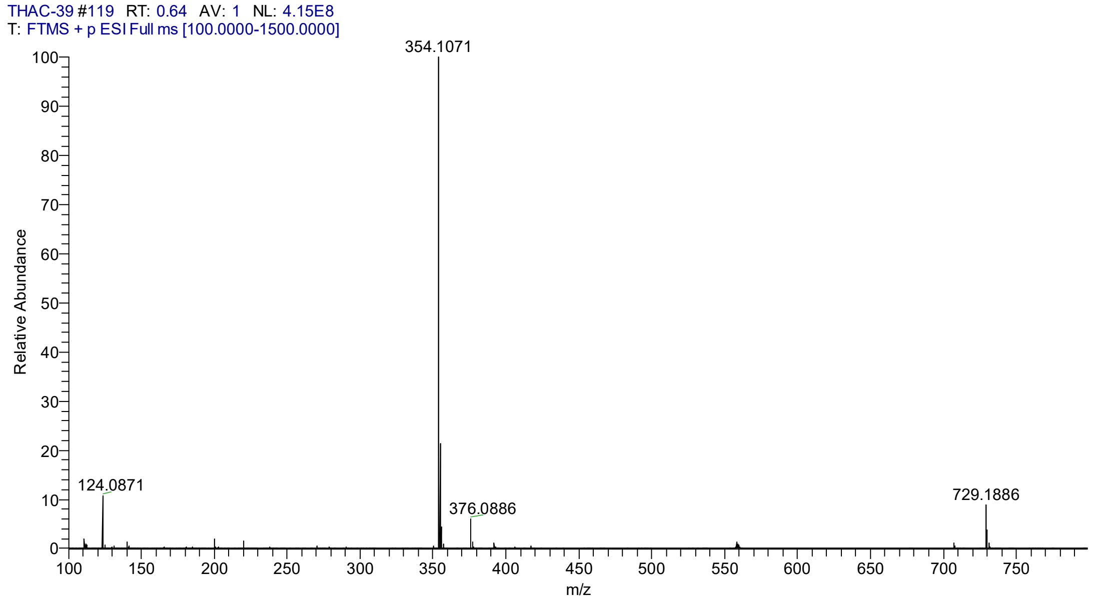

The HRMS spectrogram of compound **E44**

Compound **E45**, 2-(3,5-dimethylphenyl)-*N′*-(4-methylbenzylidene)thiazole-4-carbohydrazide:

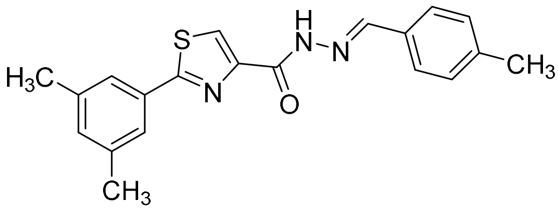

white solid, yield, 71%, m.p. 199.9-203.4 °C; ^{1}H NMR (400 MHz, DMSO-d_6) δ 11.70 (s, 1H), 8.62 (s, 1H), 8.45 (s, 1H), 7.72 (s, 2H), 7.64 (d, J = 7.6 Hz, 2H), 7.28 (d, J = 8.0 Hz, 2H), 7.16 (s, 1H), 2.35 (d, J = 8.0 Hz, 9H). ^{13}C NMR (101 MHz, DMSO-d_6) δ 168.31, 157.36, 149.86, 149.40, 140.52, 139.00, 132.80, 132.09, 129.95, 127.65, 125.76, 124.78, 21.52, 21.25. HRMS(ESI) calcd for $C_{20}H_{19}N_3OS$ [M+H]$^+$: 350.1322, found 350.1320.

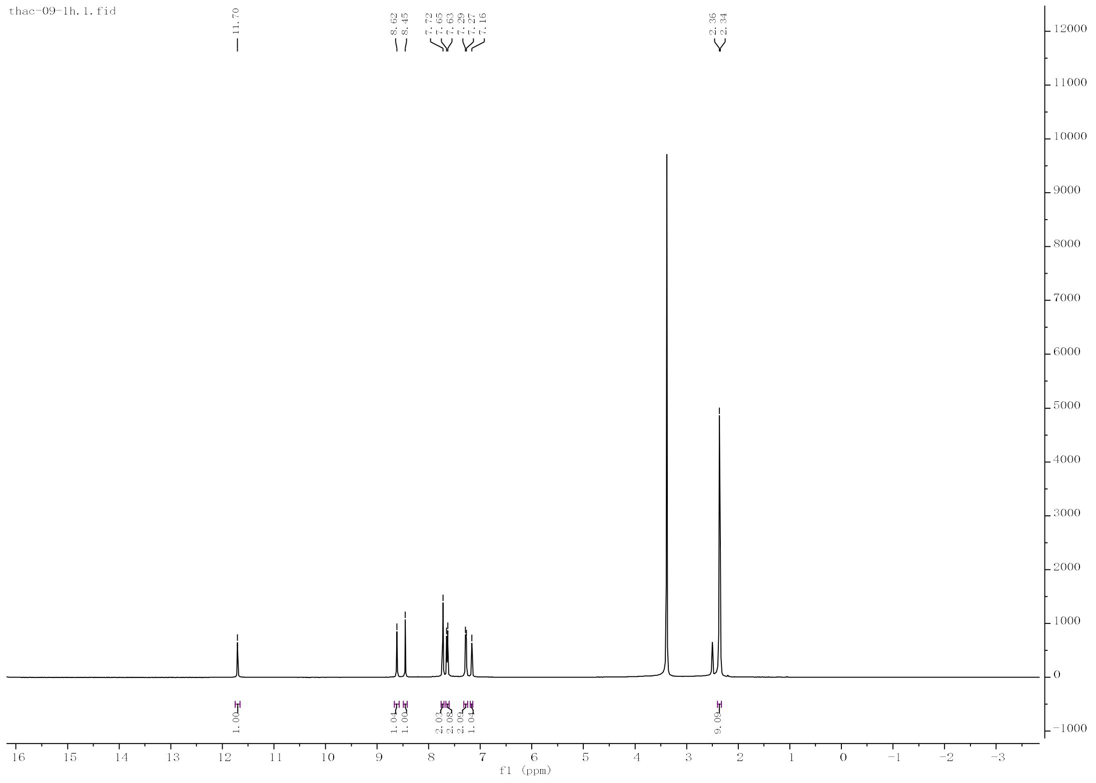

The ^{1}H NMR spectrogram of compound **E45**

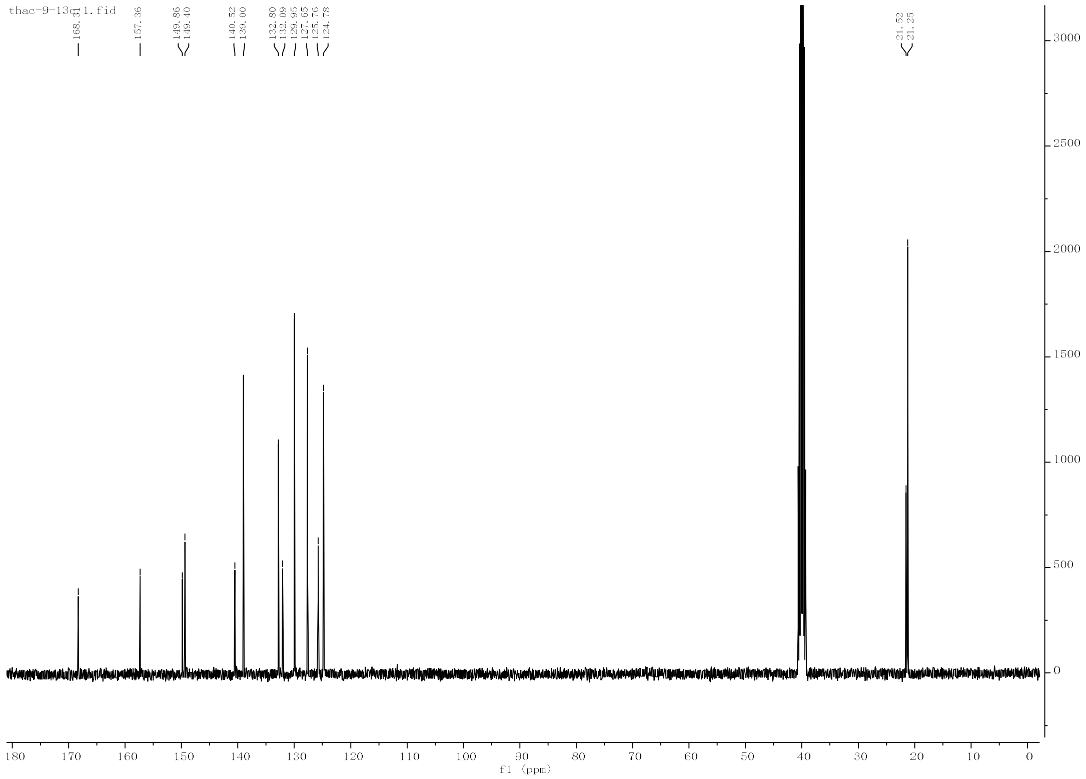

The ^{13}C NMR spectrogram of compound **E45**

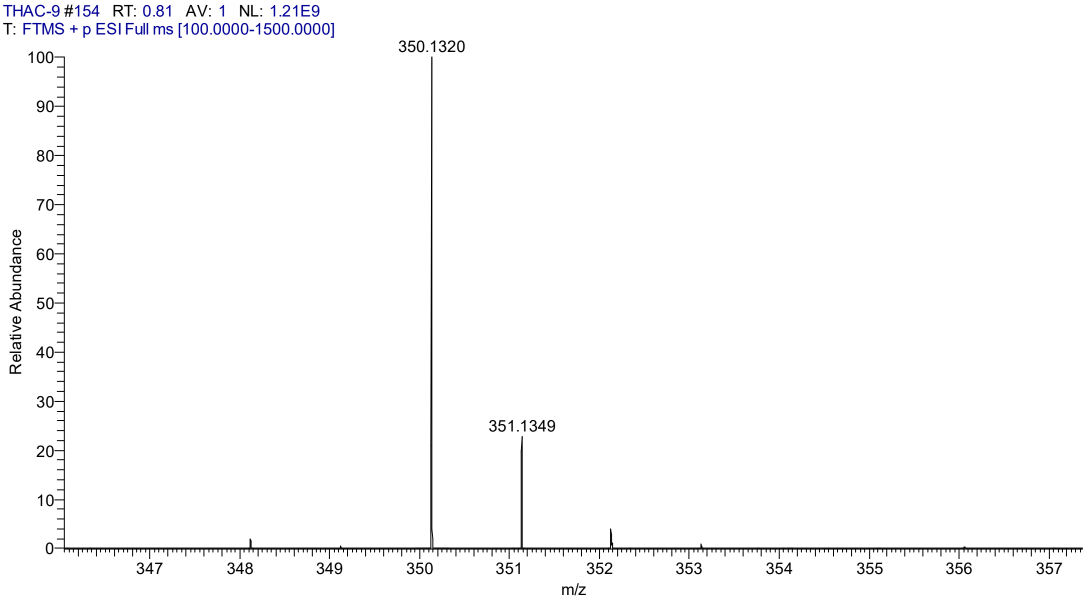

The HRMS spectrogram of compound **E45**
